# Defluoroalkylation of Trifluoromethane with Organolithium Reagents: Mechanism and Synthesis of Fluoroalkenes

**DOI:** 10.1002/anie.202516598

**Published:** 2025-11-06

**Authors:** Hodan R. Warsame, Sarah L. Patrick, James A. Bull, Philip W. Miller, Mark R. Crimmin

**Affiliations:** ^1^ Department of Chemistry Molecular Sciences Research Hub Imperial College London Shepherds Bush London W12 0BZ Unites Kingdom

**Keywords:** C–F activation, Defluoroalkylation, Fluoroalkene, Organolithium, Trifluoromethane

## Abstract

Trifluoromethane (HCF_3_, HFC‐23) is a byproduct of fluoropolymer production that has limited applications. It is often stored or destroyed at the point of production, but if released into the environment is a potent greenhouse gas with a global warming potential of 14 600 times that of CO_2_. State‐of‐the‐art chemical technologies for upgrading HCF_3_ typically occur with conservation of the CF_3_ group. These approaches will come under increased scrutiny as concern over the environmental impact of perfluoroalkyl substances (PFAS) continues to grow. A more sustainable approach involves synthetic transformations that repurpose the atomic content of HCF_3_ while also destroying the CF_3_ group. In this paper, we report a rare example of the transformation of HCF_3_ into a fluoroalkene functional group through defluoroalkylation. We rationalise product formation through DFT calculations, scale‐up the synthesis through continuous flow methods, and show that a fluoroalkene reagent derived from HCF_3_ is a competent nucleophile for the fluoroethenylation of a range of aldehydes.

## Graphical abstract

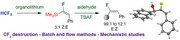

## Introduction

Trifluoromethane (HCF_3_, HFC‐23) is produced on a multi‐kilotonne scale during manufacture of poly(tetrafluoroethylene) (PTFE).^[^ ^1^ ^]^ The process for the synthesis of PTFE relies on first the fluorination of HCCl_3_ to form HCClF_2_ then coupling to generate CF_2_═CF_2_ which is polymerised. HCF_3_ forms as a by‐product of the fluorination step.^[^ ^2^ ^]^ While readily separated, HCF_3_ has limited value. Niche applications include use as a low temperature (−80 °C) refrigerant, a fire suppressant, and as a blanket gas in the semiconductor industry.^[^ ^3^ ^]^ The scale of these applications is vastly inferior to the scale of PTFE manufacturing, and so much of the HCF_3_ generated is either stored or destroyed at the point of production. HCF_3_ is environmentally damaging. It has a 100‐year global warming potential 14600 times greater than CO_2_. Despite legislation to phase‐down the use of HFCs, atmospheric concentrations of HCF_3_ are still rising slowly.^[^ ^4^ ^]^

**Figure 1:**
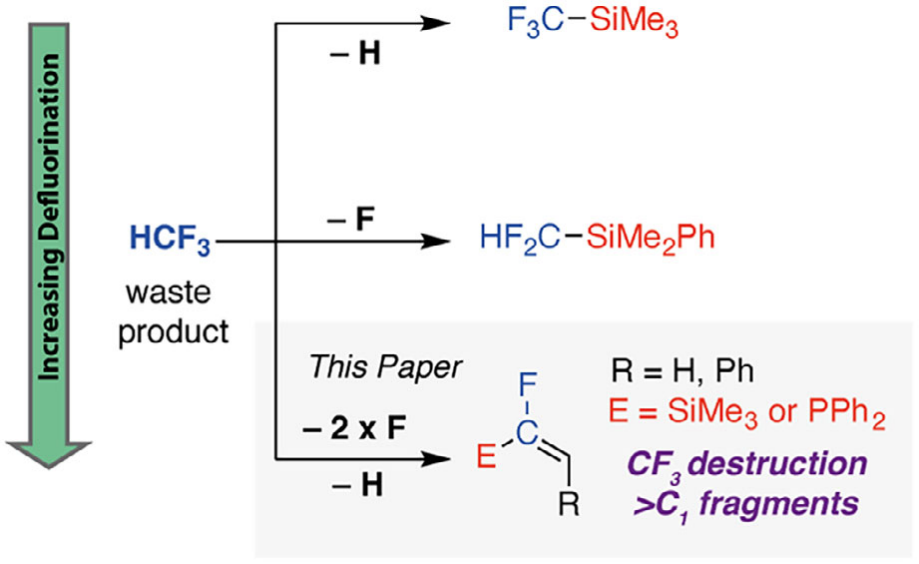
Transformation of HCF_3_ into added value fluorinated building blocks.

In recent years, the use of HCF_3_ as a simple and efficient precursor for trifluoromethylation^[^ ^5^, ^6^, ^7^ ^]^ and difluoromethylation^[^ ^8^, ^9^, ^10^, ^11^, ^12^, ^13^, ^14^, ^15^ ^]^ reactions has been realised (Figure 1). The former transformation occurs with a net loss of a hydrogen atom from HCF_3_, whereas the latter requires removal of a single fluorine atom. These processes allow HCF_3_ to be used as a C_1_ building block providing access to CF_3_ and CF_2_H groups which are attractive motifs in drug‐discovery due to their ability to modify lipophilicity, hydrogen‐bonding properties, and pharmacokinetics. While the CF_2_H group has emerged as an important bioisostere for the hydroxy group,^[^ ^16^ ^]^ there is increasing concern over use of CF_3_ groups due to the environmental fate of CF_3_ containing metabolites.^[^ ^17^, ^18^ ^]^

The generation of more complex, longer chain, fluorinated building blocks from HCF_3_ is incredibly rare, when compared to transformations to C_1_ building blocks. There are hints, however, that such transformations should be possible. For example, Mikami and coworkers have reported the formation of a fluoroalkene in modest yield from reaction of a lithium fluorenide with HCF_3_.^[^ ^13^ ^]^ 1,1‐Difluoroalkenes have also been observed as minor side products in the reaction of HCF_3_ with lithiated carbamates.^[^ ^14^ ^]^ If a general approach to generate longer chain, complex fluorinated C_n_ fragments from HCF_3_ could be achieved it might allow access to new medicinally valuable motifs or bioisosteres,^[^ ^19^ ^]^ not founded on the CF_3_ group, and open up new uses for this environmentally damaging waste‐product in drug‐discovery programs. Fluoroalkenes have been proposed as mimics for amide groups with *E* or *Z* isomers acting as surrogates of conformationally locked transoid and cisoid geometries of peptide linkages.^[^ ^20^ ^]^

In this study, we show that HCF_3_ reacts with organolithium reagents through an unusual defluoroalkylation pathway. The transformation occurs with the homologation and twofold defluorination of HCF_3_ to create a new substituted fluoroalkene motif (Figure 1). The protocol allows access to both phosphine and silyl substituted alkenes, and the latter products were demonstrated to be competent nucleophiles in the stereospecific fluoroethenylation of a range of aldehydes. Investigation of the mechanism by DFT calculations suggests that fluoroalkene formation occurs from a key organolithium intermediate and involves both an α‐elimination and 1,2‐migration step.

## Results and Discussion

**Figure 2:**
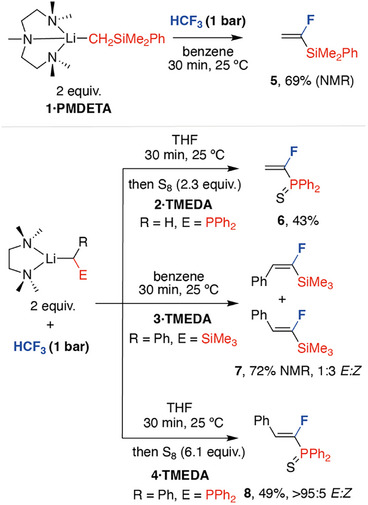
Defluoroalkylation of HCF_3_ by **1–4** to form fluoroalkene products.

**Figure 3:**
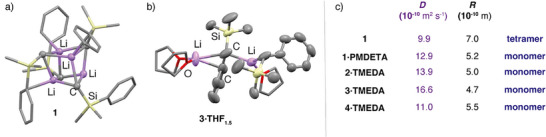
Solid state structure of a) **1** and b) **3·THF_1.5_** determined by single crystal X‐ray diffraction. c) Diffusion coefficients, D, determined by DOSY NMR spectroscopy in C_6_D_6_ solution, along with hydrodynamic radii, R, and most likely aggregation state.

**Figure 4:**
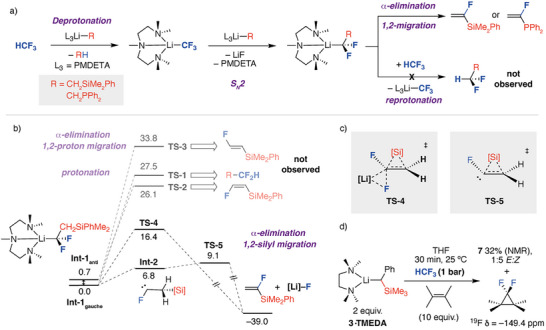
a) General pathway for defluorination of HCF_3_ via **Int‐1**. b) Onwards mechanisms from **Int‐1** calculated for reaction of HCF_3_ with **1·PMDETA** involving reprotonation, α‐elimination/1,2‐proton migration and α‐elimination/1,2‐silyl migration. c) Transition states for concerted and stepwise α‐elimination/1,2‐silyl migration pathways. c) Carbene trapping experiment. [Si] = SiMe_2_Ph, [Li] = Li·PMDETA. Gibbs free energies values in kcal mol^−1^ reported as single‐point corrected values with B3PW91‐D3/def2‐TZVPP/PCM(benzene)/GoodVibes.

A small array of phosphine and silyl substituted organolithium reagents (Figure 2 and 1–4) were prepared and their reactions with HCF_3_ were investigated.^[^ ^21^, ^22^, ^23^ ^]^ Following optimisation for the concentration, equivalents of HCF_3_, solvent, temperature, and the nature of the supporting ligand on lithium, it was discovered that **1·PMDETA** (PMDETA = pentamethylethylenetriamine) reacts quickly with excess (approx. 16 equiv) of HCF_3_ at 25 °C to selectively form CH_2_═CF(SiMe_2_Ph) (**5**) in 69% yield as measured by ^1^H NMR spectroscopy. **5** is characterised by a doublet of doublets observed at *δ* = −103.1 ppm (dd, *J* = 61.1, 32.2 Hz) in the ^19^F NMR spectrum. An HSQC experiment confirmed the proposed connectivity and geminal CH_2_ group of the terminal alkene. While **5** could be isolated, this compound is volatile, complicating its general utility. A similar reaction between **2·TMEDA** (TMEDA = tetramethylethylenediamine) and HCF_3_ led to the formation of CH_2_═CF(PPh_2_), which could be isolated as the corresponding phosphine sulfide **6** in 43% yield following oxidation with S_8_. It is notable that this reaction proceeds efficiently with only 1.1 equiv of HCF_3_ leading to much higher rates of gas destruction efficiency when compared with the silyl‐substituted nucleophile **1·PMDETA**.

The reaction of **3·TMEDA** with HCF_3_ also selectively generated fluoroalkene products, in this case a 1:3 mixture of *E/Z‐*PhCH═CF(SiMe_3_) (*E/Z*‐**7**) formed in 72% NMR yield. The major and minor stereoisomers were characterised by diagnostic resonances in the ^19^F NMR spectrum at *δ* = −106.1 ppm (d, ^3^ *J*_H–F_ = 35.6 Hz) and −113.5 ppm (d, ^3^ *J*_H–F_ = 52.2 Hz), respectively. The assignment of the connectivity and stereochemistry of the products was achieved through a combination of *J* values and computational prediction of ^19^F NMR chemical shift values (see Supporting Information for details). Ultimately, unambiguous assignment was achieved through a stereospecific proteodesilylation of the reaction mixture to generate a 3:1 mixture of *E*/*Z* PhCH═CFH and comparison against literature.^[^ ^24^ ^]^ In contrast, addition of the phosphine substituted analogue **4·TMEDA** to HCF_3_ (1.1 equiv) led to the formation of *E*‐PhCH═CF(PPh_2_) with higher selectively. The product was again isolated as the corresponding phosphine sulfide *E*‐**8** in 49% yield following oxidation with S_8_.

The mechanism to form fluoroalkene products from defluoroalkylation of HCF_3_ is clearly complex. It requires a 2:1 reaction stoichiometry of organolithium:HCF_3_ and elimination of 2 equiv of lithium fluoride. Moreover, the products form as a single constitutional isomer with both fluorine and silyl (or phosphine) substituents connected to the same carbon atom. Keen to understand this process further we sought to model potential reaction pathways through computational methods. To ensure a realistic chemical model, we first considered the aggregation state of the organolithium reagents in solution. α‐Heteroatom substituted organolithium reagents are known to crystallise as polymeric,^[^ ^25^ ^]^ hexameric,^[^ ^26^ ^]^ tetrameric,^[^ ^27^ ^]^ dimeric,^[^ ^28^, ^29^, ^30^ ^]^ or monomeric^[^ ^30^, ^31^, ^32^ ^]^ structures—depending on the nature of the supporting ligand on lithium. While **2·TMEDA** has previously been reported to be monomeric in the solid‐state,^[^ ^33^ ^]^ both dimeric and monomeric forms of **3·TMEDA** have been documented.^[^ ^34^, ^35^ ^]^ During this study, single crystals of **1** and **3·THF_1.5_** were obtained and shown to be tetrameric, dimeric in the solid‐state, respectively (Figure 3a,b). As it is not immediately obvious if the solid‐state structures persist in solution, DOSY studies on C_6_D_6_ NMR samples were conducted. The data support deaggregation in the presence of chelating ligands in solution. Hence, while **1** possesses a diffusion coefficient consistent with retention of tetrameric structures in solution, **1·PMDETA, 2·TMEDA**, **3·TMEDA** and **4·TMEDA** diffuse at faster rates and can largely be modelled as monomeric species (Figure 3c).^[^ ^36^ ^]^

Confident of the aggregation state of the organolithium reagent in solution, DFT calculations were carried out on the reaction of HCF_3_ with **1·PMDETA** to form **5** using the B3PW91‐D3 functional with energies corrected with the def2‐TZVPP basis‐set. Experimental conditions were modelled using GoodVibes correction. Previous computational studies have concluded that HCF_3_ reacts with organolithium,^[^ ^37^ ^]^ lithium silanides,^[^ ^38^ ^]^ and lithium boryl^[^ ^39^ ^]^ reagents to form difluoromethylation products through an initial deprotonation to generate a trifluoromethanide anion, which is subject to further S_N_2‐type nucleophilic attack by a second equivalent of the lithium reagent.

Based on this precedent, it was assumed that **1·PMDETA** reacts with HCF_3_ through an initial deprotonation and S_N_2 sequence to generate an organolithium intermediate (Figure 4a). Two staggered conformers of this intermediate, **Int‐1_anti_** and **Int‐1_gauche_**, were identified which were almost identical in energy (Δ*G*°_298 K_ = 0.7 kcal mol^−1^). **Int‐1_anti_** and **Int‐1_gauche_** differ by the orientation of the sterically demanding Li·PMDETA and SiMe_2_Ph substituents about the carbon–carbon bond, adopting *anti* and *gauche* conformations, respectively. A series of mechanisms were considered from these intermediates, leading to a diverse array of plausible reaction products (Figure 4b,c).

Protonation of **Int‐1** by HCF_3_ to form the difluoromethylation product was calculated to occur through **TS‐1** with an activation barrier of Δ*G* ^‡^_298 K_ = 27.5 kcal mol^−1^. This barrier is too high to be observed experimentally and essentially rules out formation of this product. Similarly, mechanisms that would lead to constitutional isomers of **5** with a vicinal relation between the F and Si atoms were ruled out based on high energy transition states. Hence, concerted steps involving α‐elimination of LiF and 1,2‐proton migration from **Int‐1** were located and give rise to a pair of stereoisomeric fluoroalkene products through **TS‐2** (Δ*G* ^‡^_298 K_ = 26.1 kcal mol^−1^) and **TS‐3** (Δ*G* ^‡^_298 K_ = 33.8 kcal mol^−1^).

Ultimately, two low energy pathways that give rise to the experimentally observed product **5** were found. The first involves a concerted α‐elimination and 1,2‐silyl group migration from **Int‐1_gauche_** occurring through the low energy transition state **TS‐4** (Δ*G* ^‡^_298 K_ = 16.4 kcal mol^−1^). **TS‐4** connected directly to the experimentally observed product **5** with extrusion of LiF·PMDETA (Δ*G*°_298 K_ = −39.0 kcal mol^−1^). The second is an analogous, but stepwise, pathway that first involves the generation of a defined carbene intermediate **Int‐2**. While we were unable to locate a transition state for the α‐elimination step, this is assumed to be a facile process. 1,2‐Silyl migration from **Int‐2** occurs through **TS‐5** (Δ*G* ^‡^_298 K_ = 9.1 kcal mol^−1^). Both these low energy pathways occur with evolution of carbene character at the site of α‐elimination. This carbene character is quenched by 1,2‐migration of the silyl group and formation of a C═C π─bond. In the transition states for 1,2‐migration, both the α‐ and β‐carbon deform toward sp^2^‐hybridisation. The migrating group adopts cationic character and is thus expected to follow known migratory aptitudes established for cationic 1,2‐rearrangements. NBO calculations evidence charge depletion at the migrating group through comparison of the NPA charges, supporting the hypothesis that this group adopts cationic character as the reaction proceeds (Tables S14–S16). The calculated mechanisms are reminiscent of that proposed for the Fritsch–Buttenberg–Wiechell rearrangement with the key difference being that **TS‐4** and **TS‐5** lead to construction of alkene rather than an alkyne functional group.^[^ ^40^, ^41^ ^]^

Both **TS‐4** and **TS‐5** would be accessible at 25 °C and based on our current understanding we believe that either (or both) mechanisms could be operational. Tentative support for the existence for the potential for the generation of carbene intermediates was provided by trapping experiments. Addition of 10 equiv of tetramethylethylene to the reaction of **3·TMEDA** with HCF_3_ in THF at 25 °C resulted in diminished yields of *E*/*Z*‐**7** along with observation of 1,1‐difluoro‐2,3,3,3‐tetramethylcyclopropane formed from [2 + 2] cycloaddition of difluoromethylene to the alkene (Figure 4d). This experiment does not directly evidence carbene formation from **Int‐1**, rather it suggests the proposed lithium trifluoromethanide intermediate [(κ^3^‐PMDETA)LiCF_3_] is prone to carbene formation. Nevertheless, the experiment provides circumstantial support for the potential of carbene generation through α‐fluoride elimination of the proposed organolithium intermediates.

**Figure 5:**
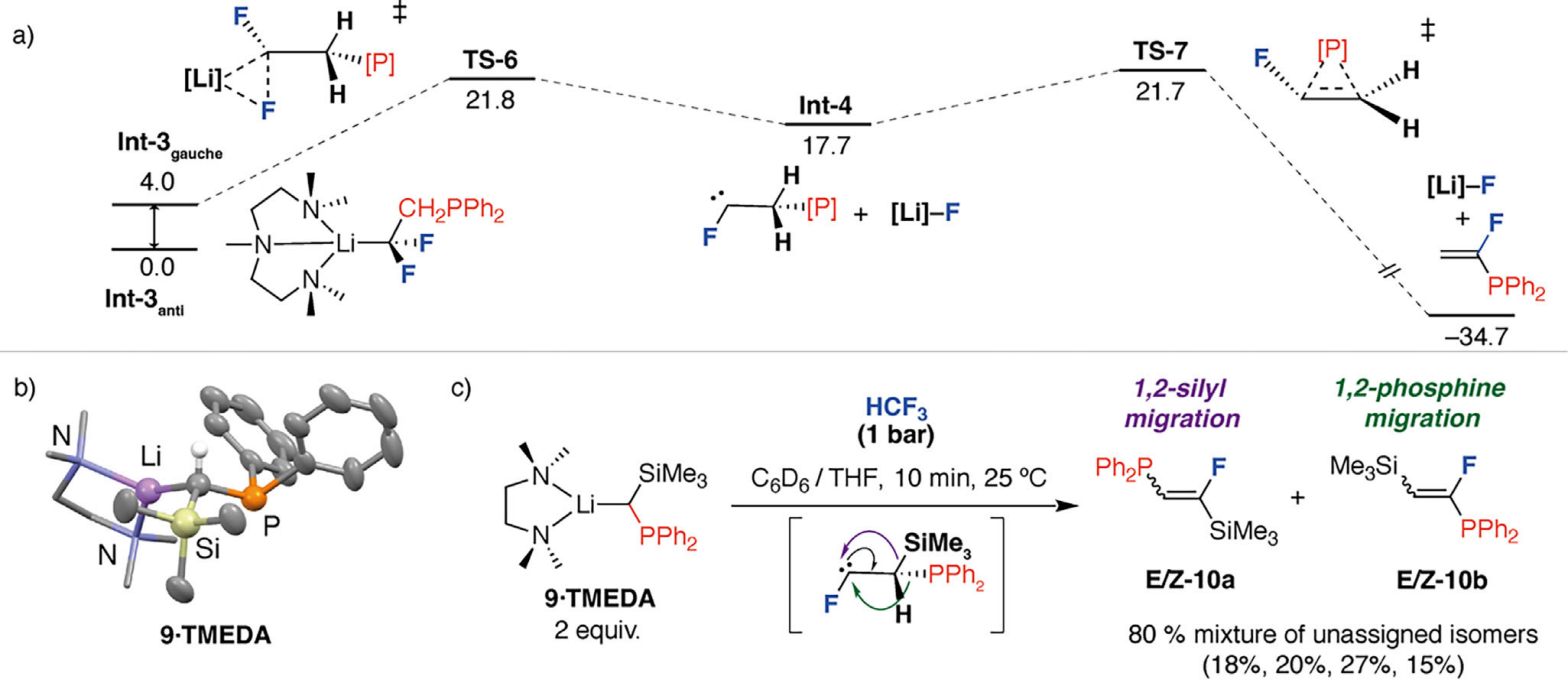
a) Stepwise pathway calculated for the reaction of **2·PMDETA** with HCF_3_. [Si] = SiMe_2_Ph, [Li] = Li·PMDETA. Gibbs free energies values in kcal mol^−1^ reported using B3PW91‐D3/def2‐TZVPP/PCM(benzene)/GoodVibes. b) Solid state structure of **9·TMEDA** determined by single crystal X‐ray diffraction. c) Reaction of **9·TMEDA** with HCF_3_ to form a mixture of 1,2‐silyl and 1,2‐phosphine migration products.

Further calculations were undertaken on the reaction of the α‐phosphine substituted nucleophile **2** with HCF_3_. For consistency and to enable a point of comparison to the pathways described above **2·PMDETA** was used as a model (Figure 5a). In this case, the anti‐isomer **Int‐3_anti_** was found to be lower than the gauche isomer **Int‐3_gauche_** (Δ*G*°_298 K_ = 4.0 kcal mol^−1^). A low energy pathway for fluoroalkene formation was again identified, but in this case only the stepwise α‐elimination and 1,2‐phosphine migration pathway could be found computationally. Evolution of **Int‐3_anti_** to a carbene intermediate **Int‐4** occurs via **TS‐6** (Δ*G* ^‡^_298 K_ = 21.8 kcal mol^−1^), this is then followed by the 1,2‐migration of the phosphine group to the carbene centre with concurrent formation of the C═C double bond by **TS‐7** (Δ*G* ^‡^_298 K_ = 21.7 kcal mol^−1^).

The local barriers for silyl and phosphine group migration from the putative carbene intermediates **Int‐2** and **Int‐4** are extremely low with Δ*G* ^‡^_298 K_ = 2.3 kcal mol^−1^ and Δ*G* ^‡^_298 K_ = 4.0 kcal mol^−1^, respectively, suggesting that both these groups have high migratory aptitude. To further probe this an intramolecular competition experiment was designed. The organolithium reagent **9·TMEDA** containing both silyl and phosphine groups was prepared. This species was crystallographically characterised and found to be monomeric in solid‐state (Figure 5b). DOSY studies in C_6_D_6_ give a diffusion coefficient of 11.2 x 10^−10^ m^2^ s^−1^ corresponding to a hydrodynamic radius of 5.1 Å, consistent with the monomeric form persisting in solution. Reaction of **9·TMEDA** with HCF_3_ in a mixture of C_6_D_6_ and THF for 10 min at 25 °C, gave **10a** and **10b** both as a mixture of *E*/*Z* stereoisomers (Figure 5c). All four products of this reaction are formed in a near equal ratio, suggesting that there is no significant preference for the migration of one group over another, likely due to the remarkably similar migratory aptitude of the SiR_3_ and PR_2_ groups.

The low barriers for silyl or phosphine group migration also provides and explanation for the modest stereocontrol in the formation of *E*/*Z*‐**7** and *E*/*Z*‐**8**. In both cases the *E*/*Z*‐ratio will be controlled by the geometry of the 1,2‐migration transition states. The low stereodifferentiation, and switch of major isomer with different organolithium reagents, suggests that the isomers of these transition states are likely very close in energy. This hypothesis is supported by DFT calculations on the stepwise pathway to form *E*/*Z*‐**7** via a carbene intermediate which suggest only a small energy difference of ΔΔ*G* ^‡^_298 K_ = 0.4 kcal mol^−1^ between transition states that lead to the *E* and *Z* isomers (see Supporting Information). Further experiments demonstrate that product ratios are sensitive to the nature of the amine ligand on lithium, solvent and reaction stoichiometry and can vary from 1:1.6 to 1:5.7 for formation of *E*/*Z*‐**7** and 5.7:1 to 99:1 for *E*/*Z*‐**8** (see Supporting Information).

**Figure 6:**
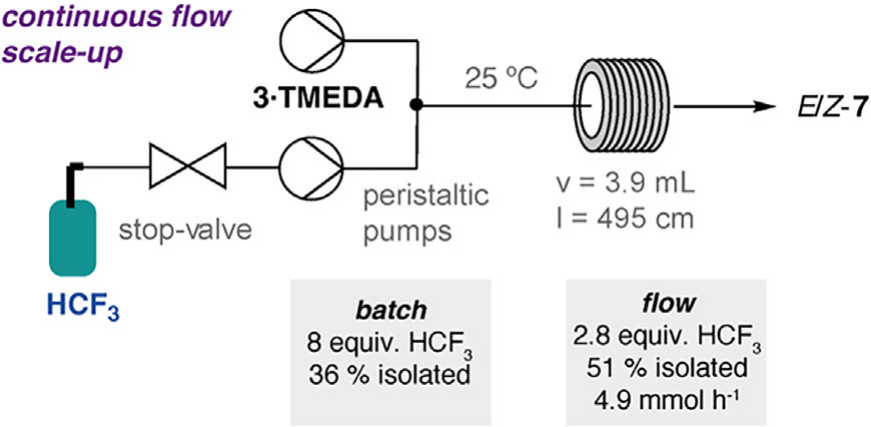
Flow setup for the synthesis of *E*/*Z*‐**7** from HCF_3_ along with comparison of optimised conditions for batch and flow processes.

The synthesis of a fluoroalkene product from HCF_3_ was scaled‐up using flow chemistry. Specifically, the production of *E/Z*‐**7** was targeted as this product is nonvolatile, bench‐stable, and potentially a useful reagent to install the fluoroalkene motif into organic molecules. Both the yield and gas destruction efficiency of the reaction could be improved by operating in flow when compared to attempts to scale‐up in batch.^[^ ^42^ ^]^ Application of the optimum process conditions to the defluoroalkylation reaction of **3·TMEDA** with HCF_3_ yielded a combined 67% (51% isolated) of a 1:3 mixture of *E*/*Z*‐**7** with just 2.8 equiv of gas, at a production rate of 4.9 mmol h^−1^ (Figure 6). This approach provided material on modest scales (100 mg–1 g), with the potential to scale further through increasing the process time, and allowed the exploration of onwards chemistry of *E/Z*‐**7**.

**Figure 7:**
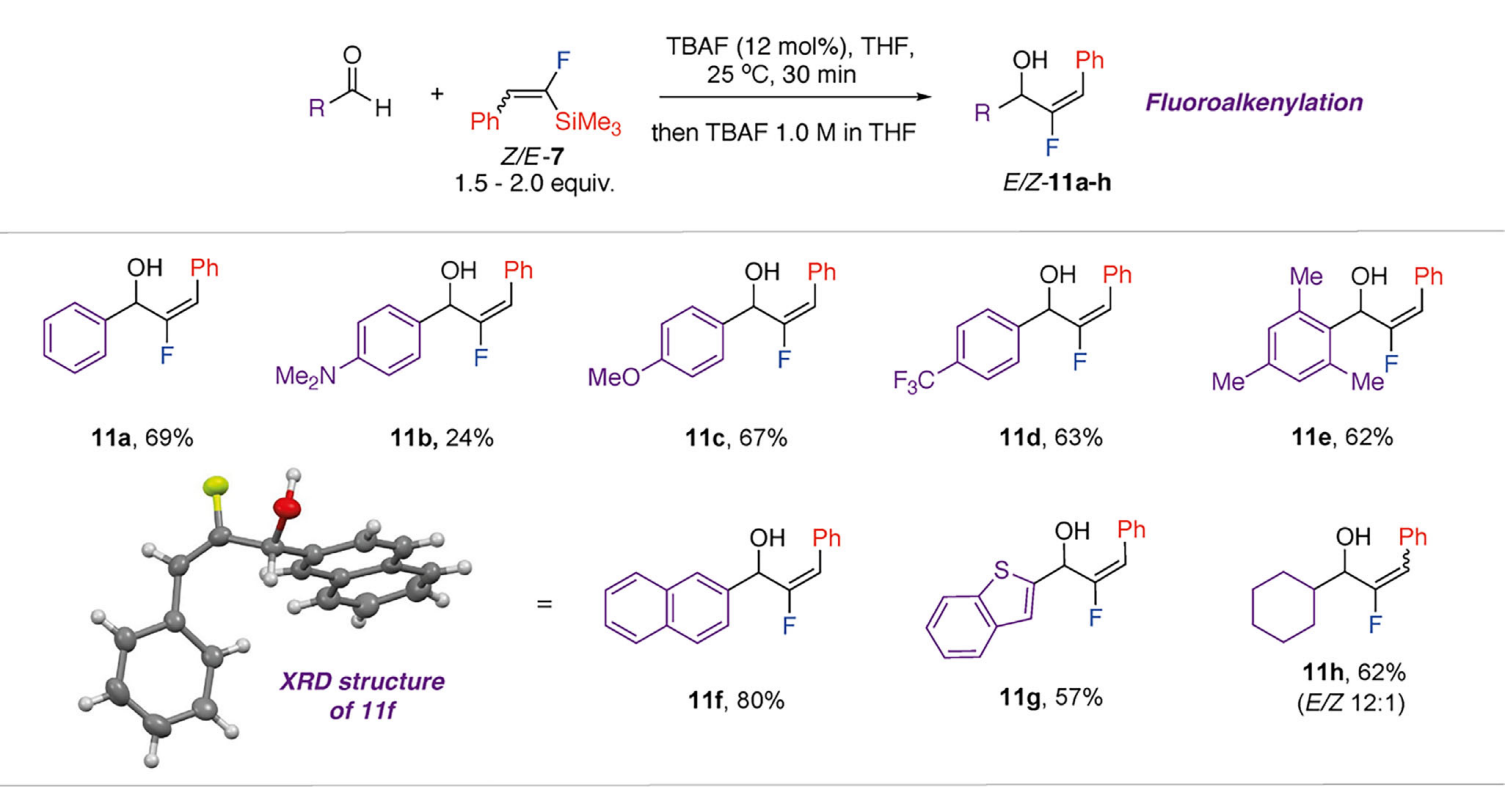
Reaction scope of fluoroalkenylation of aldehydes by *E/Z*‐**7**. Isolated yields given.

*E/Z*‐**7** was found to be an effective fluoroalkenylation reagent for simple electrophiles.^[^ ^43^, ^44^, ^45^, ^46^ ^]^ At 25 °C, using TBAF as a catalyst (12 mol%) in THF, the fluoroethenylation of aldehydes was observed using 1.5–2.0 equiv of **7**, allowing isolation of products **11a–h** (Figure 7). The reaction proceeded with good yields for both aliphatic and aromatic substrates, including those with electron‐donating and electron‐withdrawing groups, ortho‐substitution, as well as heteroaromatic substrates. For aromatic aldehydes the reaction produced solely the *E*‐isomer of **11a–g**. This is most likely due to the transformation being stereospecific and *Z*‐**7** reacting faster than *E*‐**7** under the conditions. Consistent with this argument, unreacted *E*‐**7** was observed by NMR spectroscopy prior to workup of crude reactions mixtures. Only in the case of the more reactive aliphatic aldehyde was a mixture of stereoisomeric products 12:1 *E/Z*‐**11h** observed.

The stereochemical control is notable. Alternative approaches to generate fluoroalkenes involving the hydrofluorination of alkyne functional groups, typically lead to a *trans*‐relationship between H and F atoms, rather than the c*is*‐relationship in **11a–h**.^[^ ^47^ ^]^ Broadly, fluoroalkene products can be accessed through chemoselective defluorination reactions of per or poly(fluorinated) alkenes through hydrodefluorination, nucleophilic vinylic substitution, or cross‐coupling reactions.^[^ ^48^, ^49^ ^]^ These protocols typically rely on transition metal catalysts, often based on palladium or copper, and require chemoselective reactions of the carbon–fluorine bonds to control the substitution pattern and degree of fluorination of the final product. More specifically, structural motifs related to **11a–h** can be accessed in a stereopure form, from cross‐coupling reactions of *gem*‐difluoro‐ (or *gem*‐dihalo‐) alkenes or the rearrangement of *gem*‐difluorocyclopropanes.^[^ ^50^, ^51^, ^52^, ^53^ ^]^

## Conclusions

In summary, a synthetic method has been developed for the defluoroalkylation of HCF_3_. Reaction with phosphine or silyl substituted organolithiums occurs with a 2:1 stoichiometry, extending the carbon chain and creating a new fluoroalkene functional group. Scale‐up was achieved using a continuous flow process. The products could be employed in a stereospecific fluoroethenylation of electrophiles, allowing direct access to tri‐substituted fluoroalkenes—useful bioisosteres for amide groups. Our study demonstrates an approach to repurpose HCF_3_ through creation of bench‐stable and storable reagents using flow methodology. While these reagents conserve the valuable carbon and fluorine content from HCF_3_, they do not contain CF_3_ groups and hence may contribute to sustainable uses of trifluoromethane.

## Supporting Information

The file contains synthetic procedures, flow setup, NMR spectra of all compounds, and computational methods (PDF); cartesian coordinates of the DFT‐optimised structures (XYZ); and crystal structures of CCDC 2345567 (for **1**), 2345568 (for **3·THF_1.5_**), 2476424 (for **9·TMEDA**), and 2402208 (for **11f**) crystallographic data (CIF).^[^ ^54^ ^]^ The authors have cited additional references within the Supporting Information.^[^ ^55^, ^56^, ^57^, ^58^, ^59^, ^60^, ^61^, ^62^, ^63^, ^64^, ^65^, ^66^, ^67^, ^68^, ^69^, ^70^, ^71^, ^72^, ^73^, ^74^, ^75^, ^76^, ^77^ ^]^

## Conflict of Interests

The authors declare no conflict of interest.

## Supporting information



Supporting Information

Supporting Information

## Data Availability

The data that support the findings of this study are available in the Supporting Information of this article.
