# Supplementary material for: Defluoroalkylation of Trifluoromethane with Organolithium Reagents: Mechanism and Synthesis of Fluoroalkenes
Source: Angew Chem Int Ed Engl. 2025 Nov 6;65(1):e16598. doi: 10.1002/anie.202516598 (PMC12759239; doi:10.1002/anie.202516598)
Supplement: Supplementary file 1 — Supporting Information [file ANIE-65-e16598-s002.pdf]

Defluoroalkylation of Trifluoromethane with Organolithium Reagents: Mechanism and Synthesis of Fluoroalkenes

Supplementary Information

Hodan R. Warsame, Sarah L. Patrick, James A. Bull, Philip W. Miller, Mark R. Crimmin*

m.crimmin@imperial.ac.uk

Department of Chemistry, Molecular Sciences Research Hub, 82 Wood Lane, Shepherds Bush, London, W12 0BZ, UK.

Contents

1. General Procedures: 4

2.1 Synthesis of Lithium Based Nucleophiles 5

3.1 General Procedure of Batch Scale Reactions of HCF_3 with Organolithium Reagents 1 and 3 16

3.2 Optimisation of Batch Scale Reactions of HCF_3 with Organolithium Reagents 1 and 3 16

4.1 Batch Synthesis of Fluorovinyl Silanes 5 and *Z/E*-7 19

5.1 General Procedure of Batch Scale Reactions of HCF_3 with Organolithium Reagent 2·TMEDA ... 22

5.2 Optimisation of Batch Scale Reactions of HCF_3 with Organolithium Reagent 2·TMEDA 23

5.3 Batch synthesis of (1-fluorovinyl)diphenylphosphine sulfide, 6 24

6.1 General Procedure of Batch Scale Reactions of HCF_3 with Organolithium Reagent 4·TMEDA ... 25

6.2 Optimisation of Batch Scale Reactions of HCF_3 with Organolithium Reagent 4·TMEDA 26

6.3 Synthesis of (*E*)-(1-fluoro-2-phenylvinyl)diphenylphosphine sulfide, 8 27

7.1 Batch Scale Proteodesilylation of Organolithium Reagent 9·TMEDA with HCF_3 and TBAF 28

7.2 General Procedure of Large-Scale Reactions of HCF_3 with 9·TMEDA 30

8.1 Reaction of HCF_3 with 3·TMEDA in Continuous Flow 34

9.1 Carbene Trapping Experiments 35

10.1 DOSY Studies of 1, 1·PMDETA, 2·TMEDA, 3·PMTEDA, 3·TMEDA and 3·$THF_{1.5}$, 4·TMEDA, and 9·TMEDA 37

11 Single Crystal X-ray Diffraction Data 44

11.1 Summary of Crystal Data 44

11.2 Refinement Details 46

11.3 Analysis of Crystal Structures 48

11.4 Crystal Structures 49

12.1 Fluoroalkenylation Reactions 51

13.1 IR Spectra 59

14.1 Mass Spectra 66

15.1 NMR Spectra 76

16.1 Computational Studies 128

16.2 NMR Simulations 129

16.3 1·PMDETA + HCF_3 DFT Study 131

16.4 1·PMDETA Functional Testing 134

16.5 NBO Analysis for Silyl Pathway 135

16.6 Energies for the Possible Pathways for Silyl Pathway 139

16.7 2·TMEDA + HCF_3 DFT Study 140

16.8 4·TMEDA + HCF_3 DFT Study 141

16.9 NBO Analysis for Phosphine Pathway 142

16.10 Energies for the Possible Pathways for Phosphine Pathway 144

16.11 Computational Coordinates 145

References 167

1. General Procedures:

Unless otherwise specified, standard Schlenk line and glovebox techniques were used for all manipulations under an inert atmosphere of nitrogen or argon. NMR scale reactions were performed in J. Young NMR tubes equipped with internal standard capillaries of ferrocene (^{1}H NMR spectroscopy), internal standard of α,α,α-trifluorotoluene was used (^{19}F NMR spectroscopy), and prepared in a glovebox. An MBraun Labmaster glovebox was used, operating at <0.1 ppm and H_2O and <0.1 ppm O_2.

^{1}H, ^{13}C ^{7}Li, and ^{19}F NMR spectra were recorded on BRUKER 400 MHz or 500 MHz machines and referenced against $Si(CH_3)_4$ (^{1}H and ^{13}C), $CFCl_3$ (^{19}F) or LiCl (^{7}Li). A 55 s delay was used for quantitative ^{19}F NMR integration. Data was processed using the MestReNova software package. The reported values for ^{1}H NMR data are as follows: chemical shifts (δ ppm), multiplicity (where s = singlet, d = doublet, dd = doublet of doublets, and m = multiplet), integration and coupling constant, J (Hz).

Solvents were dried over activated alumina from a solvent purification system (SPS) based upon the Grubbs design and degassed before use. Glassware was dried for >6 h prior to use at 120 °C. Benzene-d_6, toluene, diethyl ether, pentane, hexane, and THF were de-gassed and stored over 3 Å molecular sieves before use.

All reagents were acquired from Sigma Aldrich (Merck), Tokyo Chemical Industry, Scientific Laboratory Supplies, or Fluorochem and used without further purification unless specified. All aldehydes were distilled *via* vacuum distillation at 0.1 mbar prior to use. Trifluoromethane (HCF_3) was acquired from CK special gases and as a donation from Apollo Scientific and used without further purification or drying. Where liquids at 25 °C, reagents were dried over activated 3 Å molecular sieves and freeze-pump-thaw degassed prior to use. N,N,N′,N′-tetramethylethylenediamine (TMEDA) and N,N,N′,N″,N″-pentamethyldiethylenetriamine (PMDETA) were distilled over CaH_2 and dried over activated 3 Å molecular sieves and freeze-pump-thaw degassed prior to use.

Purifications were carried out by column chromatography on silica gel (tech grades, 60 Å, 230-400 mesh, 40-63 μm particle size).

AT-IR spectra were recorded on an Agilent Technologies Cary 630 FTIR spectrometer. The high-resolution mass spectrometry (HRMS) analyses were performed using electrospray ion source (ESI). ESI was performed using a Waters LCT Premier (ES-TOF) equipped with an ESI source operated in positive or negative ion mode.

2.1 Synthesis of Lithium Based Nucleophiles

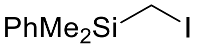

Synthesis of (iodomethyl)dimethylphenylsilane:

The synthesis is based on a literature procedure.[1]

NaI (3.55 g, 25.7 mmol, 1.9 equiv.) and (chloromethyl)dimethylphenylsilane (2.44 mL, 13.5 mmol, 1 equiv.) were dissolved in 50 mL of acetone and left to stir under reflux for 24 hours. Upon addition of (chloromethyl)dimethylphenylsilane, a colour change was observed from colourless to pale yellow. After 24 hours, a colourless precipitate was observed, and the reaction mixture was left to cool to room temperature. The reaction mixture was concentrated *in vacuo* by rotary evaporation resulting in a slurry. The product was then washed with 100 mL of *n*-hexane through a pad of Celite. The reaction was concentrated *in vacuo* by rotary evaporation resulting in a colourless liquid. Fractional distillation (95 °C, 1 atm) was carried out on the resulting solution and a pale-yellow solution was isolated (1.97 mL, 10.3 mmol, 76%).

NMR data are consistent with literature.[1]

^{1}H NMR (400 MHz, $CDCl_3$) δ/ppm: 7.54 (m, 2H, C**H**$_{Ph}$), 7.39 (m, 3H, C**H**$_{Ph}$), 2.19 (s, 2H, C**H**$_2$I), 0.45 (s, 6H, Si(C**H**$_3$)$_2$).

^{13}C NMR (101 MHz, $CDCl_3$) δ/ppm: 137.2 (s, 1C, **C**H$_{Ph}$), 134.1 (s, 1C, **C**H$_{Ph}$), 130.1 (s, 1C, **C**H$_{Ph}$), 128.4 (s, 1C, **C**H$_{Ph}$), -2.5 (s, 1C, **C**H$_2$I), -13.1 (s, 2C, Si(**C**H$_3$)$_2$).

^{29}Si NMR (80 MHz, $CDCl_3$) δ/ppm: -1.75 (s, $SiMe_2Ph$).

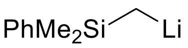

Synthesis of 1: (Iodomethyl)dimethylphenylsilane (0.693 mL, 3.62 mmol, 1 equiv.) was degassed *via* freeze-pump-thaw and dissolved in 20 mL of diethyl ether and transferred into a Schlenk *via* cannulation. At -78 °C, a 2.5 M solution of *n*BuLi in *n*-hexane (1.59 mL, 3.98 mmol, 1.1 equiv.) was added dropwise into the solution and left to thaw under a constant flow on N_2. After an hour, the high flow of N_2 evaporated off the diethyl ether leaving a white gel. The crude mixture was left under vacuum for an hour and then transferred into the glovebox. ^{1}H NMR analysis of the crude mixture showed that diethyl ether was coordinated to the organolithium. The crude product was re-dissolved in *n*-pentane (10 mL) and filtered. The mother liquor was left to crystallise at -35 °C for two days. The filtered crystals were dried *in vacuo* and isolated as a white crystalline solid (0.265 g, 1.69 mmol. 47%).

NMR data are consistent with literature.[2]

^{1}H NMR (500 MHz, C_6D_6) δ/ppm: 7.56 – 7.51 (m, 2H, C**H**$_{Ph}$), 7.28 – 7.21 (m, 2H, C**H**$_{Ph}$), 7.18 (m, 1H, C**H**$_{Ph}$), 0.25 (s, 6H, Si(C**H**$_3$)$_2$), -2.40 (s, 2H, C**H**$_2$Li).

^{13}C NMR (126 MHz, C_6D_6) δ/ppm: 142.9 (s, 1C, CH$_{Ph}$), 133.4 (s, 1C, **C**H$_{Ph}$), 129.8 (s, 1C, **C**H$_{Ph}$), 129.5 (s, 1C, **C**H$_{Ph}$), 2.3 (s, 1C, **C**H$_2$Li), -9.1 (s, 2C, Si(**C**H$_3$)$_2$).

^{7}Li NMR (194 MHz, C_6D_6) δ/ppm: 1.43.

^{29}Si NMR (99 MHz, C_6D_6) δ/ppm: -2.37 (s, $SiMe_2Ph$).

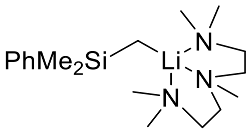

Synthesis of 1·PMDETA: In a N_2 filled glovebox, **1** (10 mg, 0.064 mmol. 1 equiv.) and PMDETA (13.4 µL, 0.064 mmol, 1 equiv.) were dissolved in 0.6 mL C_6D_6. The reaction mixture was monitored by ^{1}H NMR spectroscopy, and through integral comparison, it was found that one PMDETA ligand coordinates to **1**. DOSY studies also suggest that **1·PMDETA** is monomeric (*see Chapter 10.1*).

^{1}H NMR (400 MHz, C_6D_6) δ/ppm: 8.33 – 8.07 (m, 2H, C**H**$_{\mathbf{Ph}}$), 7.41 (m, 2H, C**H**$_{\mathbf{Ph}}$), 7.33 – 7.24 (m, 1H, C**H**$_{\mathbf{Ph}}$), 2.00 – 1.55 (m, 23H, **PMDETA**), 0.67 (s, 6H, Si(C**H**$_{\mathbf{3}}$)$_2$), -1.40 (s, 2H, C**H**$_{\mathbf{2}}$Li).

^{13}C NMR (101 MHz, C_6D_6) δ/ppm: 153.0 (s, 1C, CH$_{\mathbf{Ph}}$), 134.5 (s, 1C, **C**H$_{\mathbf{Ph}}$), 127.2 (s, 1C, **C**H$_{\mathbf{Ph}}$), 126.5 (s, 1C, **C**H$_{\mathbf{Ph}}$), 57.1 (s, 2C, N**C**H$_2$), 53.5 (s, 2C, N**C**H$_2$), 46.0 (s, 4C, N(**C**H$_3$)$_2$), 45.0 (s, 1C, N(**C**H$_3$)), 5.4 (s, 1C, **C**H$_2$Li), -8.3 (s, 2C, Si(**C**H$_3$)$_2$).

^{7}Li NMR (156 MHz, C_6D_6) δ/ppm: 2.23.

^{29}Si NMR (80 MHz, C_6D_6) δ/ppm: -5.06.

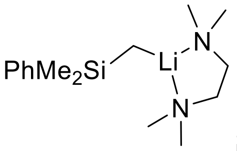

Synthesis of 1·TMEDA: In a N_2 filled glovebox, **1** (10 mg, 0.064 mmol. 1 equiv.) and TMEDA (9.60 µL, 0.064 mmol, 1 equiv.) were dissolved in 0.6 mL C_6D_6. The reaction mixture was monitored by ^{1}H NMR spectroscopy, and through integral comparison, it was found that one TMEDA ligand coordinates to **1**.

^{1}H NMR (400 MHz, C_6D_6) δ/ppm: 8.11 – 7.85 (m, 2H, C**H**$_{\textbf{Ph}}$), 7.35 (m, 2H, C**H**$_{\textbf{Ph}}$), 7.26 – 7.21 (m, 1H, C**H**$_{\textbf{Ph}}$), 1.76 (m, 16H, **TMEDA**), 0.61 (s, 6H, Si(C**H**$_{\textbf{3}}$)$_{\textbf{2}}$), -1.47 (s, 2H, C**H**$_{\textbf{2}}$Li).

^{13}C NMR (101 MHz, C_6D_6) δ/ppm: 150.2 (s, 1C, CH$_{\textbf{Ph}}$), 133.8 (s, 1C, **C**H$_{\textbf{Ph}}$), 127.5 (s, 1C, **C**H$_{\textbf{Ph}}$), 127.1 (s, 1C, **C**H$_{\textbf{Ph}}$), 56.4 (s, 2C, N(**C**H$_2$)), 45.6 (s, 4C, N(**C**H$_3$)$_2$), 4.5 (s, 1C, **C**H$_2$Li), -8.9 (s, 2C, Si(**C**H$_3$)$_2$).

^{7}Li NMR (156 MHz, C_6D_6) δ/ppm: 2.61.

^{29}Si NMR (80 MHz, C_6D_6) δ/ppm: -4.77.

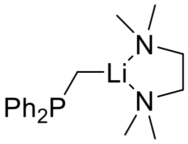

Synthesis of 2·TMEDA:

In a N_2 filled glovebox, methyldiphenylphosphane (0.93 mL, 5.00 mmol, 1 equiv.) and TMEDA, (0.75 mL, 5.00 mmol, 1 equiv.) were dissolved in 15 mL of THF and transferred into a Schlenk. At – 78 °C, a 2.5 M solution of *n*BuLi in *n*-hexane (2.20 mL, 5.50 mmol, 1.1 equiv.) was added dropwise to the reaction mixture and a colour change from colourless to red was observed. The mixture was left to react at room temperature for 16 hours and a further colour change to brown was observed. The solvent, THF, was removed in vacuo and the oil residue was redissolved in *n*-pentane (15 mL) and a pale-yellow precipitate formed. The solid was filtered *via* cannula filtration and the remaining pale-yellow precipitate was dried *in vacuo*. The compound was isolated as a pale yellow solid (0.79 g, 2.44 mmol, 49%).

^{1}H NMR (400 MHz, C_6D_6) δ/ppm: 7.93 (t, *J* = 7.0 Hz, 4H, C**H$_{Ph}$**), 7.27 (t, *J* = 7.4 Hz, 4H, C**H$_{Ph}$**), 7.11 (t, *J* = 7.3 Hz, 2H, C**H$_{Ph}$**), 1.84 (s, 12H, NC**H$_3$**, **TMEDA**), 1.69 (s, 4H, C**H$_2$**, **TMEDA**), 0.21 (s, 2H, C**H$_2$**Li).

^{13}C NMR (101 MHz, C_6D_6) δ/ppm: 152.1 (d, *J* = 7.7 Hz, 1C, **C**H$_{Ph}$), 132.5 (d, J = 14.6 Hz, 1C, **C**H$_{Ph}$), 127.4 (d, J = 14.6 Hz 1C, **C**H$_{Ph}$), 125.8 (s, 1C, **C**H$_{Ph}$), 56.7 (s, 2C, **C**H$_2$), 46.0 (s, 4C, N**C**H$_3$), 4.0 (d, *J* = 35.7 Hz, 1C, **C**H$_2$Li).

^{31}P{^{1}H} NMR (162 MHz, C_6D_6) δ/ppm: 1.28.

^{7}Li NMR (156 MHz, C_6D_6) δ/ppm: 2.28.

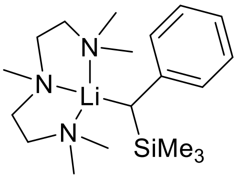

Synthesis of 3·PMDETA: The synthesis is based on a literature procedure.[3]

In a N_2 filled glovebox, benzyltrimethylsilane (1.16 mL, 6.09 mmol, 1 equiv.) and PMDETA (1.27 mL, 6.09 mmol, 1 equiv.) were dissolved in 20 mL of *n*-pentane, and added into a Schlenk. At -78 °C, a 2.5 M solution of nBuLi in *n*-hexane (2.68 mL, 6.69, 1.1 equiv.) was added dropwise to the Schlenk. The reaction mixture was thawed to room temperature, and a colour change from colourless to pale yellow was observed. The reaction mixture was left stirring for 12 hours at room temperature, and a yellow precipitate formed. The solution was filtered *via* cannula filtration and the remaining yellow precipitate was dried *in vacuo*. The compound was isolated as a bright yellow solid (0.764 g, 2.22 mmol, 38%).

^{1}H NMR (400 MHz, C_6D_6) δ/ppm: 7.11 (m, 2H, C**H$_{Ph}$**), 6.81 (s, 2H, C**H$_{Ph}$**), 6.46 (t, *J* = 7.0, 1H C**H$_{Ph}$**), 2.02 – 1.35 (m, 23H, **PMDETA**), 1.89 (s, 1H, C**H**Li), 0.50 (s, 9H, Si**(CH_3)$_3$**).

^{13}C NMR (101 MHz, C_6D_6) δ/ppm: 159.5 (s, 1C, CH$_{Ph}$), 129.1 (s, 1C, **C**H$_{Ph}$), 120.7 (s, 1C, **C**H$_{Ph}$), 109.4 (s, 1C, **C**H$_{Ph}$), 57.7 (s, 2C, N**C**H_2), 54.0 (s, 2C, N**C**H_2), 46.0 (overlapping, 11C, N(**C**H_3)), 45.9 (s, 1C, **C**HLi), 3.5 (s, 3C, Si**(CH_3)$_3$**).

^{7}Li NMR (156 MHz, C_6D_6) δ/ppm: 0.50.

^{29}Si NMR (99 MHz, C_6D_6) δ/ppm: -14.05.

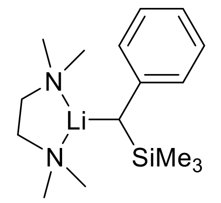

Synthesis of 3·TMEDA: The synthesis is based on a literature procedure.[3]

In a N_2 filled glovebox, benzyltrimethylsilane (1.16 mL, 6.09 mmol, 1 equiv.) and TMEDA (0.912 mL, 6.09 mmol, 1 equiv.) was dissolved in 20 mL of *n*-pentane and added into a Schlenk. At -78 °C, a 2.5 M solution of *n*BuLi in *n*-hexane (2.68 mL, 6.69 mmol, 1.1 equiv.) was added dropwise to the Schlenk. The reaction mixture was thawed, and a colour change from colourless to pale yellow. The reaction mixture was left stirring for 12 hours at room temperature, and a yellow precipitate formed. The solution was filtered *via* cannula filtration and the remaining yellow precipitate was dried *in vacuo*. The compound was isolated as a bright yellow solid (1.01 g, 3.53 mmol, 58%).

^{1}H NMR (500 MHz, C_6D_6) δ/ppm: 7.03 (m, 2H, C**H_{Ph}**), 6.74 (s, 2H, C**H_{Ph}**), 6.33 (m, 1H, C**H_{Ph}**), 1.99 (s, 1H, C**H**Li), 1.65 (s, 16H, **TMEDA**), 0.46 (s, 9H, Si**(CH_3)$_3$**).

^{13}C NMR (126 MHz, C_6D_6) δ/ppm: 156.9 (s, 1C, **C**H_{Ph}), 130.5, (s, 1C, **C**H_{Ph}), 119.0 (s, 1C, **C**H_{Ph}), 109.7, (s, 1C, **C**H_{Ph}), 56.1 (s, 2C, N(**C**H_2)), 45.4 (s, 4C, N(**C**H_3)$_2$), 42.1 (s, 1C, **C**HLi), 2.97 (s, 3C, Si**(CH_3)$_3$**).

^{7}Li NMR (194 MHz, C_6D_6) δ/ppm: 1.57.

^{29}Si NMR (99 MHz, C_6D_6) δ/ppm: -15.14.

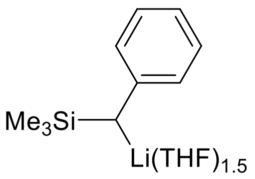

Synthesis of 3·THF$_{1.5}$: In a N_2 filled glovebox, benzyltrimethylsilane (2.32 mL, 12.18 mmol, 1 equiv.) and THF (2.99 mL, 36.54 mmol, 3 equiv.) was dissolved in 20 mL of pentane and added into a Schlenk. At -78 °C, a 1.6M solution of *n*BuLi in *n*-hexane (8.38 mL, 13.40 mmol, 1.1 equiv.) was added dropwise into the Schlenk. The reaction mixture was thawed to room temperature and colour change was observed from colourless to pale yellow. The solution was concentrated *in vacuo* and the crude product redissolved in *n*-pentane. The solution was further concentrated *in vacuo* and a bright yellow solid crashed out. The solid was washed in *n*-pentane and filtered twice. The resulting yellow precipitate was dried *in vacuo* and isolated as a bright yellow crystalline solid (0.578 g, 2.80 mmol, 23%).

^{1}H NMR (500 MHz, C_6D_6) δ/ppm: 7.03 – 6.94 (m, 2H, C**H$_{Ph}$**), 6.83 (d, *J* = 7.8 Hz, 2H, C**H$_{Ph}$**), 6.23 (t, *J* = 7.7 Hz, 1H, C**H$_{Ph}$**), 3.26 – 3.17 (m, 6H, THF), 2.10 (s, 1H, C**H**Li), 1.29 – 1.18 (m, 6H, THF), 0.47 (s, 9H, Si**(CH$_3$)$_3$**).

^{13}C NMR (126 MHz, C_6D_6) δ/ppm: 130.2, (s, 1C, **C**H$_{Ph}$), 128.2, (s, 1C, **C**H$_{Ph}$), 118.6, (s, 1C, **C**H$_{Ph}$), 109.9, (s, 1C, **C**H$_{Ph}$), 68.2, (**C**H$_2$O), 25.3 (**C**H$_2$), 39.4 (s, 1C, **C**HLi), 2.5 (s, 3C, Si**(CH$_3$)$_3$**).

^{7}Li NMR (194 MHz, C_6D_6) δ/ppm: 0.10.

^{29}Si NMR (99 MHz, C_6D_6) δ/ppm: -14.87.

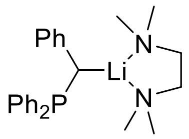

Synthesis of 4·TMEDA

In a N_2 filled glovebox, benzyldiphenylphosphane (1.00 g, 3.64 mmol, 1 equiv.) and TMEDA, (0.55 mL, 3.64 mmol, 1 equiv.) were dissolved in 15 mL of toluene and transferred into a Schlenk. At –78 °C, a 2.5 M solution of *n*BuLi in *n*-hexane (1.60 mL, 4.00 mmol, 1.1 equiv.) was added dropwise to the reaction mixture and a colour change from colourless to orange was observed. The reaction mixture was heated to 100 °C for two hours and a further colour change to red was observed. The solvent, toluene, was removed *in vacuo* and the oil residue was redissolved in *n*-pentane (15 mL) and an orange precipitate formed. The solid was filtered *via* cannula filtration and the remaining orange precipitate was dried *in vacuo*. The compound was isolated as an orange solid (1.07 g, 2.69 mmol, 74%).

^{1}H NMR (400 MHz, C_6D_6) δ/ppm: 7.84 (ddd, *J* = 8.0, 6.6, 1.4 Hz, 4H, C$\mathbf{H_{Ph}}$), 7.19 – 7.13 (m, 6H, C$\mathbf{H_{Ph}}$), 7.06 (dt, *J* = 9.2, 7.4 Hz, 4H, C$\mathbf{H_{Ph}}$), 6.43 (t, *J* = 7.1 Hz, 1H, C$\mathbf{H_{Ph}}$), 3.19 (d, *J* = 7.7 Hz, 1H, C**H**Li), 1.52 (s, 12H, NC$\mathbf{H_3}$), 1.35 (s, 4H, C$\mathbf{H_2}$).

^{13}C NMR (126 MHz, C_6D_6) δ/ppm: 154.4 (d, *J* = 23.4 Hz, $\mathbf{C}H_{Ph}$), 148.6 (d, *J* = 13.6 Hz, $\mathbf{C}H_{Ph}$), 132.6 (s, $\mathbf{C}H_{Ph}$), 132.5 (s, $\mathbf{C}H_{Ph}$), 130.2 (s, $\mathbf{C}H_{Ph}$), 128.4 (s, $\mathbf{C}H_{Ph}$), 118.1 (s, $\mathbf{C}H_{Ph}$), 112.1 (d, *J* = 2.6 Hz, $\mathbf{C}H_{Ph}$), 56.1 (s, $\mathbf{C}H_2$), 44.7 (s, N$\mathbf{C}H_3$), 44.4 (d, *J* = 8.7 Hz, **C**HPLi).

^{31}P NMR (202 MHz, C_6D_6) δ/ppm: -19.0 (q, *J* = 7.4 Hz).

^{31}P{^{1}H} NMR (162 MHz, C_6D_6) δ/ppm: -19.0.

^{7}Li NMR (156 MHz, C_6D_6) δ/ppm: 1.02.

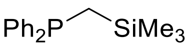

Synthesis of diphenyl((trimethylsilyl)methyl)phosphane:

Synthesis is based on a literature procedure.[4]

In a N_2 filled glovebox, chlorodiphenylphosphine (2.51 mL, 13.6 mmol, 1 equiv.) was dissolved in 1 mL of Et_2O and transferred into an ampoule. At 0 °C, a 1.0 M solution of (trimethylsilyl)methyl) magnesium bromide in Et_2O (29 mL, 29 mmol, 2.1 equiv.) was added dropwise and the reaction mixture left to stir at room temperature for two hours. At 0 °C, the reaction was quenched with 20 mL saturated degassed NH_4Cl and left to stir for 20 minutes at room temperature. The organic layer was then washed with 20 mL of degassed water and dried with $MgSO_4$. The solution was concentrated *in vacuo*, resulting in isolation of the product as a colourless oil (2.15 g, 7.91 mmol, 58%).

^{1}H NMR (400 MHz, C_6D_6) δ/ppm: 7.51 – 7.41 (m, 4H, C$\mathbf{H_{Ph}}$), 7.11 – 7.02 (m, 6H, C$\mathbf{H_{Ph}}$), 1.22 (s, 2H, C$\mathbf{H_2}$), -0.04 (s, 9H, Si$\mathbf{(CH_3)_3}$).

^{13}C NMR (101 MHz, C_6D_6) δ/ppm: 142.4 (d, *J* = 15.5 Hz, CH_{Ph}), 133.2 (d, *J* = 19.7 Hz, $\mathbf{C}H_{Ph}$), 128.8 (d, *J* = 2.3 Hz, $\mathbf{C}H_{Ph}$), 128.8 (d, *J* = 2.7 Hz, CH_{Ph}), 15.2 (d, *J* = 30.3 Hz, $\mathbf{C}H_2$), 0.18 (d, *J* = 4.9 Hz, Si$\mathbf{(CH_3)_3}$).

^{31}P NMR (162 MHz, C_6D_6) δ/ppm: -22.2 (t, *J* = 6.2 Hz).

^{31}P{^{1}H} NMR (162 MHz, C_6D_6) δ/ppm: -22.2 (s).

^{29}Si NMR (C_6D_6) δ/ppm: 0.65 (s). (from **^{29}Si–^{1}H HMBC**)

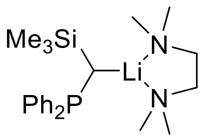

Synthesis of 9·TMEDA

In a N_2 filled glovebox, diphenyl((trimethylsilyl)methyl)phosphane (1.20 mL, 4.43 mmol, 1 equiv.) and TMEDA, (0.66 mL, 4.43 mmol, 1 equiv.) were dissolved in 15 mL of *n*-pentane and transferred into a Schlenk. At –78 °C, a 2.5 M solution of *n*BuLi in *n*-hexane (1.95 mL, 4.87 mmol, 1.1 equiv.) was added dropwise to the reaction mixture and a colour change from colourless to bright yellow was observed. The reaction mixture was left to react at room temperature for two hours and yellow solid precipitated. The solid was filtered *via* cannula filtration and the remaining yellow precipitate was dried *in vacuo*. The compound was isolated as a yellow solid (0.86 g, 2.18 mmol, 49%).

^{1}H NMR (400 MHz, C_6D_6) δ/ppm: 7.82 (ddt, *J* = 7.6, 4.5, 1.5 Hz, 4H, C**H$_{Ph}$**), 7.18 (t, *J* = 0.9 Hz, 3H, C**H$_{Ph}$**), 7.14 – 7.13 (m, 1H, C**H$_{Ph}$**), 7.09 – 6.98 (m, 2H, C**H$_{Ph}$**), 1.60 (s, 12H, NC**H$_3$**), 1.39 (s, 4H, NC**H$_2$**), 0.45 (d, *J* = 1.0 Hz, 9H, Si**(CH$_3$)$_3$**), 0.31 (d, *J* = 5.4 Hz, 1H, C**H**Li).

^{13}C NMR (101 MHz, C_6D_6) δ/ppm: 153.5 (d, *J* = 24.0 Hz, **C**H$_{Ph}$), 132.1 (d, *J* = 18.2 Hz, **C**H$_{Ph}$), 128.2 (s, **C**H$_{Ph}$) 126.3 (s, **C**H$_{Ph}$), 56.4 (s, N**C**H$_2$), 45.4 (s, N**C**H$_3$), 8.7 (d, *J* = 50.3 Hz, **C**HLi), 5.3 (d, *J* = 8.3 Hz, Si**(CH$_3$)$_3$**).

^{31}P NMR (162 MHz, C_6D_6) δ/ppm: -4.54 (s).

^{29}Si NMR (80 MHz, C_6D_6) δ/ppm: -6.04 (s). (from **^{29}Si–^{1}H HMBC**)

3.1 General Procedure of Batch Scale Reactions of HCF_3 with Organolithium Reagents 1 and 3

In a N_2 filled glovebox, 0.6 mL of a 0.1 M solution of **1** and **3** in C_6D_6 or THF was added to a J. Young NMR tube equipped with a standard (ferrocene capillary or known amount of α,α,α-trifluorotoluene), and a t=0 ^1H NMR spectrum was recorded. The solution was degassed once *via* freeze-pump-thaw and HCF_3 (1 bar, 25 °C, 0.088 mmol, 16 equiv.) was added to the J. Young NMR tube. The J. Young NMR tube was inverted several times, and a t=1 ^1H and ^{19}F NMR spectra was recorded. The yield was determined *in situ* upon integral comparison to the ferrocene internal standard in the ^1H NMR spectrum or through *in situ* upon integral comparison to a known amount of α,α,α-trifluorotoluene in the ^{19}F NMR spectrum.

3.2 Optimisation of Batch Scale Reactions of HCF_3 with Organolithium Reagents 1 and 3

- **Ligand Screen**

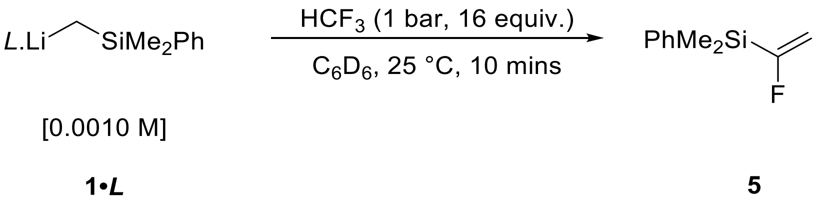

Nucleophile	Yield (%)[a]
**1·PMDETA**	69
**1·TMEDA**	44

Table S1: Results of ligand variation. [a]Yield calculated by ^1H NMR spectroscopy using ferrocene as standard.

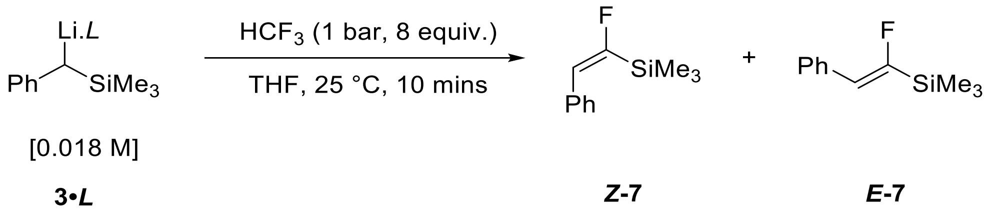

Nucleophile	Yield (%)[b]	Ratio of Isomers (*Z*:*E*)
**2·THF$_{1.5}$**	59	4.4:1
**2·PMDETA**	83	3.9:1
**2·TMEDA**	72	3.0:1

Table S2: Results of ligand variation. [b]Yield calculated by ^{19}F NMR spectroscopy using α,α,α-trifluorotoluene as an internal standard.

- **Concentration screen**

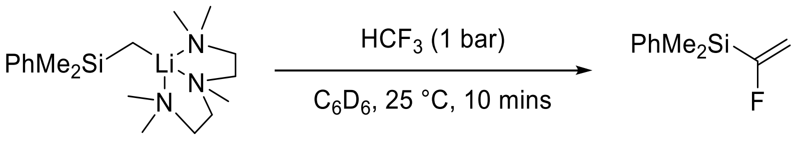

1•PMDETA **5**

Concentration of **1·PMDETA** (M) {amount of **1·PMDETA** (mmol)}	Approx. equivalent of HCF_3 (headspace of NMR tube = 2.2 mL)	Yield of **3** (%)[a]
0.073 {0.044}	2	25
0.037 {0.022}	4	35
0.018 {0.011}	8	42
0.0010 {0.0055}	16	69

Table S3: Effect of HCF_3 equivalence/concentration of **1·PMDETA**. [a]Yield calculated by ^{1}H NMR spectroscopy using ferrocene as standard.

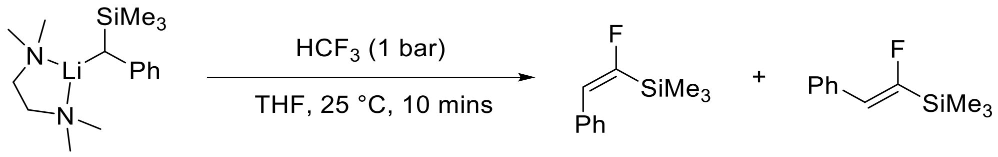

3•TMEDA ***Z*-7** ***E*-7**

Concentration of **3·TMEDA** (M) {amount of **3·TMEDA** (mmol)}	Approx. equivalence of HCF_3 (headspace of NMR tube = 2.2 mL)	Yield of ***Z/E*-7** (%)[b]	Ratio of Isomers (*Z*:*E*)
0.183 {0.110}	0.80	43, 10	4.3:1
0.081 {0.048}	1.8	31, 8	3.9:1
0.037 {0.022}	4	34, 6	5.7:1
0.018 {0.011}	8	54, 18	3.0:1
0.0010 {0.0055}	16	50, 32	1.6:1

Table S4: Effect of HCF_3 equivalence/concentration of **3·TMEDA**. [b]Yield calculated by ^{19}F NMR spectroscopy using α,α,α-trifluorotoluene as an internal standard.

- **Solvent screen**

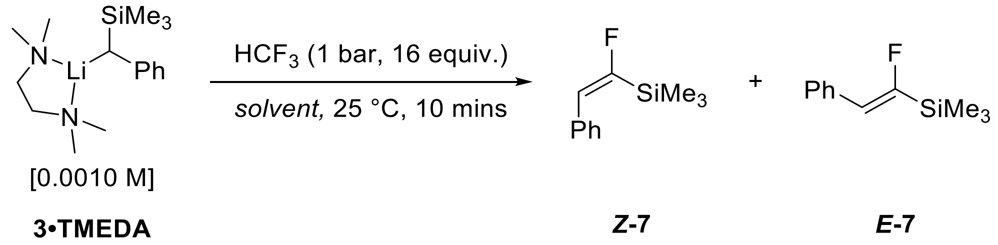

Solvent	Yield (%)[a,b]
C_6D_6	41[b]
C_6H_6	40, 28[a]
Toluene	21, <11[a]
THF	50, 32[a]

Table S5: Results of solvent screen. [a]Yield calculated by ^{1}H NMR spectroscopy using ferrocene as standard. [b]Yield calculated by ^{19}F NMR spectroscopy using α,α,α-trifluorotoluene as an internal standard.4.1 Batch synthesis of fluorovinyl silanes 5 and *Z/E*-7

4.1 Batch Synthesis of Fluorovinyl Silanes 5 and *Z/E*-7

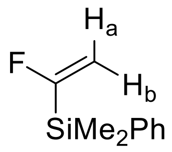

Synthesis of (1-fluorovinyl)dimethyl(phenyl)silane, 5:

NMR Scale Procedure: In a N_2 filled glovebox, 55 µL of a 0.1 M solution of **1·PMDETA** (0.0055 mmol) was dissolved in 0.545 mL of C_6D_6 was added to a J. Young NMR tube equipped with a ferrocene capillary internal standard, and a t=0 ^1H NMR spectrum was recorded. The solution was degassed once *via* freeze-pump-thaw and HCF_3 (1 bar, 25 °C, 0.088 mmol, 16 equiv.) was added to the J. Young NMR tube. The J. Young NMR tube was inverted multiple times, and a t=1 ^1H and ^{19}F NMR spectra was recorded. Upon addition of HCF_3, a colour change from pale cream to pale brown was observed. The yield was determined *in situ* upon integral comparison [δ/ppm: -1.50 (s, 1H, C$\underline{H}_2$)] to the ferrocene internal standard in the ^1H NMR spectrum (41%).

Preparative Procedure: In a N_2 filled glovebox, **1** (0.122 g, 0.781 mmol, 1 equiv.) and **PMDETA** (163 µL, 0.781 mmol, 1 equiv.) was dissolved in 20 mL of toluene and transferred to an ampoule. The solution was degassed once *via* freeze-pump-thaw and HCF_3 (1 bar, 25 °C, 6.26 mmol, 8 equiv.) was added to the ampoule. The reaction was left to stir for 30 minutes at room temperature and a colour change from pale yellow to pale brown was observed. The reaction was filtered to remove LiF and PMDETA and concentrated *in vacuo* by rotary evaporation before being purified by silica column chromatography, eluted with 100% *n*-pentane. A colourless oil was isolated (20.8%, *in-situ* ^{19}F NMR yield).

NMR data are consistent with literature.[5]

^1H NMR (400 MHz, CDCl$_3$, 298 K) δ/ppm: 7.61 – 7.55 (m, 2H, C**H$_{Ph}$**), 7.43 – 7.35 (m, 3H, C**H$_{Ph}$**), 5.34 (dd, $^3J_{HaF}$ = 33.1, $^2J_{HaHb}$ = 3.0 Hz, 1H, **H$_a$**C=CF), 4.79 (dd, $^3J_{HbF}$ = 61.8, $^2J_{HaHb}$ = 2.9 Hz, 1H, **H$_b$**C=CF), 0.46 (s, 6H, Si(C**H$_3$**)$_2$Ph).

^{13}C NMR (126 MHz, CDCl$_3$) δ/ppm: 174.4 (d, $^1J_{CF}$ = 282.0 Hz, CH=**C**F), 135.1 (s, 1C, **C**H$_{Ph}$), 133.9 (s, 1C, **C**H$_{Ph}$), 129.8 (s, 1C, **C**H$_{Ph}$), 128.0 (s, 1C, **C**H$_{Ph}$), 107.1 (d, 1C, $^2J_{CF}$ = 7.9 Hz, **C**H=CF), -4.1 (s, 2C, Si(**C**H$_3$)$_2$Ph)

^{19}F NMR (377 MHz, CDCl$_3$, 298K) δ/ppm: -104.0 (dd, $^3J_{FHb}$ = 61.9, $^3J_{FHa}$ = 33.1 Hz, **F**C=CH$_a$H$_b$).

$^{19}F\{^1H\}$ NMR (377 MHz, CDCl$_3$, 298K) δ/ppm: -104.0 (s).

Synthesis of *Z/E*-1-fluoro-2-phenyl-1-trimethylsilylethene, *Z/E*-7:

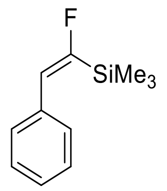

NMR Scale Procedure: In a N_2 filled glovebox, 0.6 mL of a 0.1 M solution of **3·TMEDA** (0.011 mmol, 1 equiv.) in THF was added to a J. Young NMR tube. The solution was degassed once *via* freeze-pump-thaw and HCF_3 (1 bar, 25 °C, 0.088 mmol, 8 equiv.) was added to the J. Young NMR tube. The J. Young NMR tube was inverted multiple times. Upon addition of HCF_3, a colour change from yellow to pale brown *via* a black intermediate was observed. The yield of the isomers was determined in situ upon integral comparison to a known amount of α,α,α-trifluorotoluene in the ^{19}F NMR spectrum (72%) (3:1 *Z*:*E*).

Preparative Procedure: In a N_2 filled glovebox, **3·TMEDA** (1.16 g, 4.05 mmol, 1 equiv.) was dissolved in 100 mL of THF making the concentration of the solution 0.0807 M and transferred into a Strauss flask (Headspace = 400 mL). The solution was degassed *via* freeze-pump-thaw and HCF_3 (1 bar, 25 °C, 16.1 mmol, 2 equiv.) was added to the Strauss flask. The reaction mixture was left to stir for 30 minutes at room temperature and a colour change was observed from green to brown *via* a black intermediate. The reaction was filtered to remove LiF and TMEDA and concentrated *in vacuo* by rotary evaporation before being purified by silica column chromatography, eluted with 100% *n*-pentane. A colourless oil was isolated (168 mg, 0.863 mmol, 36 %, 3.8:1 (*Z*:*E*)).

NMR data are consistent with literature.[6]

***Z*-1-fluoro-2-phenyl-1-trimethylsilylethene:**

^{1}H NMR (400 MHz, $CDCl_3$) δ/ppm: 7.65 – 7.22 (m, 10H, C**H$_{Ph}$**), 7.10 (d, 1H, $^3J_{HF}$ = 35.1 Hz, *Z*-**H**C=CF), 0.14 (s, 9H, Si**(CH$_3$)$_3$**).

^{13}C NMR (126 MHz, $CDCl_3$) δ/ppm: 170.0 (d, 1C, $^1J_{CF}$ = 275.0 Hz, CH=**C**F), 129.1 (s, 1C, **C**H$_{Ph}$), 129.1 (s, 1C, **C**H$_{Ph}$), 128.1 (s, 1C, **C**H$_{Ph}$), 127.4 (s, 1C, **C**H$_{Ph}$), 125.3 (d, J = 18.0 Hz, **C**H), -1.6 (s, 3C, Si**(CH$_3$)$_3$**).

^{19}F NMR (377 MHz, CDCl3) δ/ppm: -106.1 (d, $^3J_{FH}$ = 35.1 Hz, *Z*-HC=C**F**).

^{19}F{^{1}H} NMR (377 MHz, $CDCl_3$) δ/ppm: -106.1 (s).

IR (thin film)/cm^{-1}: 3088, 3034, 2954, 1813, 1476, 1251, 1034, 842, 669.

MS (TOF MS ES+) m/z: calculated for $[C_{11}H_{11}FSi]^+$: 194.0922; Found 194.0922 / 194.0926

***E*-1-fluoro-2-phenyl-1-trimethylsilylethene:**

^{1}H NMR (400 MHz, $CDCl_3$) δ/ppm: 7.43 – 7.22 (m, 10H, C**H_{Ph}**), 5.91 (d, $^{3}J_{HF}$ = 52.1 Hz, 1H, *E*-**H**C=CF), 0.31 (s, 9H, Si**$(CH_3)_3$**).

^{13}C NMR (126 MHz, $CDCl_3$) δ/ppm: 133.7 (d, 1C, $^{2}J_{CF}$ = 17.6 Hz, **C**H=CF), 129.0 (d, 1C, $^{1}J_{CF}$ = 201.5 Hz, CH=**C**F), 128.4 (s, 1C, **C**H_{Ph}), 125.4 (s, 1C, **C**H_{Ph}), 125.2 (s, 1C, **C**H_{Ph}), 119.7 (s, 1C, **C**H_{Ph}), -1.6 (s, 3C, Si**$(CH_3)_3$**).

^{19}F NMR (377 MHz, $CDCl_3$) δ/ppm: -113.5 (d, $^{3}J_{FH}$ = 52.1 Hz, *E*-HC=C**F**).

^{19}F{^{1}H} NMR (377 MHz, $CDCl_3$) δ/ppm: -113.5 (s).

IR (thin film)/cm^{-1}: 3088, 3034, 2954, 1813, 1476, 1251, 1034, 842, 669.

MS (TOF MS ES+) m/z: calculated for $[C_{11}H_{11}FSi]^{+}$: 194.0922; Found 194.0922 / 194.0926

5.1 General Procedure of Batch Scale Reactions of HCF_3 with Organolithium Reagent 2·TMEDA

In a N_2 filled glovebox, **2·TMEDA** (7.7 mg, 0.024 mmol, 1 equiv.) was dissolved in 0.6 mL of C_6D_6/THF and was added to a J. Young NMR tube equipped with a standard (known amount of hexafluorobenzene). The solution was degassed once via freeze-pump-thaw and HCF_3 (1 bar, 25 °C, 0.088 mmol, 3.7 equiv.) was added to the J. Young NMR tube. The J. Young NMR tube was inverted several times, and a ^{19}F NMR spectra was recorded. The yield was determined by ^{19}F NMR spectroscopy by comparison to a known amount of hexafluorobenzene added once the reaction was complete.

5.2 Optimisation of Batch Scale Reactions of HCF_3 with Organolithium Reagent 2·TMEDA

- **Concentration Screen**

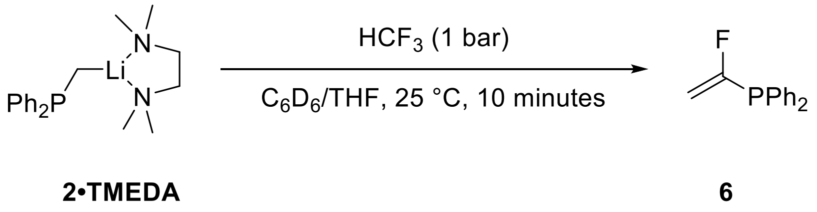

Concentration of 2·TMEDA (M) {amount of 2·TMEDA (mmol)}	Approx. equivalent of HCF_3 (headspace of NMR tube = 2.2 mL)	Yield of 6 (%)[c]
0.20 {0.110}	0.8	40
0.13 {0.080}	1.1	47
0.073 {0.057}	1.5	60
0.037 {0.024}	3.7	52

Table S6: Effect of HCF_3 equivalence/concentration on **2·TMEDA**. [c] Yield calculated by ^{19}F NMR spectroscopy using hexafluorobenzene as an internal standard.

- **Solvent Screen**

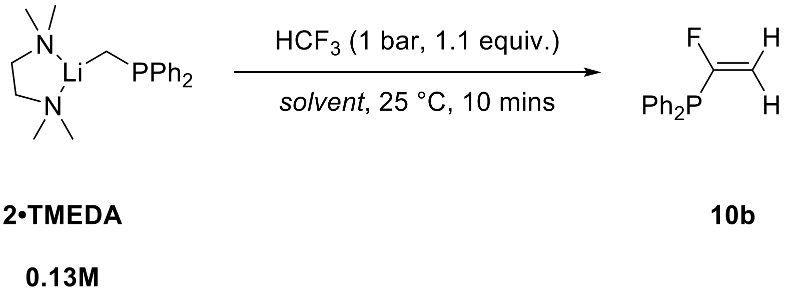

Solvent	Yield of 10b (%)[c]
C_6D_6/THF (1:1)	47
THF	45
C_6D_6	35
Toluene	32

Table S7: Results of solvent screen. [c] Yield calculated by ^{19}F NMR spectroscopy using hexafluorobenzene as an internal standard.

5.3 Batch synthesis of (1-fluorovinyl)diphenylphosphine sulfide, 6

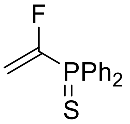

Preparative Procedure: In a N_2 filled glovebox, **2·TMEDA** (0.174 g, 0.540 mmol, 1 equiv.) was dissolved in 5 mL of THF making the concentration of the solution 0.11 M and transferred into an ampoule (Headspace = 15 mL). The solution was degassed *via* freeze-pump-thaw and HCF_3 (1 bar, 25 °C, 0.594 mmol, 1.1 equiv.) was added to the ampoule. The reaction was left to stir for 30 minutes at room temperature and a colour change from pale yellow to orange was observed. Sulfur, S_8, (0.317 g, 1.24 mmol, 2.3 equiv.) was added to the ampoule and the solution was left to heat at 100 °C for 1.5 hours, and a colour change was observed from orange to dark brown. The reaction was concentrated *in vacuo* by rotary evaporation before being purified by silica column chromatography, eluted with 100% *n*-pentane – 90/10 *n*-pentane/ethyl acetate gradient. A pale-yellow oil was isolated (30.5 mg, 0.116 mmol, 43%)

^{1}H NMR (400 MHz, $CDCl_3$) δ/ppm: 7.83 (ddt, *J* = 14.0, 8.3, 1.2 Hz, 4H, C**H$_{Ph}$**) , 7.61 – 7.53 (m, 2H, C**H$_{Ph}$**), 7.49 (ddd, *J* = 8.6, 6.6, 3.2 Hz, 4H, C**H$_{Ph}$**), 6.01 (ddd, *J* = 49.5, 7.8, 3.7 Hz, 1H, ***cis*-H**HC=CFPPh$_2$), 5.74 (ddd, *J* = 27.2, 19.9, 3.7 Hz, 1H, ***trans*-H**HC=CFPPh$_2$).

^{13}C NMR (126 MHz, $CDCl_3$) δ/ppm: 163.0 (dd, *J* = 292.5, 101.8 Hz, HHC=**CF**(S=PPh$_2$))), 132.6 (d, *J* = 3.2 Hz), 132.3 (d, *J* = 11.1 Hz), 130.4 (d, *J* = 88.8 Hz), 129.1 (d, *J* = 13.0 Hz), 110.1 (dd, *J* = 24.0, 10.1 Hz).

^{19}F NMR (377 MHz, $CDCl_3$) δ/ppm: -107.6 (ddd, *J* = 61.4, 49.5, 19.9 Hz).

^{19}F{^{1}H} NMR (377 MHz, $CDCl_3$) δ/ppm: -107.6 (d, *J* = 61.4 Hz).

^{31}P NMR (202 MHz, $CDCl_3$) δ/ppm: 35.2 – 34.2 (m).

^{31}P{^{1}H} NMR (162 MHz, $CDCl_3$) δ/ppm: 34.7 (d, *J* = 61.4 Hz).

MS (TOF MS ES$^+$) m/z: calculated for $[C_{14}H_{12}FPS+H]^+$: 263.0454; found: 263.0447.

IR (thin film)/cm^{-1}: 3030 (C-H sp^2 stretch), 2915 (C-H stretch), 1813, 1631 (C=C stretch), 1476 1433, 1178, 1100, 914, 718, 673

6.1 General Procedure of Batch Scale Reactions of HCF_3 with Organolithium Reagent 4·TMEDA

In a N_2 filled glovebox, **4·TMEDA** (43.9 mg, 0.110 mmol, 1 equiv.) was dissolved in 0.6 mL of C_6D_6/THF and was added to a J. Young NMR tube equipped with a standard (known amount of hexafluorobenzene). The solution was degassed once via freeze-pump-thaw and HCF_3 (1 bar, 25 °C, 0.088 mmol, 0.8 equiv.) was added to the J. Young NMR tube. The J. Young NMR tube was inverted several times, and a ^{19}F NMR spectra was recorded. The yield was determined *in situ* upon integral comparison to a known amount of hexafluorobenzene in the ^{19}F NMR spectrum.

6.2 Optimisation of Batch Scale Reactions of HCF_3 with Organolithium Reagent 4·TMEDA

- **Concentration Screen**

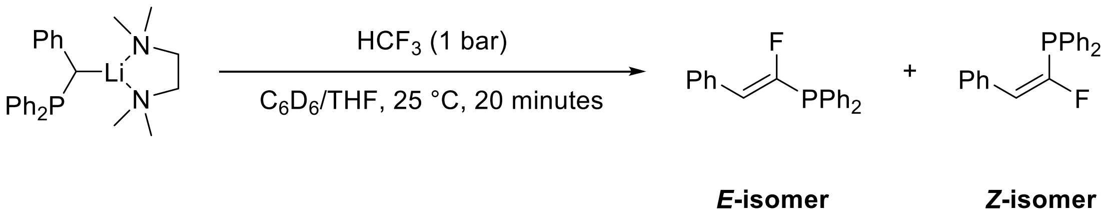

Concentration of organolithum (M) {amount of organolithium (mmol)}	Approx. equivalent of HCF_3 (headspace of NMR tube = 2.2 mL)	Yield (%)[c]	*E*:*Z*
0.20 {0.110}	0.8	68	7.8:1
0.13 {0.080}	1.1	52	5.1:1
0.10 {0.059}	1.5	48	6.9:1
0.037 {0.024}	3.7	22	9.6:1
0.023 {0.014}	6.3	28	99:1

Table S8: Effect of HCF_3 equivalents/concentration on **4·TMEDA**. [c] Yield calculated by ^{19}F NMR spectroscopy using hexafluorobenzene as an internal standard

- **Solvent Screen**

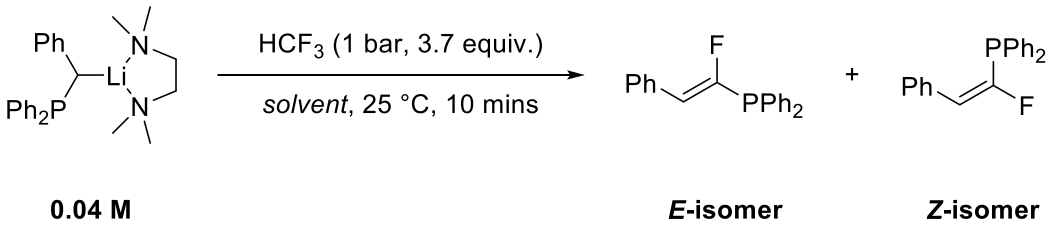

Solvent	Yield (%)[c]	*E*:*Z*
C_6D_6/THF (1:1)	22	9.6:1
THF	19	7.7:1
C_6D_6	8	99:1

Table S9: Results of solvent screen. [c] Yield calculated by ^{19}F NMR spectroscopy using hexafluorobenzene as an internal standard.

6.3 Synthesis of (*E*)-(1-fluoro-2-phenylvinyl)diphenylphosphine sulfide, 8

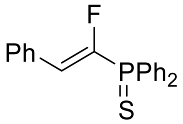

Preparative Procedure: In a N_2 filled glovebox, **4·TMEDA** (0.214 g, 0.537 mmol, 1 equiv.) was dissolved in 5 mL of THF making the concentration of the solution 0.11 M and transferred into an ampoule (Headspace = 15 mL). The solution was degassed *via* freeze-pump-thaw and HCF_3 (1 bar, 25 °C, 0.605 mmol, 1.1 equiv.) was added to the ampoule. The reaction was left to stir for 30 minutes at room temperature and a colour change from pale yellow to orange was observed. Sulfur, S_8, (0.837 g, 3.26 mmol, 6.1 equiv.) was added to the ampoule and the solution was left to heat at 100 °C for 3 days, and a colour change was observed from orange to dark brown. The reaction was concentrated *in vacuo* by rotary evaporation before being purified by silica column chromatography, eluted with 100% *n*-pentane – 90/10 *n*-pentane/ethyl acetate gradient. A yellow oil was isolated (44.4 mg, 0.131 mmol, 49%).

^{1}H NMR (400 MHz, $CDCl_3$) δ/ppm: 7.92 – 7.82 (m, 4H, C**H$_{Ph}$**), 7.68 – 7.61 (m, 2H, C**H$_{Ph}$**), 7.58 – 7.45 (m, 6H, C**H$_{Ph}$**), 7.42 – 7.34 (m, 3H, C**H$_{Ph}$**), 7.20 (dd, J = 41.5, 8.9 Hz, 1H, **H**PhC=CFPPh$_2$).

^{13}C NMR (126 MHz, $CDCl_3$) δ/ppm: 155.8 (dd, *J* = 297.0, 107.8 Hz, H(Ph)C=**C**F(S=PPh$_2$), 132.6 (d, *J* = 3.1 Hz), 132.4 (d, *J* = 11.3 Hz), 132.0 (d, *J* = 12.2 Hz), 131.0 (d, J = 89.5 Hz), 130.4 (d, *J* = 7.5 Hz), 130.0 (s), 129.3 (s), 129.2 (d, *J* = 3.6 Hz), 123.9 (d, *J* = 25.3 Hz).

^{19}F NMR (377 MHz, $CDCl_3$) δ/ppm: -117.7 (dd, *J* = 58.6, 41.5 Hz).

^{19}F{^{1}H} NMR (377 MHz, $CDCl_3$) δ/ppm: -117.7 (d, *J* = 58.8 Hz).

^{31}P NMR (202 MHz, $CDCl_3$) δ/ppm: 36.8 – 35.3 (m).

^{31}P{^{1}H} NMR (162 MHz, $CDCl_3$) δ/ppm: 36.0 (d, *J* = 58.8 Hz).

MS (TOF MS ES$^+$) m/z: calculated for $[C_{20}H_{17}FPS]^+$: 339.0773; found: 339.0776.

IR (thin film)/cm^{-1}: 3053 (C-H sp^2 stretch), 2917 (C-H stretch), 1720 (C=C stretch), 1482, 1437, 1100, 1055, 721, 691.

7.1 Batch Scale Proteodesilylation of Organolithium Reagent 9·TMEDA with HCF_3 and TBAF

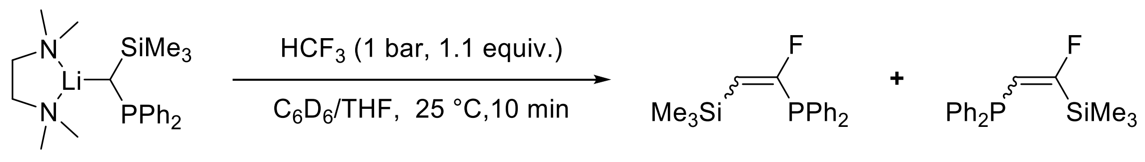

9•TMEDA

0.13 M

80% (Total Yield)
18, 20, 27, 15 %

In a N_2 filled glovebox, **9·TMEDA** (31.1 mg, 0.079 mmol, 1 equiv.) was dissolved in 0.6 mL of C_6D_6/THF and was added to a J. Young NMR tube. The solution was degassed once via freeze-pump-thaw and HCF_3 (1 bar, 25 °C, 0.088 mmol, 1.1 equiv.) was added to the J. Young NMR tube. The J. Young NMR tube was inverted several times, and TBAF 1.0 M in THF (80 µL, 0.079 mmol, 1 equiv.) was added to the J. Young NMR tube. The yield was determined *in situ* upon integral comparison to a known amount of hexafluorobenzene in the ^{19}F NMR spectrum (80%).

^{19}F NMR (377 MHz, None) δ/ppm: -63.7 (dd, *J* = 36.8, 25.5 Hz), -71.0 (dd, *J* = 38.9, 18.2 Hz), -77.8 (t, *J* = 66.4 Hz), -89.6 (dd, *J* = 63.7, 57.8 Hz).

^{19}F{^{1}H} NMR (377 MHz, None) δ/ppm: -63.7 (d, *J* = 25.5 Hz), -71.0 (d, *J* = 18.2 Hz), -77.8 (d, *J* = 66.4 Hz), -89.6 (d, *J* = 63.7 Hz).

^{31}P{^{1}H} NMR (162 MHz, None) δ/ppm: -6.4 (d, *J* = 66.4 Hz), -10.2 (d, *J* = 25.5 Hz), -30.8 (d, *J* = 63.7 Hz), -34.7 (d, *J* = 18.2 Hz).

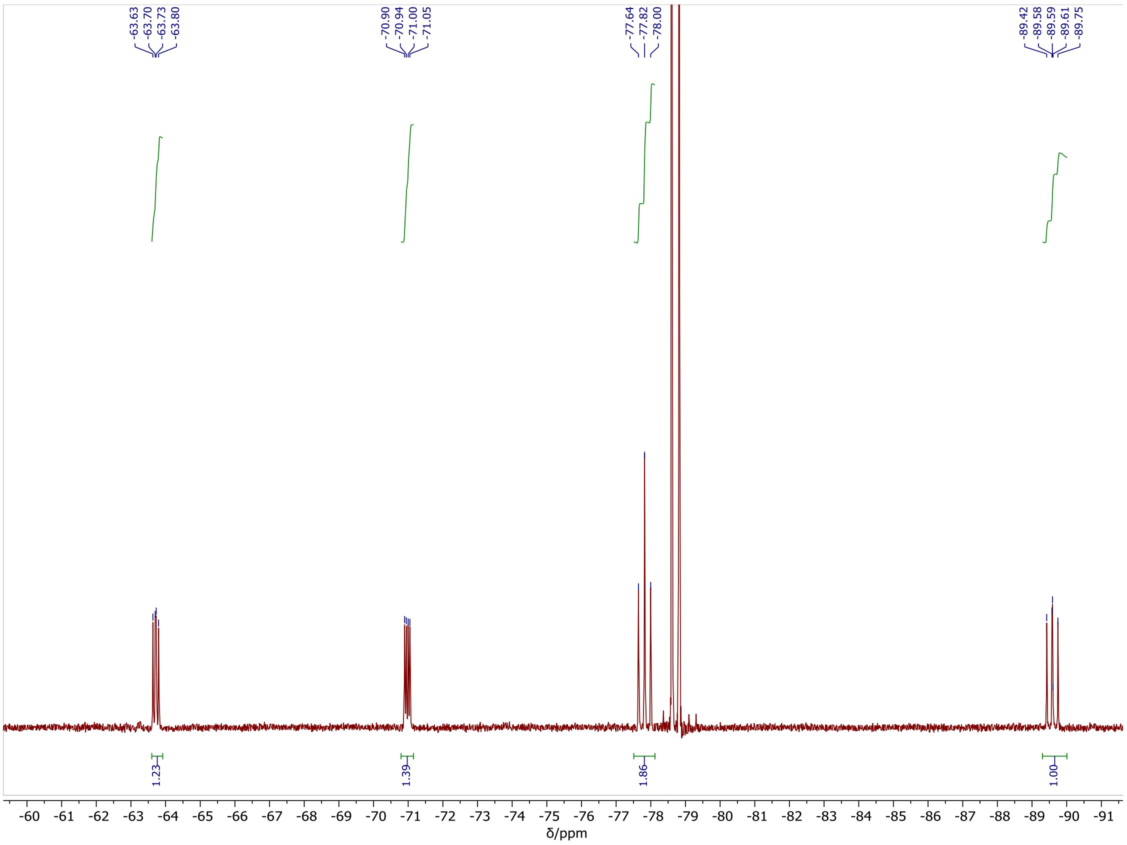

Figure S1: ^{19}F NMR spectra of reaction of **9·TMEDA** + HCF_3.

7.2 General Procedure of Large-Scale Reactions of HCF_3 with 9·TMEDA

In a N_2 filled glovebox, **9·TMEDA** (0.217 g, 0.549 mmol, 1 equiv.) was dissolved in 5 mL of THF making the concentration of the solution 0.11 M and transferred into an ampoule (Headspace = 15 mL). The solution was degassed *via* freeze-pump-thaw and HCF_3 (1 bar, 25 °C, 0.605 mmol, 1.1 equiv.) was added to the ampoule. The reaction mixture was left to stir for 30 minutes at room temperature and a colour change from pale yellow to orange was observed. Sulfur, S_8, (0.243 g, 0.947 mmol, 1.7 equiv.) was added to the ampoule and the reaction mixture was left to heat at 100 °C for 3 days, and a colour change was observed from orange to dark brown. The reaction was concentrated *in vacuo* by rotary evaporation before being purified by silica column chromatography, eluted with 50/50 *n*-hexane/toluene. **S1** was separated and isolated as a single isomer, while **S2** and **S3** were obtained as a mixture.

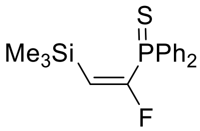

Synthesis of S1: Yellow oil isolated (6.2 mg, 0.0185 mmol, 6.7%) using general procedure.

^{1}H NMR (400 MHz, $CDCl_3$) δ/ppm: 7.82 (ddt, *J* = 13.9, 8.4, 1.2 Hz, 4H, C**H**$_{Ph}$), 7.58 – 7.38 (m, 6H, CH$_{Ph}$), 6.12 (dd, *J* = 47.6, 30.1 Hz, 1H, ***cis*-H(**Me_3Si)C=C**F**(S=**P**Ph_2)), 0.27 (s, 9H, Si(**C**H_3)$_3$).

^{13}C NMR (126 MHz, $CDCl_3$) δ/ppm: 161.3 (dd, *J* = 303.5, 106.1 Hz, C=**C**(F)P), 132.3 (s, **C**H$_{Ph}$), 132.2 (d, *J* = 7.2 Hz, **C**H$_{Ph}$), 131.6 (s, **C**H$_{Ph}$), 129.0 (d, *J* = 13.0 Hz, **C**H$_{Ph}$), 123.1 (dd, *J* = 30.0, 10.4 Hz, **C**=C(F)P), 1.8 (s, Si(**C**H_3)$_3$).

^{19}F NMR (377 MHz, $CDCl_3$) δ/ppm: -61.8 (dd, *J* = 87.3, 47.6 Hz).

^{31}P{^{1}H} NMR (162 MHz, $CDCl_3$) δ/ppm: 35.3 (d, *J* = 87.3 Hz).

^{31}P NMR (162 MHz, $CDCl_3$) δ/ppm: 38.37 – 30.14 (m).

^{29}Si NMR ($CDCl_3$) δ/ppm: -7.59 (s). (from **^{29}Si–^{1}H HMBC**)

MS (TOF MS ES$^+$) m/z: calculated for: calculated for $[C_{17}H_{21}FSiPS]^+$: 335.0855; found: 335.0846.

IR (thin film)/cm^{-1}: 2960 (C-H sp^2 stretch), 2855 (C-H stretch), 1603 (C=C stretch), 1441, 1362, 1254, 1146, 1098, 798.

Synthesis of, S2/3: Yellow oil isolated (27.8 mg, 0.0831 mmol, 30.2 %, 2.9:1) using general procedure.

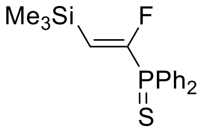

S2:

^{1}H NMR (400 MHz, CDCl$_3$) δ/ppm: 7.80 (ddd, *J* = 13.8, 7.1, 1.2 Hz, 4H, C**H$_{Ph}$**), 7.48 (tdd, *J* = 7.1, 2.5, 1.5 Hz, 6H, C**H$_{Ph}$**), 6.53 (dd, *J* = 65.0, 12.4 Hz, 1H, ***trans***-(Me$_3$Si)**H**C=C(S=**P**Ph$_2$)**F**), 0.21 (s, 9H, Si**(CH$_3$)$_3$**).

^{13}C NMR (126 MHz, CDCl$_3$) δ/ppm: 164.8 (dd, *J* = 289.6, 98.9 Hz, (Me$_3$Si)HC=**C**(S=PPh$_2$)F), 131.9 (d, *J* = 3.1 Hz, CH$_{Ph}$), 131.7 (d, *J* = 11.1 Hz, **C**H$_{Ph}$), 128.6 (d, *J* = 12.9 Hz, **C**H$_{Ph}$), 128.4 (s, **C**H$_{Ph}$), 122.9 (dd, *J* = 19.4, 12.9 Hz, (Me$_3$Si)H**C**=C(S=PPh$_2$)F) -0.92 (s, Si**(CH$_3$)$_3$**).

^{19}F NMR (377 MHz, CDCl$_3$) δ/ppm: -94.1 (dd, *J* = 83.2, 65.0 Hz).

^{31}P{^{1}H} NMR (162 MHz, CDCl$_3$) δ/ppm: 35.8 (d, *J* = 83.2 Hz).

^{31}P NMR (162 MHz, CDCl$_3$) δ/ppm: 36.5 – 35.2 (m).

^{29}Si NMR (CDCl$_3$) δ/ppm: -5.25 (s). (from **^{29}Si–^{1}H HMBC**).

MS (TOF MS ES$^+$) m/z: calculated for: calculated for [$C_{17}H_{21}FSiPS$]$^+$: 335.0855; found: 335.0851.

IR (thin film)/cm^{-1}: 2952 (C-H sp^2 stretch), 2896 (C-H stretch), 1610 (C=C stretch), 1435, 1249, 1101, 1010, 846, 721.

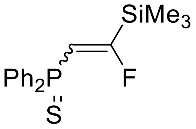

S3: The stereochemistry of S3 is not known.

^{1}H NMR (400 MHz, CDCl$_3$) δ/ppm: 7.90 – 7.82 (m, 4H, C**H$_{Ph}$**), 7.58 – 7.51 (m, 6H, C**H$_{Ph}$**), 6.60 (dd, *J* = 43.5, 11.2 Hz, 1H, **H**Ph$_2$PC=C(**F**)SiMe$_3$), 0.20 (d, *J* = 1.0 Hz, 9H, Si**(CH$_3$)$_3$**).

^{13}C NMR (126 MHz, CDCl$_3$) δ/ppm: 186.4 (dd, *J* = 304.1, 27.7 Hz, H(S=PPh$_2$)C=**C**F(SiMe$_3$)), 134.9 (d, *J* = 88.1 Hz, **C**H$_{Ph}$), 131.3 (d, *J* = 2.9 Hz, **C**H$_{Ph}$), 131.0 (d, *J* = 11.0 Hz, **C**H$_{Ph}$), 130.2 (d, *J* = 87.6 Hz, **C**H$_{Ph}$), 118.4 (dd, *J* = 94.1, 12.2 Hz, H(S=PPh$_2$)**C**=CF(SiMe$_3$)), -1.41 (d, *J* = 3.4 Hz, Si**(CH$_3$)$_3$**).

^{19}F NMR (377 MHz, CDCl$_3$) δ/ppm: -63.7 (dd, *J* = 57.0, 43.5 Hz)

^{31}P{^{1}H} NMR (162 MHz, CDCl$_3$) δ/ppm: 27.9 (d, *J* = 57.0 Hz).

^{31}P NMR (162 MHz, CDCl$_3$) δ/ppm: 28.3 – 27.4 (m).

^{29}Si NMR (CDCl$_3$) δ/ppm: -5.25 (s). (from **^{29}Si–^{1}H HMBC**).

MS (TOF MS ES$^+$) m/z: calculated for: calculated for [$C_{17}H_{21}FSiPS$]$^+$: 335.0855; found: 335.0851.

IR (thin film)/cm^{-1}: 2952 (C-H sp^2 stretch), 2896 (C-H stretch), 1610 (C=C stretch), 1435, 1249, 1101, 1010, 846, 721.

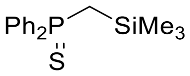

Synthesis of diphenyl((trimethylsilyl)methyl)phosphine sulfide, S4: Yellow crystalline solid isolated (38 mg, 0.125 mmol, 45.4 %) using general procedure.

^{1}H NMR (400 MHz, $CDCl_3$) δ/ppm: 7.92 – 7.78 (m, 4H, C**H_{Ph}**), 7.47 – 7.37 (m, 6H, C**H_{Ph}**), 2.02 (d, J = 16.4 Hz, 2H, C**H_2**P), 0.02 (s, 9H, Si**$(CH_3)_3$**).

^{13}C NMR (101 MHz, $CDCl_3$) δ/ppm: 136.2 (d, J = 80.2 Hz, **C**H_{Ph}), 131.4 (d, J = 3.0 Hz, **C**H_{Ph}), 131.1 (d, J = 10.6 Hz, **C**H_{Ph}), 128.8 (d, J = 12.2 Hz, **C**H_{Ph}), 21.6 (d, J = 47.4 Hz, **C**H_2P), 0.85 (d, J = 3.3 Hz, Si**$(CH_3)_3$**).

^{31}P{^{1}H} NMR (162 MHz, $CDCl_3$) δ/ppm: 38.6 (s).

^{31}P NMR (162 MHz, $CDCl_3$) δ/ppm: 38.6 (p, J = 14.9 Hz).

^{29}Si NMR ($CDCl_3$) δ/ppm: 1.77 (s). (from **^{29}Si–^{1}H HMBC**).

MS (TOF MS ES^{+}) m/z: calculated for: calculated for $[C_{16}H_{22}SiPS]^{+}$: 305.0949; found: 305.0935.

IR (thin film)/cm^{-1}: 2948 (C-H sp^{2} stretch), 2892 (C-H stretch), 1433, 1245, 1098, 835, 688.

8.1 Reaction of HCF_3 with 3·TMEDA in Continuous Flow

General flow procedure using our previously reported optimum conditions.[7] After being primed with dry THF, a 0.11 M solution of organolithium **3·TMEDA** in THF was delivered through peristaltic pump A, set to the desired flowrate 2.0 mL min^{-1}. HCF_3 (3.1 bar) was delivered through peristaltic pump B, set to the desired flowrate 2.5 mL min^{-1} and mixed at a T junction to give segmented flow through the coil reactor (ID = 1 mm, length 495 cm, 3.9 ml reactor volume, T_R = 1 min 3 s).

These conditions in flow were comparable to yields at NMR scale with a combined 72% yield of *Z/E*-4 (3:1 *Z/E*). An extended run of the system using 80 ml solution in THF (0.11 M, **3·TMEDA**, 2.53 g) confirmed the yield within error (67% *in situ*, 51% isolated), giving a productivity of 4.75 mmol h^{-1}. For the extended run, sonication was used to avoid fouling of the reactor system.

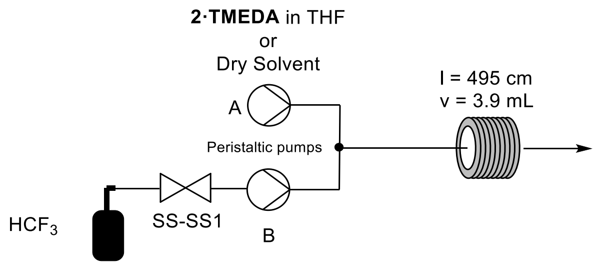

Figure S2: General schematic of **3·TMEDA** with HCF_3 in flow.

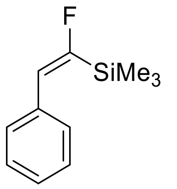

The crude solution was concentrated *in vacuo* by rotary evaporation before being purified by silica column chromatography, eluted with 100% *n*-pentane. A 3:1 mixture of Z/E-1-fluoro-2-phenyl-1-trimethylsilylethene, was isolated as a colourless oil (0.43 g, 2.23 mmol, 51%).

9.1 Carbene Trapping Experiments

The reaction of **3·TMEDA** with HCF_3 was carried out in the presence of 10 equivalents of tetramethylethylene (TME), which is known to form difluoroproponation products in the presence of difluorocarbene.[8] After 10 minutes, product inhibition was observed, and a singlet was observed in the ^{19}F NMR spectrum at -149.4 ppm which is indicative of the formation of difluorocyclopropane. This result suggests the formation of difluorocarbene.[8]

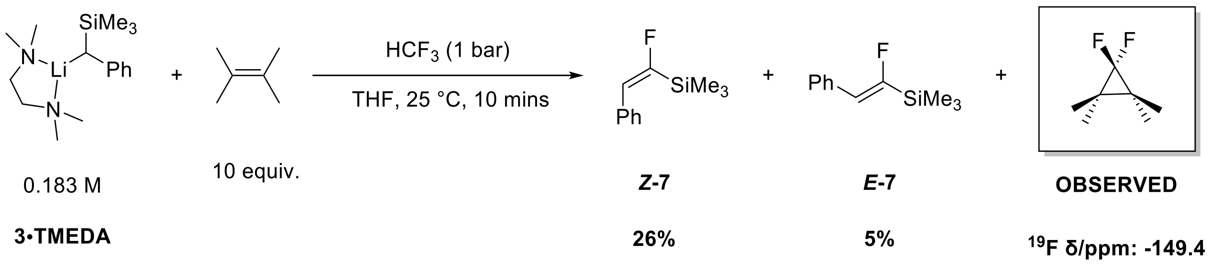

Scheme S1: Tetramethylethylene trapping experiment. Difluorocyclopropane was observed in the ^{19}F NMR spectrum. Yield calculated by ^{19}F NMR spectroscopy using α,α,α-trifluorotoluene as an internal standard.

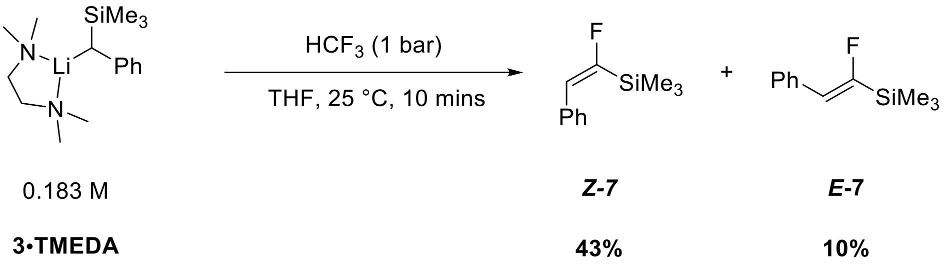

Scheme S2: Standard conditions. Yield calculated by ^{19}F NMR spectroscopy using α,α,α-trifluorotoluene as an internal standard.

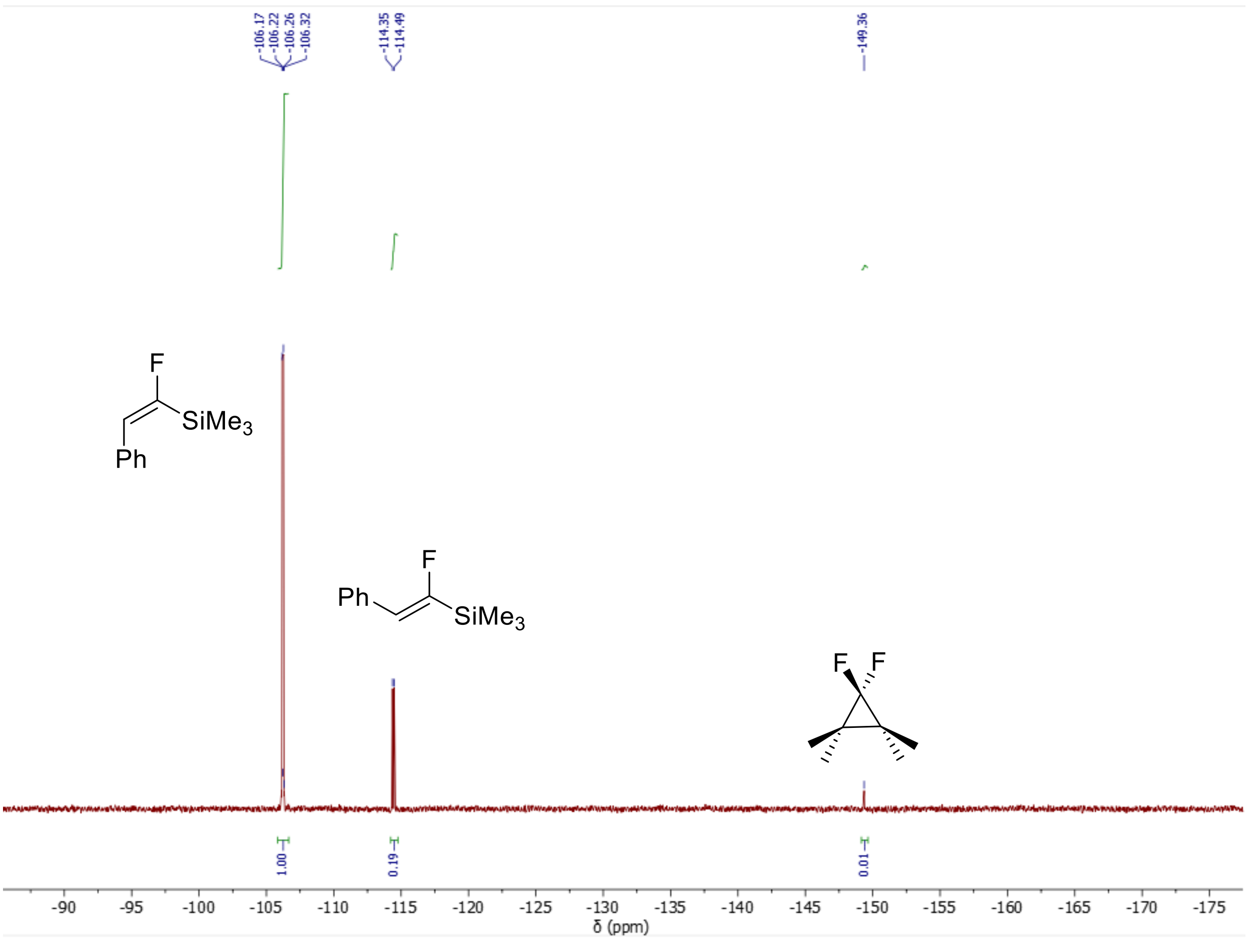

Figure S3: ^{19}F NMR spectrum of carbene trapping experiment. Difluorocyclopropane observed in <5% at -149.4 ppm.

10.1 DOSY Studies of 1, 1·PMDETA, 2·TMEDA, 3·PMTEDA, 3·TMEDA and 3·THF$_{1.5}$, 4·TMEDA, and 9·TMEDA

DOSY spectra were recorded on BRUKER 500 MHz machines at 298K, durene or tetrakis(trimethylsilyl)silane (TTMS) was used as an internal standard in the ^{1}H NMR. All peaks are referenced against residual solvent and values are quoted in ppm. The hydrodynamic radius was calculated by using Stokes-Einstein Equation.[9]

DFT calculations were computed for the experimental results. Geometries were optimised and their volumes were calculated using the B3PW91 functional with 6-31G** basis set for C, H, and 6-311+G* basis set for O, N, Si, Li with solvent corrections (PCM, benzene, ε = 2.2706).

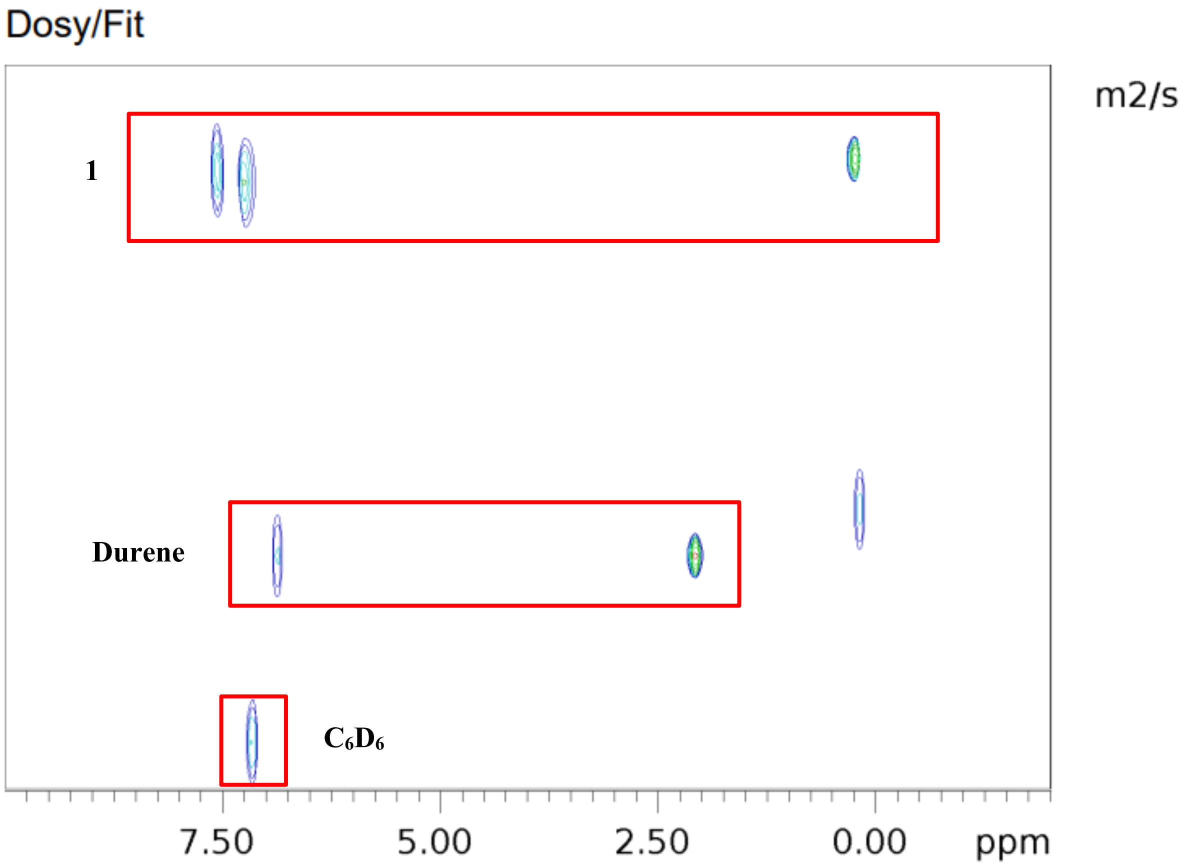

Figure S4: DOSY spectrum of **1** in C_6D_6

Compound diffusion coefficient: 9.88 x 10^{-10} m^2/s

Compound HD radius: 6.99 Å

Compound HD radius (DFT calculation result): 6.96 Å (Tetramer)

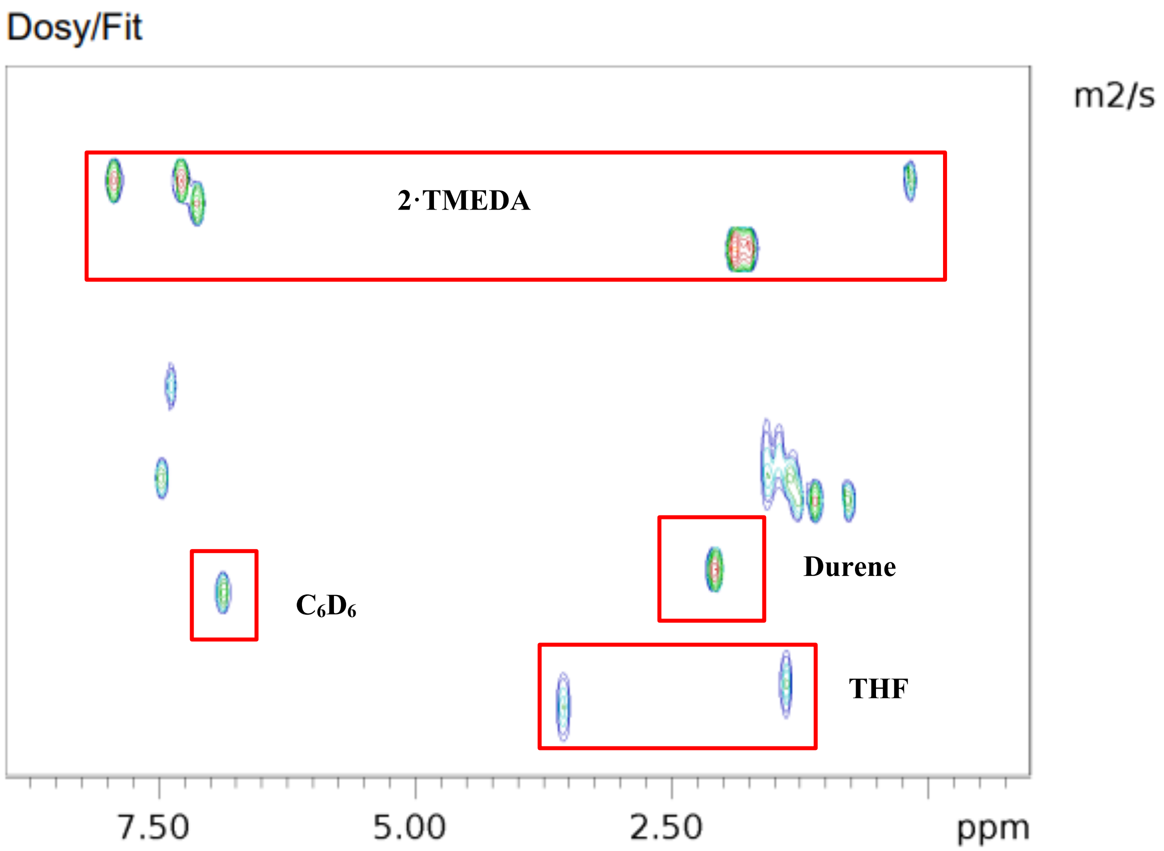

Figure S5: DOSY spectrum of **2·TMEDA** in C_6D_6

Compound diffusion coefficient: 1.39 x 10^{-9} m^2/s

Compound HD radius: 4.99 Å

Compound HD radius (DFT calculation result): 5.96 Å (Monomer), 7.14 Å (Dimer)

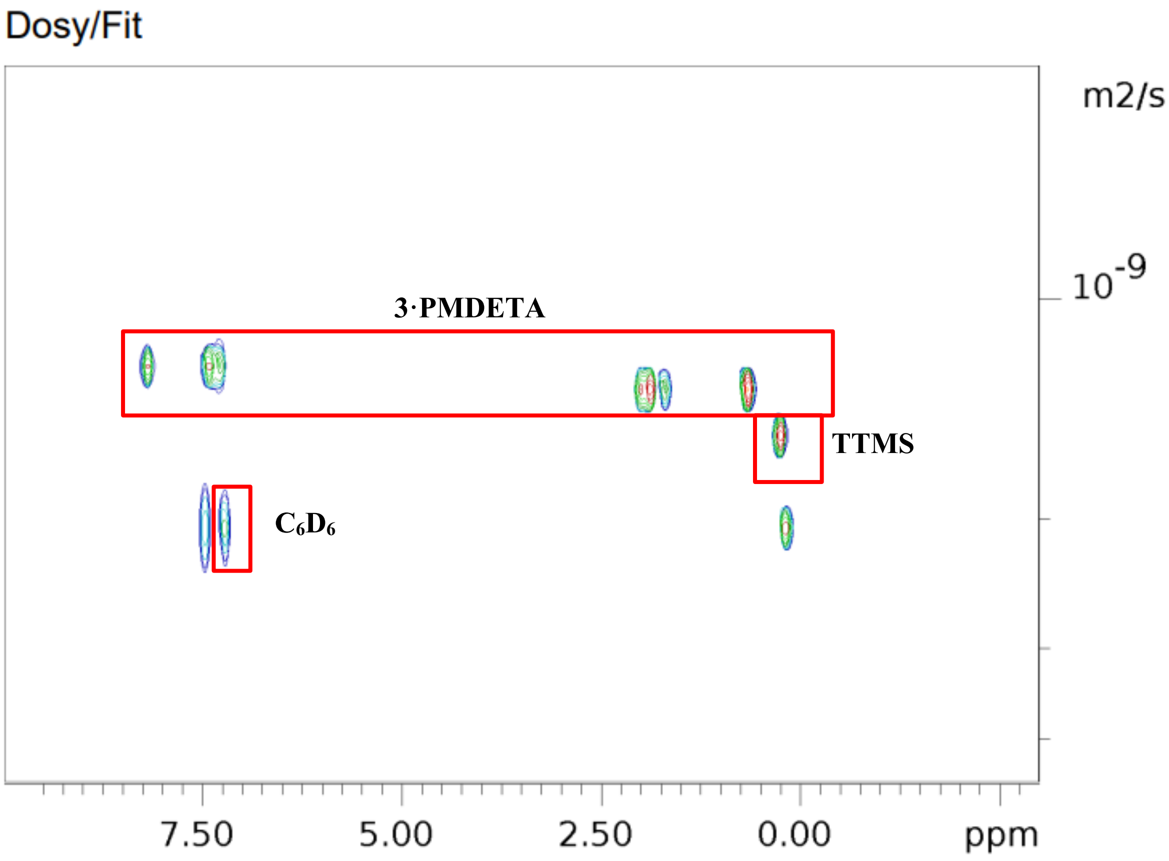

Figure S6: DOSY spectrum of **3·PMDETA** in C_6D_6

Compound diffusion coefficient: 1.29 x 10^{-9} m^2/s

Compound HD radius: 5.20 Å

Compound HD radius (DFT calculation result): 5.92 Å (Monomer)

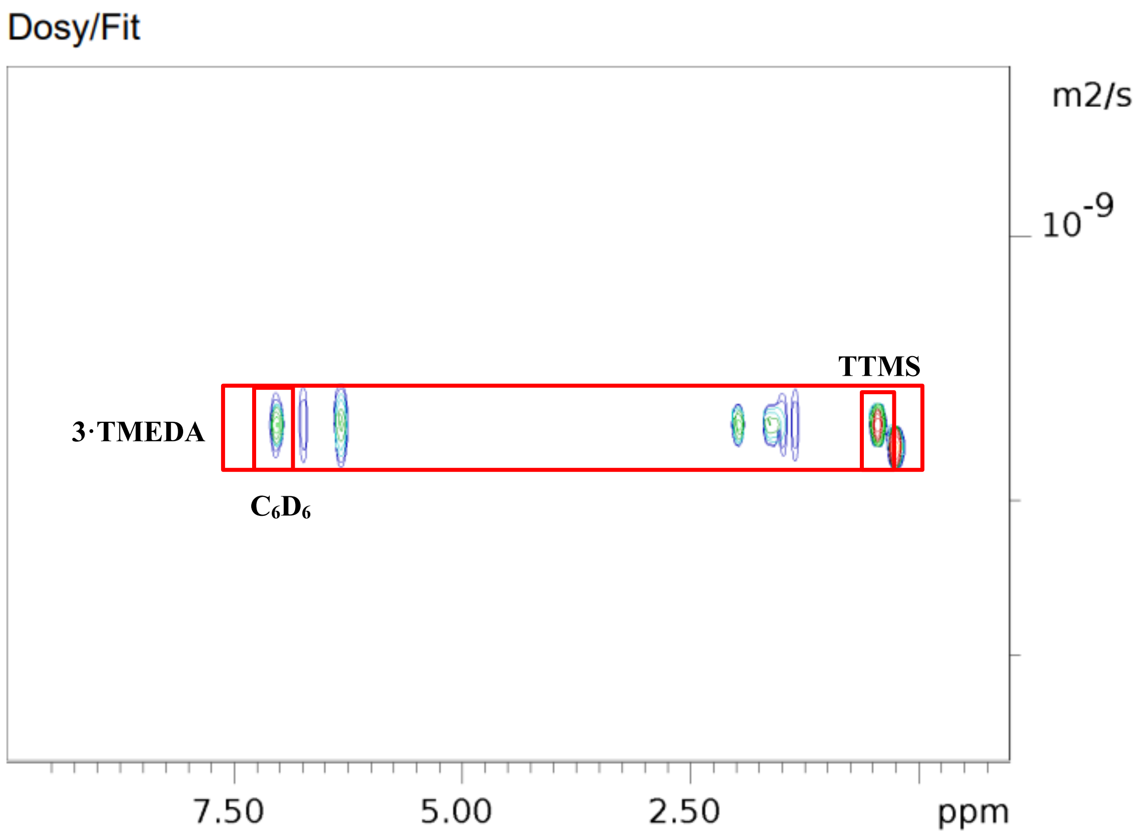

Figure S7: DOSY spectrum of **3·TMEDA** in C_6D_6

Compound diffusion coefficient: 1.66 x 10^{-9} m^2/s

Compound HD radius: 4.74 Å

Compound HD radius (DFT calculation result): 5.71 Å (Monomer)

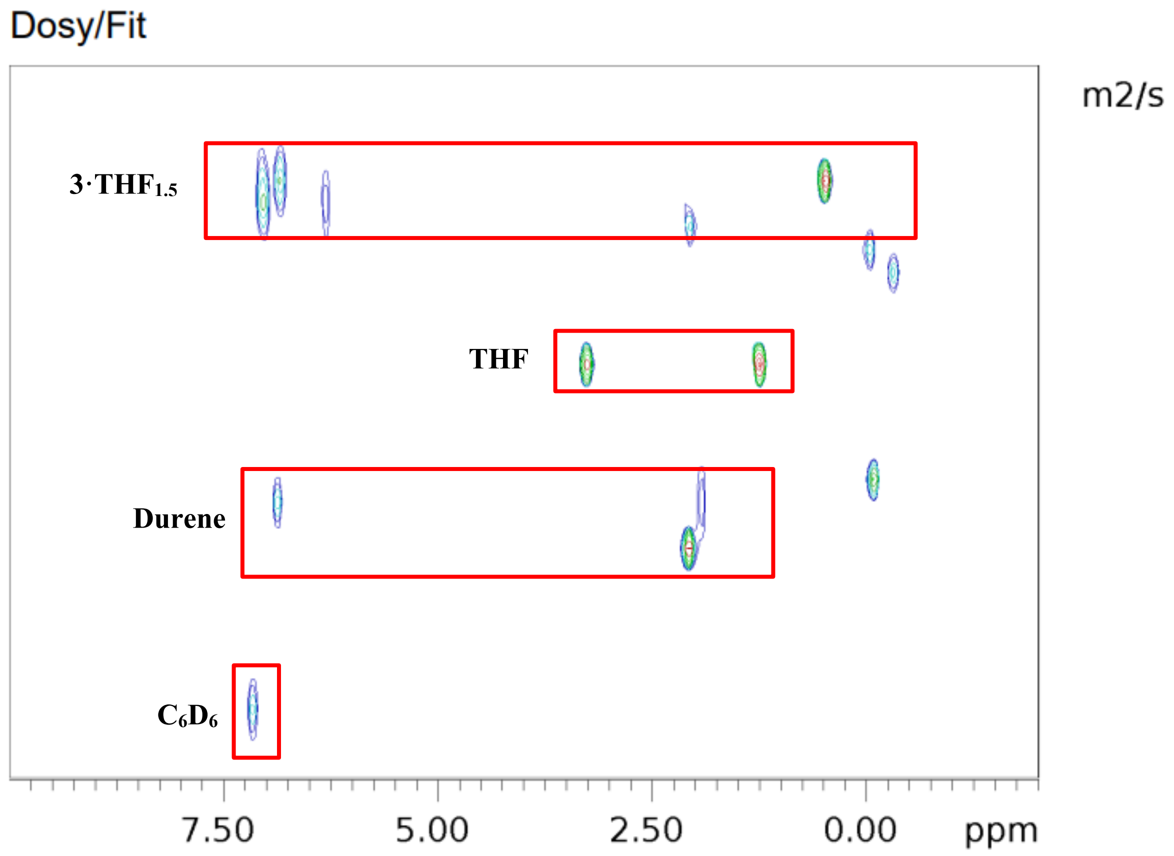

Figure S8: DOSY spectrum of **3·THF$_{1.5}$** in C_6D_6

Compound diffusion coefficient: 1.20 x 10^{-9} m^2/s

Compound HD radius: 5.33 Å

Compound HD radius (DFT calculation result): 5.75 Å (**5** with two THF molecules bonded to Li)

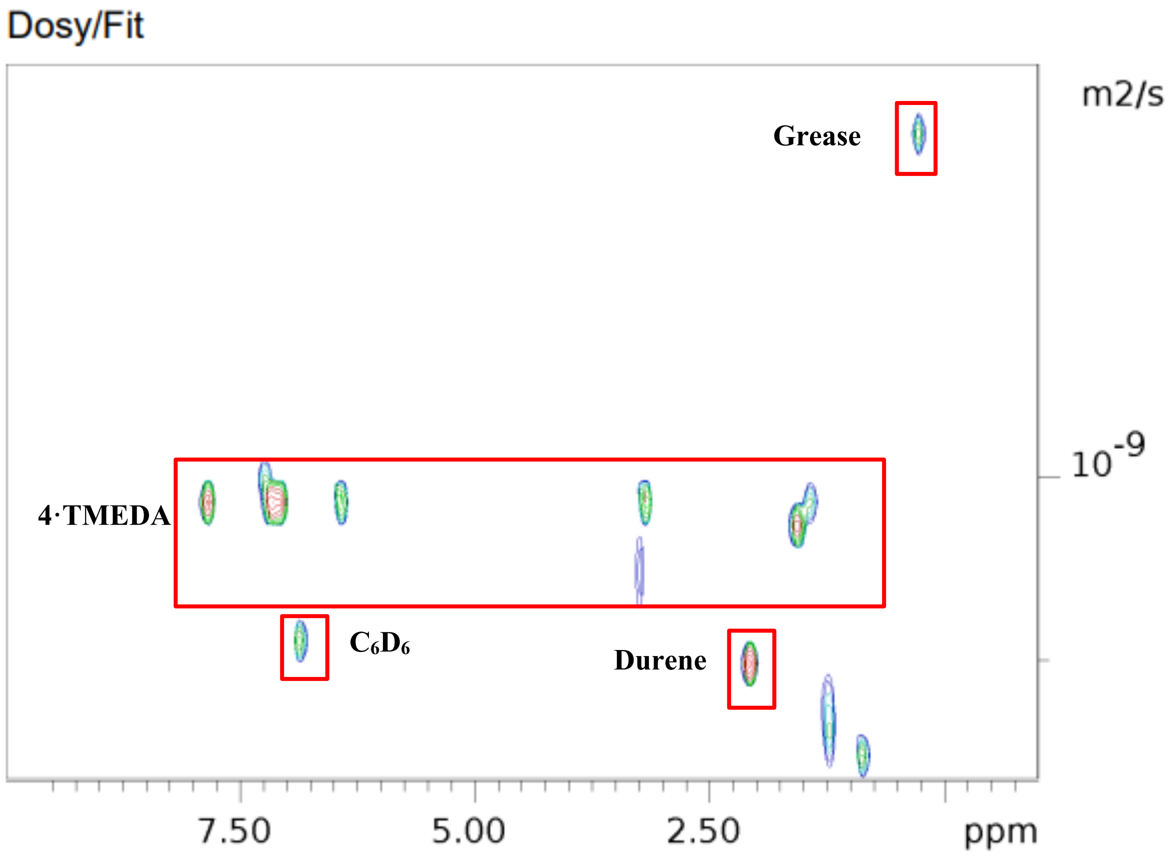

Figure S9: DOSY spectrum of **4·TMEDA** in C_6D_6

Compound diffusion coefficient: 1.10 x 10^{-9} m^2/s

Compound HD radius: 5.50 Å

Compound HD radius (DFT calculation result): 6.17 Å (Monomer)

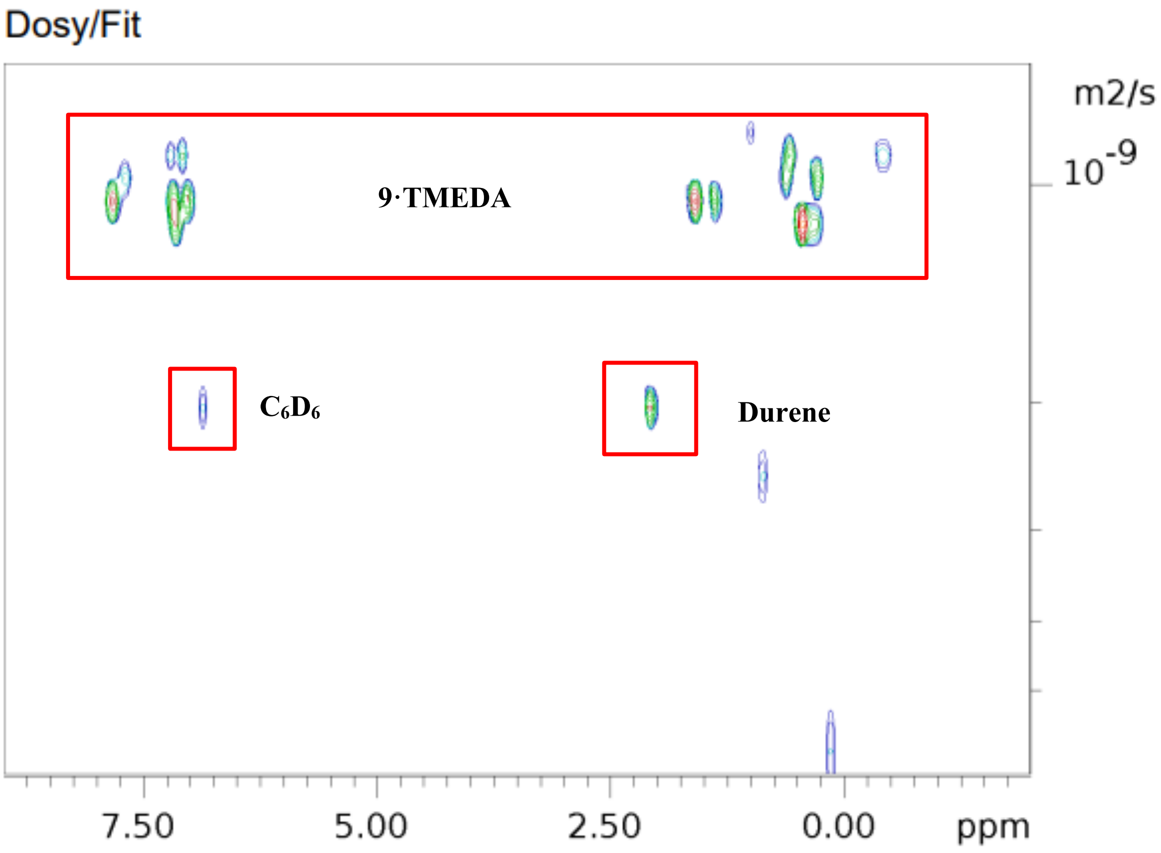

Figure S10: DOSY spectrum of **9·TMEDA** in C_6D_6

Compound diffusion coefficient: 1.12 x 10^{-9} m^2/s

Compound HD radius: 5.14 Å

Compound HD radius (DFT calculation result): 6.12 Å

11 Single Crystal X-ray Diffraction Data

11.1 Summary of Crystal Data

compound	**1**	**3·THF$_{1.5}$**
CCDC number	2345567	2345568
formula	$C_{36}H_{52}Li_4Si_4$	$C_{32}H_{54}Li_2O_3Si_2$
formula weight (g·mol^{-1})	624.89	556.81
colour, habit	colourless block	yellow block
crystal size (mm)	0.12 x 0.07 x 0.05	0.277 x 0.187 x 0.14
crystal system	triclinic	monoclinic
space group	*P*-1 (no. 2)	*C*2/*c* (no. 15)
a (Å)	12.6223(6)	17.3585(5)
b (Å)	12.8053(8)	10.7408(3)
c (Å)	13.0737(8)	19.1760(8)
α (°)	74.620(5)	90
β (°)	77.089(5)	99.760(3)
γ (°)	74.539(5)	90
V (Å³)	1937.0(2)	3523.5(2)
Z	2	4
T (K)	173.00(10)	173.00(14)
D_c (g·cm^{-3})	1.071	1.050
radiation used, μ (mm^{-1})	Cu Kα, 1.567	Cu Kα, 1.110
F(000)	672	1216
absorption correction	multi-scan	analytical
min-max transmission	0.96507 - 1.00000	0.809 - 0.883
Θ range for data collection (°)	3.555 - 73.609	4.862 - 73.666
no. of unique reflns measured, obs [F > 4σ(F)]	7389, 5338	8115, 5668
R_{int}, R_{sigma}	0.0433, 0.0778	0.0300, 0.0302
completeness to Θ(full) (°)	0.983 to 67.684	0.996 to 67.684
no. of parameters, restraints	437, 240	277, 184
R_1, wR_2 [F > 4σ(F)]	0.0449, 0.1046	0.0547, 0.1525
R_1, wR_2 [all data]	0.0689, 0.1223	0.0768, 0.1717
GooF	1.000	1.060

Table S10: A summary of the crystallographic data for the structures of **1** and **3·THF$_{1.5}$**. Data were collected using a Xcalibur PX Ultra A diffractometer, and the structures were solved and refined using the OLEX2,[10] and SHELX-2019.[10-12] program systems. $R_1 = \Sigma||F_o| - |F_c||/\Sigma|F_o|$; $wR_2 = \{\Sigma[w(F_o^2 - F_c^2)^2] / \Sigma[w(F_o^2)^2]\}^{1/2}$; $w^{-1} = \sigma_2(F_o^2) + (aP)^2 + bP$.

compound	**9·TMEDA**	**11f**
CCDC number	2476424	2402208
formula	$C_{22}H_{36}LiN_2PSi_1$	$C_{19}H_{15}FO$
formula weight (g·mol^{-1})	394.53	278.31
colour, habit	colourless thin plate needle	colourless blocky needle
crystal size (mm)	0.358 x 0.156 x 0.13	0.209 x 0.111 x 0.062
crystal system	triclinic	monoclinic
space group	*P*-1 (no. 2)	$P2_1/n$ (no. 14)
a (Å)	7.5792(6)	15.1781(16)
b (Å)	9.3463(9)	5.1026(6)
c (Å)	17.7125(16)	19.077(2)
α (°)	83.898(8)	90
β (°)	80.464(7)	109.559(13)
γ (°)	79.805(8)	90
V (Å^3)	1214.00(19)	1392.3(3)
Z	2	4
T (K)	174(3)	173.1(2)
D_c (g·cm^{-3})	1.079	1.328
radiation used, μ (mm^{-1})	Cu Kα, 1.519	Cu Kα, 0.724
F(000)	428	584
absorption correction	analytical	analytical
min-max transmission	0.722 - 0.858	0.898 - 0.961
Θ range for data collection (°)	4.823 - 73.359	4.550 - 73.454
no. of unique reflns measured, obs [F > 4σ(F)]	4690, 3340	2761, 1885
R_{int}, R_{sigma}	0.0637, 0.0977	0.0605, 0.0669
completeness to Θ(full) (°)	0.987 to 67.684	0.997 to 67.684
no. of parameters, restraints	251, 0	311, 846
R_1, wR_2 [F > 4σ(F)]	0.0818, 0.2142	0.0501, 0.1181
R_1, wR_2 [all data]	0.1039, 0.2556	0.0795, 0.1429
GooF	1.047	1.069

Table S10 continued. A summary of the crystallographic data for the structures of **9·TMEDA** and **11f**.

11.2 Refinement Details

1

The Li-C***H***$_2$ hydrogens were found in the ΔF maps and freely refined.

3·THF$_{1.5}$

The crystal of **3·THF$_{1.5}$** was found to be a two-component twin in a ratio of ca. 0.69:0.31, described by the approximate twin law [-1.00 0.00 -0.00 -0.00 -1.00 -0.00 0.37 -0.01 1.00]. The asymmetric unit contains only one $C_{10}H_{15}Si_1$ fragment, two crystallographically independent Li atoms (at 0.5 occupancy each), and three crystallographically independent THF molecules (at overall 0.5 occupancy each).

The molecule is disordered across a 2-fold rotation axis located along the Li1-O1 bond (hence Li1 and O1 were modelled at 0.5 occupancy). Furthermore, Li2 and the two THF molecules coordinated to it are disordered across a centre of inversion located ca. 2.0 Å from Li2 (and hence it was modelled at 0.5 total occupancy). These two THF molecules were found to be disordered across two symmetry-unique orientations each, at ratio of ca. 0.26:0.24 (O2 and O2A-based THF) and 0.30:0.20 (O3 and O3A-based THF). Only the non-hydrogen atoms of the major orientations were modelled anisotropically, the rest were modelled isotropically. The Li-C***H***(Ph) hydrogen atom H1 was found in the ΔF maps and freely refined.

9·TMEDA

Despite repeated attempts, only twinned, weakly diffracting thin plate needle crystals could be obtained. These were found to be extremely fragile, and most either did not diffract or disintegrated when touched with the nylon loop of the mount under a microscope, under a stream of N_2 gas. The first crystal that gave acceptable diffraction was measured.

While the crystal likely exhibited twinning, modelling this gave a worse model. Nevertheless, the high R_1, wR_2, and R_{int} may be the result of unmodelled twinning. The Li-C***H***(Si)(P) atom H1 was found in the ΔF maps. It was ultimately modelled using the conventional riding model that put it very close to free refinement. While minor disorder of the TMEDA group could be seen in the ΔF maps, including a second orientation gave a worse model.

11f

The phenyl group was found to be disordered. It was modelled in two orientations, with both sharing the C4 carbons atom. The naphthyl group was also found to be disordered. It was modelled in two orientations, with both sharing C10 and C18 carbons atoms. For both the above disorders, the two orientations refined to very close to 0.5:0.5 occupancies using separate free variables, and thus were both modelled using a fixed 0.5:0.5 occupancy ratio. The two orientations were restrained to be similar, and all non-hydrogen atoms were refined anisotropically. The H1, H2 and H3 hydrogens were found in

the ΔF maps. Ultimately, the C–***H*** atoms H1 and H3 were treated using the conventional riding model that put them close to the positions obtained from free refinement. The O–***H*** atom H2 was refined freely.

11.3 Analysis of Crystal Structures

1

1 crystallises as a tetrameric structure in the solid-state (Figure S11). Each lithium atom adopts opposite corners of a distorted cube bridged by 3-centre,2-electron interactions with the CH_2SiMe_2Ph ligand. Bridging Li–C bond distances range between 2.194(5) and 2.298(5) Å. The organic ligand appears constrained and cants back toward the tetrameric core, with coordination at lithium augmented a close interaction with the ipso and ortho carbon atoms of the phenyl group ranging between 2.519(5) and 2.634(5) Å.

3·$THF_{1.5}$

3·$THF_{1.5}$ adopts a dimeric structure in the solid-state (Figure S12). The structure is disordered over both a two-fold rotation axis and an inversion operation. The organic ligand occupies one bridging and one terminal position in the structure. The bridging ligand approaches a side-on interaction with the metals suggestive of charge delocalisation across the phenyl ring and a η^2-binding mode. The remaining coordination at lithium is completed by three molecules of THF.

11.4 Crystal Structures

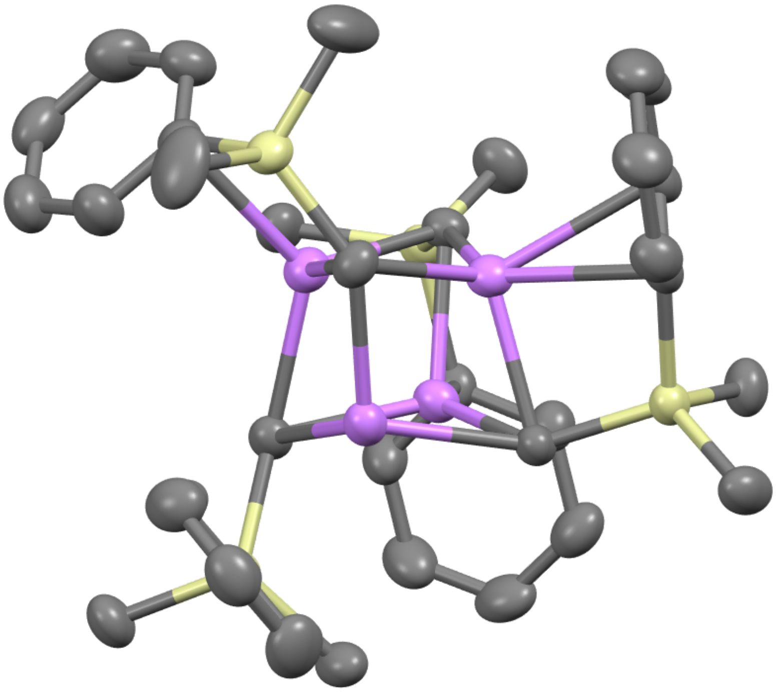

Figure S11: Crystal structure of **1**, thermal ellipsoids are at the 50 % probability level. Hydrogen atoms are hidden for clarity.

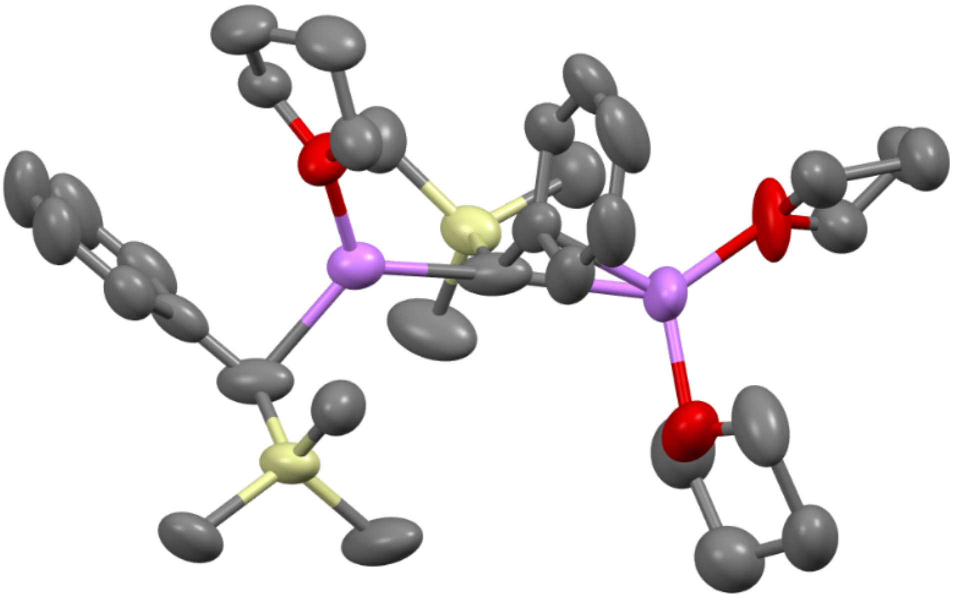

Figure S12: Crystal structure of **3**·**THF**$_{1.5}$, thermal ellipsoids are at the 50 % probability level. Hydrogen atoms and the minor occupancy orientations of the disordered THF molecules are hidden for clarity.

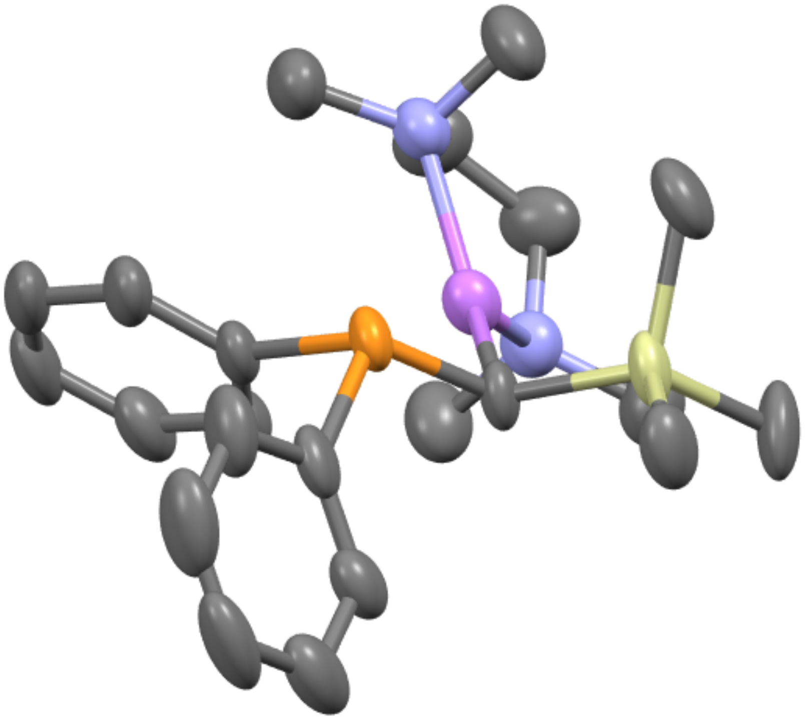

Figure S13: Crystal structure of **9·TMEDA**, thermal ellipsoids are at the 50 % probability level. Hydrogen atoms and the minor occupancy orientations of the disordered THF molecules are hidden for clarity.

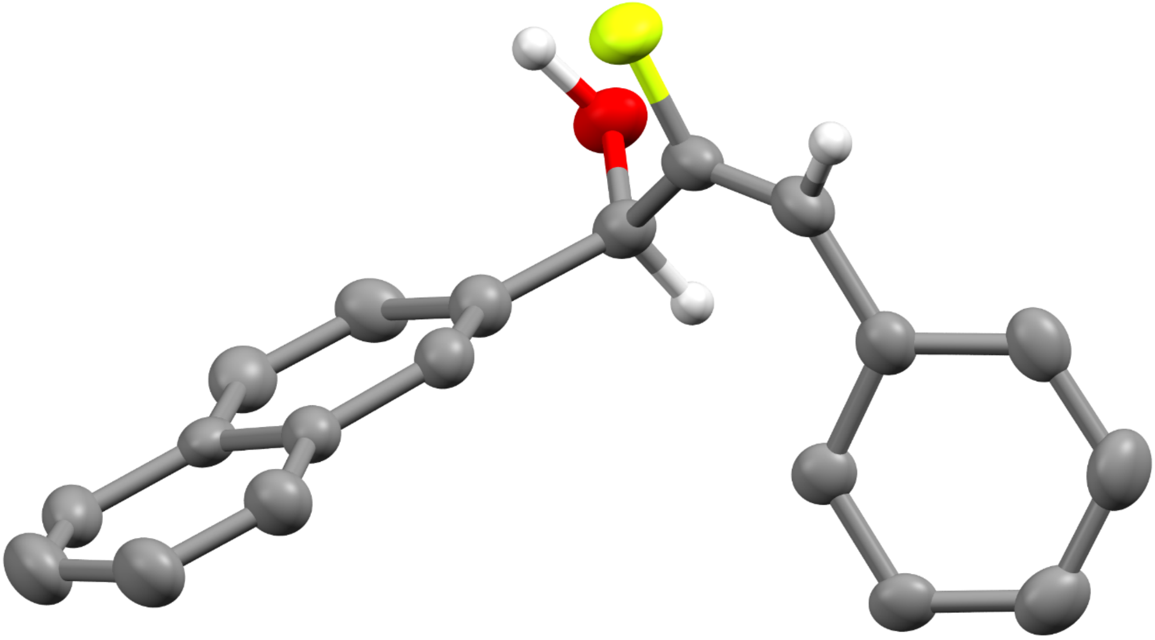

Figure S14: Crystal structure of ***11f***, thermal ellipsoids are at the 50 % probability level. Most hydrogen atoms and the second orientation of the disordered groups are hidden for clarity

12.1 Fluoroalkenylation Reactions

General Procedure for fluoroalkenylation Reactions: Aldehyde (0.22 mmol), fluorovinylsilane ***Z*/*E*-7** mixture (75 mg, 0.39 mmol, 1.5 - 2 equiv.) and TBAF (25.8 µL, 1 M in THF, 12 mol%) were stirred in THF (2 mL) for 30 min in a sealed 20 mL vial in a dinitrogen filled glovebox. TBAF (1 M in THF, 1.5 equiv.) was added, and the reaction mixture was stirred for 30 min before being removed from the glovebox. The reaction mixture was then concentrated *in vacuo* by rotary evaporation. The crude product was purified by silica column chromatography, eluted with *n*-pentane/ethyl acetate 10%, to give the desired fluoroethenylated alcohol products.

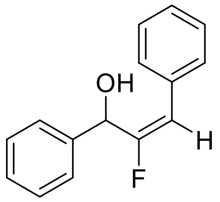

Synthesis of (*E*)-2-fluoro-1,3-diphenylprop-2-en-1-ol, 11a: White solid isolated (22.6 mg, 0.099 mmol, 69%) using general procedure and precipitated using *n*-pentane.

NMR data are consistent with literature.[6]

^{1}H NMR (400 MHz, $CDCl_3$, 298 K) δ/ppm: 7.52 – 7.28 (m, 10H, C**H**$_{Ph}$), 6.52 (d, 1H, $^3J_{HF}$ = 20.3 Hz, C**H**=CF), 5.69 (dd, 1H, $^3J_{HF}$ = 26.9, $^3J_{HH}$ = 7.6 Hz, C**H**(OH)), 2.31 (d, 1H, $^3J_{HH}$ = 7.6 Hz, O**H**).

^{13}C NMR (126 MHz, $CDCl_3$, 298 K) δ/ppm: 159.1 (d, 1C, $^1J_{CF}$ = 257.5 Hz, CH=**C**F), 139.1 (s, 1C, **C**H$_{Ph}$), 132.6 (d, 1C, $^4J_{CF}$ = 12.2 Hz, **C**$^{IV}_{Ph}$), 131.6 (s, 1C, **C**H$_{Ph}$)128.6 (d, $^4J_{CF}$ = 3.6 Hz, 1C, **C**$^{IV}_{Ph}$), 128.2 (s, 1C, **C**H$_{Ph}$), 127.5 (s, 1C, **C**H$_{Ph}$), 126.7 (s, 1C, **C**H$_{Ph}$), 126.4 (s, 1C, **C**H$_{Ph}$), 111.2 (d, $^2J_{CF}$ = 25.6 Hz, 1C, **C**H=CF), 69.2 (d, $^2J_{CF}$ = 25.7 Hz, 1C, **C**H(OH)).

^{19}F NMR (376 MHz, $CDCl_3$, 298 K) δ/ppm: -119.7 (dd, $^3J_{HF}$ = 26.9, $^3J_{HF}$ = 20.3 Hz, 1F, CH=C**F**).

IR (thin film)/cm^{-1}: 3375 (br, OH stretch), 3030, 2920 (C-H stretch), 2898, 1495, 1454, 1222, 1137, 1021, 852, 695.

MS (TOF MS ES$^-$) m/z: calculated for $[C_{15}H_{12}FO–H]^-$: 226.0799; found: 266.0796.

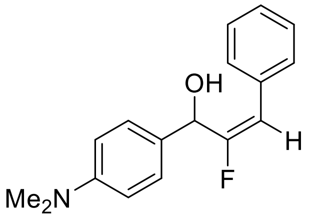

Synthesis of (*E*-1-(4-(dimethylamino) phenyl)-2-fluoro-3-phenylprop-2-en-1-ol, 11b: Cream-white solid isolated (9.1 mg, 0.034 mmol, 23%) using general procedure and precipitated using *n*-pentane.

^{1}H NMR (400 MHz, CDCl$_3$, 298 K) δ/ppm: 7.37 (d, J = 8.6 Hz, 2H, C**H**$_{Ph}$), 7.32 (d, J = 7.3 Hz, 2H, C**H**$_{Ph}$), 7.28 (d, J = 6.9 Hz, 1H. C**H**$_{Ph}$), 7.23 (d, J = 7.4 Hz, 2H, C**H**$_{Ph}$), 6.74 (d, J = 8.8 Hz, 2H, C**H**$_{Ph}$), 6.45 (d, J = 20.6 Hz, 1H, C**H**=CF), 5.59 (dd, $^{3}J_{HF}$ = = 26.5, $^{3}J_{HH}$ = 7.5 Hz, 1H), 2.97 (d, J = 0.9 Hz, 6H, N**Me$_2$**), 2.16 (dd, J = 7.5, 1.0 Hz, 1H, O**H**).

^{13}C NMR (126 MHz, CDCl$_3$, 298 K) δ/ppm: 159.7 (d, 1C, $^{1}J_{CF}$ = 257.9 Hz, CH=**C**F), 150.5 (s, 1C, CH$_{Ph}$), 132.8 (d, 1C, $^{4}J_{CF}$ = 12.7 Hz, **C**^{IV}$_{Ph}$), 128.6 (d, $^{4}J_{CF}$ = 2.8 Hz, 1C, **C**^{IV}$_{Ph}$), 128.4 (s, 1C, **C**H$_{Ph}$), 127.6 (s, 1C, **C**H$_{Ph}$), 127.3 (s, 1C, **C**H$_{Ph}$), 126.9 (s, 1C, **C**H$_{Ph}$), 112.4 (s, 1C, **C**H$_{Ph}$), 110.3 (d, $^{2}J_{CF}$ = 26.0 Hz, 1C, **C**H=CF), 69.1 (d, $^{2}J_{CF}$ = 25.2 Hz, 1C, **C**H(OH)), 40.4 (s, 2C, N**Me$_2$**).

^{19}F NMR (376 MHz, CDCl$_3$, 298 K) δ/ppm: -119.1 (dd, J = 26.4, 20.5 Hz 1F, CH=C**F**).

IR (thin film)/cm^{-1}: 3403 (br, OH stretch), 3028, 2881 (C-H stretch), 2801, 1612, 1521, 1444, 1349, 1224 (C-N stretch), 1133, 1019, 790, 697.

MS (TOF MS ES^{-}) m/z: calculated for [$C_{17}H_{17}FNO$–H]-: 272.1463; found: 272.1451.

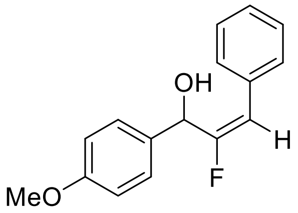

Synthesis of (*E*)-2-fluoro-1-(4-methoxyphenyl)-3-phenylprop-2-en-1-ol, 11c: White solid isolated (36.4 mg, 0.141 mmol, 67%) using general procedure and precipitated using *n*-pentane.

^{1}H NMR (400 MHz, CDCl$_3$, 298 K) δ/ppm: 7.42 (d, 2H, $^3J_{HH}$ = 8.4 Hz, *o*-CH$_{Ph}$), 7.39-7.32 (m, 3H, CH$_{Ph}$), 7.24 (d, 2H, $^3J_{HH}$= 7.4 Hz, CH$_{Ph}$), 6.93 (d, 2H, $^3J_{HH}$ = 8.4 Hz, *m*-CH$_{Ph}$), 6.48 (d, 1H, $^3J_{HF}$ = 20.4 Hz, C**H**=CF), 5.63 (dd, 1H, $^3J_{HF}$ = 26.5 Hz, $^3J_{HH}$ = 7.4 Hz, C**H**(OH)), 3.82 (s, 3H, OC**H**$_3$), 2.28 (d, 1H, $^3J_{HH}$ = 7.4 Hz, O**H**).

^{13}C NMR (100 MHz, CDCl$_3$, 298 K) δ/ppm: 159.6 (s, 1C, **C**$^{IV}_{Ph}$), 159.3 (d, 1C, $^1J_{CF}$ = 256.6 Hz, CH=**C**F), 132.8 (s, 1C, **C**$^{IV}_{Ph}$), 131.4 (s, 1C, **C**$^{IV}_{Ph}$), 128.7 (s, 2C, **C**H$_{Ph}$), 128.6 (s, 2C, **C**H$_{Ph}$), 127.9 (s, 2C, **C**H$_{Ph}$), 127.53 (s, 1C, **C**H$_{Ph}$), 114.1 (s, 2C, **C**H$_{Ph}$), 110.9 (d, 1C, $^3J_{CF}$ = 25.5 Hz, **C**H=CF), 68.9 (d, 1C, $^3J_{CF}$ = 25.7 Hz), 59.3 (s, 1C, O**C**H$_3$).

^{19}F NMR (376 MHz, CDCl$_3$, 298 K) δ/ppm: -119.61 (dd, 1F, $^3J_{HF}$ = 26.5 Hz, $^3J_{HF}$ = 20.4 Hz, C**F** (alkene)).

IR (ATR)/cm^{-1}: 3382 (br, OH stretch), 3006, 1677, 1608, 1509, 1442, 1247, 1142, 1023, 896, 751, 697.

MS (TOF MS ES$^-$) m/z: calculated for $[C_{16}H_{14}FO_2–H]^-$: 257.0983; found: 257.0979.

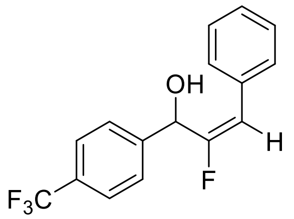

Synthesis of (*E*)-2-fluoro-3-phenyl-1-(4-(trifluoromethyl) phenyl)prop-2-en-1-ol, 11d: White solid isolated (27.6 mg, 0.093 mmol, 63%) using general procedure and precipitated using *n*-pentane.

^{1}H NMR (400 MHz, $CDCl_3$, 298 K) δ/ppm: 7.69 – 7.55 (m, 4H, C**H**$_{Ph}$), 7.43 – 7.30 (m, 3H, C**H**$_{Ph}$), 7.28 (m, 3H, C**H**$_{Ph}$), 6.56 (d, J = 20.0 Hz, 1H, C**H**=CF), 5.74 (d, J = 26.8 Hz, 1H, **H**C-CF), 2.47 (s, 1H, O**H**).

^{13}C NMR (101 MHz, $CDCl_3$, 298 K) δ/ppm: 159.0 (d, 1C, $^1J_{CF}$ = 257.5 Hz, CH=**C**F), 143.5 (s, 1C, **C**H$_{Ph}$), 132.8 (d, 1C, $^4J_{CF}$ = 12.0 Hz, **C**$^{IV}_{Ph}$), 131.1 (s, 1C, **C**H$_{Ph}$), 130.8 (s, 1C, **C**H$_{Ph}$), 129.3 (s, 1C, **C**H$_{Ph}$), 129.1 (d, $^4J_{CF}$ = 2.6 Hz, 1C, **C**$^{IV}_{Ph}$), 128.3 (s, 1C, **C**H$_{Ph}$), 127.3 (s, 1C, **C**H$_{Ph}$), 126.1 (q, 1C, $^1J_{CF}$ = 3.8 Hz, **C**F$_3$), 112.5 (d, $^2J_{CF}$ = 24.8 Hz, 1C, **C**H=CF), 68.3 (d, $^2J_{CF}$ = 26.3 Hz, 1C, **C**H(OH)).

^{19}F NMR (377 MHz, $CDCl_3$, 298 K) δ/ppm: -62.6 (s, 3F, C**F**$_3$), -120.2 (dd, J = 26.9, 19.9 Hz, 1F, CH=C**F**).

IR (thin film)/cm^{-1}: 3358 (br, OH stretch), 3068, 2928 (C-H stretch), 1681, 1623, 1325 (C-F stretch), 1167, 1126, 1070, 1018, 895, 697.

MS (TOF MS ES$^-$) m/z: calculated for [$C_{16}H_{11}F_4O$-H]-: 295.0752; found: 295.0752.

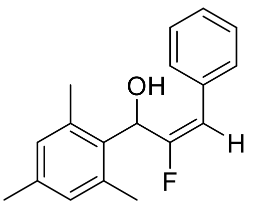

Synthesis of (*E*)-2-fluoro-1-mesityl-3-phenylprop-2-en-1-ol, 11e: White solid isolated (24.2 mg, 0.090 mmol, 62%) using general procedure.

^{1}H NMR (400 MHz, CDCl$_3$, 298 K) δ/ppm: 7.31 – 7.25 (m, 3H, C**H**$_{Ph}$), 7.25 – 7.11 (m, 2H, C**H**$_{Ph}$), 6.72 (s, 2H, C**H**$_{Ph}$), 6.31 (d, J = 20.2 Hz, 1H, C**H**=CF), 5.83 (dd, $^3J_{HF}$ = 28.4, $^3J_{HH}$ = 4.5 Hz, 1H), 2.19 (d, J = 9.0 Hz, 1H, O**H**), 2.15 (s, 3H, **Me**), 2.13 (s, 6H, **Me**).

^{13}C NMR (101 MHz, CDCl$_3$, 298 K) δ/ppm: 160.0 (d, 1C, $^1J_{CF}$ = 260.8 Hz, CH=**C**F), 137.9 (s, 1C, **C**H$_{Ph}$), 137.5 (s, 1C, **C**H$_{Ph}$), 133.7 (d, 1C, $^4J_{CF}$ = 12.3 Hz, **C**$^{IV}_{Ph}$), 132.3 (s, 1C, **C**H$_{Ph}$), 130.6 (s, 1C, **C**H$_{Ph}$), 129.1 (d, $^4J_{CF}$ = 2.7 Hz, 1C, **C**$^{IV}_{Ph}$), 129.0 (s, 1C, **C**H$_{Ph}$), 127.9 (s, 1C, **C**H$_{Ph}$), 112.0 (d, $^2J_{CF}$ = 27.6 Hz, 1C, **C**H=CF), 68.7 (d, $^2J_{CF}$ = 25.2 Hz, 1C, **C**H(OH)), 21.2 (s, 1C, **Me**), 21.1 (d, $^6J_{CF}$ = 3.9 Hz, 2C, **Me**).

^{19}F NMR (377 MHz, CDCl$_3$, 298 K) δ/ppm: -111.2 (dd, J = 28.6, 20.1 Hz, 1F, CH=C**F**).

IR (thin film)/cm^{-1}: 3330 (br, OH stretch), 3027, 2919 (C-H stretch), 2859, 1610, 1450, 1444, 1213, 1141, 1049, 857, 699.

MS (TOF MS ES$^-$) m/z: calculated for [$C_{18}H_{18}FO$-H]- 269.1347; found: 269.1347.

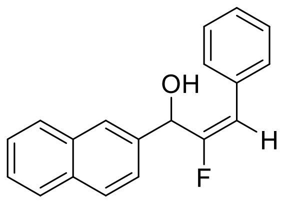

Synthesis of (*E*)-2-fluoro-1-(naphthalen-2-yl)-3-phenylprop-2-en-1-ol, 11f: White solid isolated (57.8 mg, 0.208 mmol, 80%) using general procedure and precipitated using *n*-pentane.

^{1}H NMR (400 MHz, $CDCl_3$, 298 K) δ/ppm: δ 7.95 (s, 1H, C**H**$_{Ar}$), 7.91 – 7.85 (m, 3H, C**H**$_{Ar}$), 7.61 (d, 1H, $^{3}J_{HH}$ = 8.6 Hz, C**H**$_{Ar}$), 7.57 – 7.52 (m, 2H, C**H**$_{Ar}$), 7.43 – 7.37 (m, 2H (C**H**$_{Ar}$), 7.36 – 7.32 (m, 3H, C**H**$_{Ar}$)), 6.60 (d, 1H, $^{3}J_{HF}$ = 20.2 Hz, C**H**=CF), 5.88 (dd, 1H, $^{3}J_{HF}$ = 26.6 Hz, $^{3}J_{HH}$ = 7.6 Hz, C**H**(OH)), 2.56 (d, 1H, $^{3}J_{HH}$ = 7.6 Hz, O**H**).

^{13}C NMR (100 MHz, $CDCl_3$, 298 K) δ/ppm: 159.2 (d, 1C, $^{1}J_{CF}$ = 257.5 Hz, CH=**C**F), 136.6 (s, 1C, **C**$^{IV}_{Ar}$), 133.2 (d, 2C, $^{4}J_{CF}$ = 6.0 Hz, **C**$^{IV}_{Ar}$), 132.7 (d, 1C, $^{4}J_{CF}$ = 12.3 Hz, **C**$^{IV}_{Ar}$), 128.7 (s, 4C, **C**H$_{Ar}$), 128.6 (s, 1C, **C**H$_{Ar}$), 128.2 (s, 1C, **C**H$_{Ar}$), 127.7 (d, 2C, $^{5}J_{CF}$ = 4.1 Hz, **C**H$_{Ar}$), 126.4 (d, 2C, $^{5}J_{CF}$ = 3.2 Hz, **C**H$_{Ar}$), 125.4 (s, 1C, **C**H$_{Ar}$), 124.4 (s, 1C, **C**H$_{Ar}$), 111.5 (d, 1C, $^{2}J_{CF}$ = 25.3 Hz, **C**H=CF), 69.5 (d, 1C, $^{2}J_{CF}$ = 25.9 Hz, **C**H(OH)).

^{19}F NMR (376 MHz, $CDCl_3$, 298 K) δ/ppm: -119.14 (dd, 1F,$^{3}J_{HF}$ = 26.6, $^{3}J_{HF}$ = 20.2 Hz, CH=C**F**).

IR (ATR)/cm^{-1}: 3368 (br, OH stretch), 3062, 1686, 1597, 1498, 1453, 1218, 1136, 1045, 851, 777, 749, 695.

MS (TOF MS ES^{-}) m/z: calculated for $[C_{19}H_{14}FO–H]^{-}$: 277.1034; found: 277.1035.

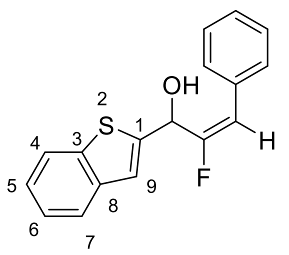

Synthesis of (*E*)-1-(benzo[b]thiophen-2-yl)-2-fluoro-3-phenylprop-2-en-1-ol, 11g: White solid isolated (32.4 mg, 0.114 mmol, 57%) using general procedure and precipitated using *n*-hexane.

^{1}H NMR (400 MHz, CDCl$_3$, 298 K) δ/ppm: 7.84 (d, 1H, $^{3}J_{HH}$ = 7.8 Hz, 8-C**H**$_{Ar}$), 7.76 (d, 1H, $^{3}J_{HH}$ = 7.8 Hz, 4-C**H**$_{Ar}$), 7.39 – 7.34 (m, 10h, C**H**$_{Ar}$), 7.32 (d, 1H, $^{3}J_{HH}$ = 6.9 Hz, C**H**$_{Ar}$), 7.29 – 7.25 (m, 2H, C**H**$_{Ar}$), 6.58 (d, 1H, $^{3}J_{HF}$ = 19.7 Hz, C**H**=CF), 5.92 (dd, 1H, $^{3}J_{HF}$ = 25.0 Hz, $^{3}J_{HH}$ = 8.0 Hz, C**H**(OH)), 2.67 (d, 1H, $^{3}J_{HH}$ = 8.0 Hz, O**H**).

^{13}C NMR (100 MHz, CDCl$_3$, 298 K) δ/ppm: 157.9 (d, 1C, $^{1}J_{CF}$ = 257.6 Hz, CH=**C**F), 143.6 (s, 1C, **C**$^{IV}_{Ar}$), 140.1 (s, 1C, **C**$^{IV}_{Ar}$), 139.5 (s, 1C, **C**$^{IV}_{Ar}$), 132.2 (d, 1C, $^{4}J_{CF}$ = 12.1 Hz, **C**$^{IV}_{Ar}$), 128.9 (s, 2C, **C**H$_{Ar}$), 128.8 (s, 2C, **C**H$_{Ar}$), 127.9 (s, 1C, **C**H$_{Ar}$), 124.7 (s, 1C, **C**H$_{Ar}$), 124.5 (s, 1C, **C**H$_{Ar}$), 123.8 (s, 1C, **C**H$_{Ar}$), 122.5, (s, 1C, **C**H$_{Ar}$), 122.1 (s, 1C, **C**H$_{Ar}$), 111.5 (d, 1C, $^{3}J_{CF}$ = 24.5 Hz, **C**H=CF), 66.4 (d, 1C, $^{3}J_{CF}$ = 26.1 Hz, **C**H(OH)).

^{19}F NMR (376 MHz, CDCl$_3$, 298 K) δ/ppm: -120.62 (dd, 1F, $^{3}J_{HF}$ = 25.0 Hz, $^{3}J_{HF}$ = 19.7 Hz, CH=C**F**).

IR (ATR)/cm^{-1}: 3326 (br, OH stretch), 3060, 1686, 1455, 1146, 1103, 1036, 851, 736, 695.

MS (TOF MS ES^{-}) m/z: calculated for [$C_{17}H_{12}FOS$-H]$^{-}$: 283.0598; found: 283.0599.

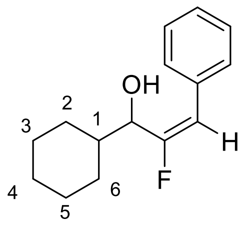

Synthesis of (*E*)-1-cyclohexyl-2-fluoro-3-phenylprop-2-en-1-ol, 11h: Pale yellow oil isolated (32.8 mg, 0.140 mmol, 62%) using general procedure. Isolated as a mixture of E/Z isomers 10:1.

^{1}H NMR (400 MHz, CDCl$_3$, 298 K) δ/ppm: E isomer: 7.34 (m, 2H, *m*-C**H**$_{Ph}$), 7.26 (m, 3H, *o,p*-C**H**$_{Ph}$), 6.38 (d, 1H, $^3J_{HF}$ = 21.6 Hz, C**H**=CF), 4.18 (dm, 1H, $^3J_{HF}$ = 29.3 Hz, C**H**(OH)), 2.04 (d, 1H, $^3J_{HH}$ = 12.3 Hz, 1-C**H**), 1.93 – 1.49 (m, 10h, 1-C**H**, 2,6-C**H$_2$**), 1.36 – 1.03 (m, 4H, 3,5-C**H$_2$**), 1.05 – 0.77 (m, 2H, 4-C**H$_2$**). **Z isomer:** 7.50 (d, 2H, $^3J_{HH}$ = 7.6 Hz, *m*-C**H**$_{Ph}$), 5.73 (d, 1H, $^3J_{HF}$ = 39.5 Hz), 3.93 (dm, 1H, $^3J_{HF}$ = 18.3 Hz).
Partial assignment of minor isomer due to overlapping peaks.

^{13}C NMR (100 MHz, CDCl$_3$, 298 K) δ/ppm: E isomer: 160.4 (d, 1C, $^1J_{CF}$ = 257.0 Hz, CH=**C**F), 133.0 (d, 1C, $^3J_{CF}$ = 13.1 Hz, i-**C**$^{IV}_{Ph}$), 128.8 (s, 2C, *o*-CH$_{Ph}$), 128.5 (s, 2C, *m*-**C**H$_{Ph}$), 127.2 (s, 1C, *p*-**C**H$_{Ph}$), 111.2 (d, 1C, $^2J_{CF}$ = 26.2 Hz, **C**H=CF), 72.3 (d, 1C, $^2J_{CF}$ = 26.3 Hz, **C**H(OH)), 40.9 (s, 1C, 1-**C**H), 29.3 (s, 1C, 2-**C**H), 29.2 (s, 1C, 6-**C**H), 26.2 (s, 1C, 4-**C**H), 25.9 (s, 1C, 3-**C**H), 25.8 (s, 1C, 6-**C**H). **Z isomer:** 128.5 (s, 2C, *m*-**C**H$_{Ph}$), 107.3 (m, 1C, **C**HCF), 76.2 (d, $^2J_{CF}$ = 28.8 Hz, **C**H(OH)), 41.4 (s, 1C, 1-**C**H), 28.3 (s, 1C, 6-**C**H), 26.3 (s, 1C, 3-**C**H), 26.0 (s, 1C, 6-**C**H).
Partial assignment of minor isomer due to low resonance and overlapping peaks.

^{19}F NMR (376 MHz, CDCl$_3$, 298 K) δ/ppm: *Z*-isomer: -117.19 (dd, 1F, $^3J_{HF}$ = 39.5, $^3J_{HF}$ = 18.3 Hz, *Z*-CH=C**F**), ***E*-isomer:** -120.41 (dd, 1F, $^3J_{HF}$= 29.3, $^3J_{HF}$ = 21.6 Hz, *E*-CH=C**F**).

IR (thin film)/cm^{-1}: 3369 (br, OH stretch), 3032, 2922 (C-H stretch), 2851, 1680, 1478, 1446, 1142, 1012, 751, 675.

MS (TOF MS ES$^-$) m/z: calculated for [C$_{15}$H$_{18}$FO-H]$^-$: 233.1347; found: 233.1346.

13.1 IR Spectra

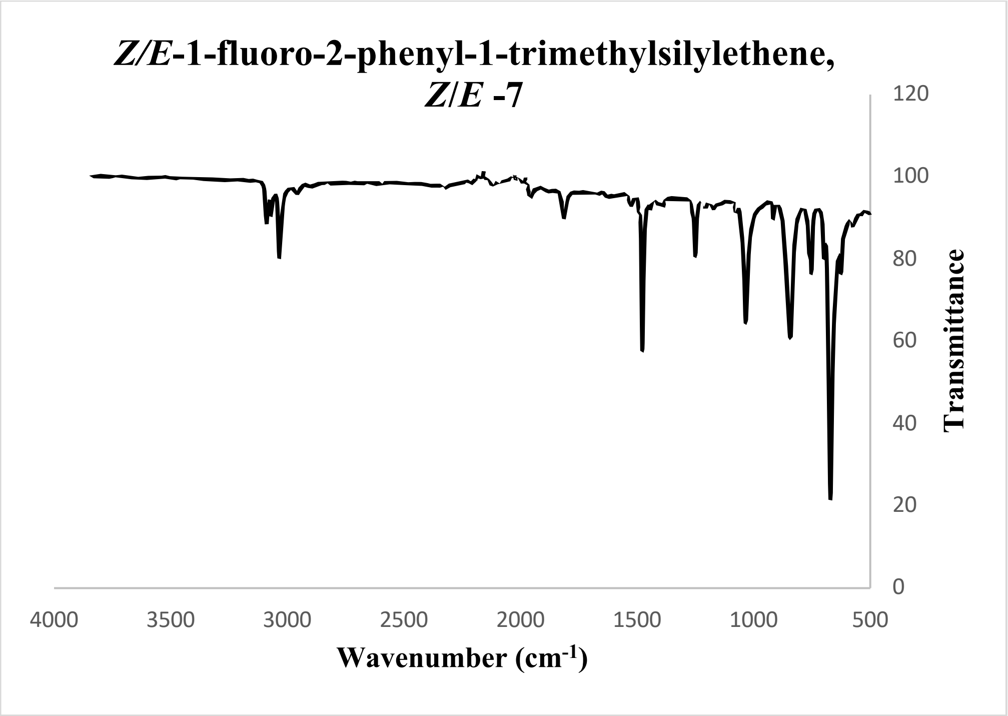

IR Spectra of Z/E-1-fluoro-2-phenyl-1-trimethylsilylethene, *Z*/*E*-7

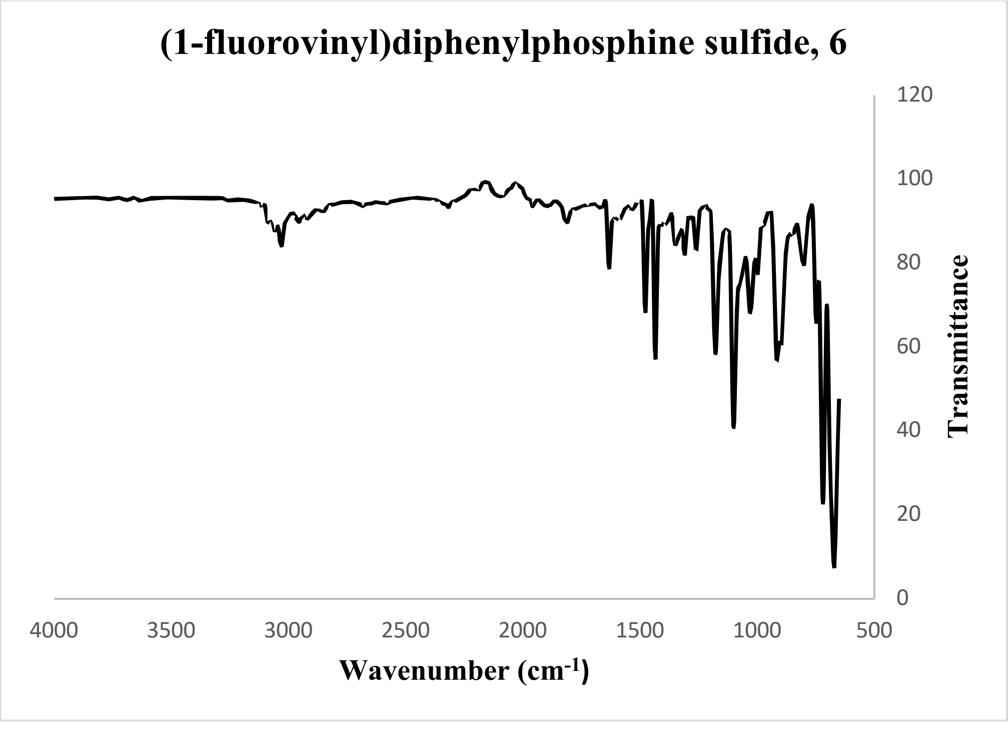

IR Spectra of (1-fluorovinyl)diphenylphosphine sulfide, 6

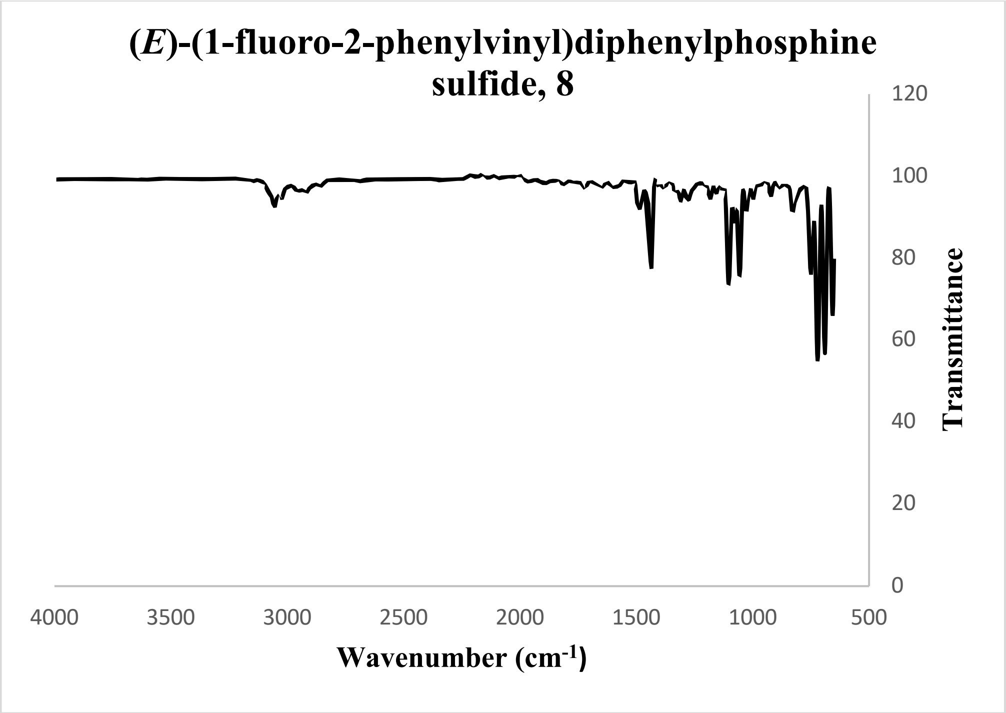

IR Spectra of (E)-(1-fluoro-2-phenylvinyl)diphenylphosphine sulfide, 8

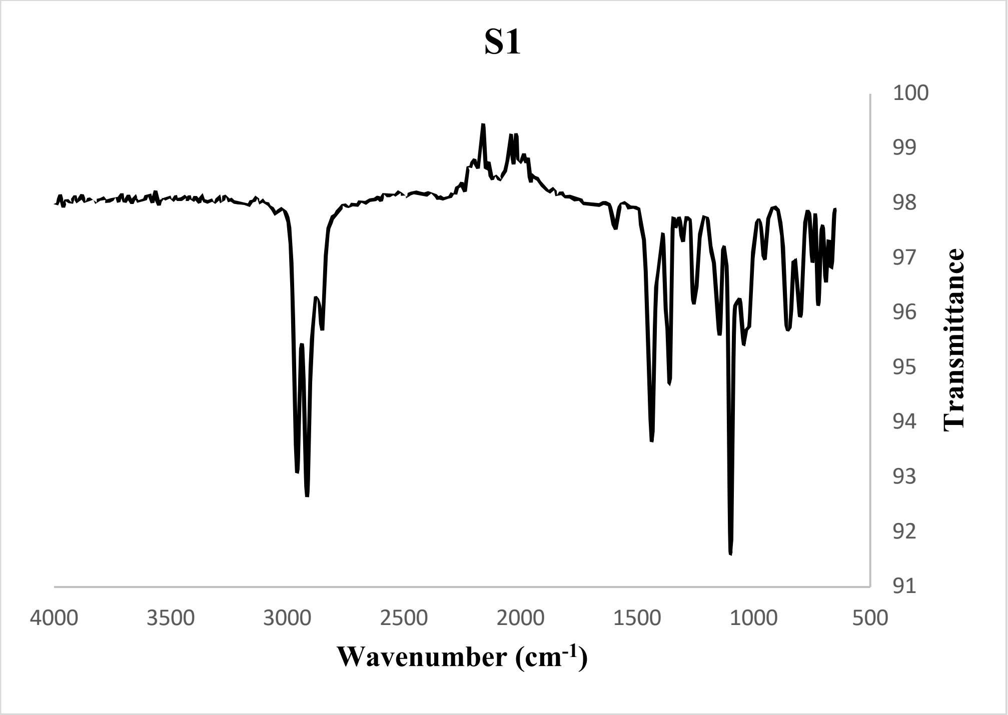

IR Spectra of S1

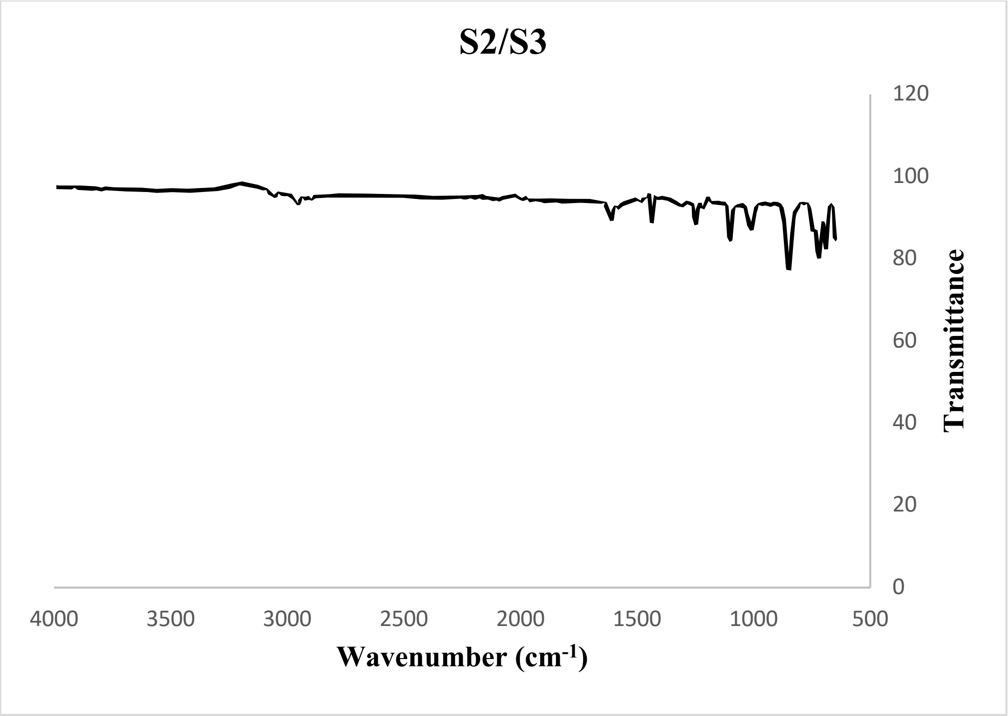

IR Spectra of S2/S3

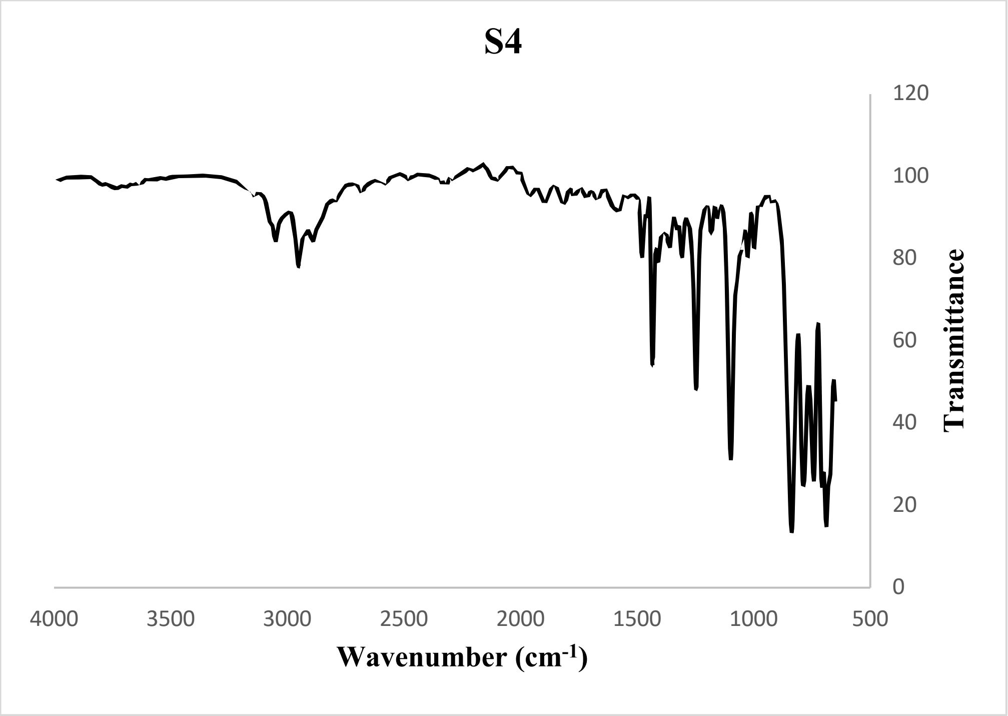

IR Spectra of S4

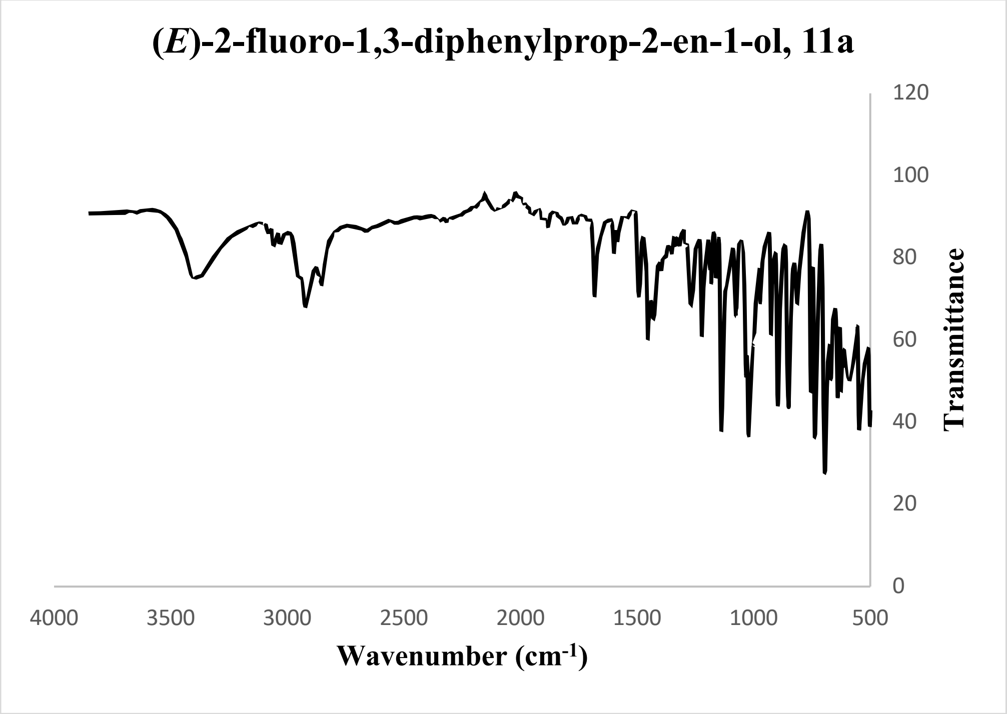

IR Spectra of (*E*)-2-fluoro-1,3-diphenylprop-2-en-1-ol, 11a

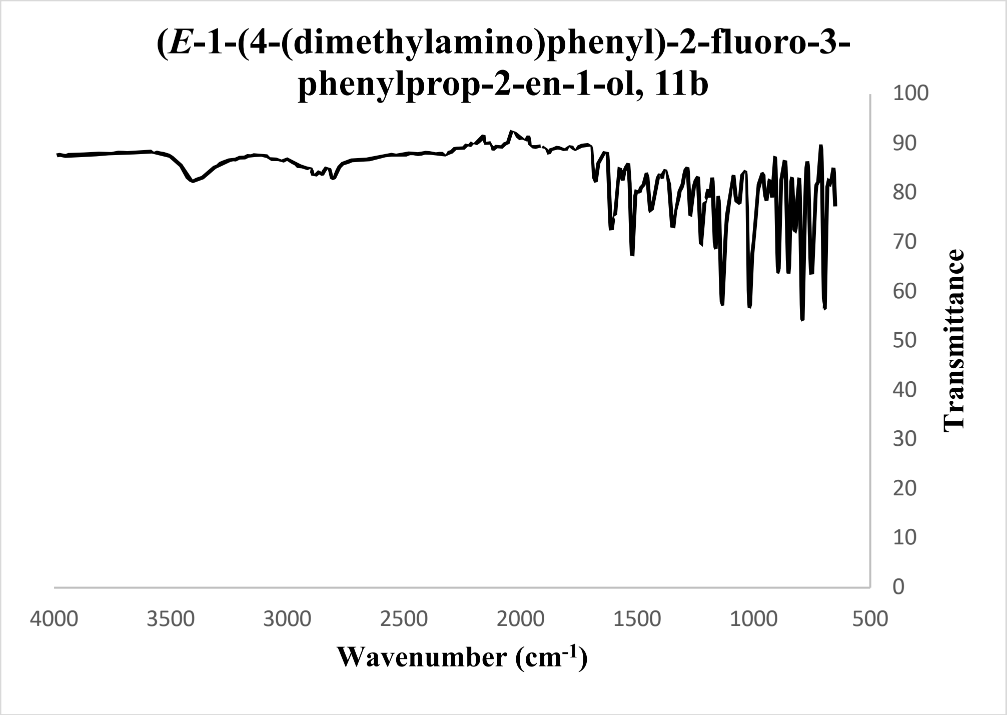

IR Spectra of (*E*-1-(4-(dimethylamino)phenyl)-2-fluoro-3-phenylprop-2-en-1-ol, 11b

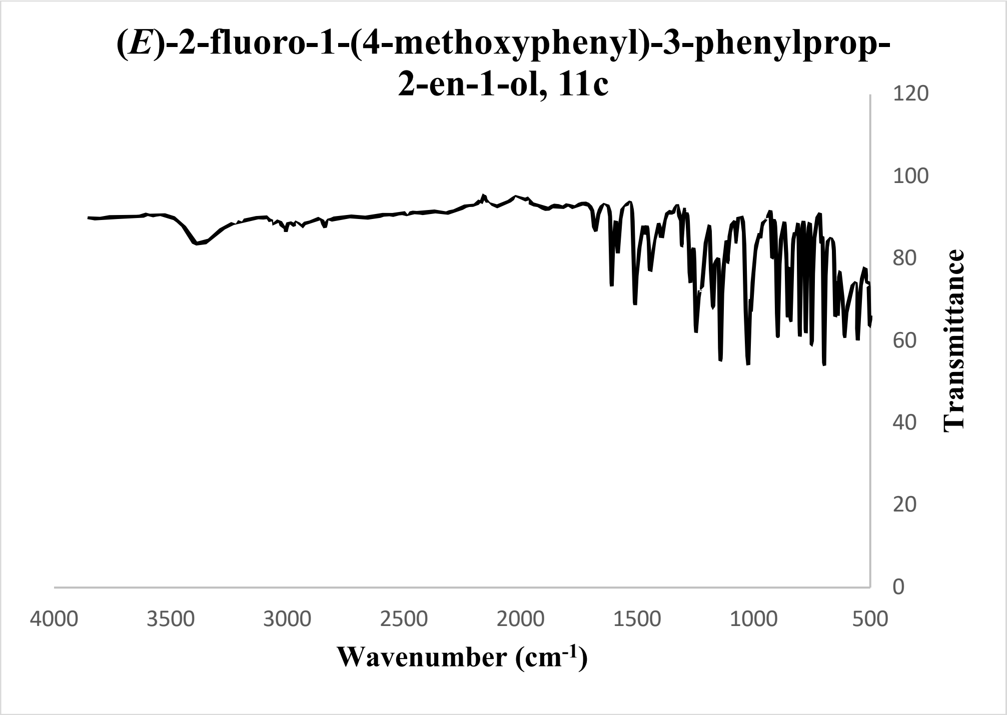

IR Spectra of (*E*)-2-fluoro-1-(4-methoxyphenyl)-3-phenylprop-2-en-1-ol, 11c

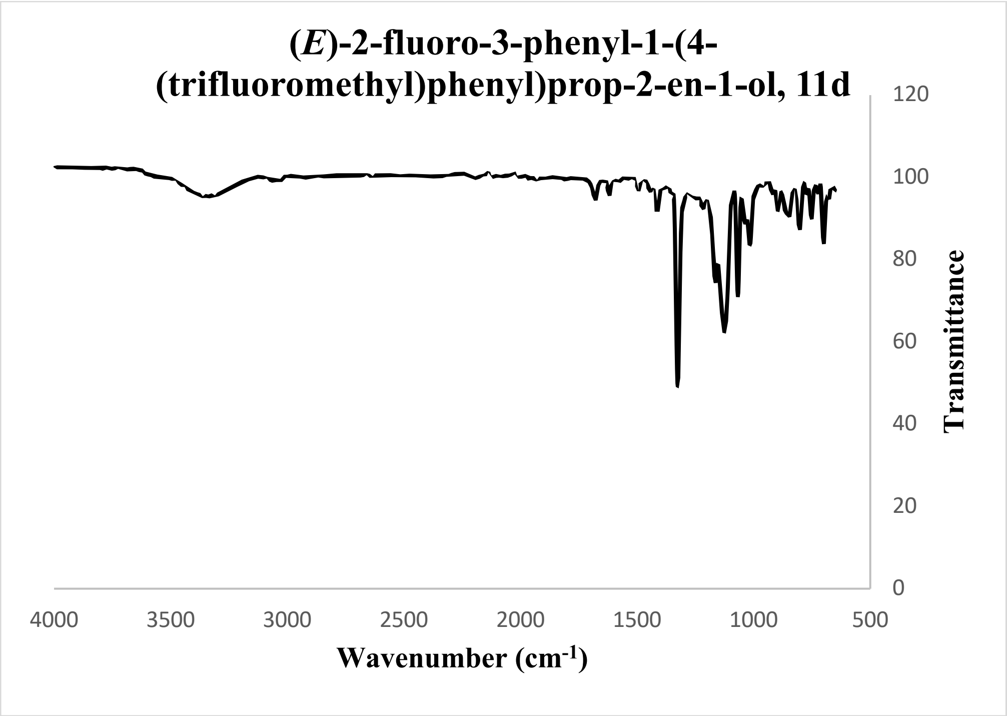

IR Spectra of (*E*)-2-fluoro-3-phenyl-1-(4-(trifluoromethyl)phenyl)prop-2-en-1-ol, 11d

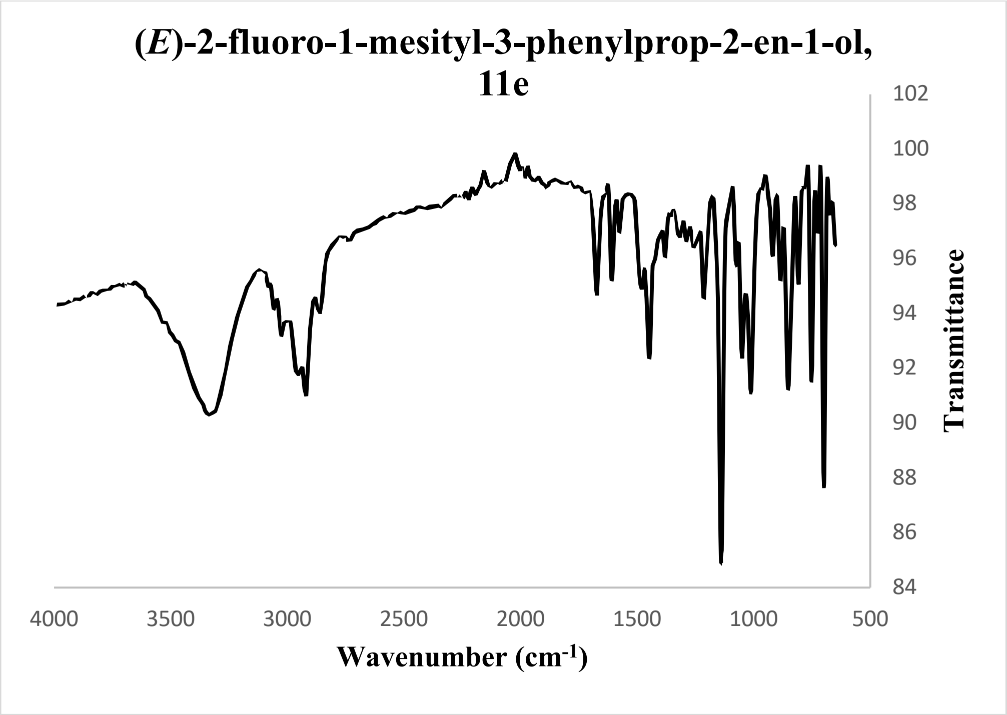

IR Spectra of (*E*)-2-fluoro-1-mesityl-3-phenylprop-2-en-1-ol, 11e

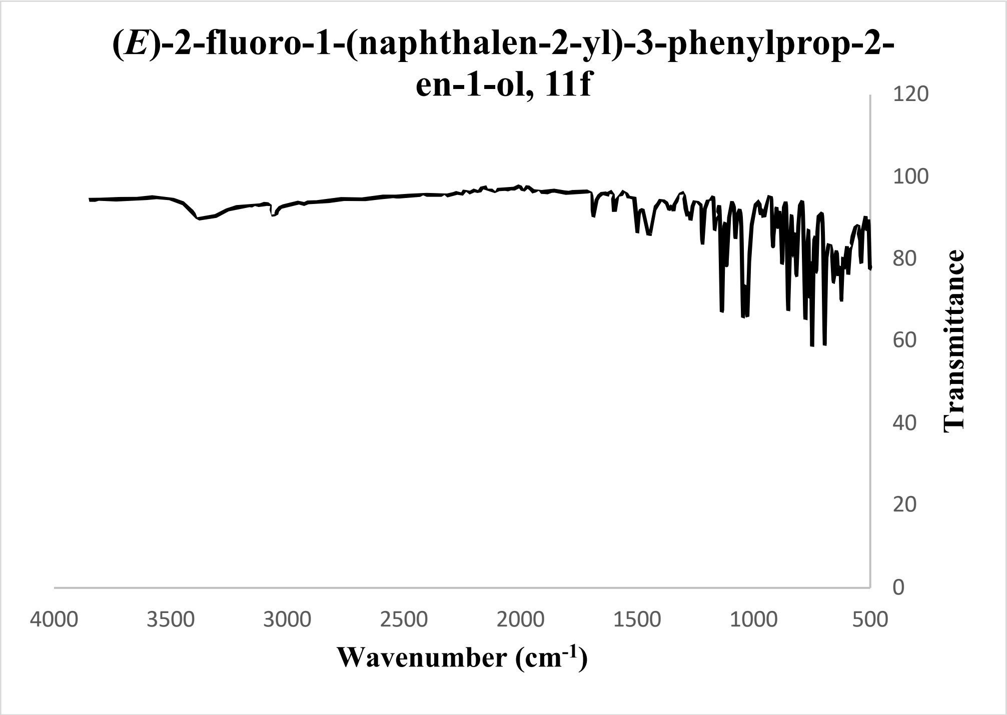

IR Spectra of (*E*)-2-fluoro-1-(naphthalen-2-yl)-3-phenylprop-2-en-1-ol, 11f

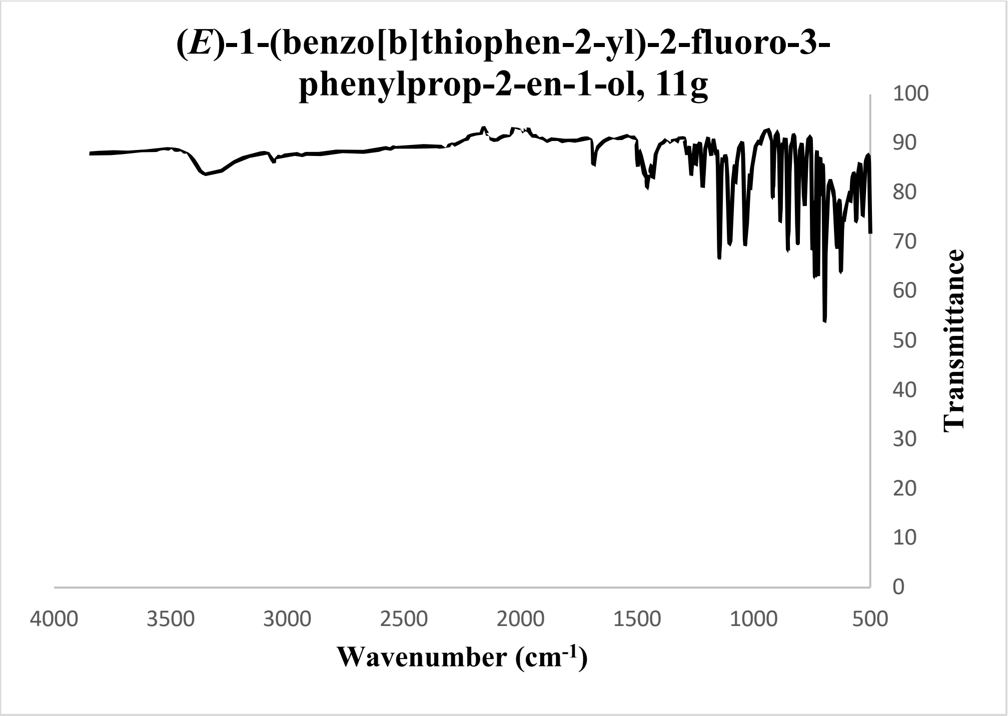

IR Spectra of (*E*)-1-(benzo[b]thiophen-2-yl)-2-fluoro-3-phenylprop-2-en-1-ol, 11g

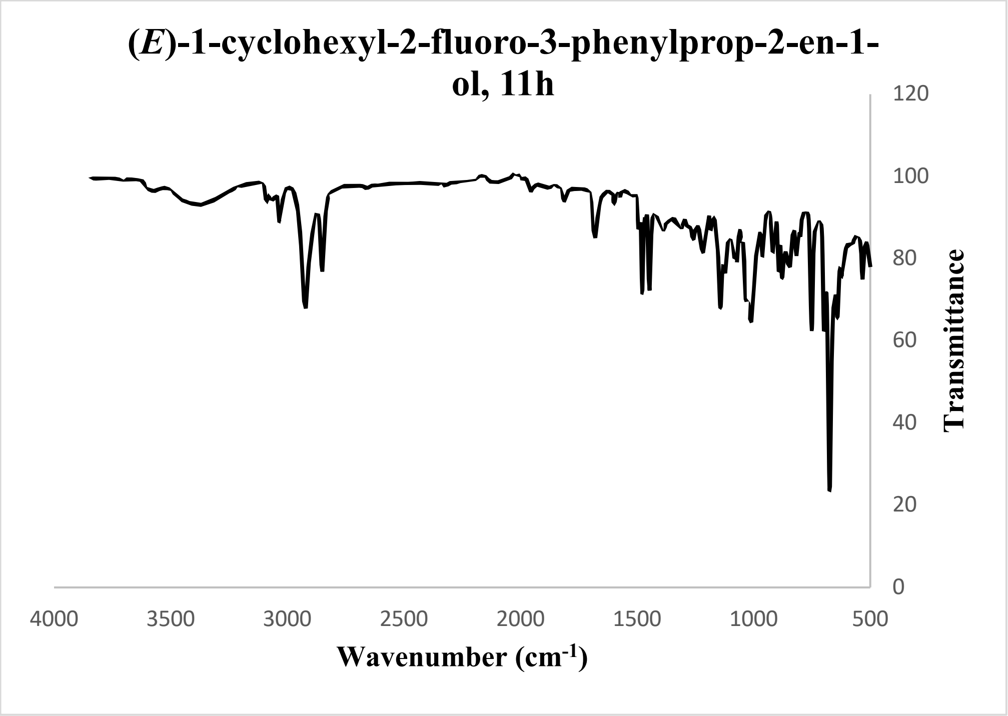

IR Spectra of (*E*)-1-cyclohexyl-2-fluoro-3-phenylprop-2-en-1-ol, 11h

14.1 Mass Spectra

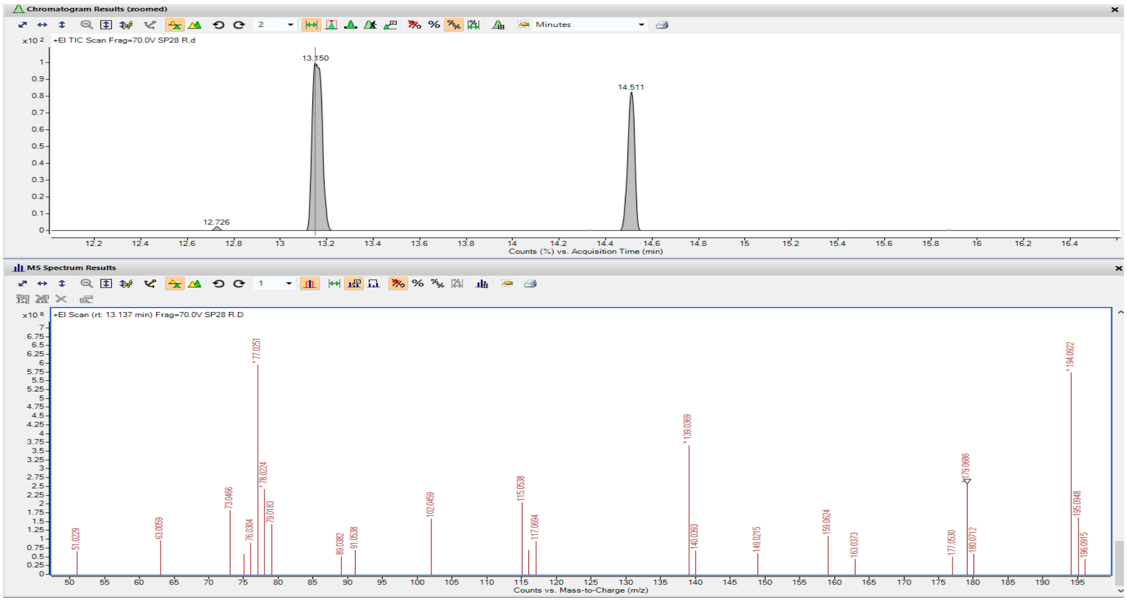

Mass Spectra of Z/E-1-fluoro-2-phenyl-1-trimethylsilylethene, *Z*/*E*-7

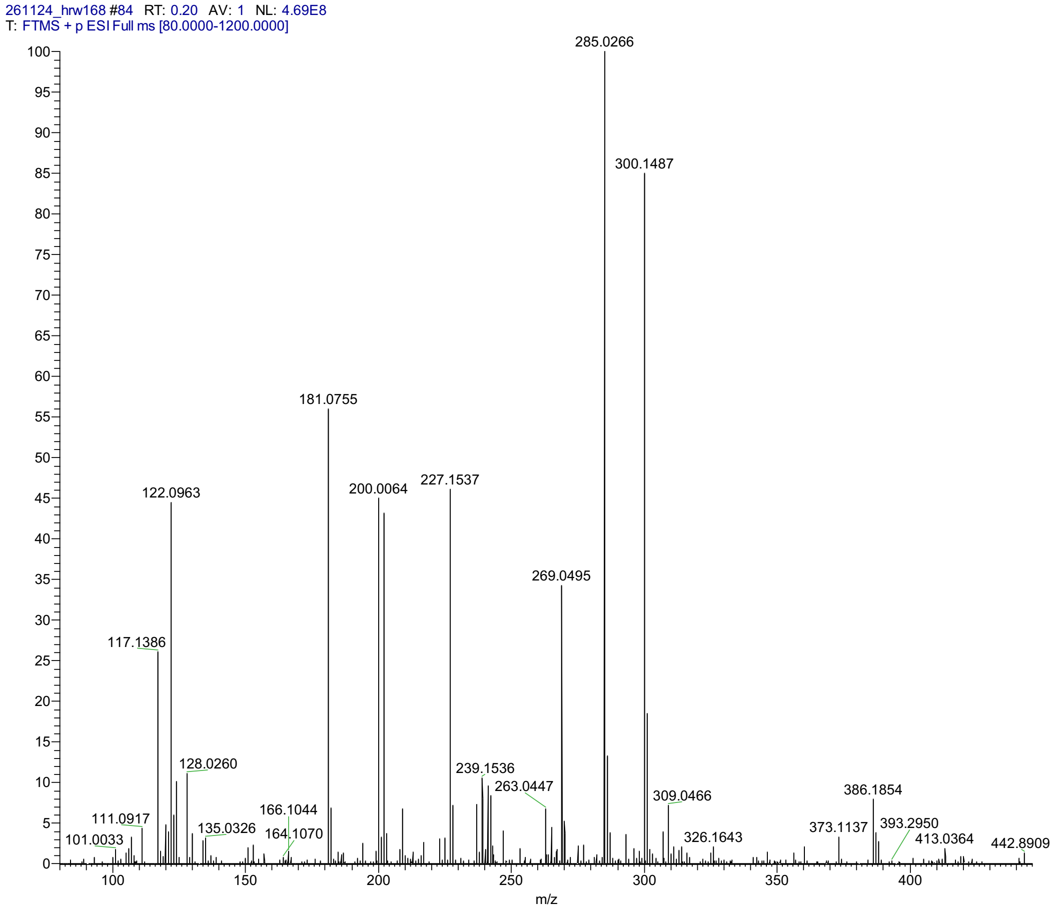

Mass Spectra of (1-fluorovinyl)diphenylphosphine sulfide, 6

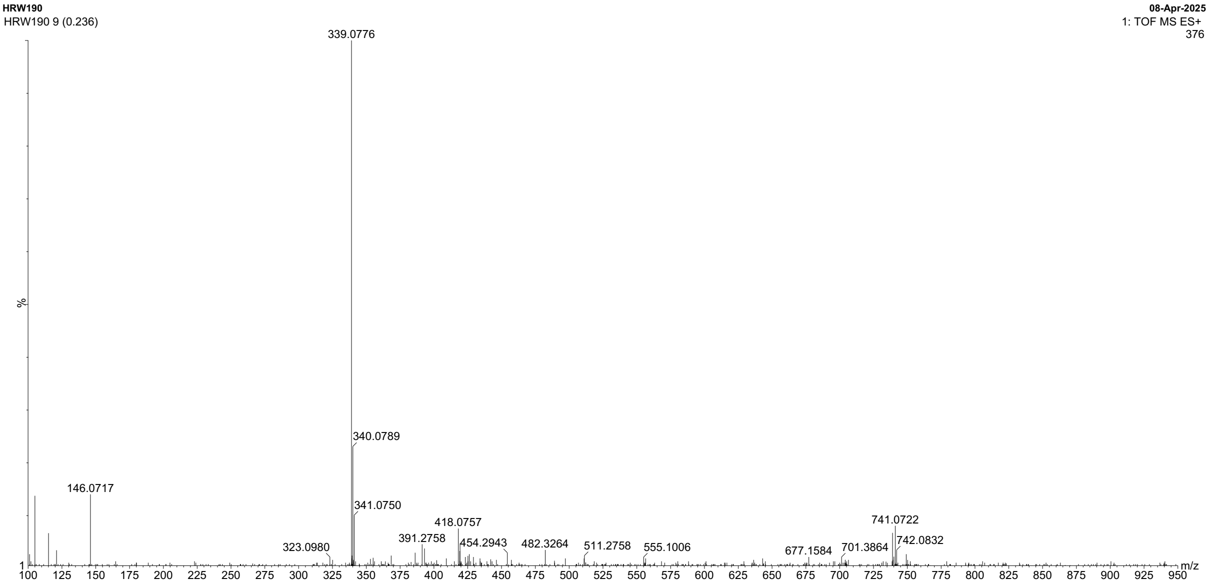

Mass Spectra of (E)-(1-fluoro-2-phenylvinyl)diphenylphosphine sulfide, 8

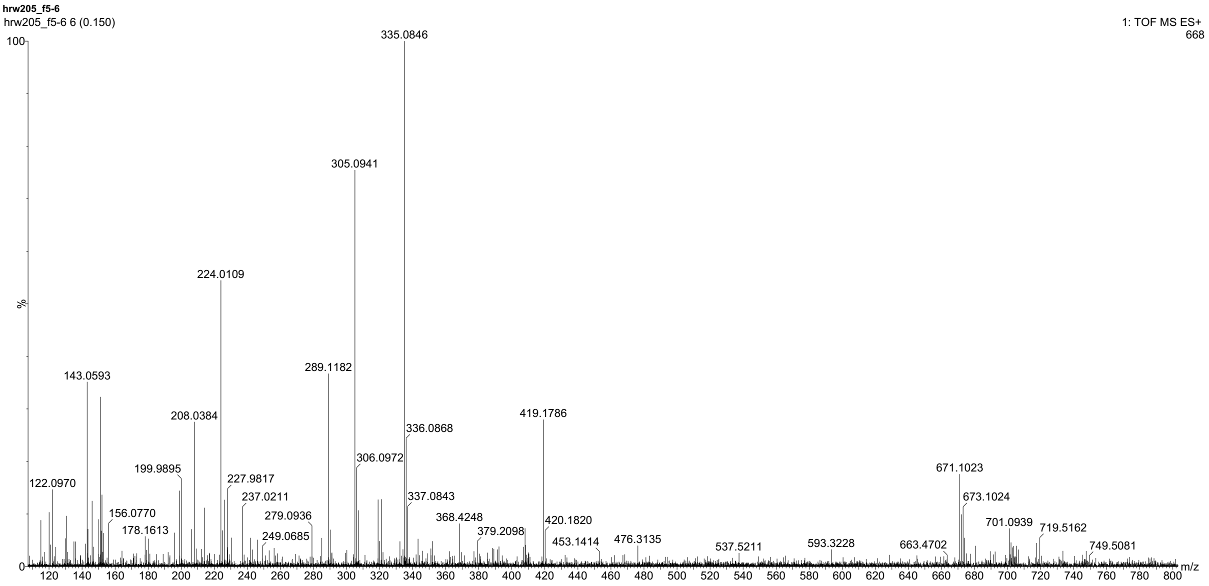

Mass Spectra of S1

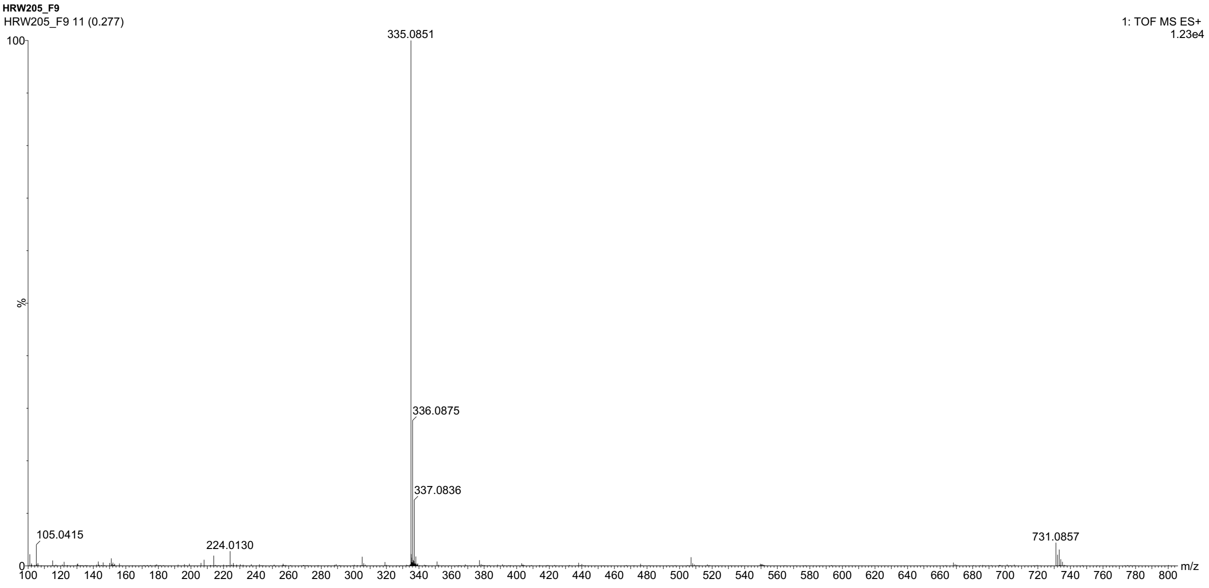

Mass Spectra of S2/S3

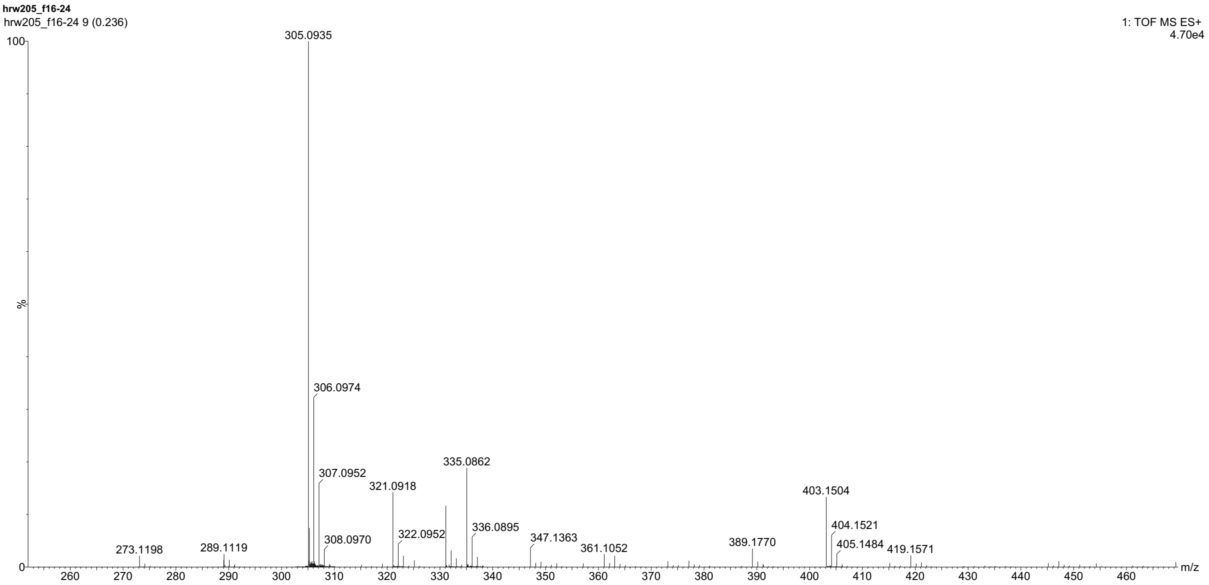

Mass Spectra of S4

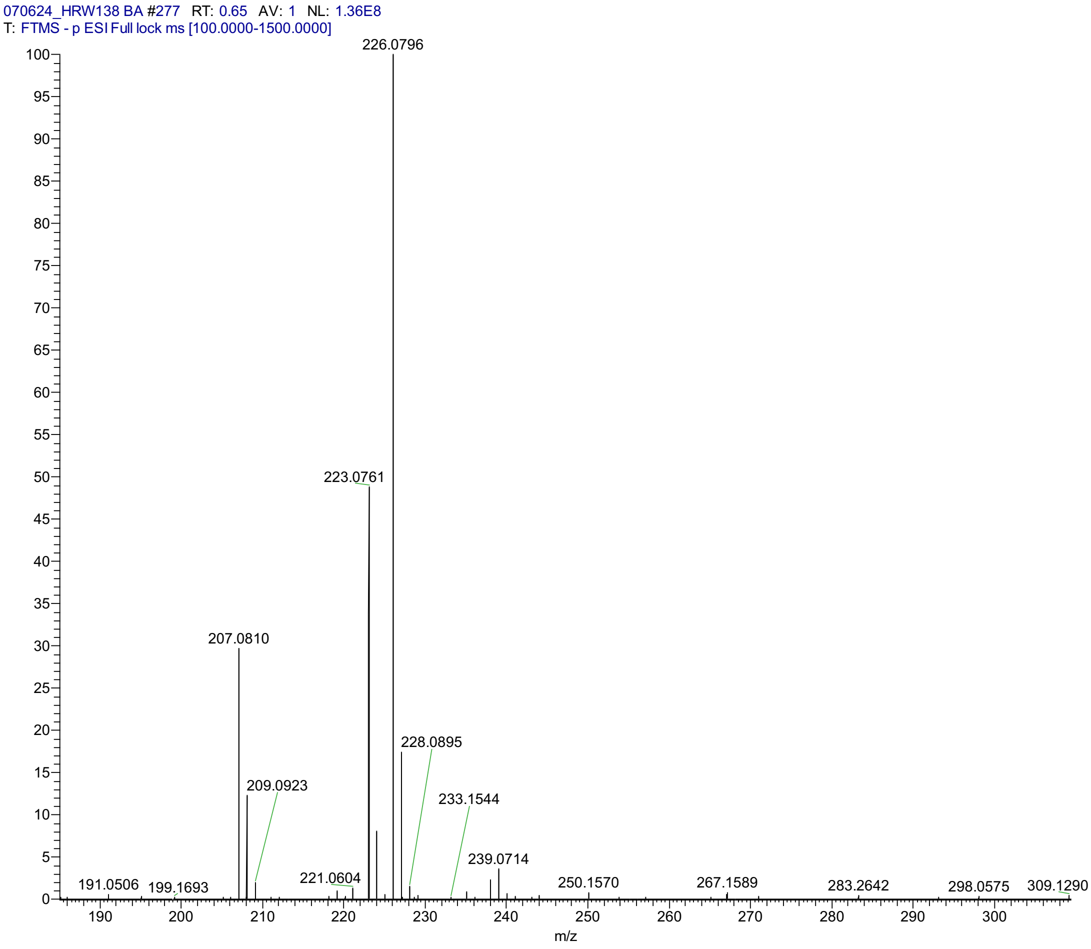

Mass Spectra of (*E*)-2-fluoro-1,3-diphenylprop-2-en-1-ol, 11a

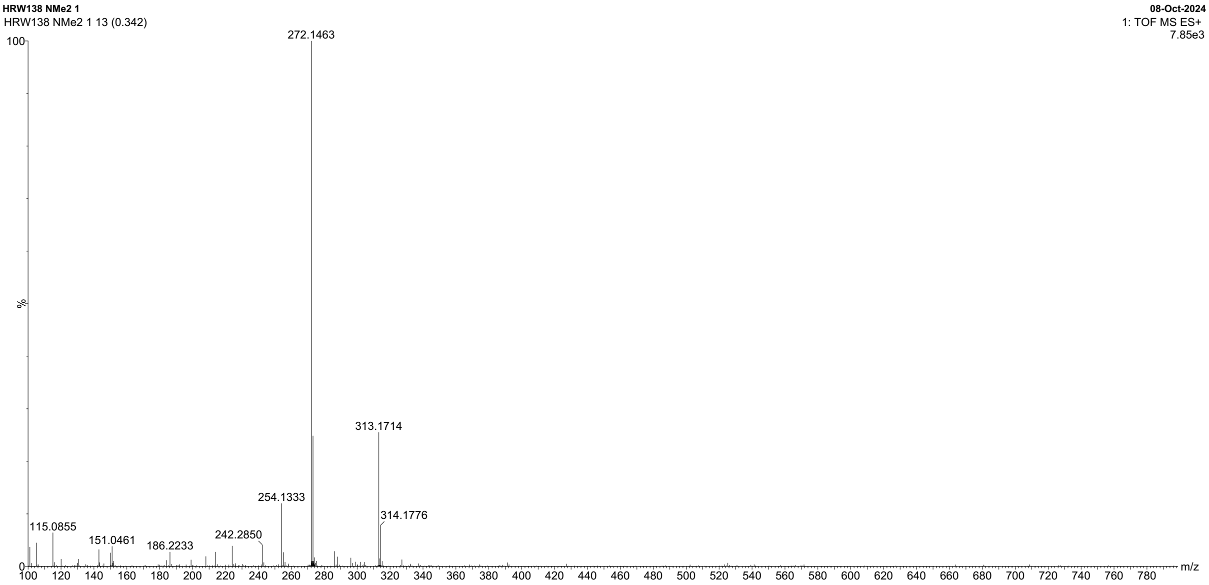

Mass Spectra of (*E*-1-(4-(dimethylamino)phenyl)-2-fluoro-3-phenylprop-2-en-1-ol, 11b

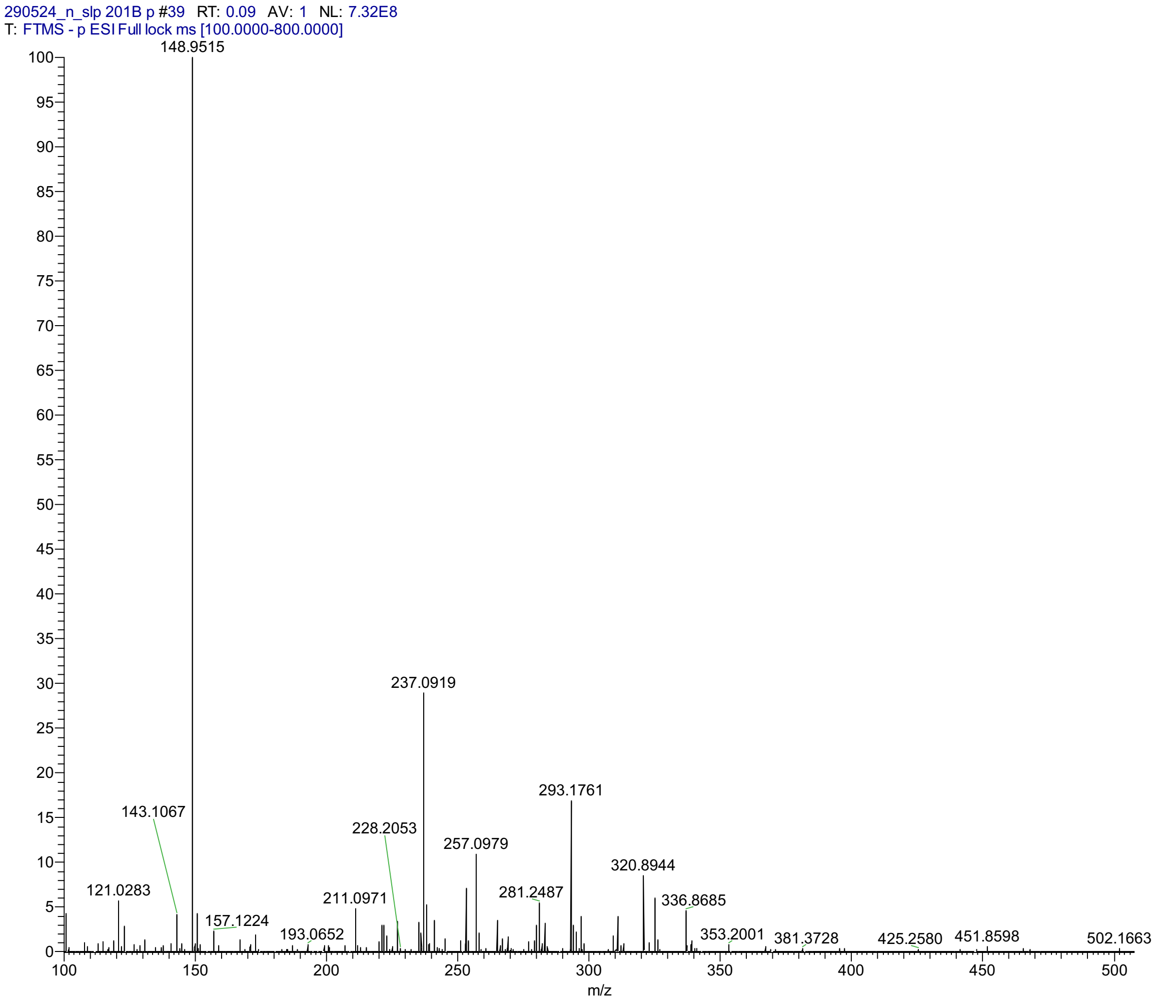

Mass Spectra of (*E*)-2-fluoro-1-(4-methoxyphenyl)-3-phenylprop-2-en-1-ol, 11c

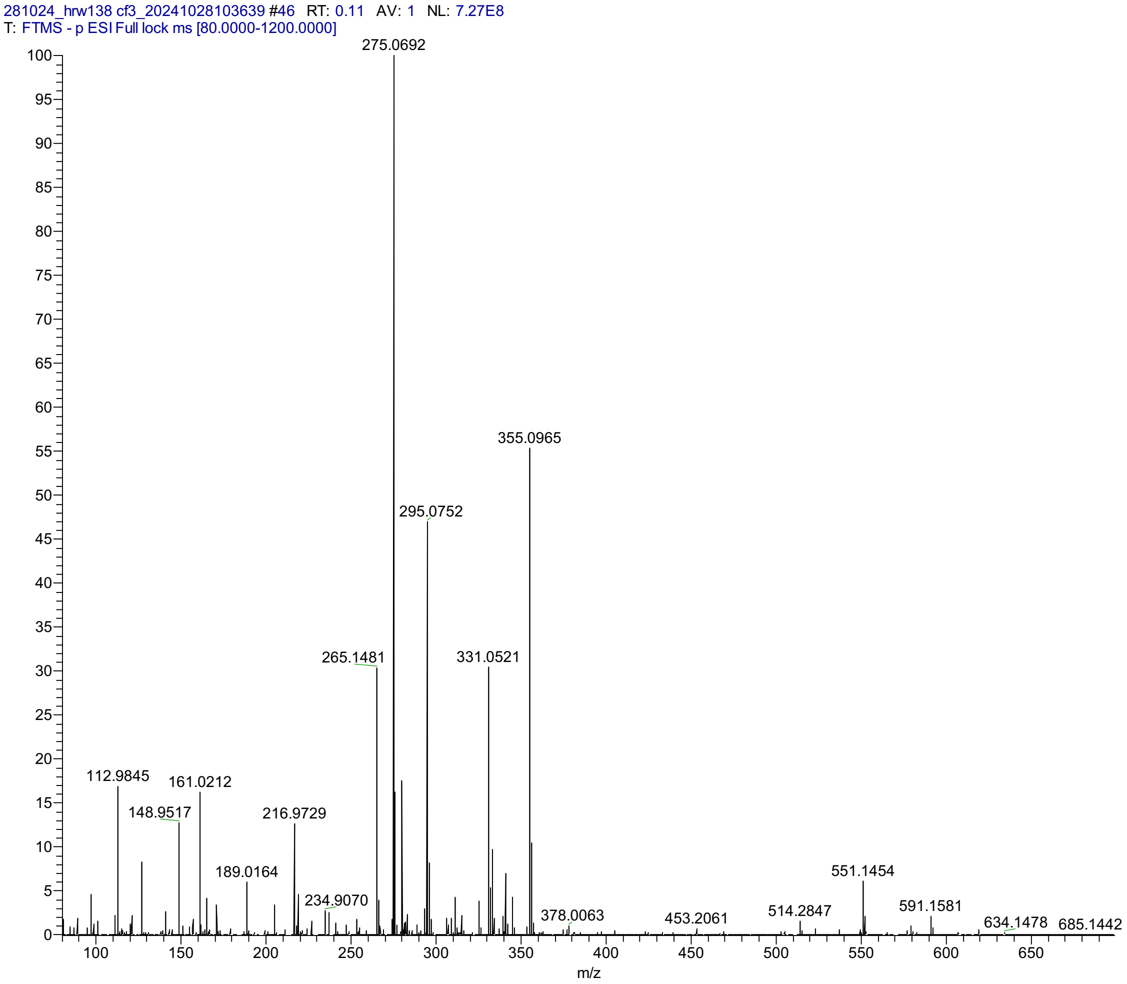

Mass Spectra of (*E*)-2-fluoro-3-phenyl-1-(4-(trifluoromethyl)phenyl)prop-2-en-1-ol, 11d

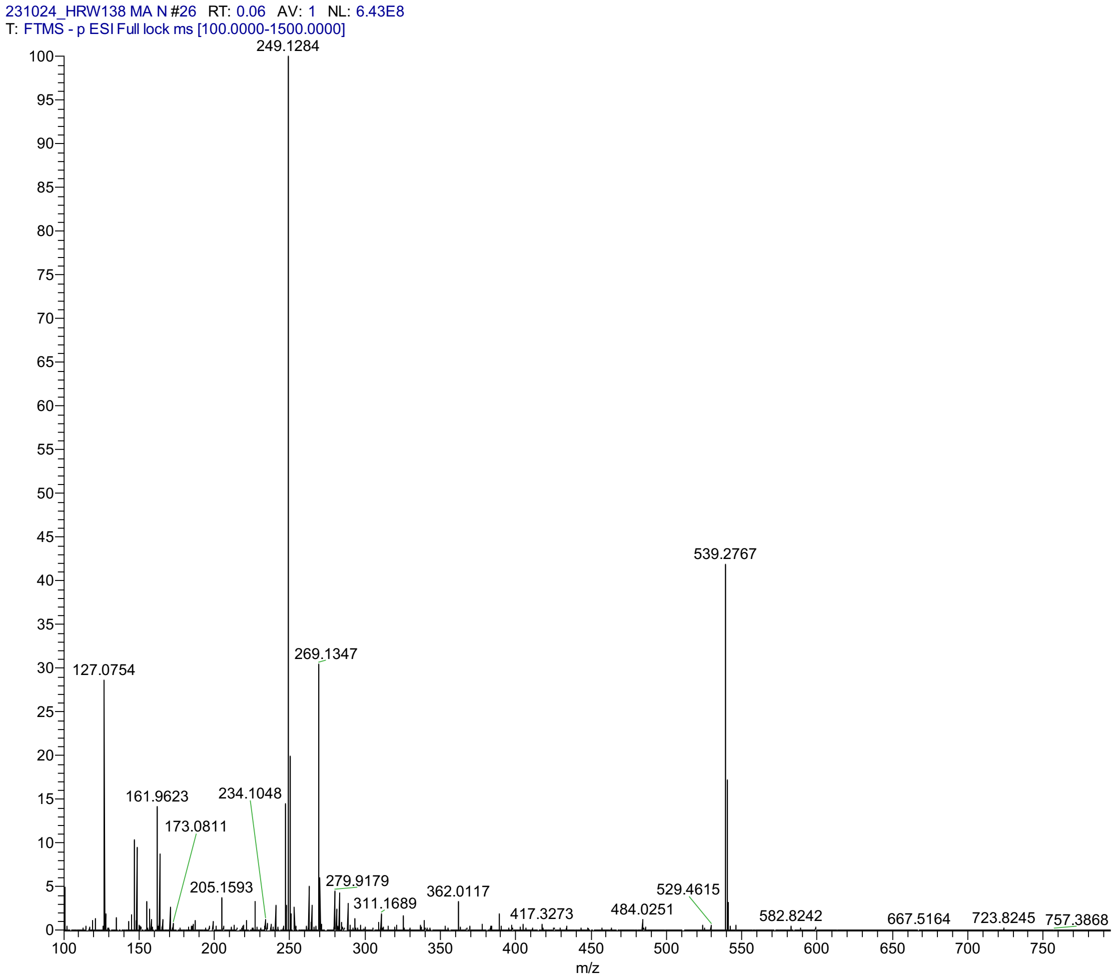

Mass Spectra of (*E*)-2-fluoro-1-mesityl-3-phenylprop-2-en-1-ol, 11e

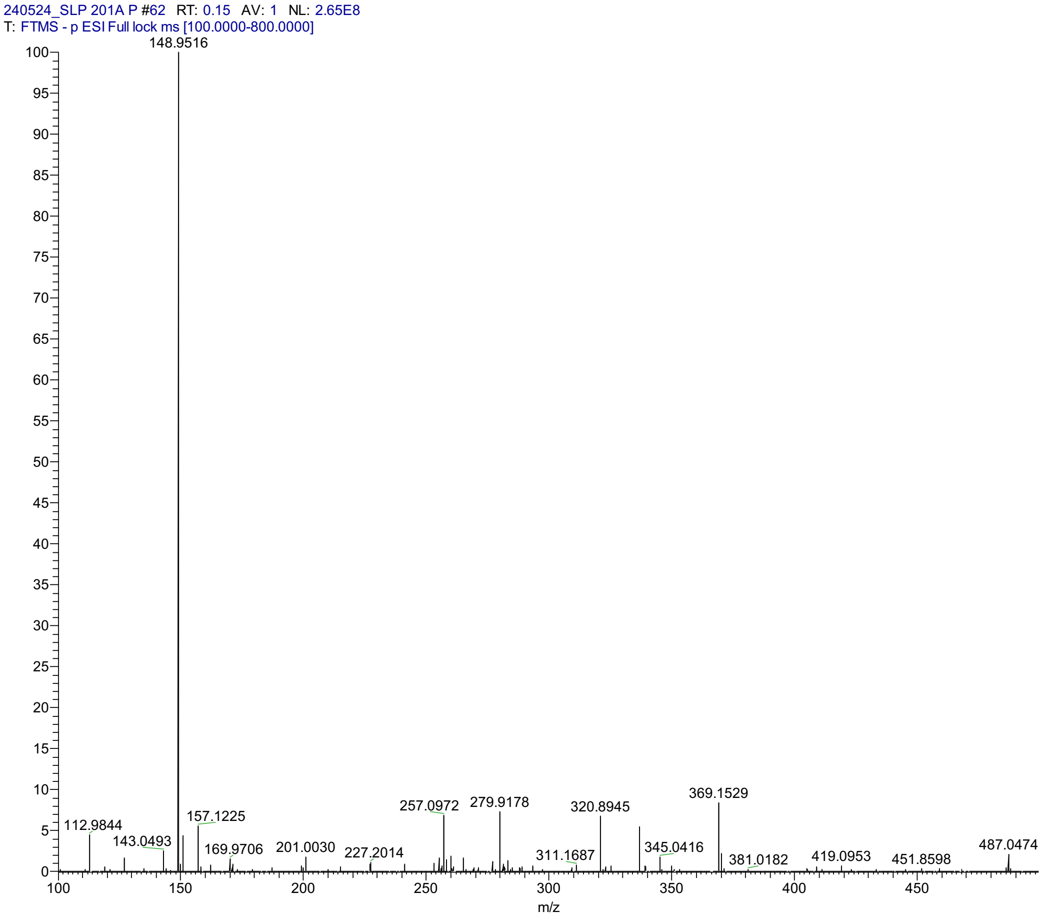

Mass Spectra of (*E*)-2-fluoro-1-(naphthalen-2-yl)-3-phenylprop-2-en-1-ol, 11f

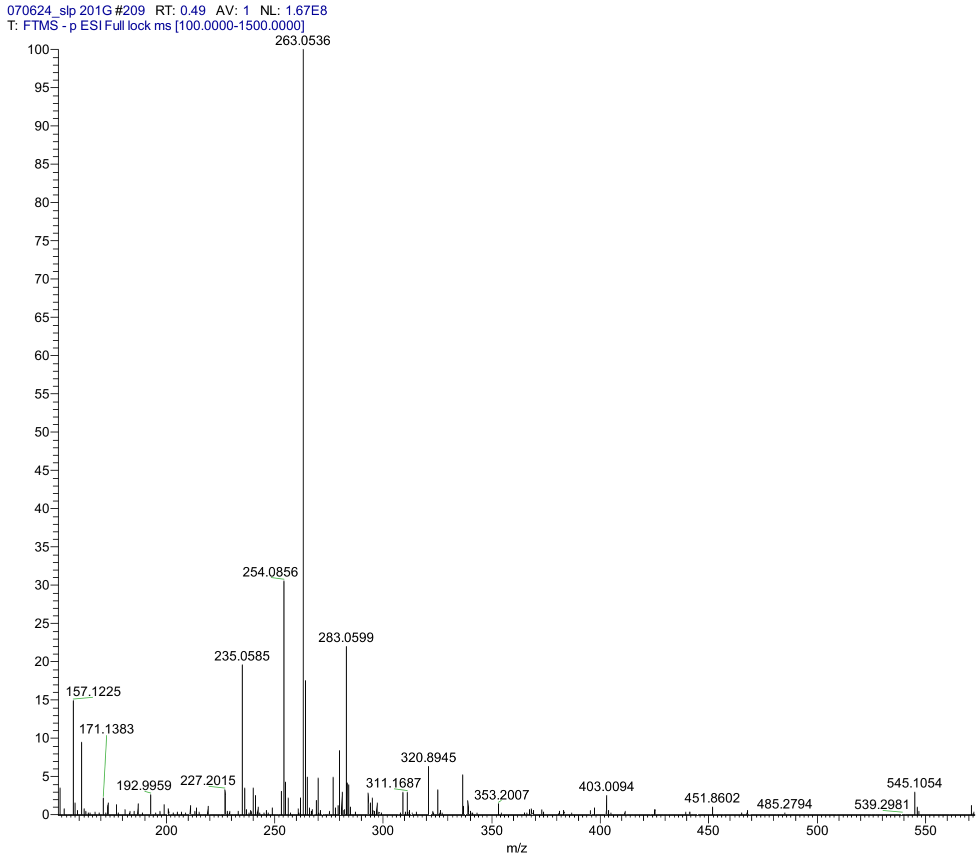

Mass Spectra of (*E*)-1-(benzo[b]thiophen-2-yl)-2-fluoro-3-phenylprop-2-en-1-ol, 11g

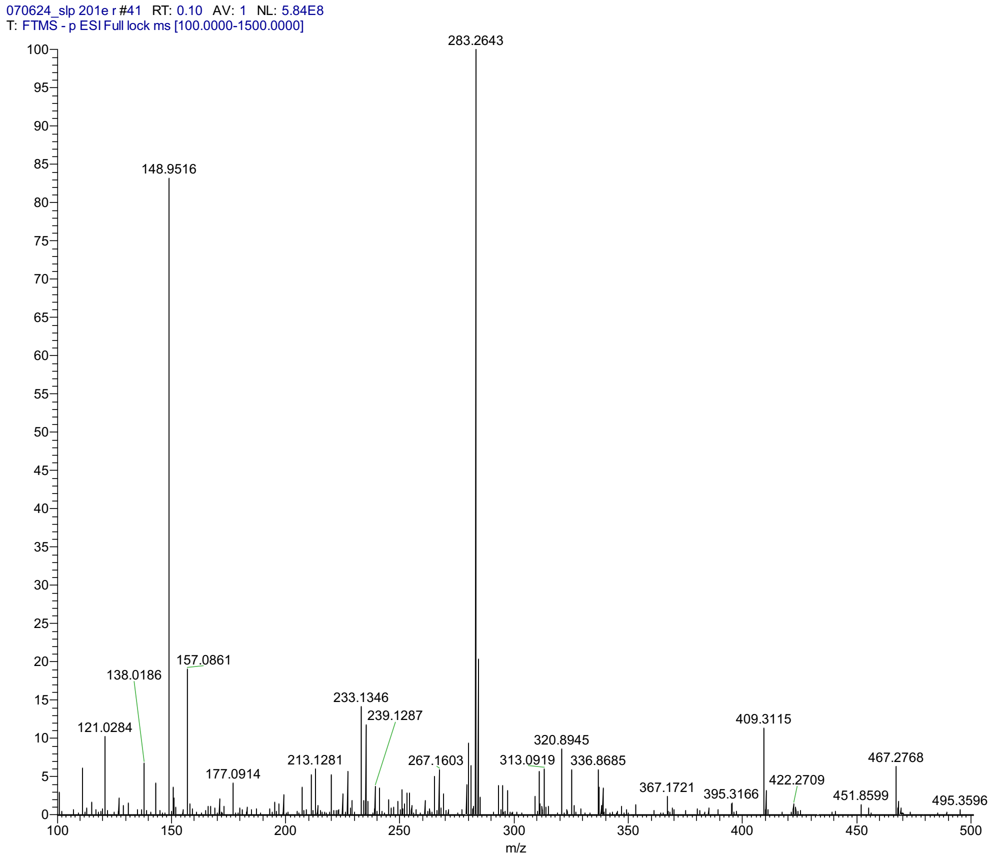

Mass Spectra of (*E*)-1-cyclohexyl-2-fluoro-3-phenylprop-2-en-1-ol, 11h

15.1 NMR Spectra

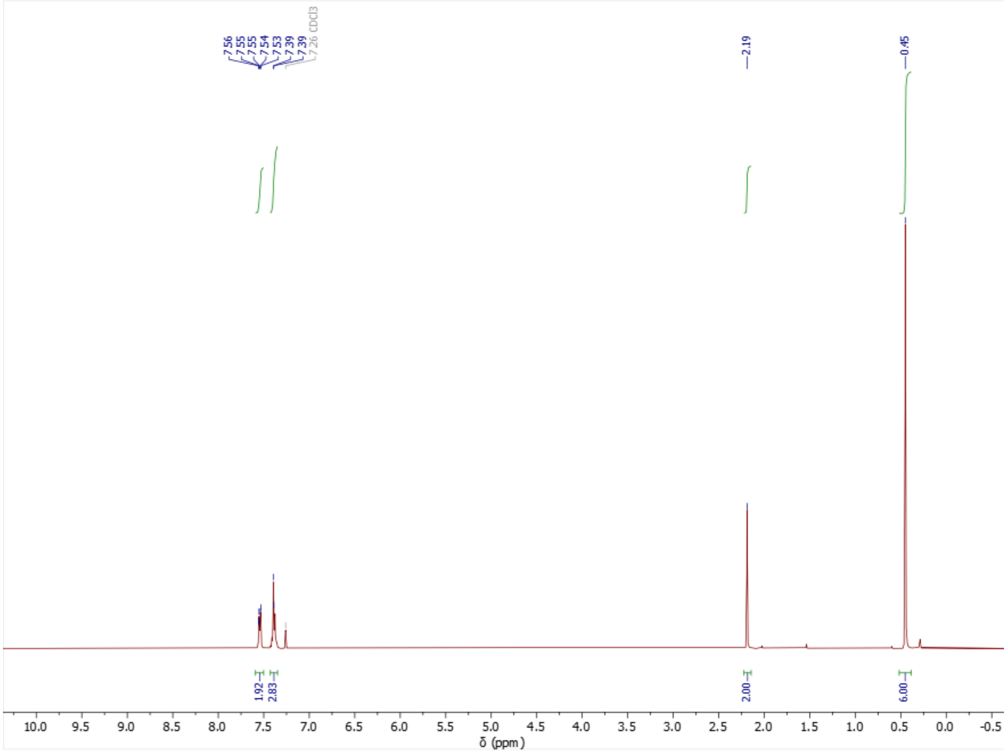

^{1}H NMR spectra of (iodomethyl)dimethylphenylsilane

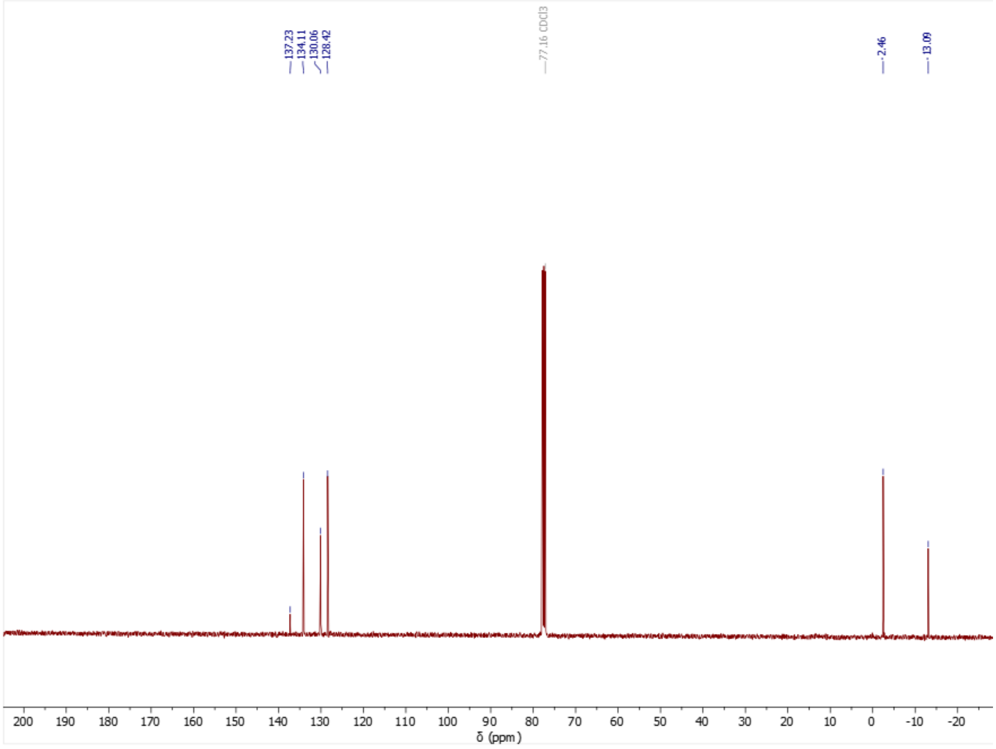

^{13}C NMR spectra of (iodomethyl)dimethylphenylsilane

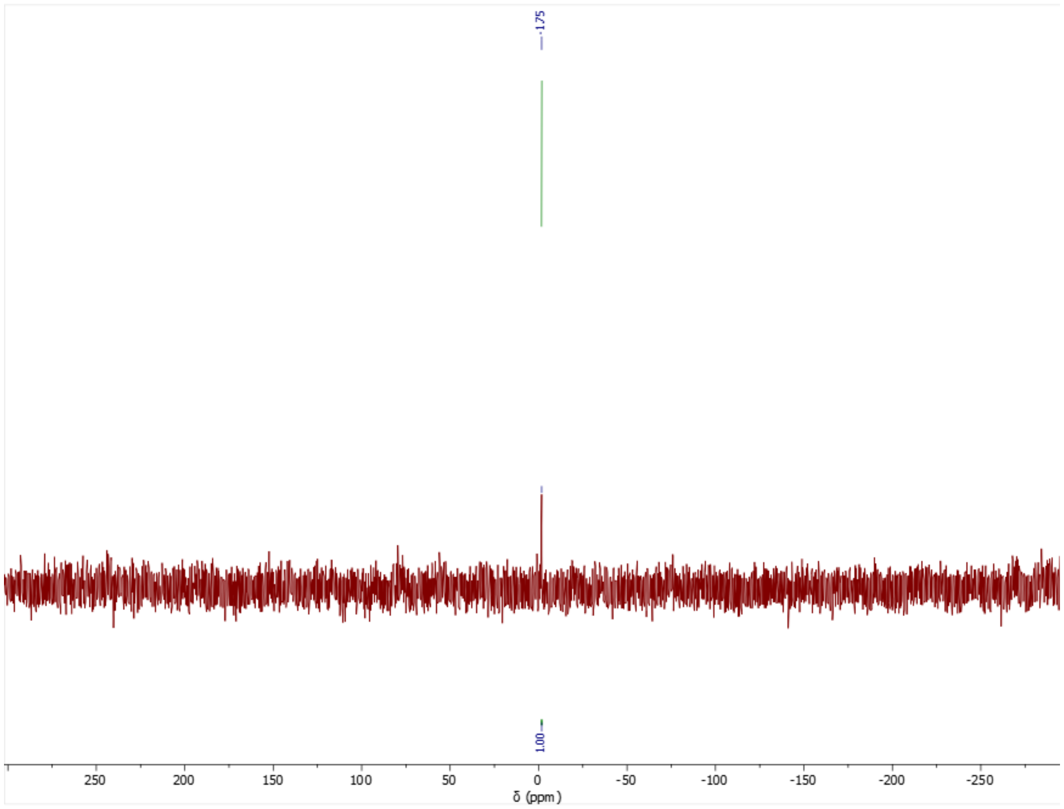

^{29}Si NMR spectra of (iodomethyl)dimethylphenylsilane

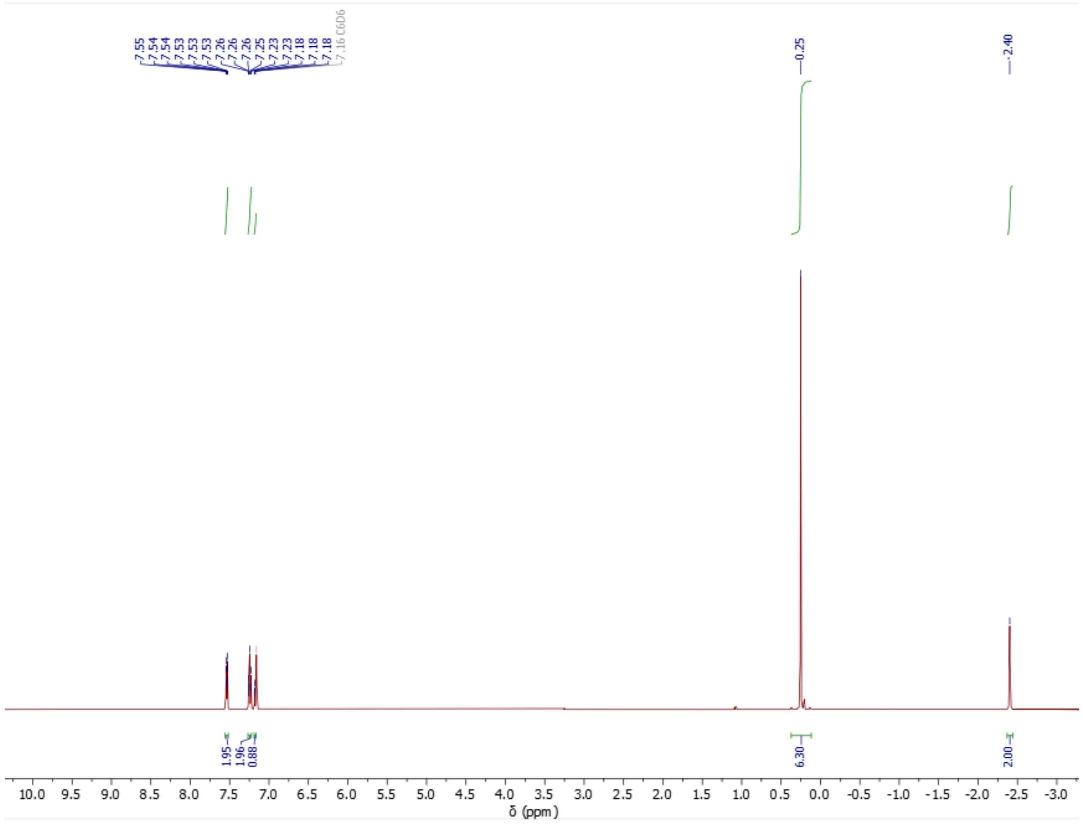

^{1}H NMR spectra of (dimethylphenylsilyl)methyllithium **1**

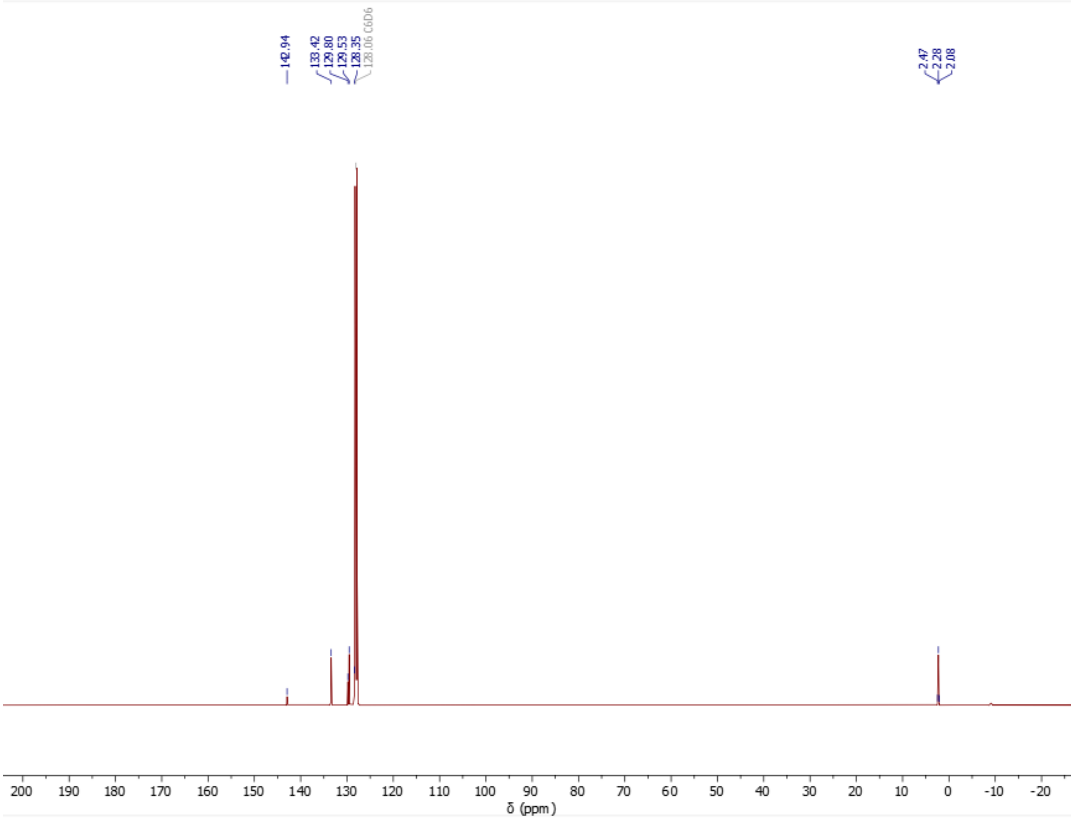

^{13}C NMR spectra of (dimethylphenylsilyl)methyllithium **1**

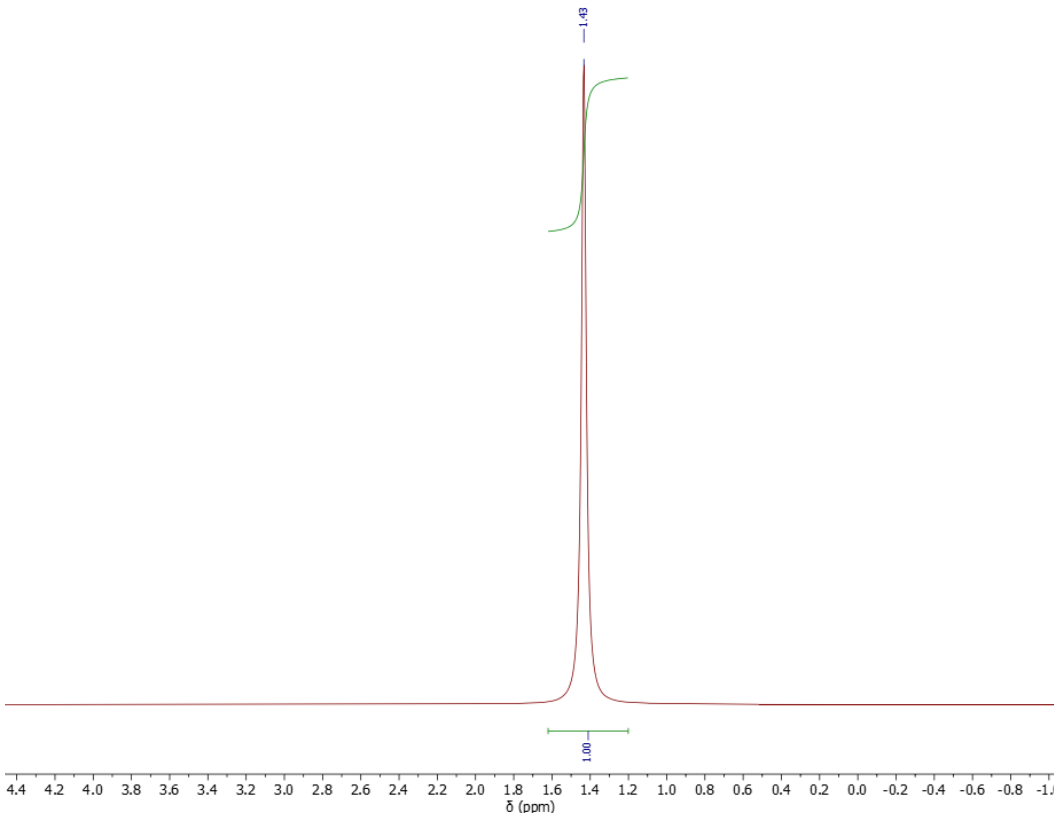

^{7}Li NMR spectra of (dimethylphenylsilyl)methyllithium **1**

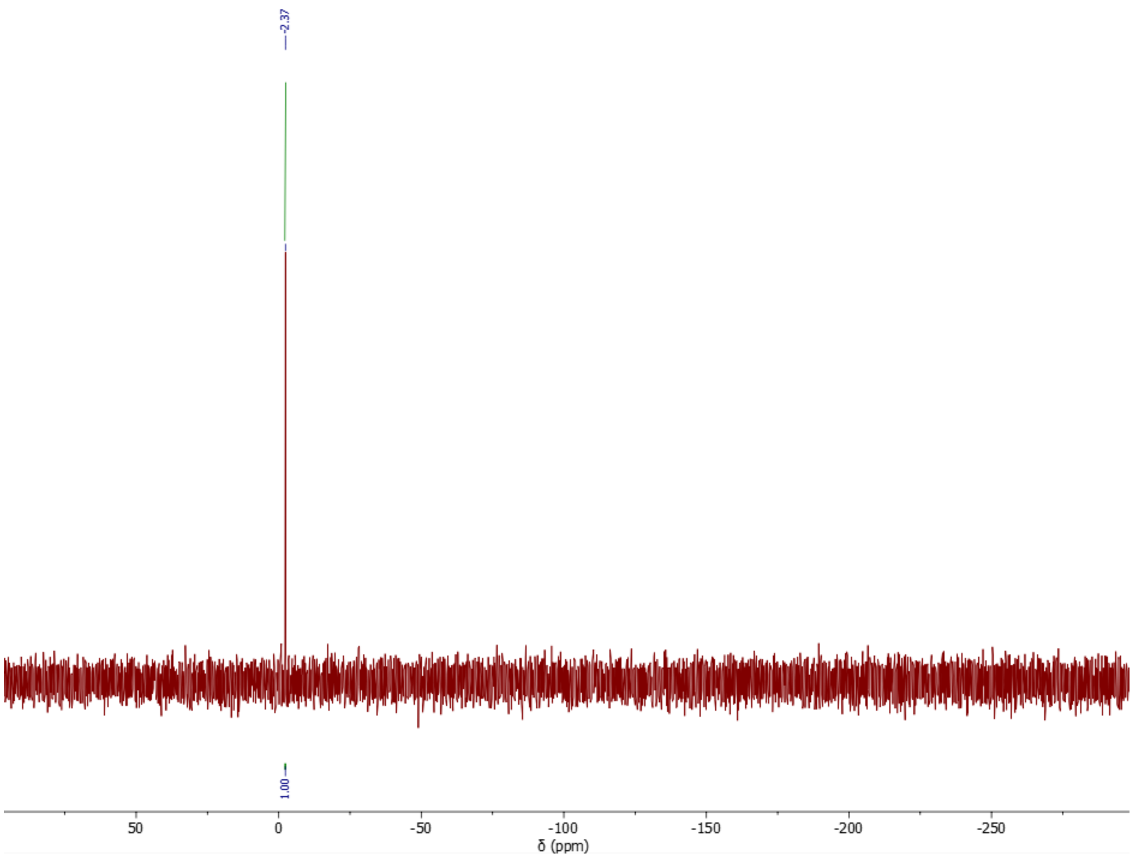

^{29}Si NMR spectra of (dimethylphenylsilyl)methyllithium **1**

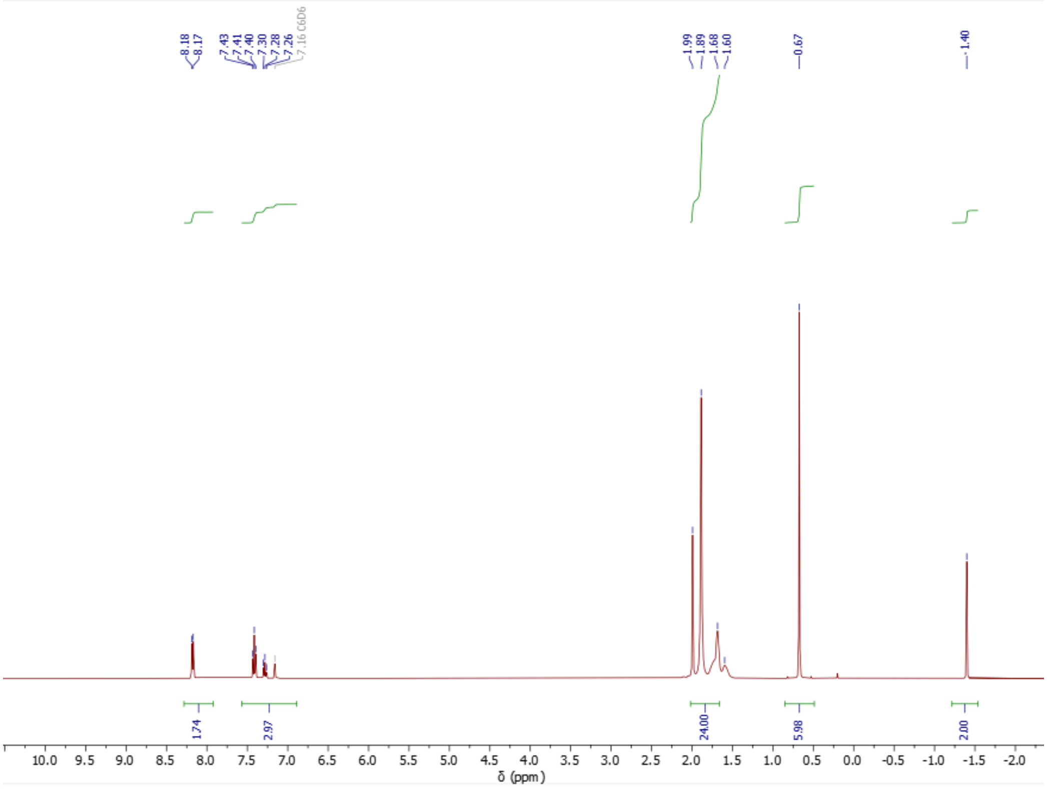

^{1}H NMR spectra of (dimethylphenylsilyl)methyllithium **1·PMDETA** complex

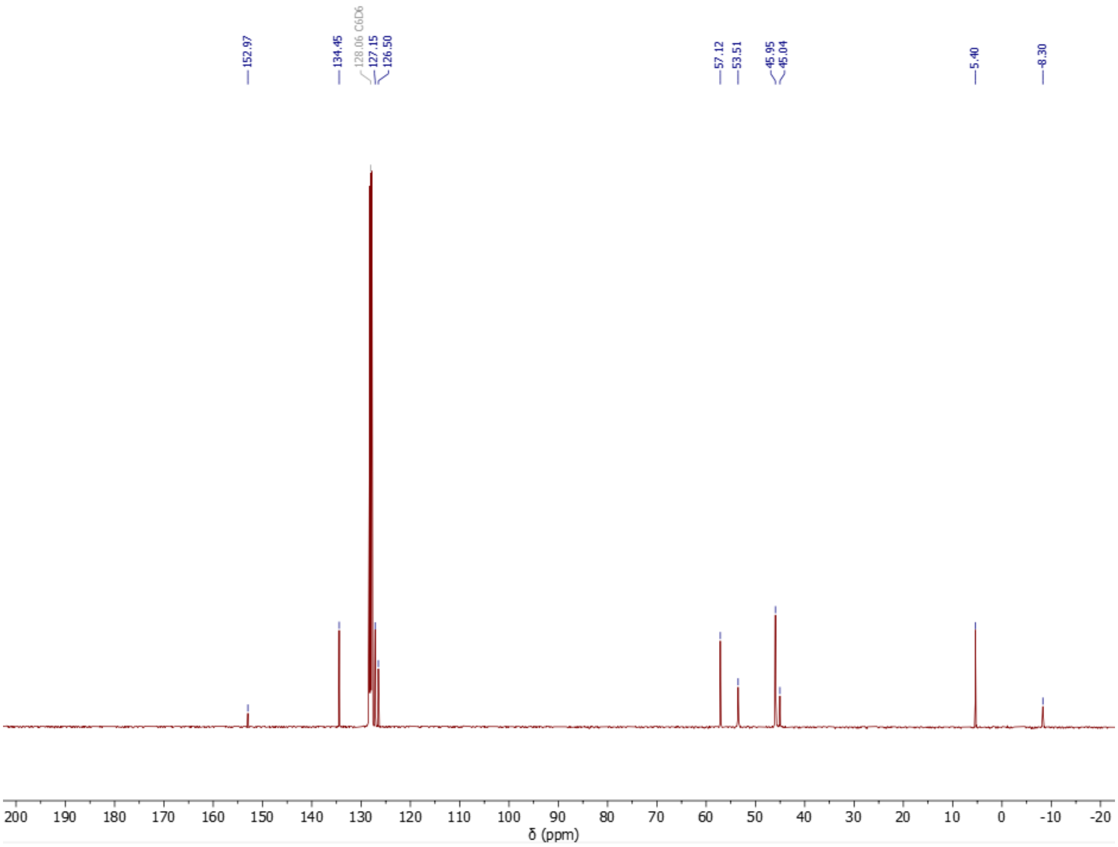

^{13}C NMR spectra of (dimethylphenylsilyl)methyllithium **1·PMDETA** complex

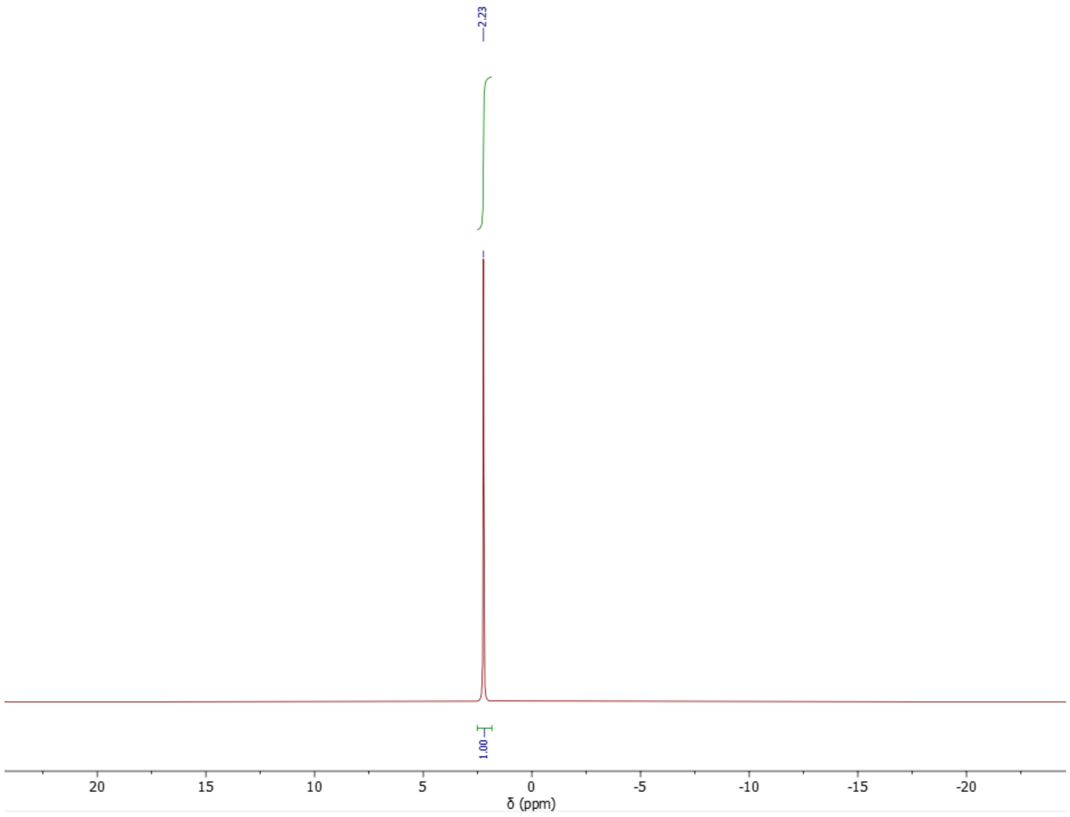

^{7}Li NMR spectra of (dimethylphenylsilyl)methyllithium **1·PMDETA** complex

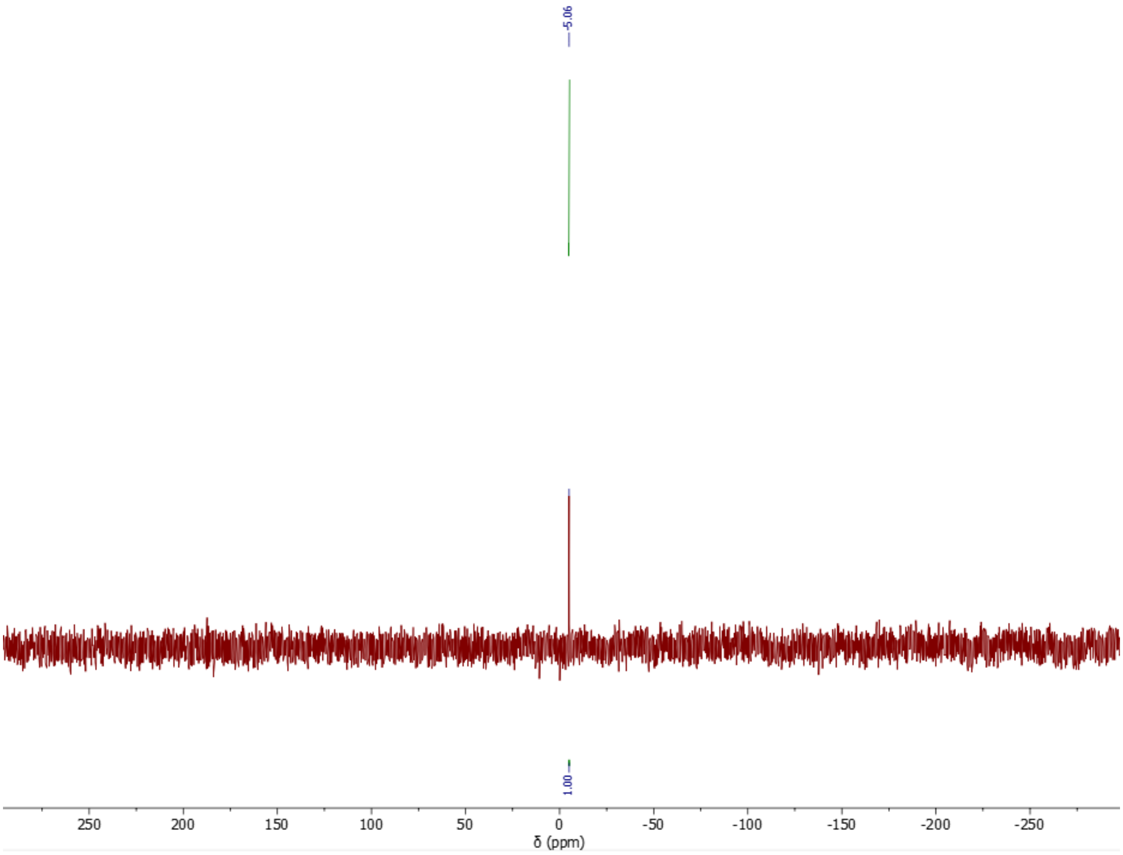

^{29}Si NMR spectra of (dimethylphenylsilyl)methyllithium **1·PMDETA** complex

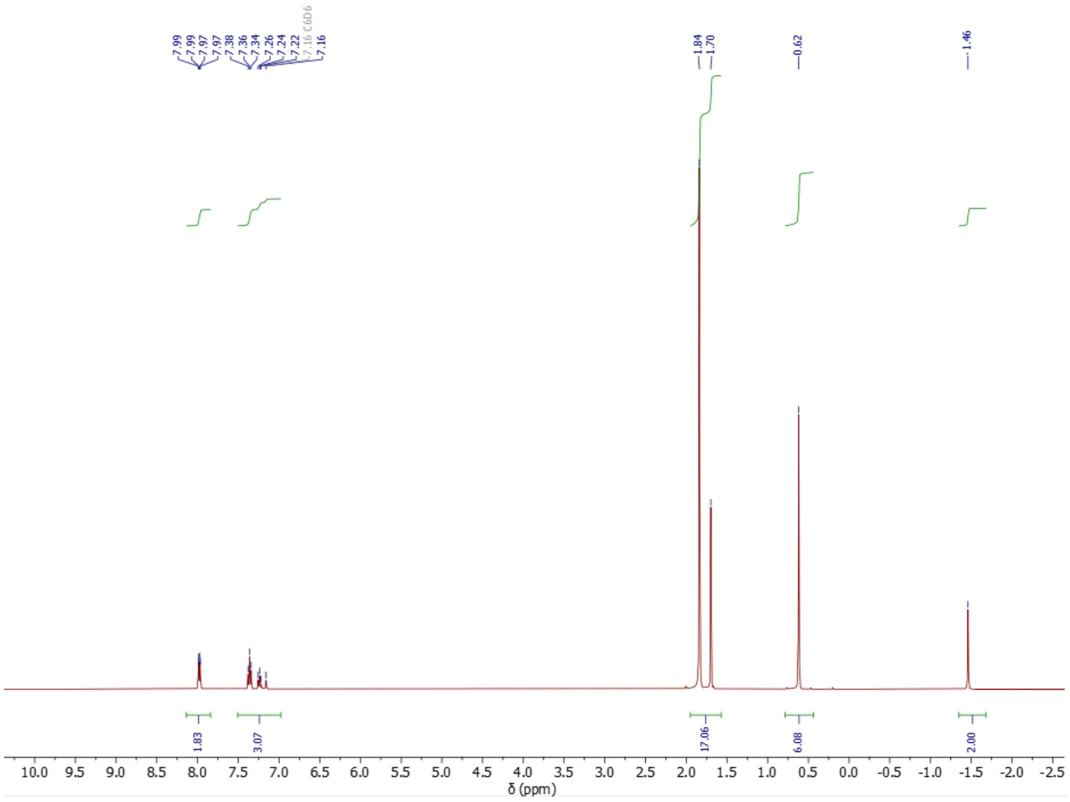

^{1}H NMR spectra of (dimethylphenylsilyl)methyllithium **1·TMEDA** complex

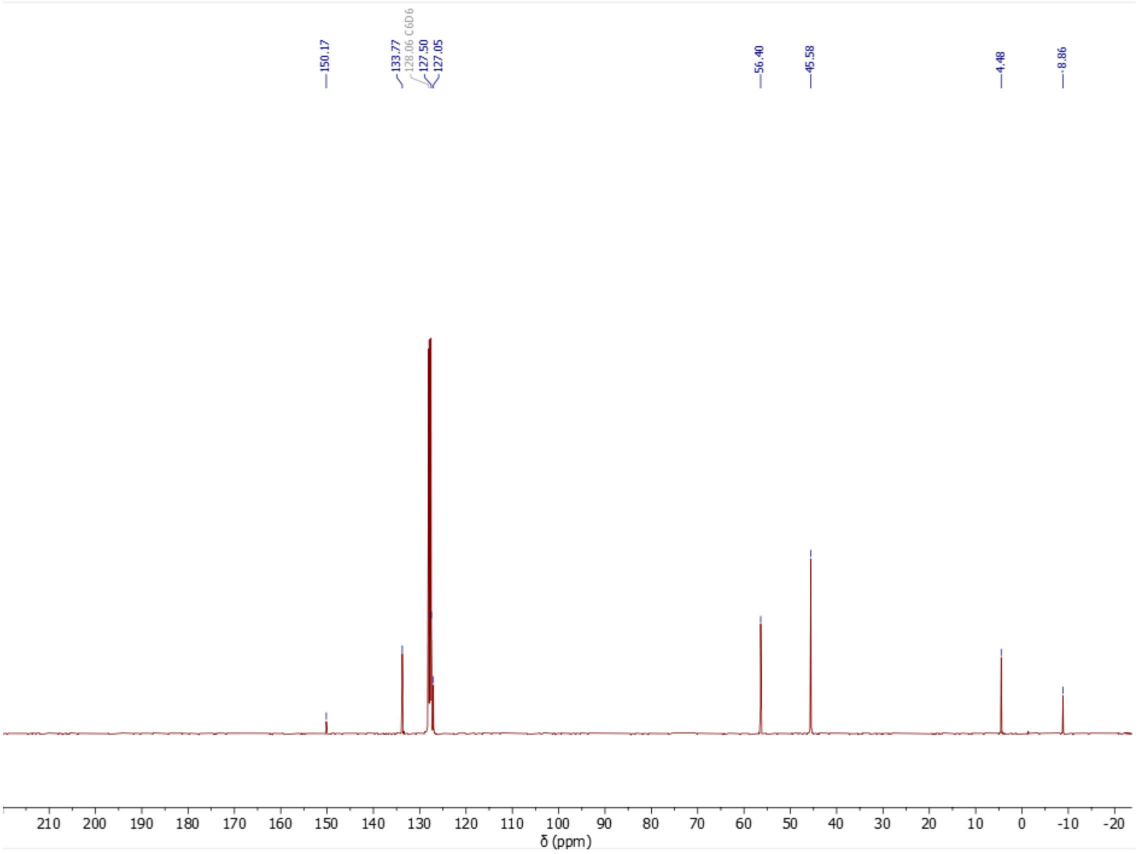

^{13}C NMR spectra of (dimethylphenylsilyl)methyllithium **1·TMEDA** complex

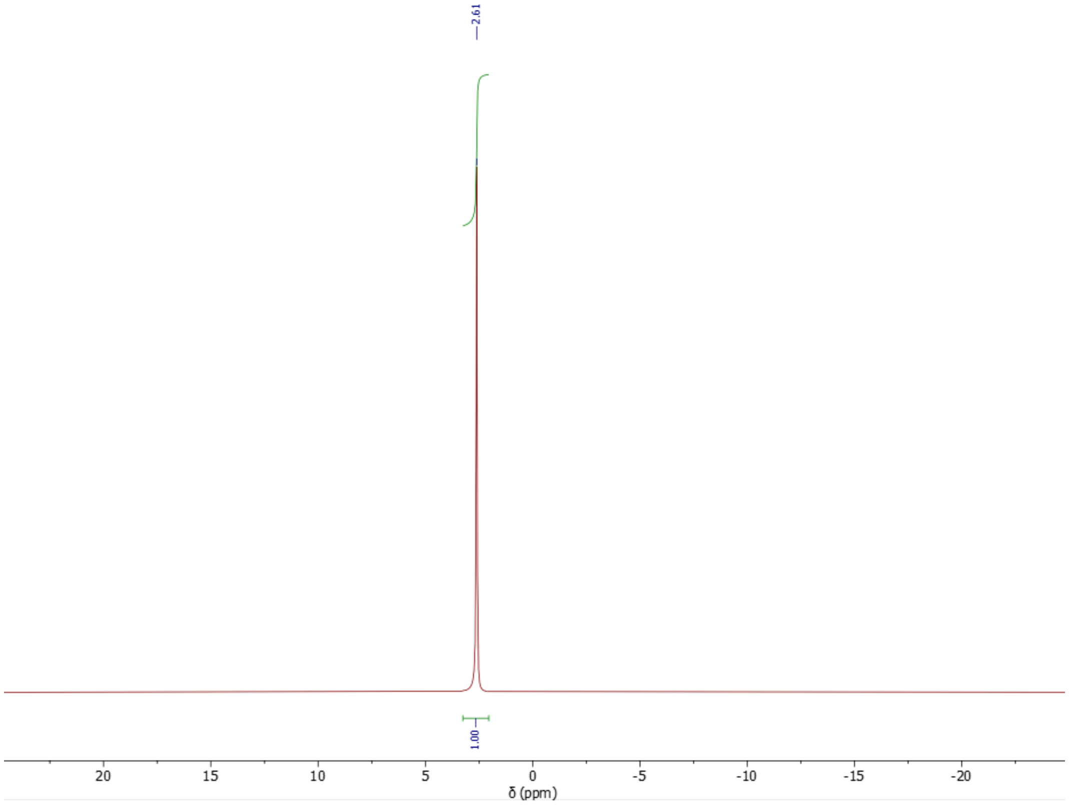

^{7}Li NMR spectra of (dimethylphenylsilyl)methyllithium **1·TMEDA** complex

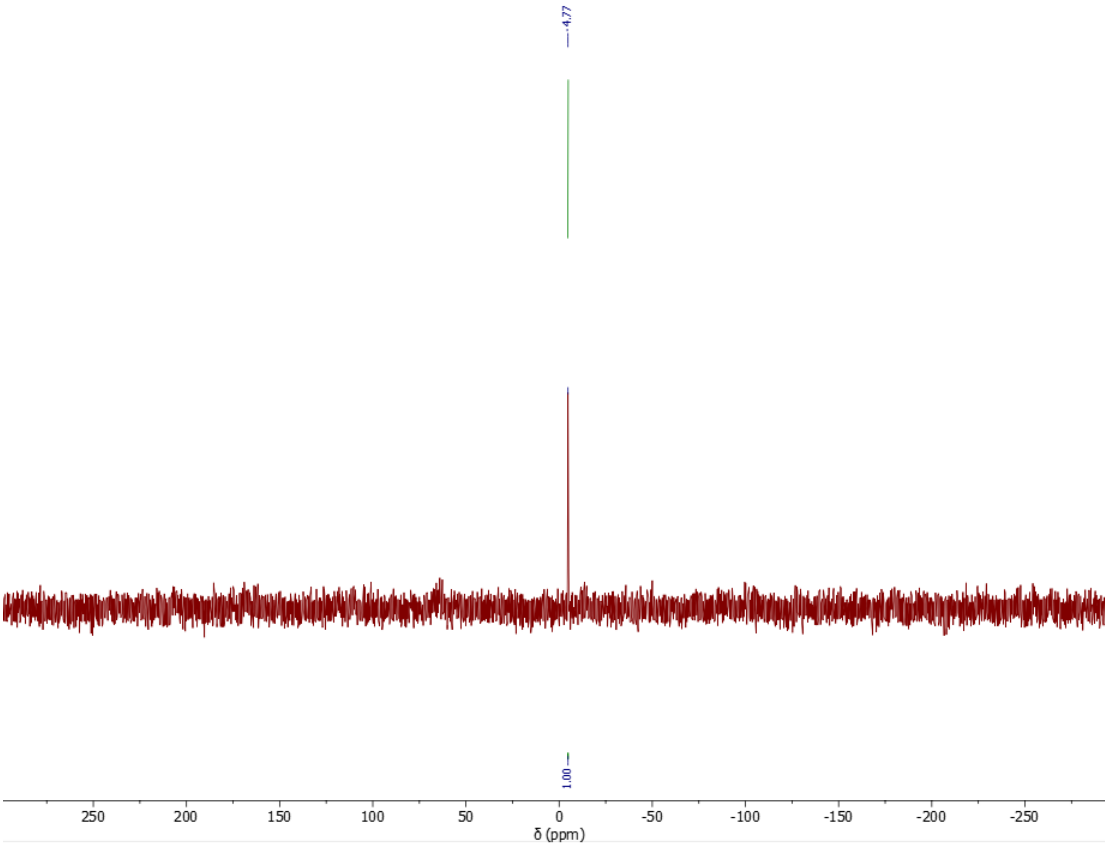

^{29}Si NMR spectra of (dimethylphenylsilyl)methyllithium **1·TMEDA** complex

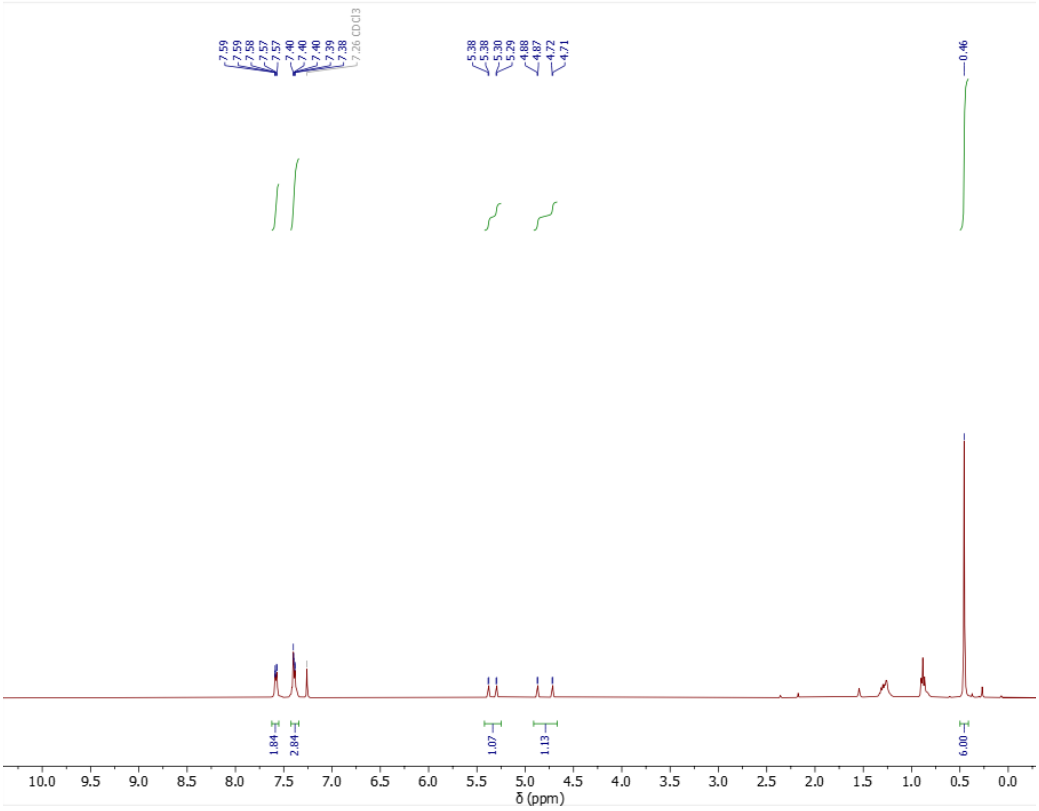

^{1}H NMR spectra of (1-fluorovinyl)dimethyl(phenyl)silane, **5**

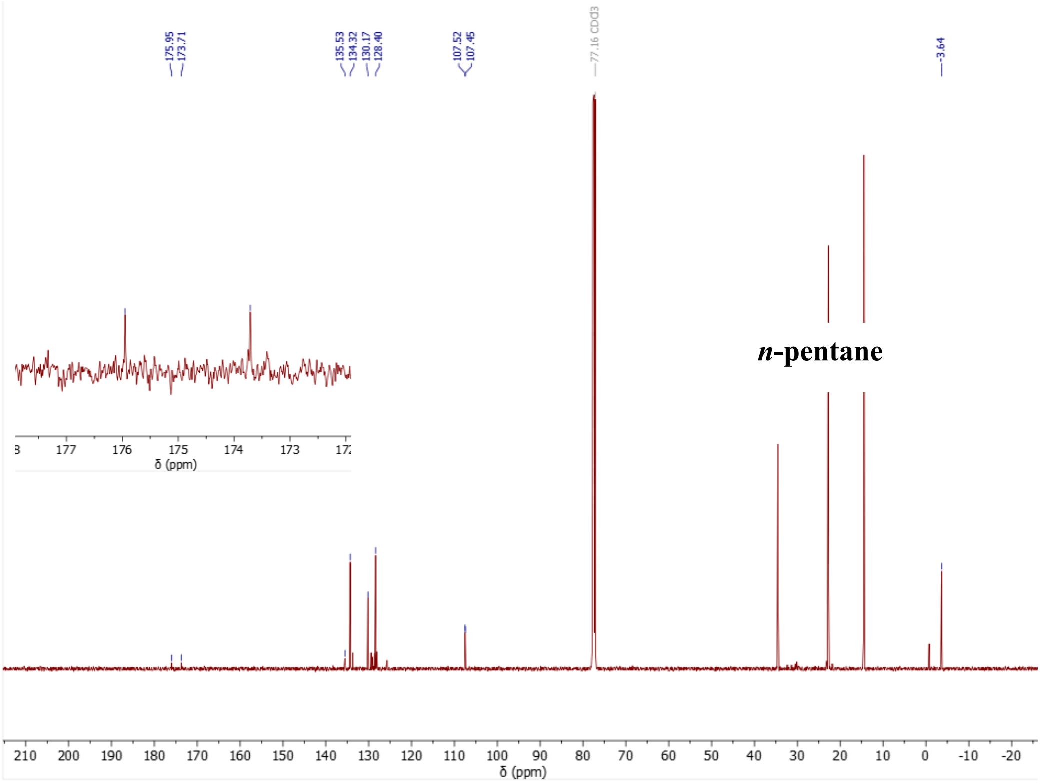

^{13}C NMR spectra of (1-fluorovinyl)dimethyl(phenyl)silane, **5**

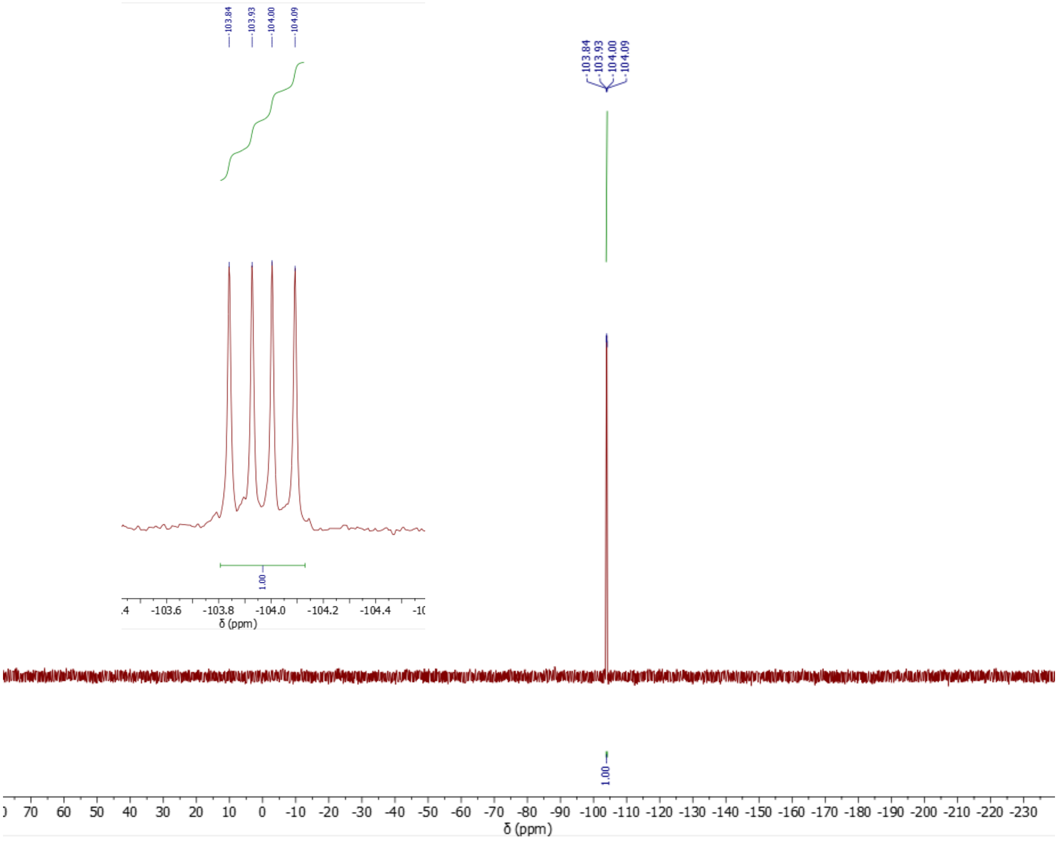

^{19}F NMR spectra of (1-fluorovinyl)dimethyl(phenyl)silane, **5**

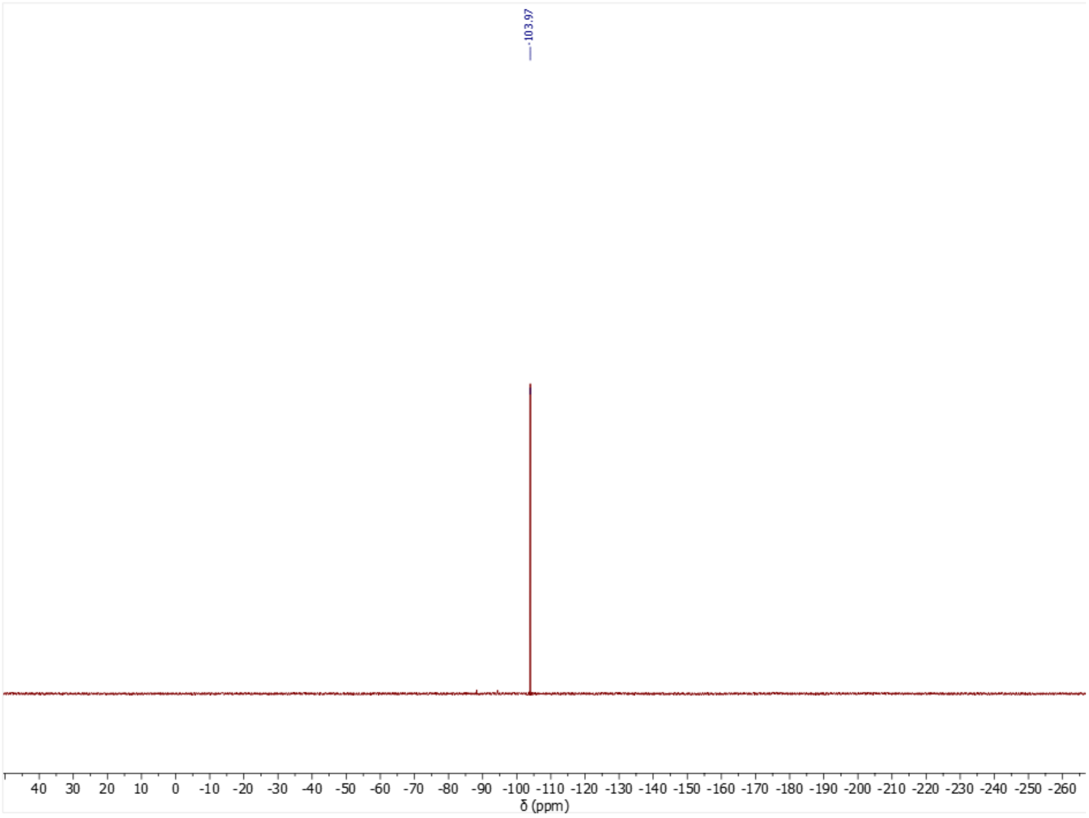

$^{19}F\{^{1}H\}$ NMR spectra of (1-fluorovinyl)dimethyl(phenyl)silane, **5**

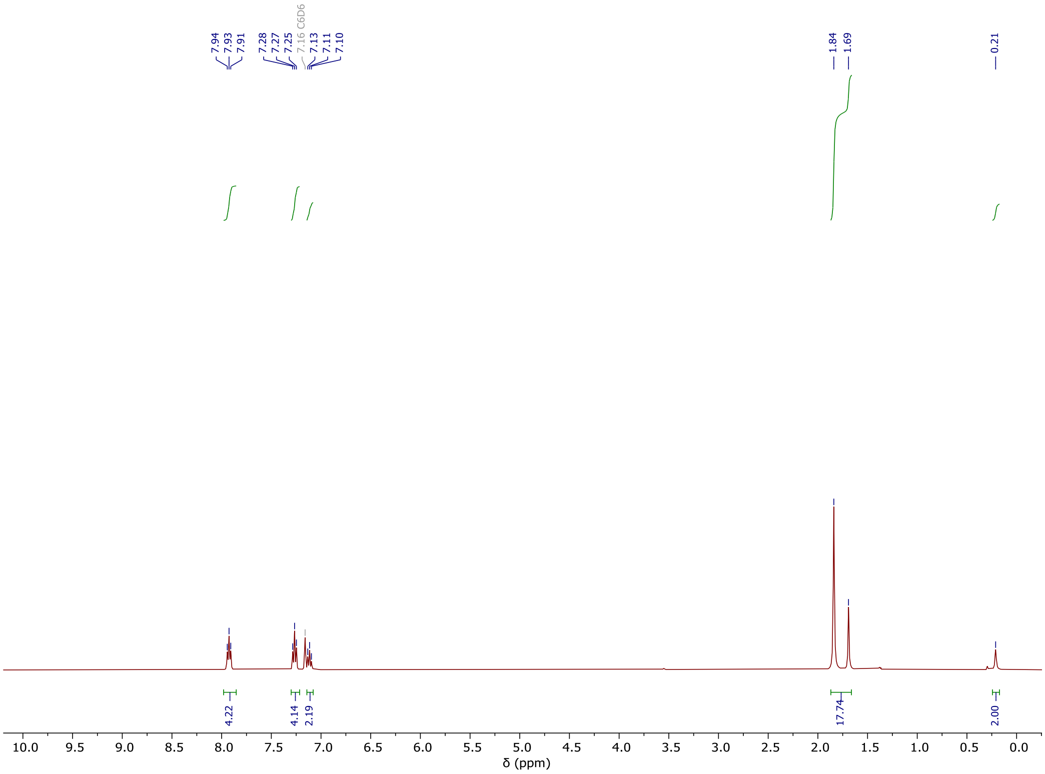

^{1}H NMR spectra of ((diphenylphosphaneyl)methyl)lithium **2·TMEDA** complex

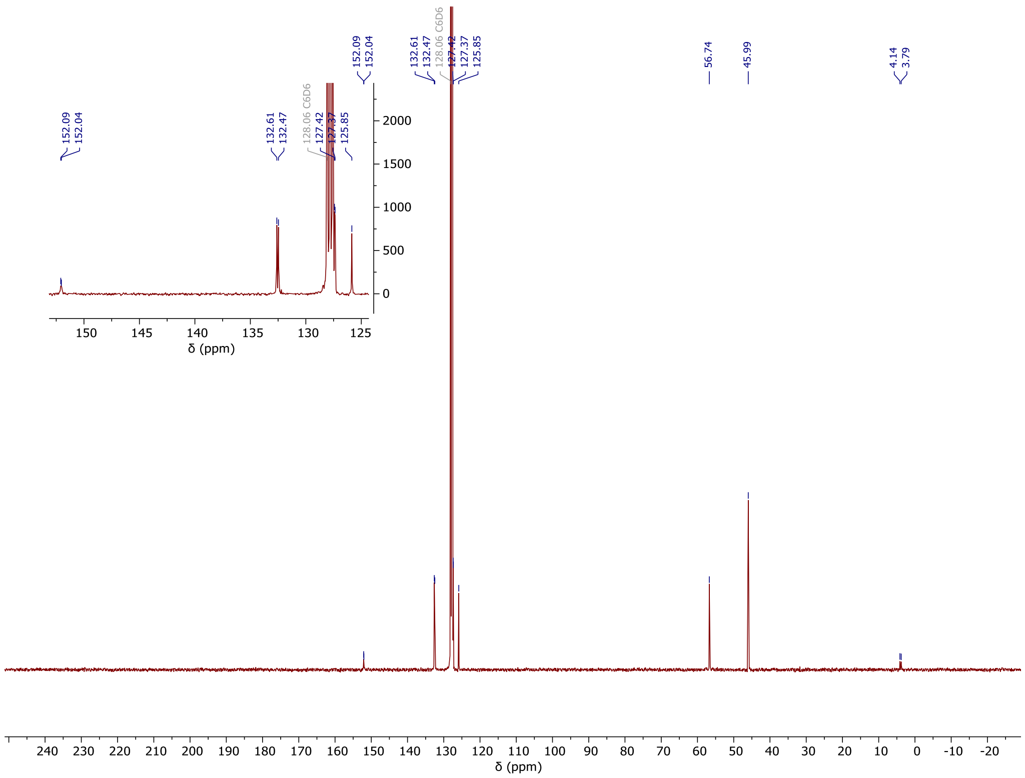

^{13}C NMR spectra of ((diphenylphosphaneyl)methyl)lithium **2·TMEDA** complex

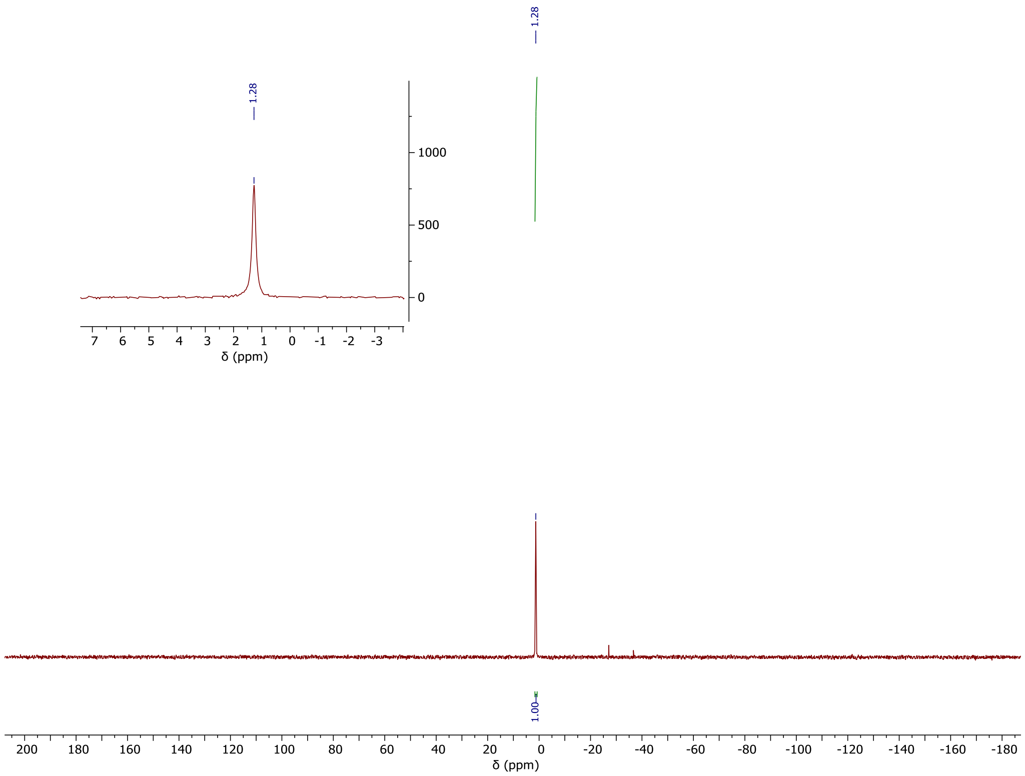

^{31}P{^{1}H} NMR spectra of ((diphenylphosphaneyl)methyl)lithium **2·TMEDA** complex

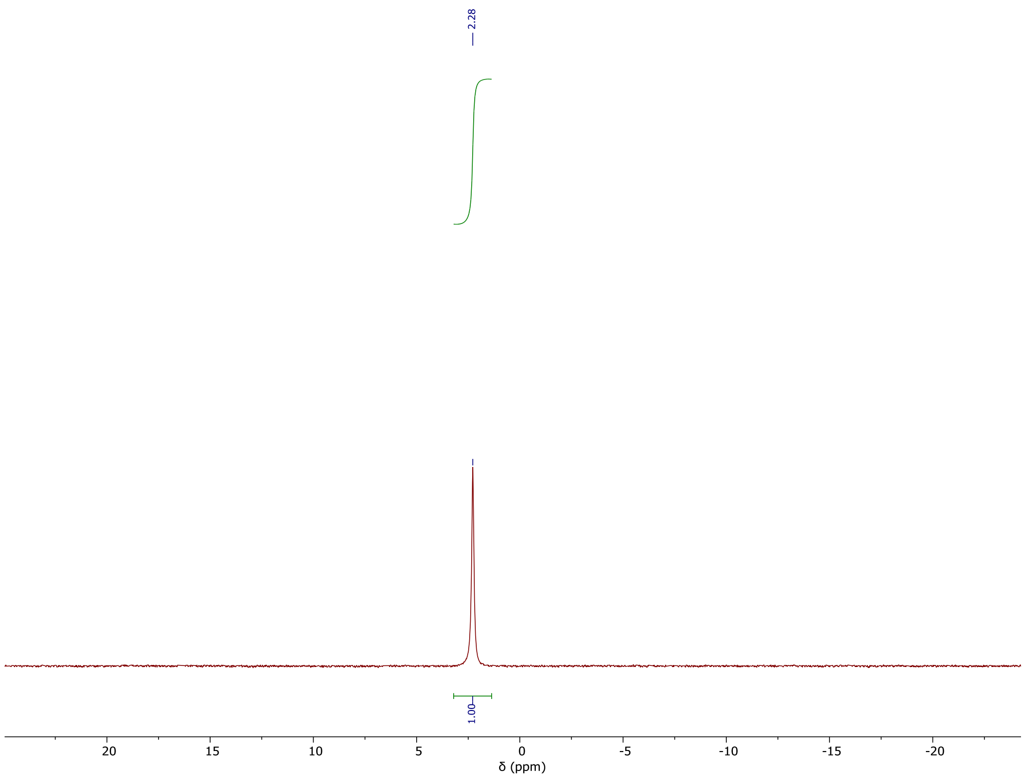

^{7}Li NMR spectra of ((diphenylphosphaneyl)methyl)lithium **2·TMEDA** complex

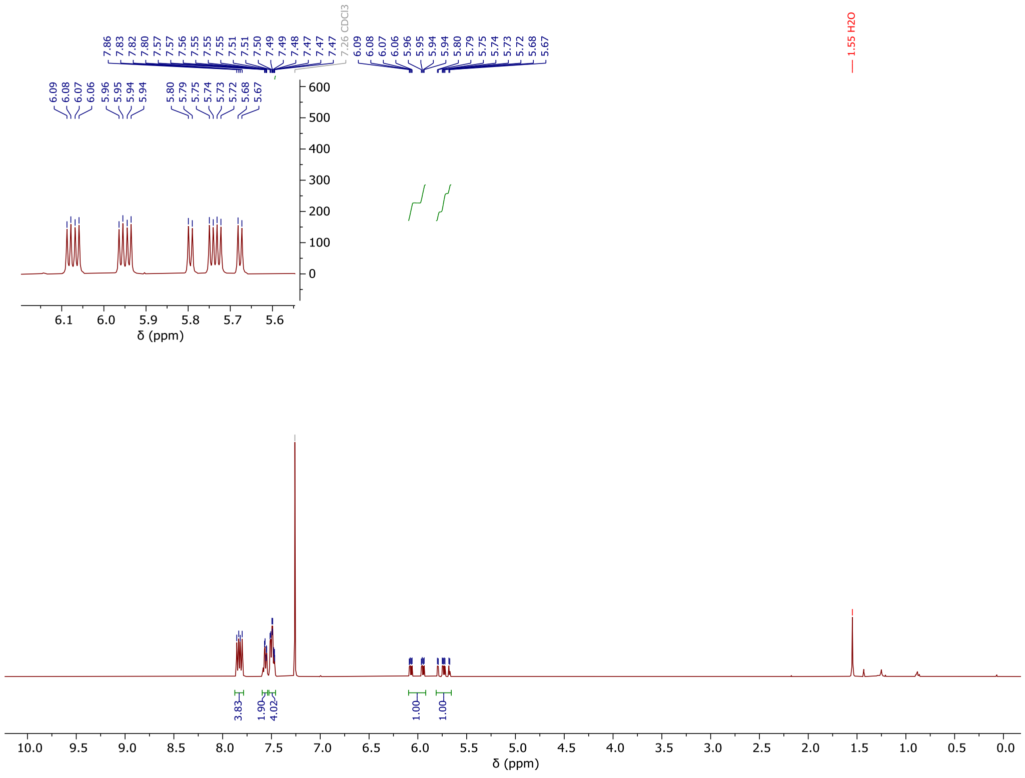

^{1}H NMR spectra of (1-fluorovinyl)diphenylphosphine sulfide, **6**

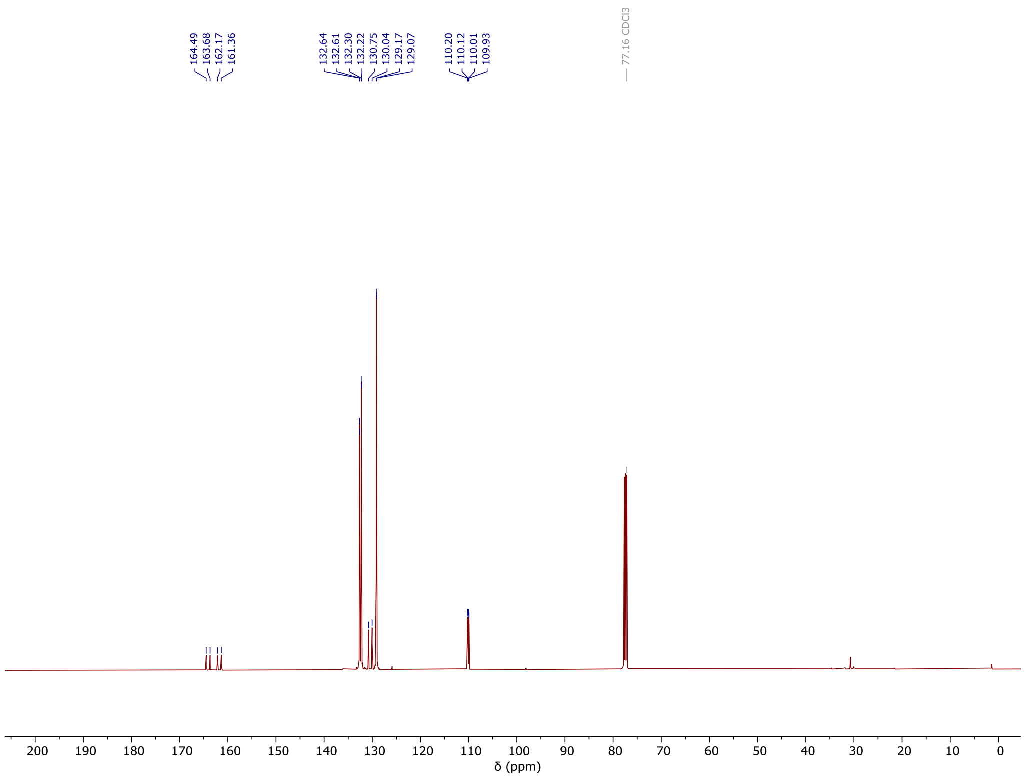

^{13}C NMR spectra of (1-fluorovinyl)diphenylphosphine sulfide, **6**

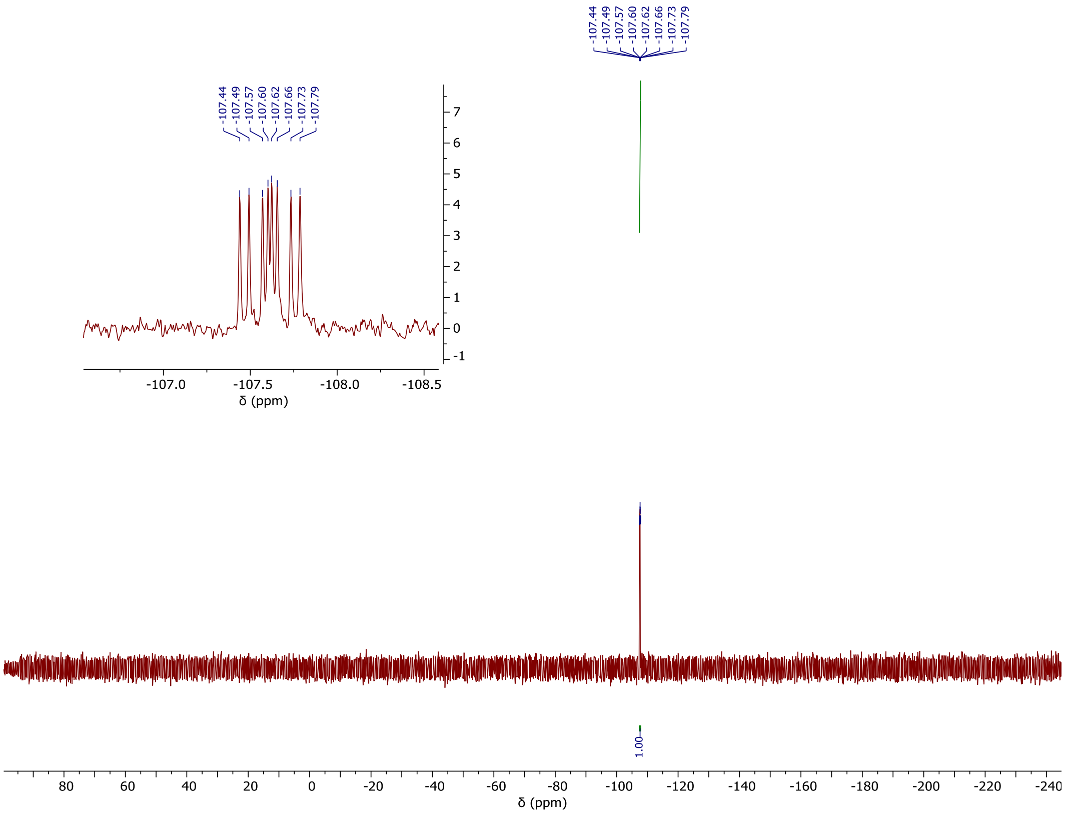

^{19}F NMR spectra of (1-fluorovinyl)diphenylphosphine sulfide, **6**

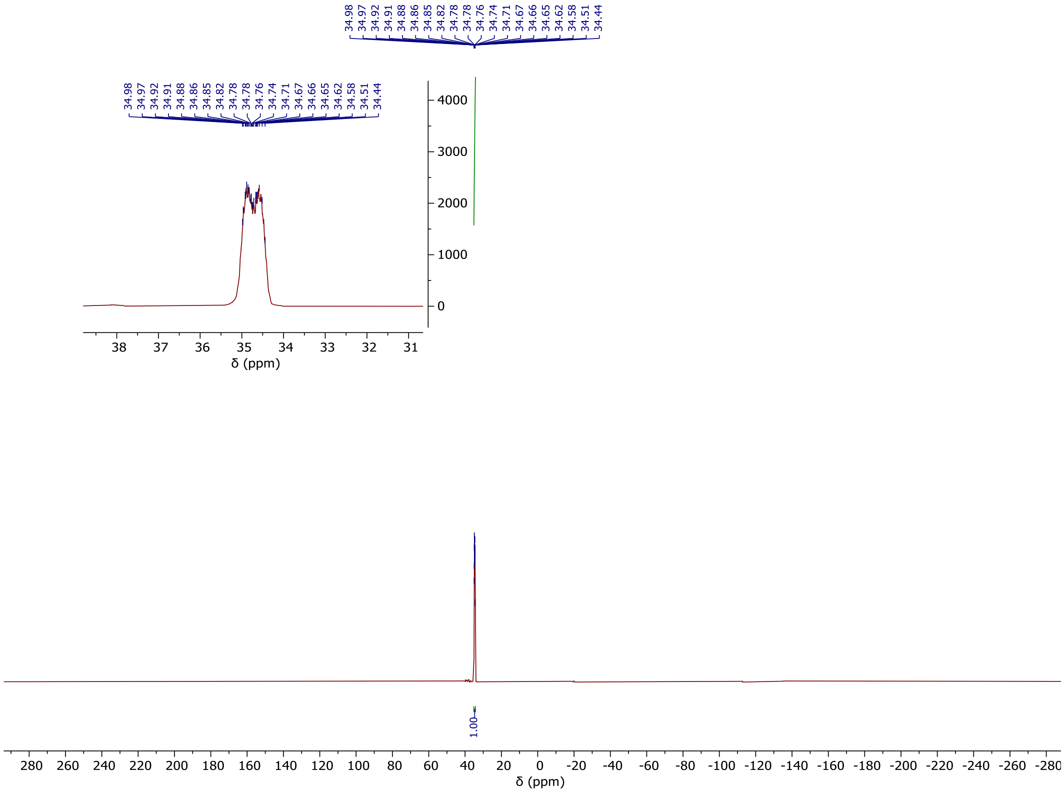

^{31}P NMR spectra of (1-fluorovinyl)diphenylphosphine sulfide, **6**

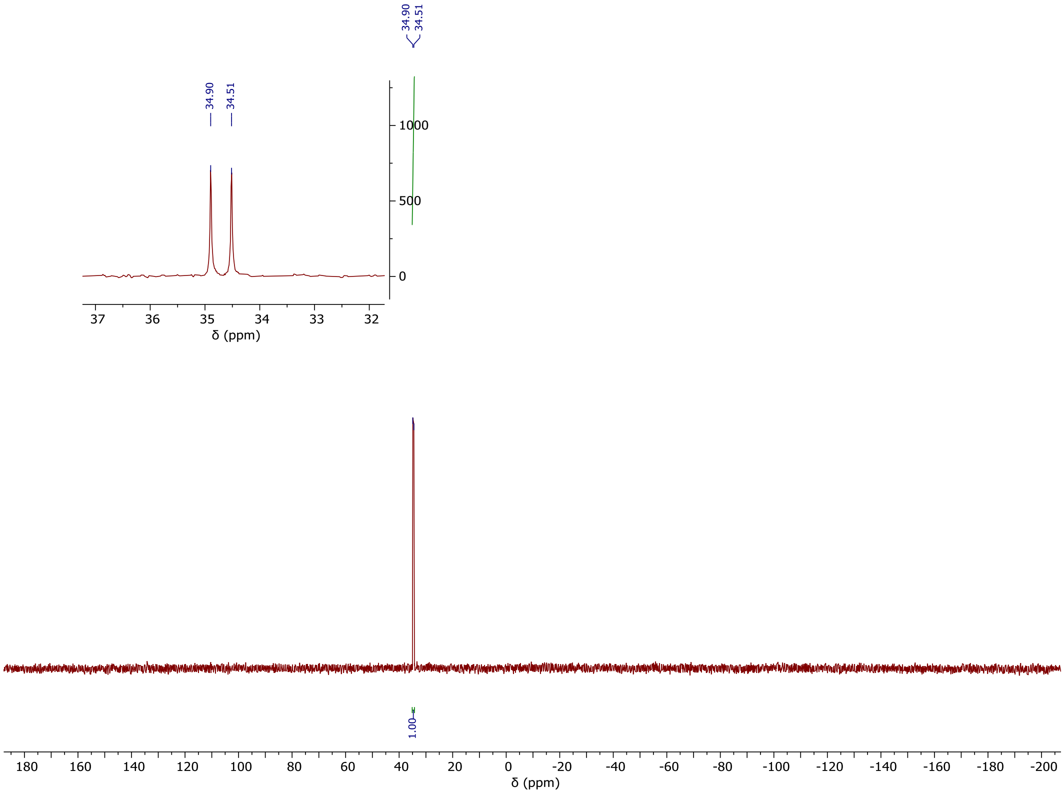

^{31}P{^{1}H} NMR spectra of (1-fluorovinyl)diphenylphosphine sulfide, **6**

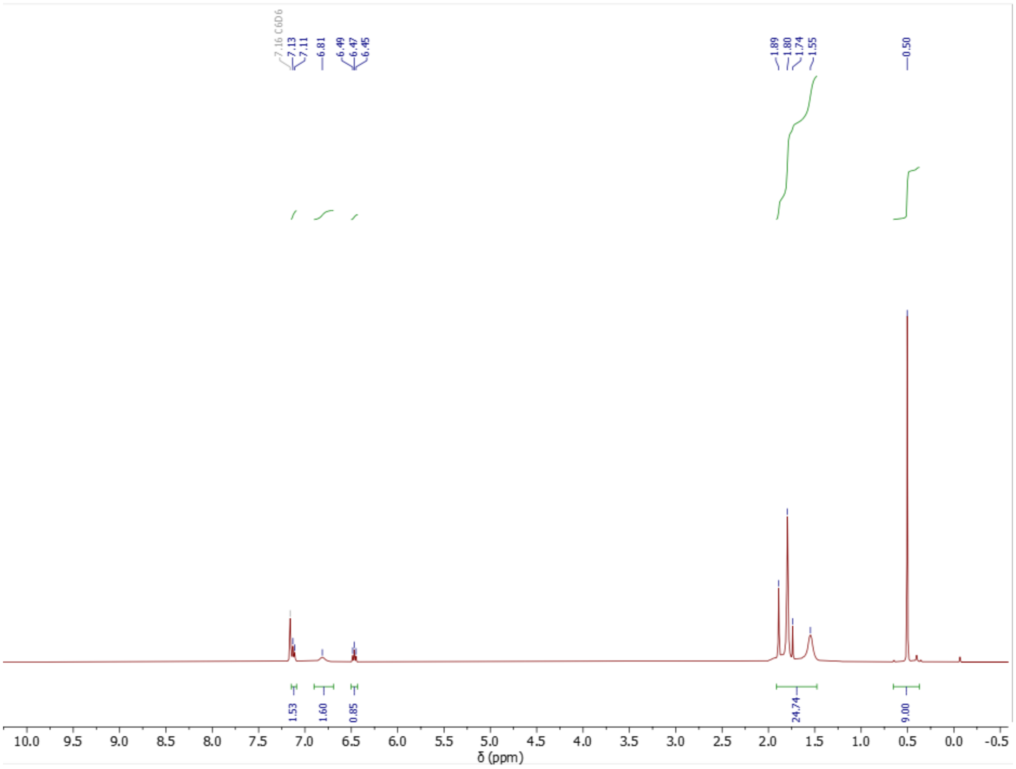

^{1}H NMR spectra of α-(trimethylsilyl)benzyllithium **3·PMDETA** complex

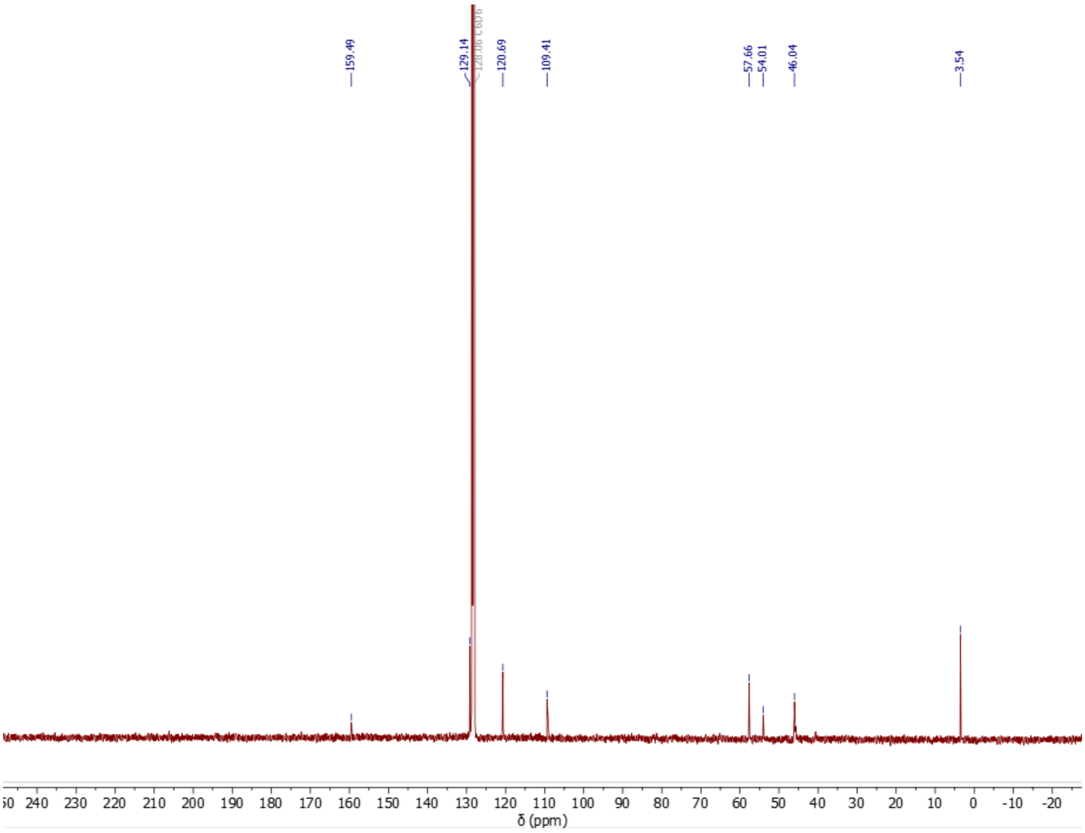

^{13}C NMR spectra of α-(trimethylsilyl)benzyllithium **3·PMDETA** complex

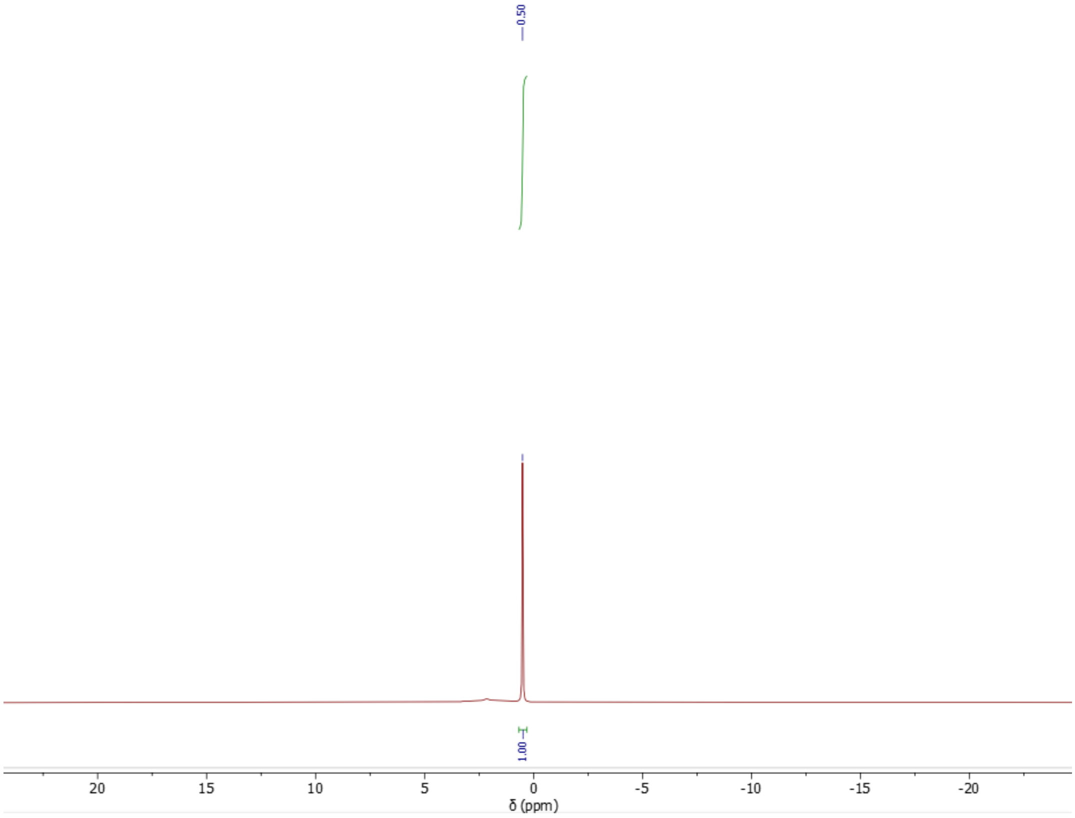

^{7}Li NMR spectra of α-(trimethylsilyl)benzyllithium **3·PMDETA** complex

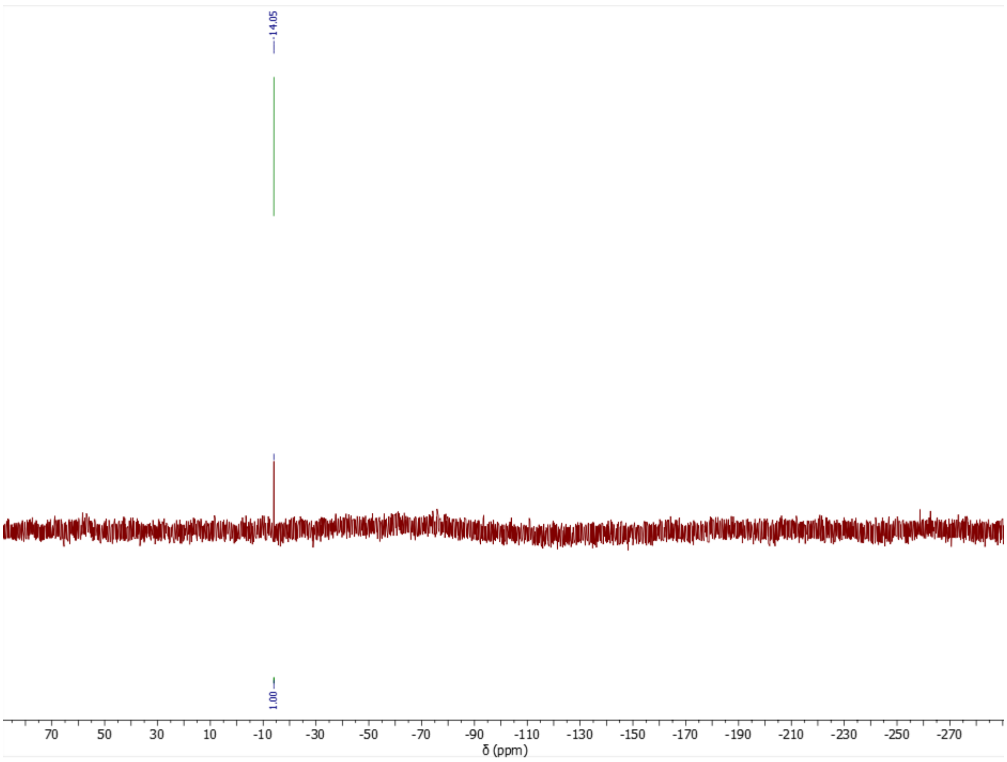

^{29}Si NMR spectra of α-(trimethylsilyl)benzyllithium **3·PMDETA** complex

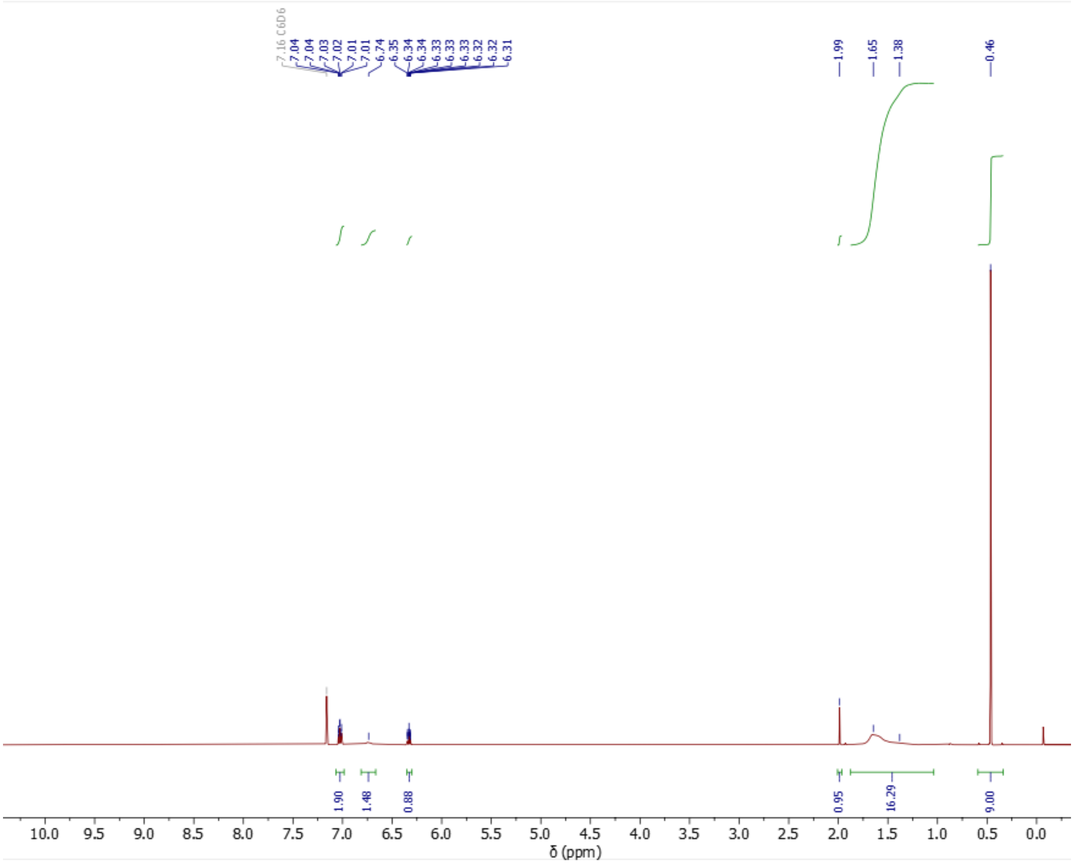

^{1}H NMR spectra of α-(trimethylsilyl)benzyllithium **3·TMEDA** complex

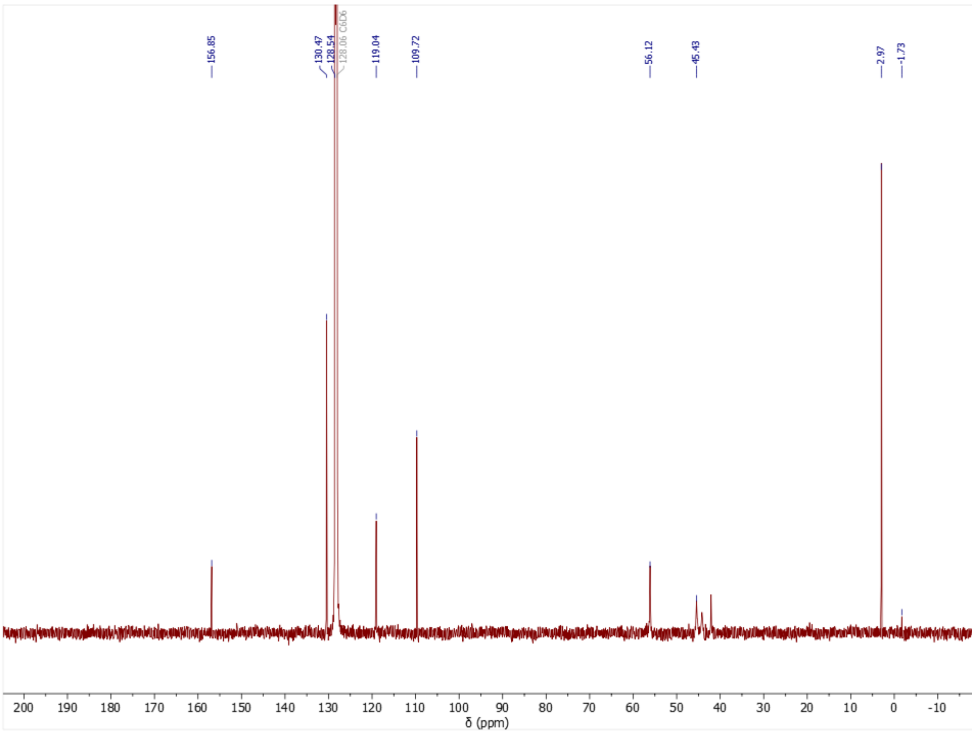

^{13}C NMR spectra of α-(trimethylsilyl)benzyllithium **3·TMEDA** complex

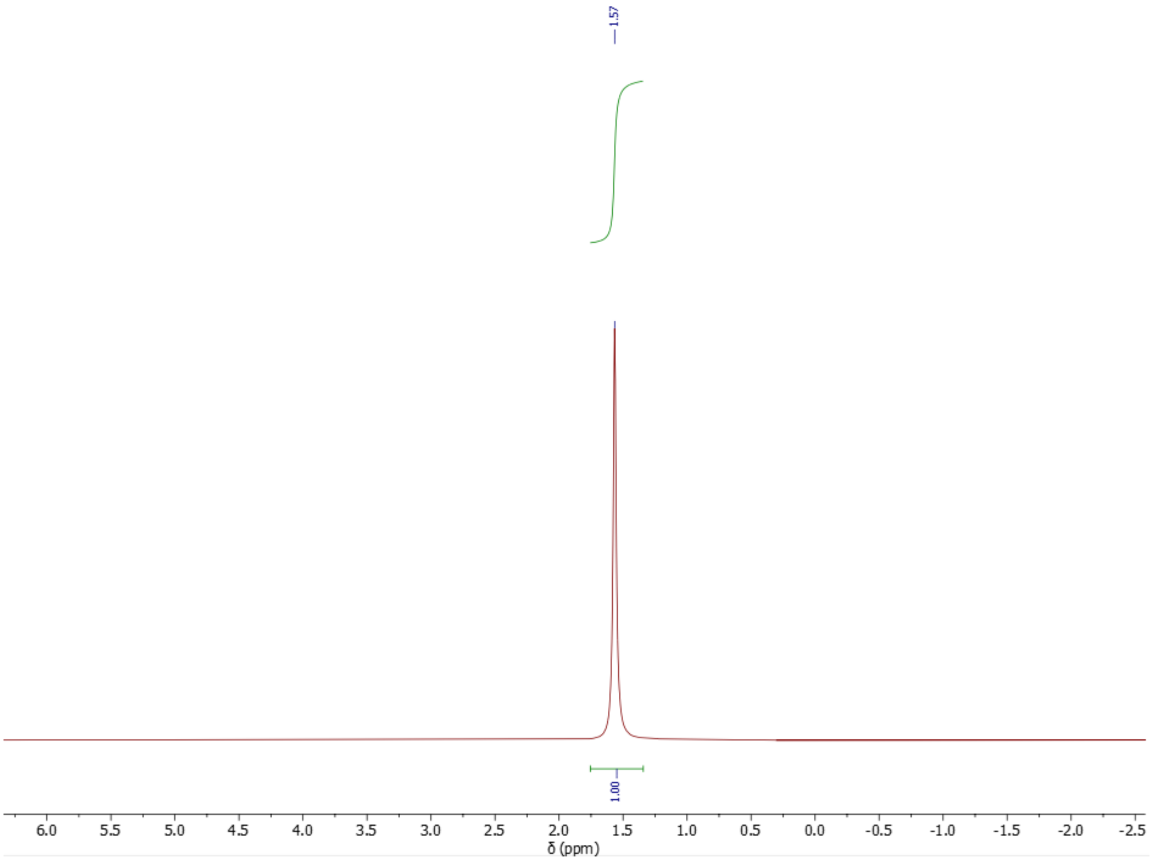

^{7}Li NMR spectra of α-(trimethylsilyl)benzyllithium **3·TMEDA** complex

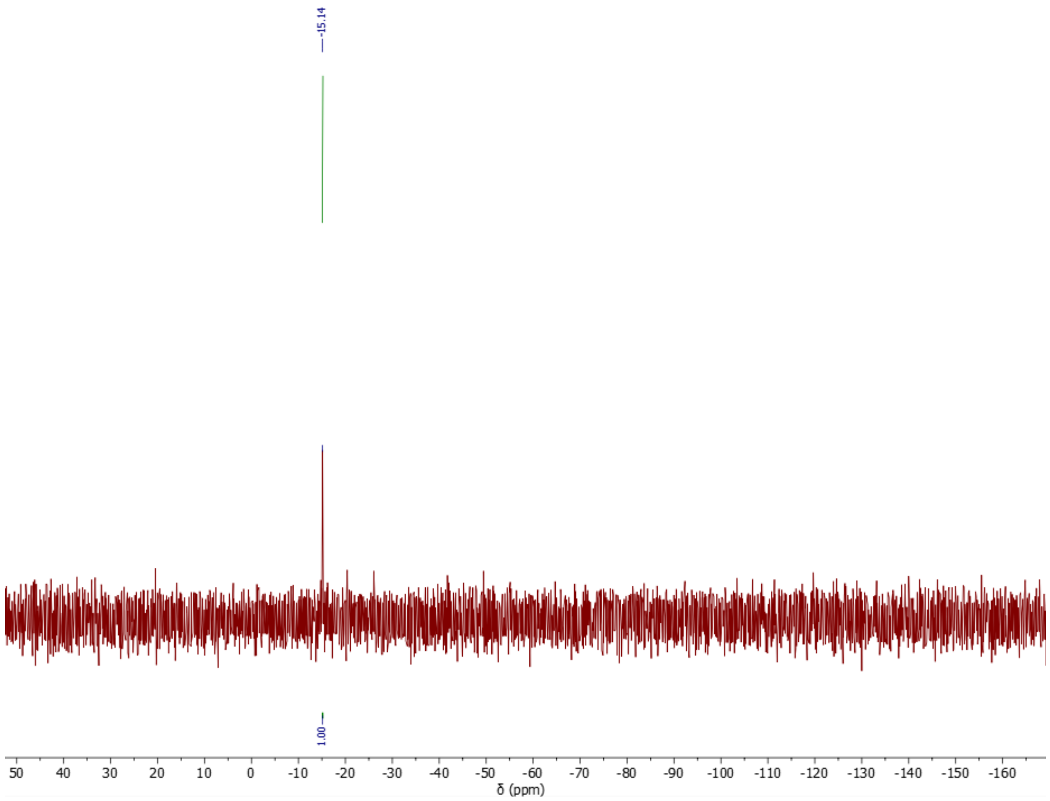

^{29}Si NMR spectra of α-(trimethylsilyl)benzyllithium **3·TMEDA** complex

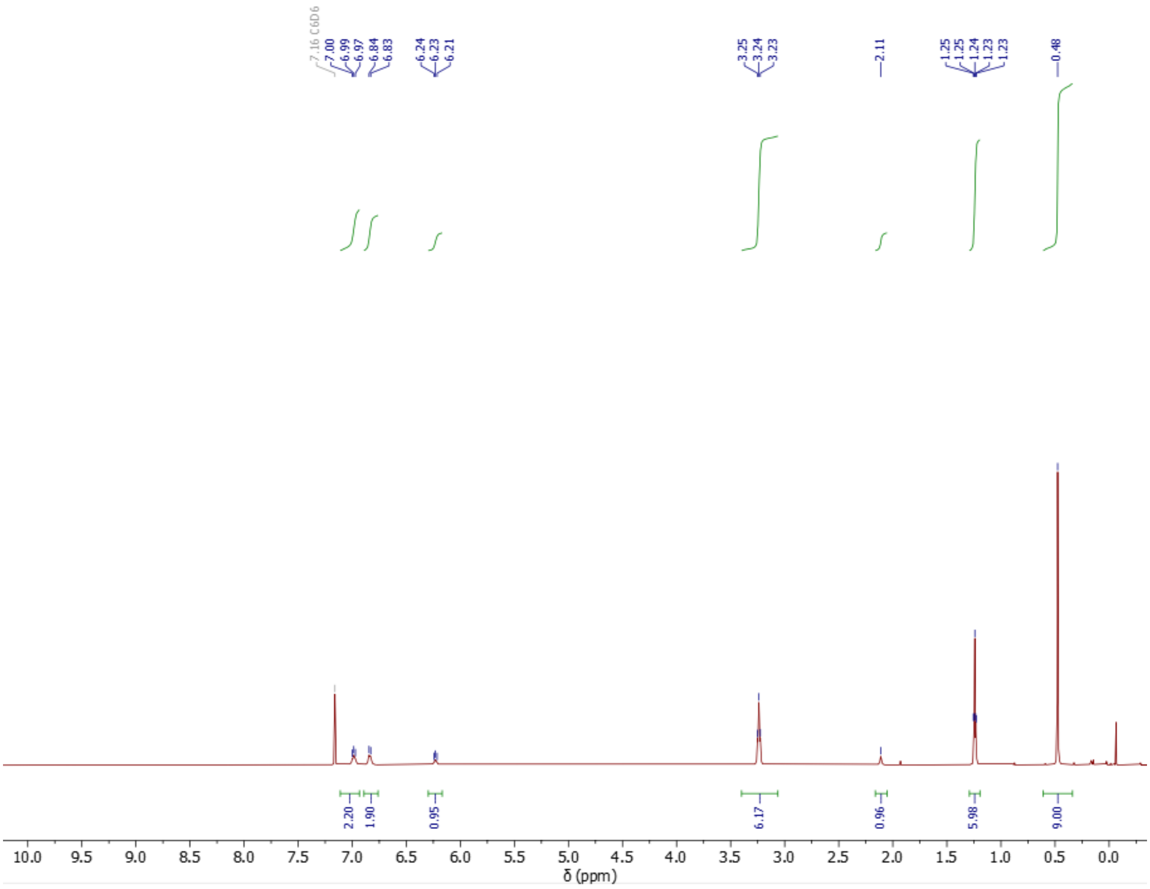

^{1}H NMR spectra of α-(trimethylsilyl)benzyllithium **3·THF$_{1.5}$** complex

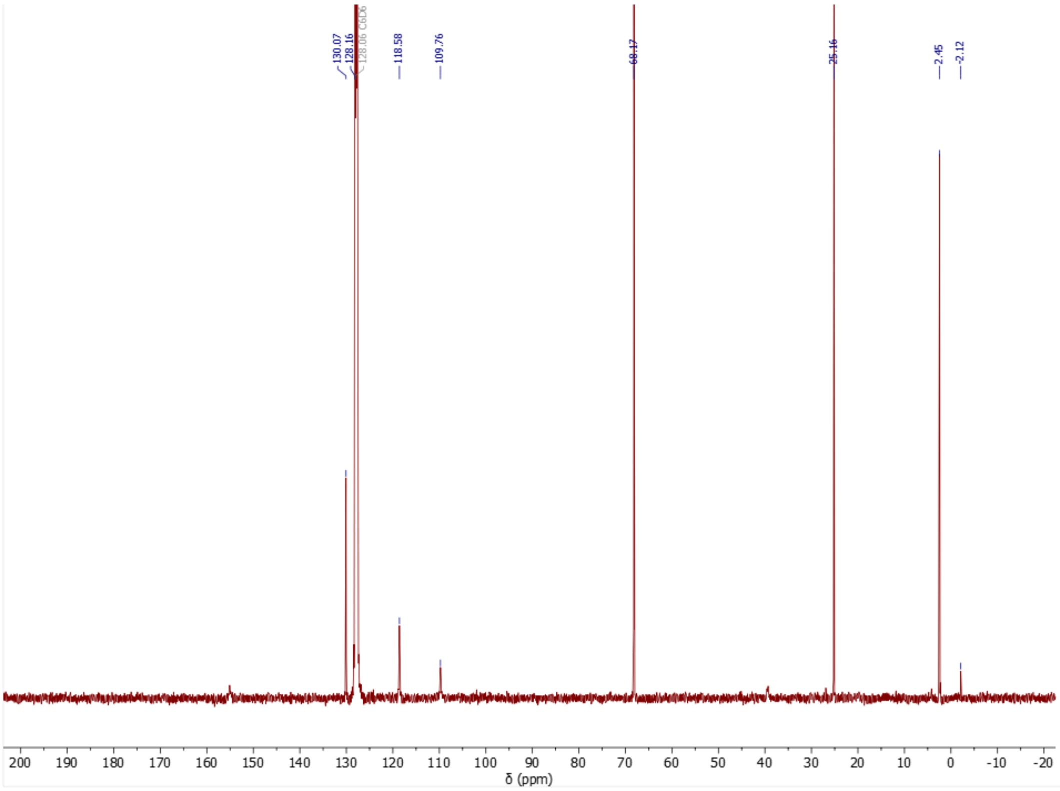

^{13}C NMR spectra of α-(trimethylsilyl)benzyllithium **3·THF$_{1.5}$** complex

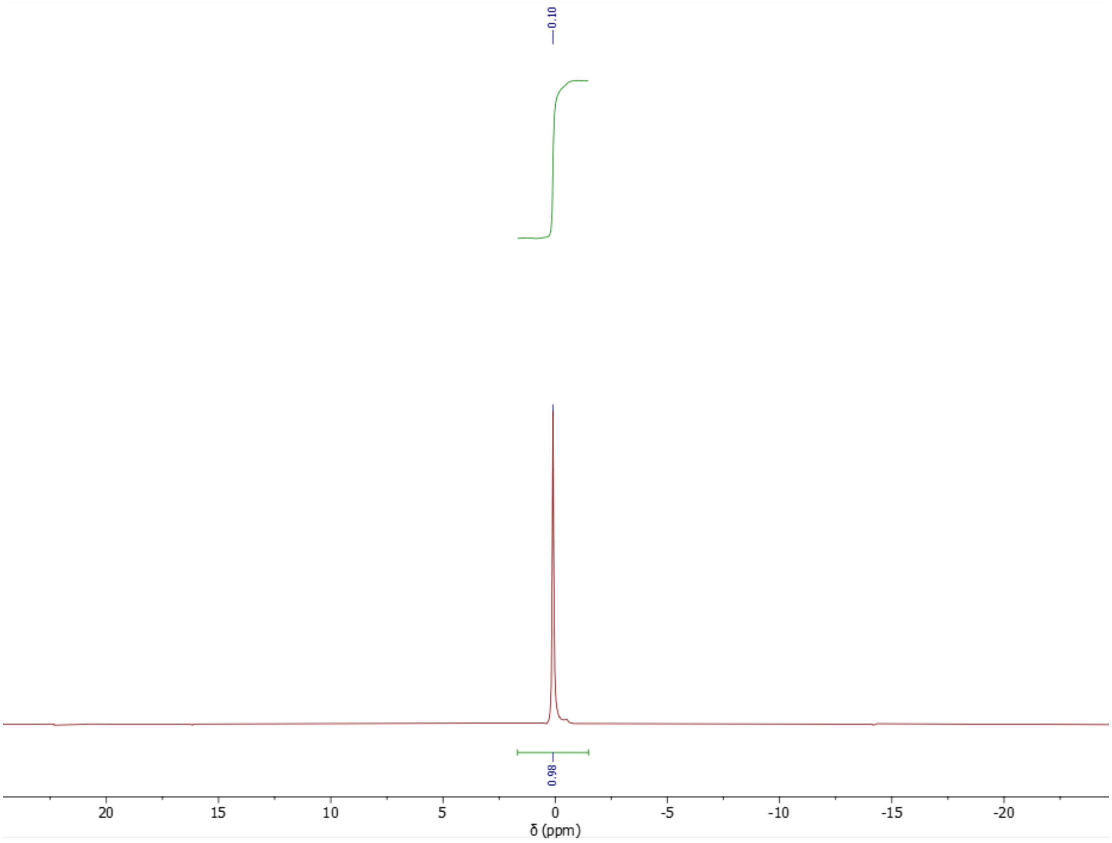

^{7}Li NMR spectra of α-(trimethylsilyl)benzyllithium **3·THF$_{1.5}$** complex

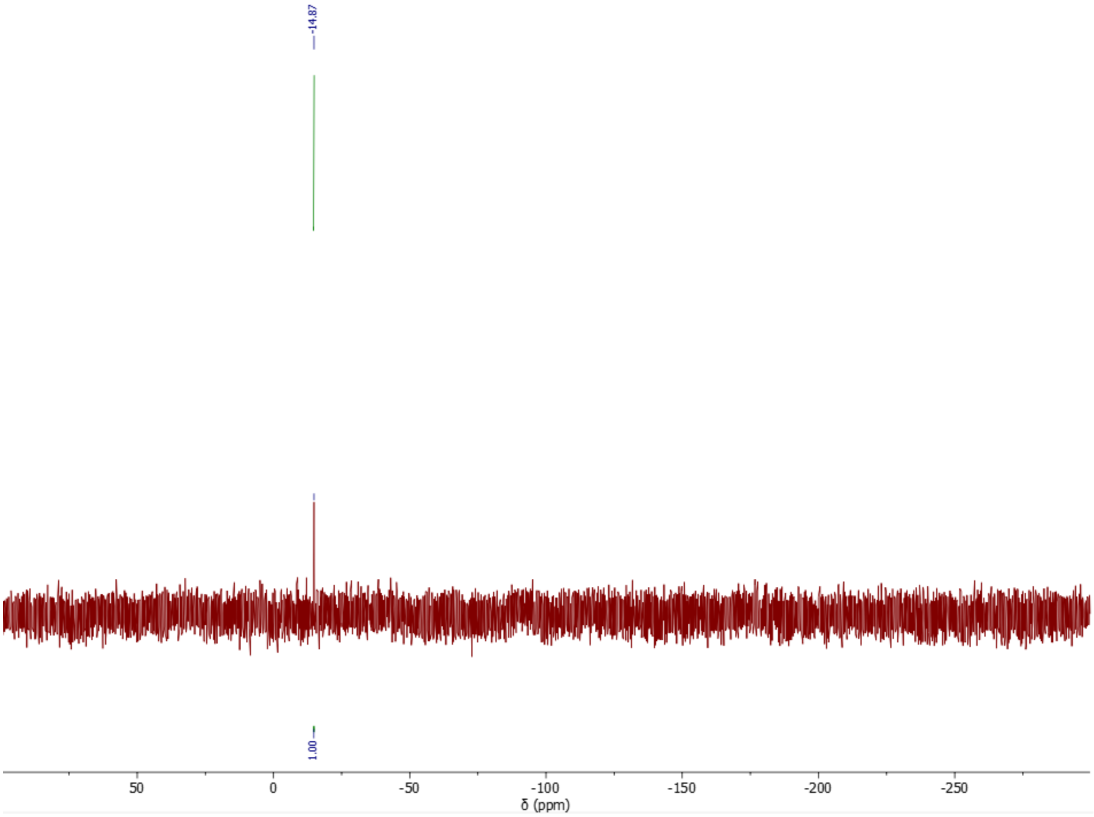

^{29}Si NMR spectra of α-(trimethylsilyl)benzyllithium **3·THF$_{1.5}$** complex

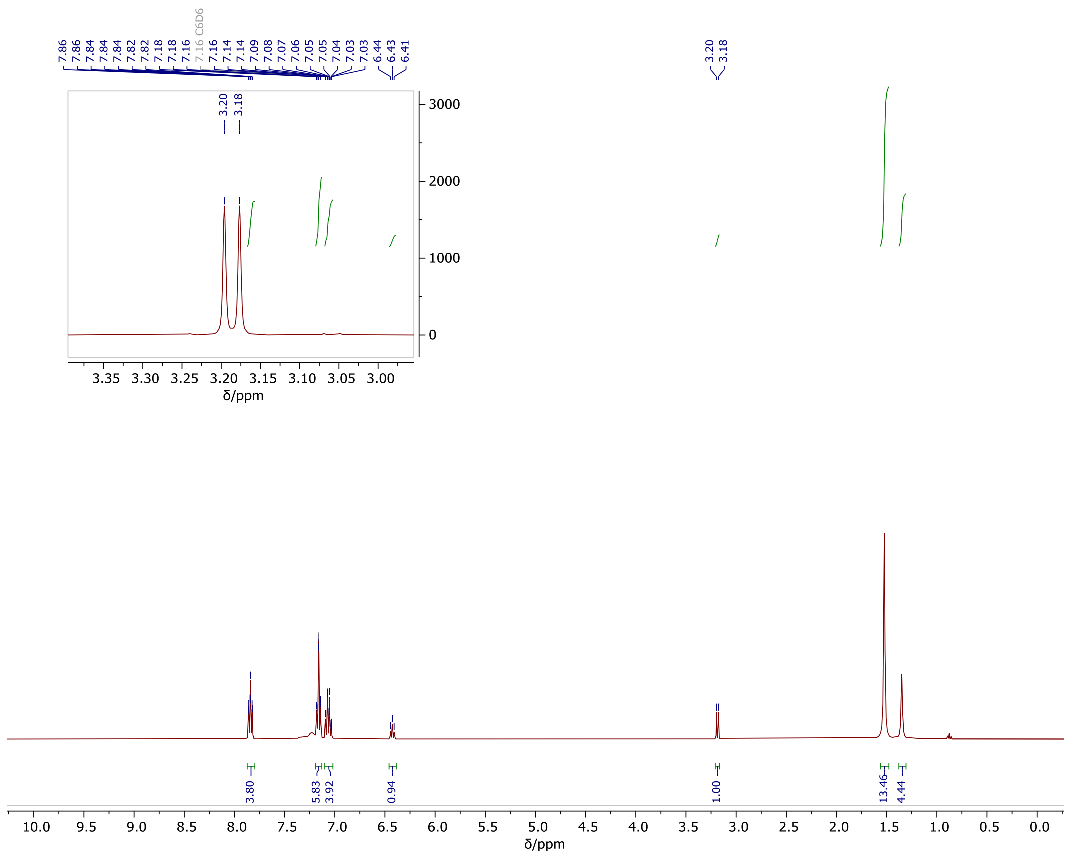

^{1}H NMR spectra of ((diphenylphosphaneyl)(phenyl)methyl)lithium **4·TMEDA** complex

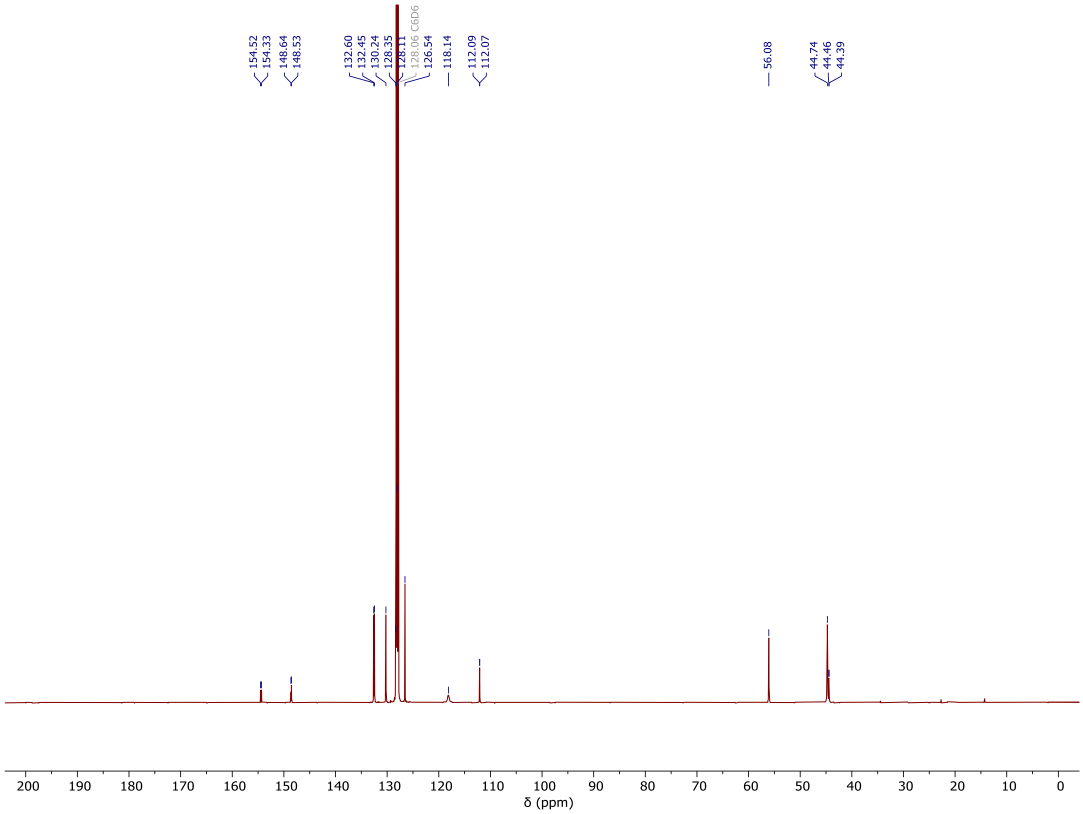

^{13}C NMR spectra of ((diphenylphosphaneyl)(phenyl)methyl)lithium **4·TMEDA** complex

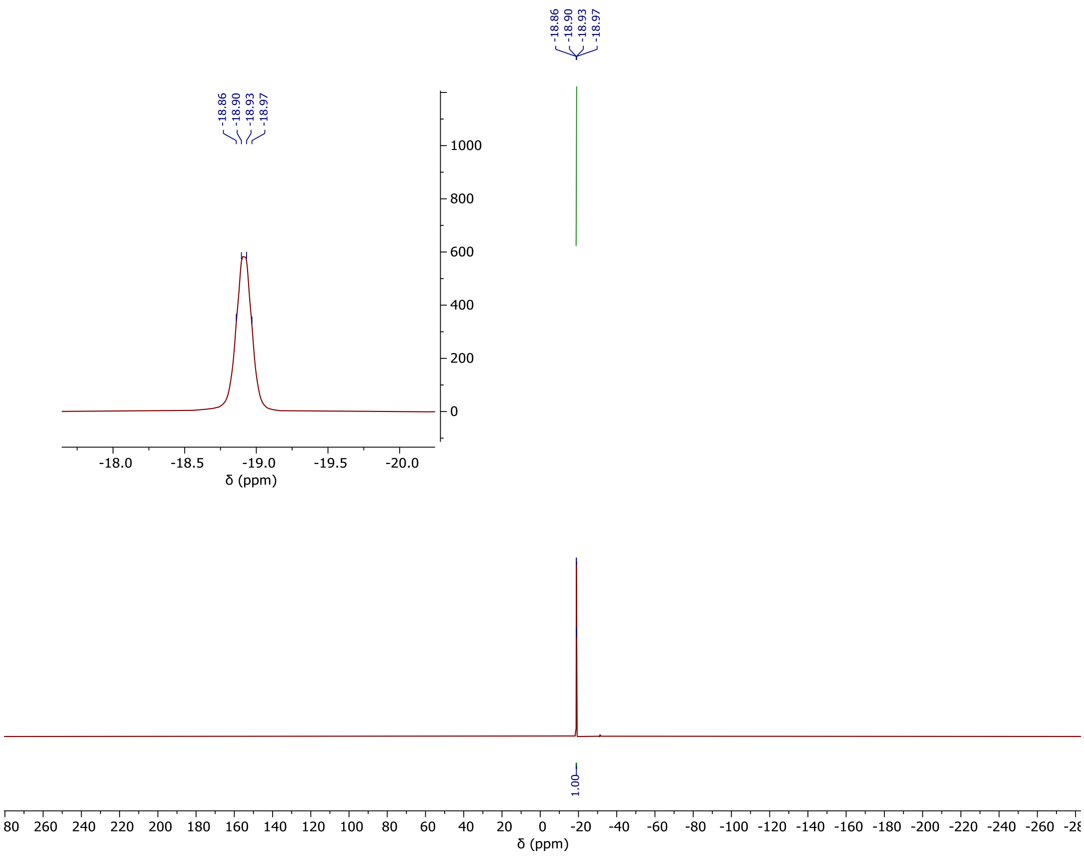

^{31}P NMR spectra of ((diphenylphosphaneyl)(phenyl)methyl)lithium **4·TMEDA** complex

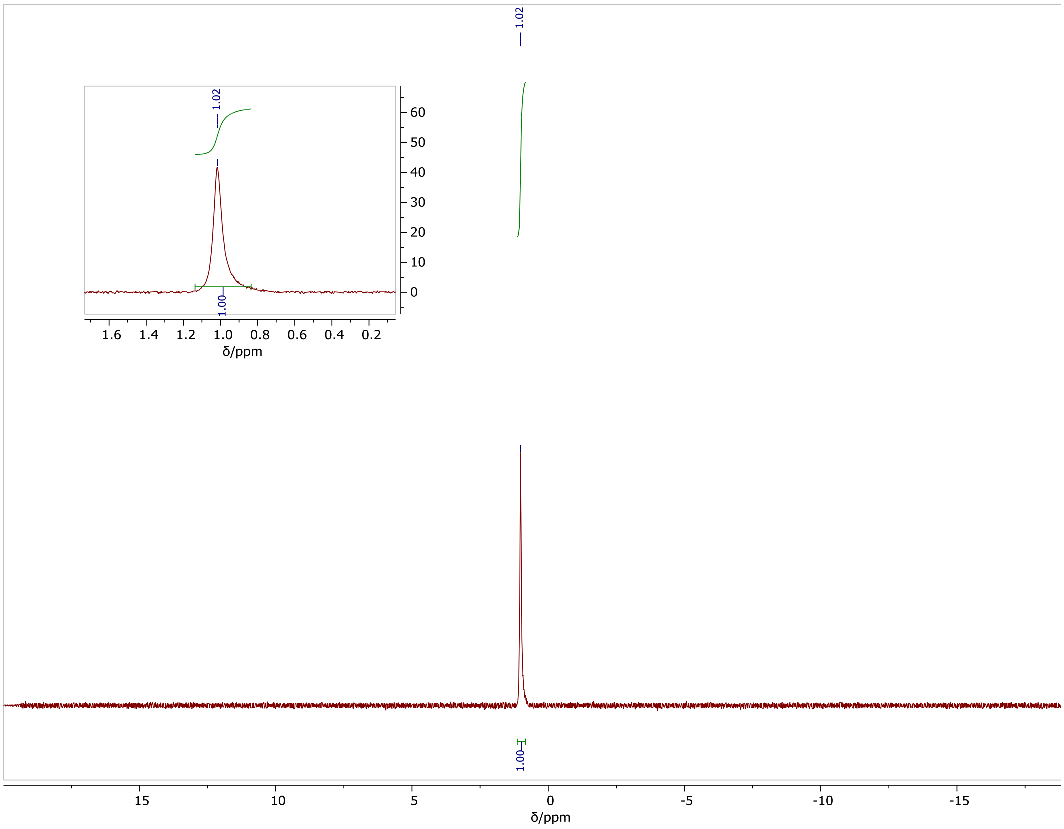

^{7}Li NMR spectra of ((diphenylphosphaneyl)(phenyl)methyl)lithium **4·TMEDA** complex

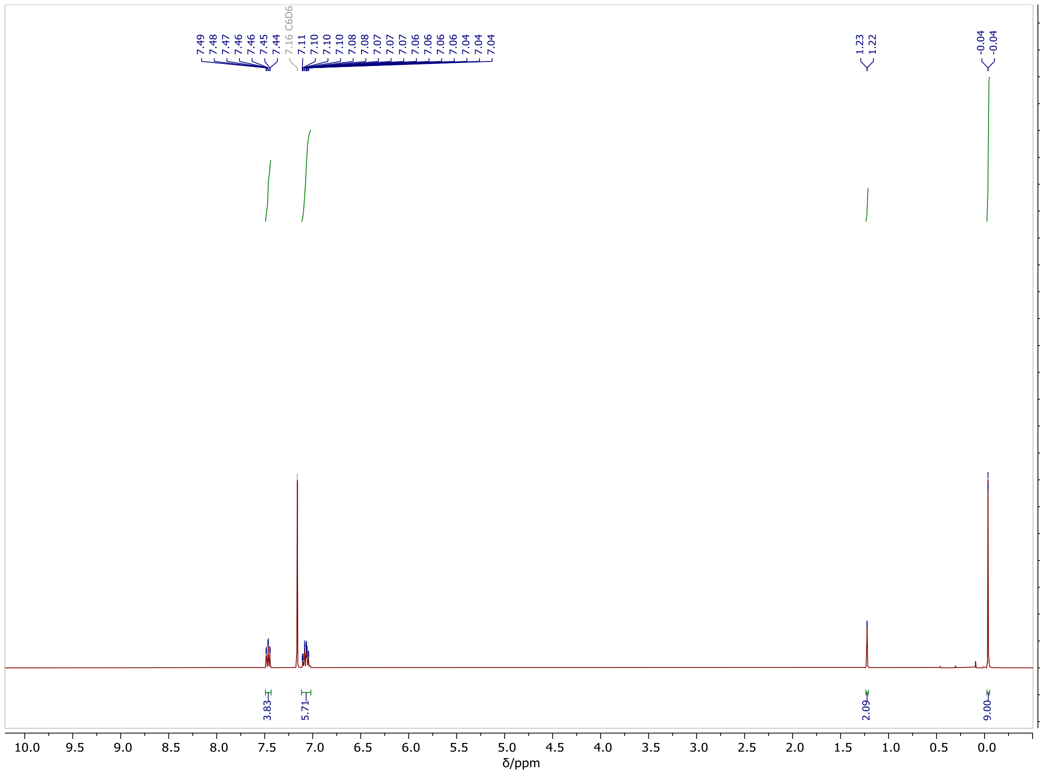

^{1}H NMR spectra of diphenyl((trimethylsilyl)methyl)phosphane

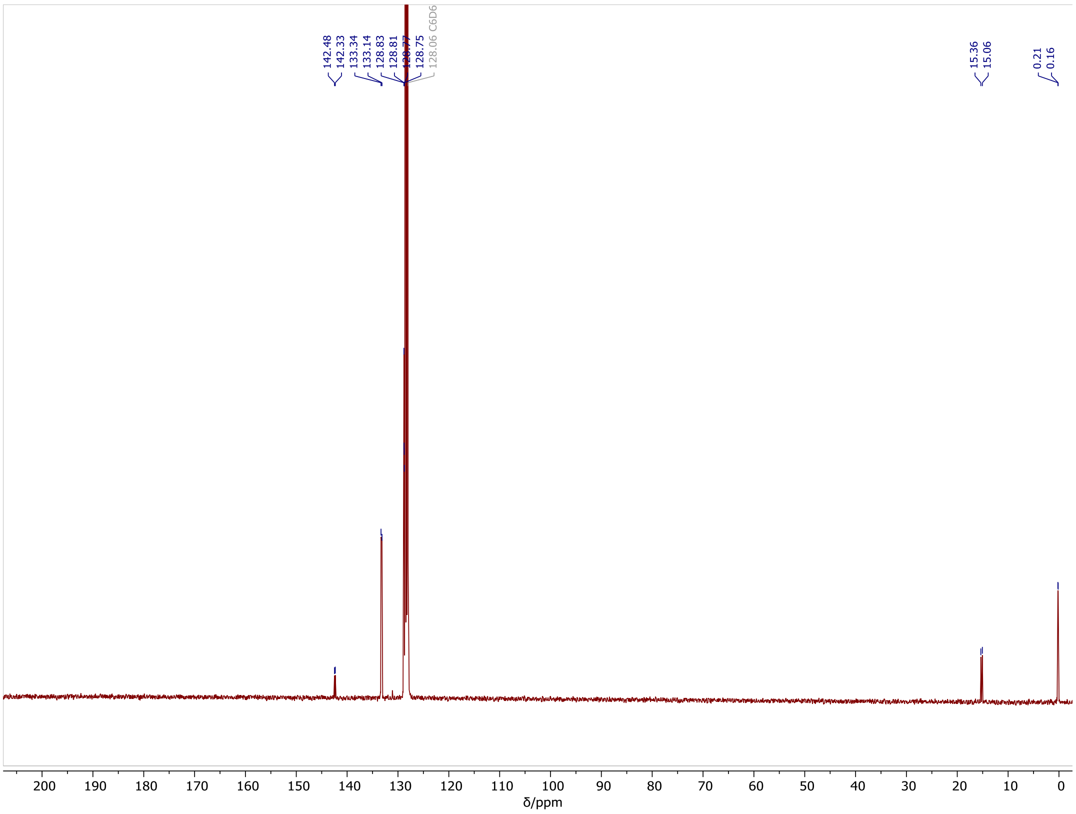

^{13}C NMR spectra of diphenyl((trimethylsilyl)methyl)phosphane

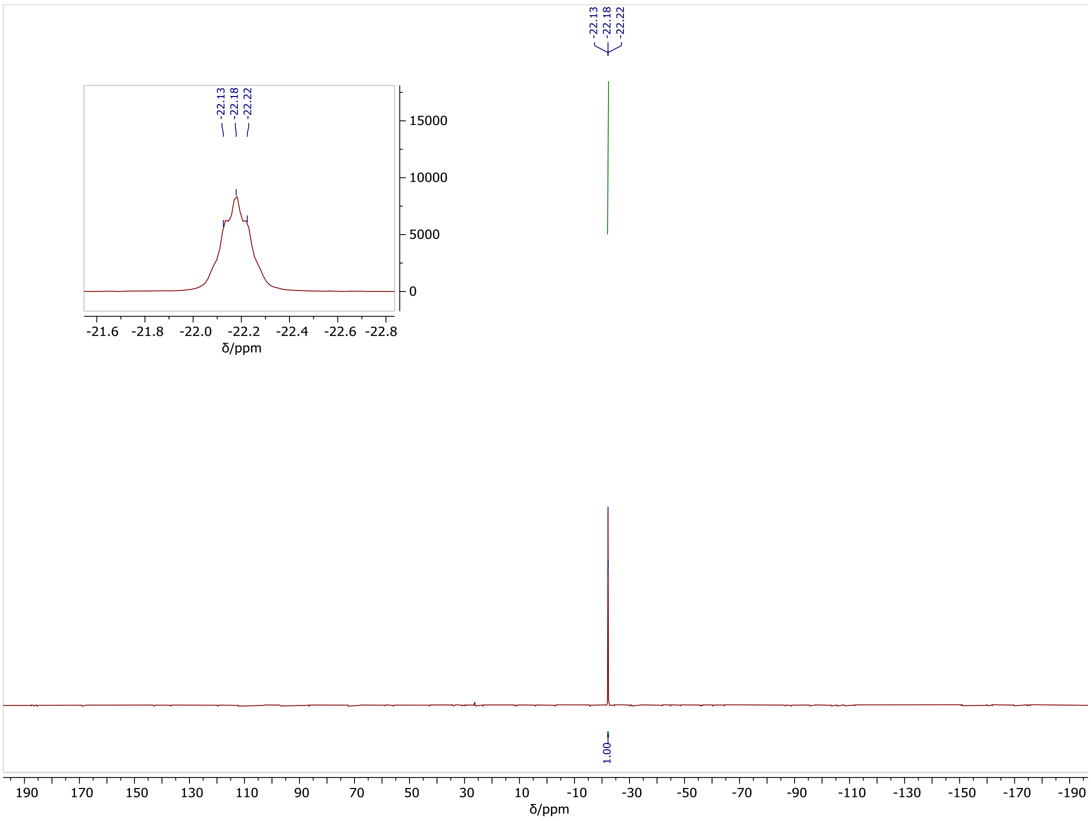

^{31}P NMR spectra of diphenyl((trimethylsilyl)methyl)phosphane

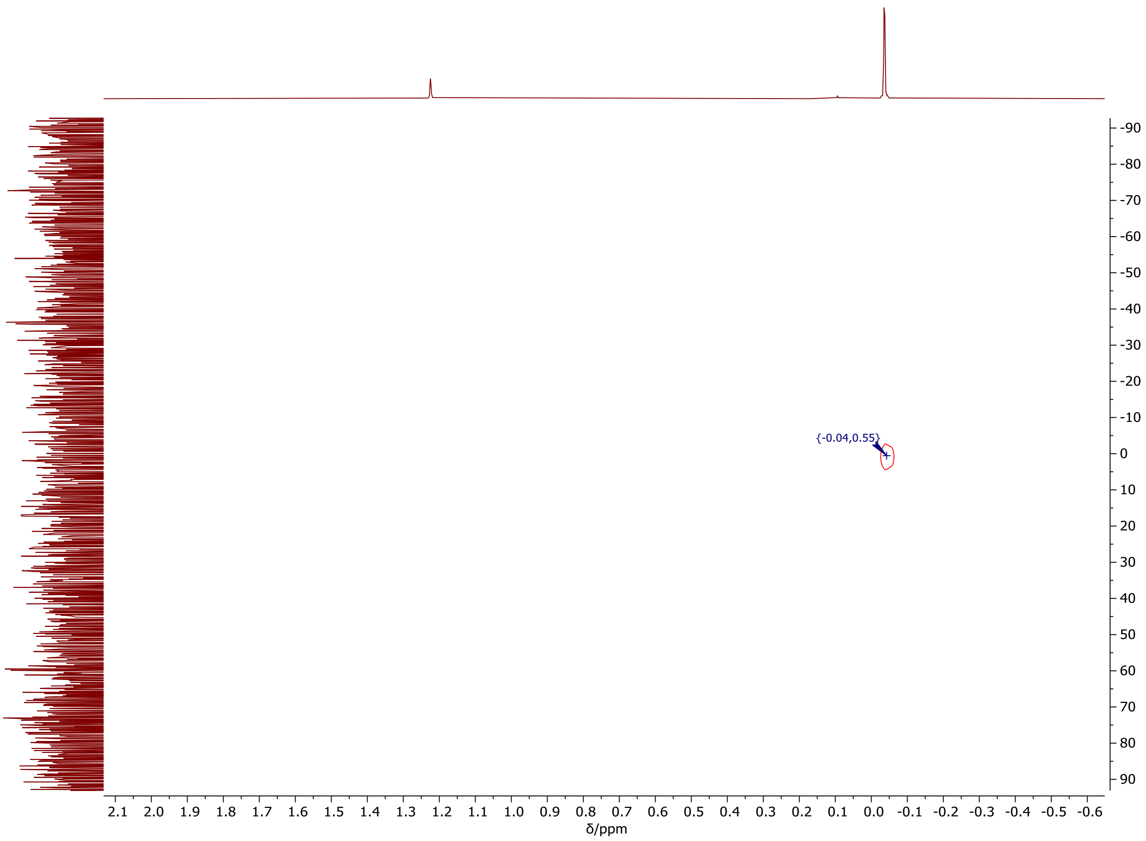

^{29}Si–^{1}H HMBC NMR spectra of diphenyl((trimethylsilyl)methyl)phosphane

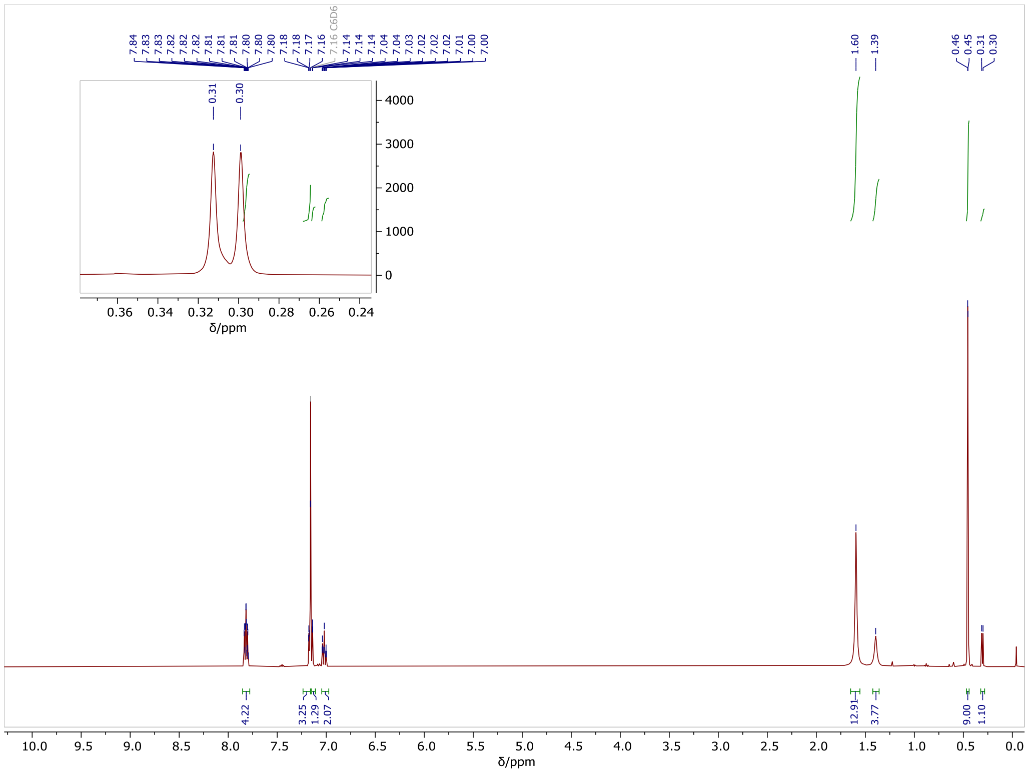

^{1}H NMR spectra of ((diphenylphosphaneyl)(trimethylsilyl)methyl)lithium **9·TMEDA** complex

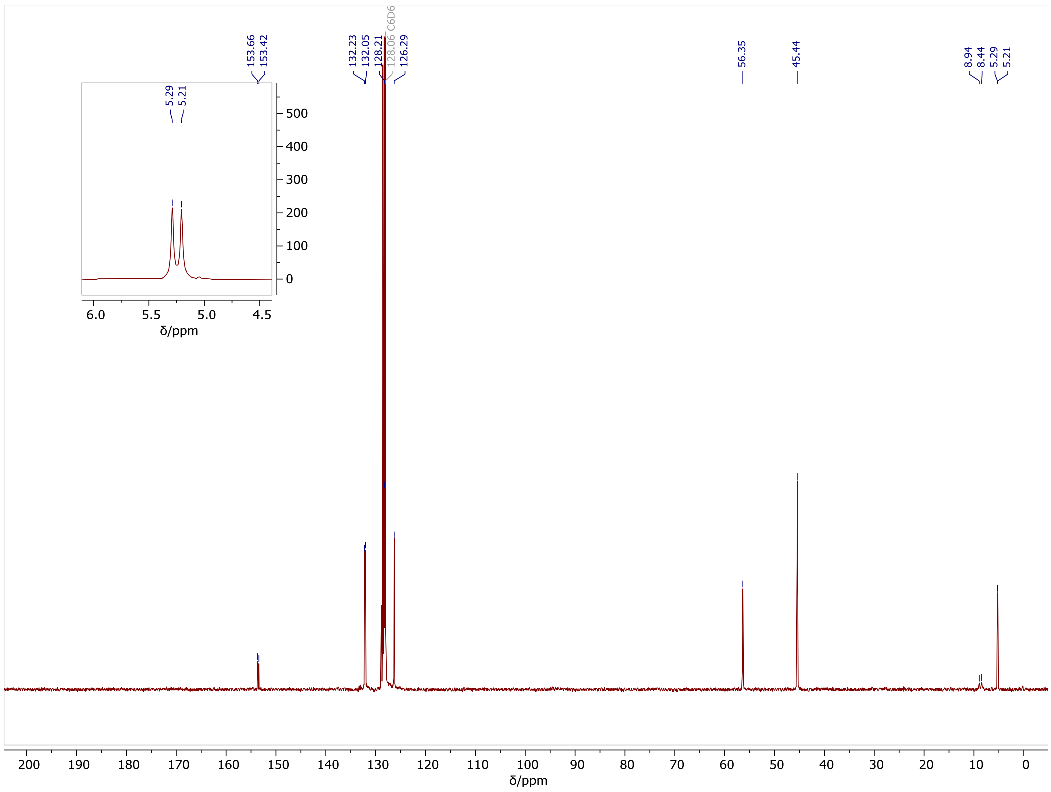

^{13}C NMR spectra of ((diphenylphosphaneyl)(trimethylsilyl)methyl)lithium **9·TMEDA** complex

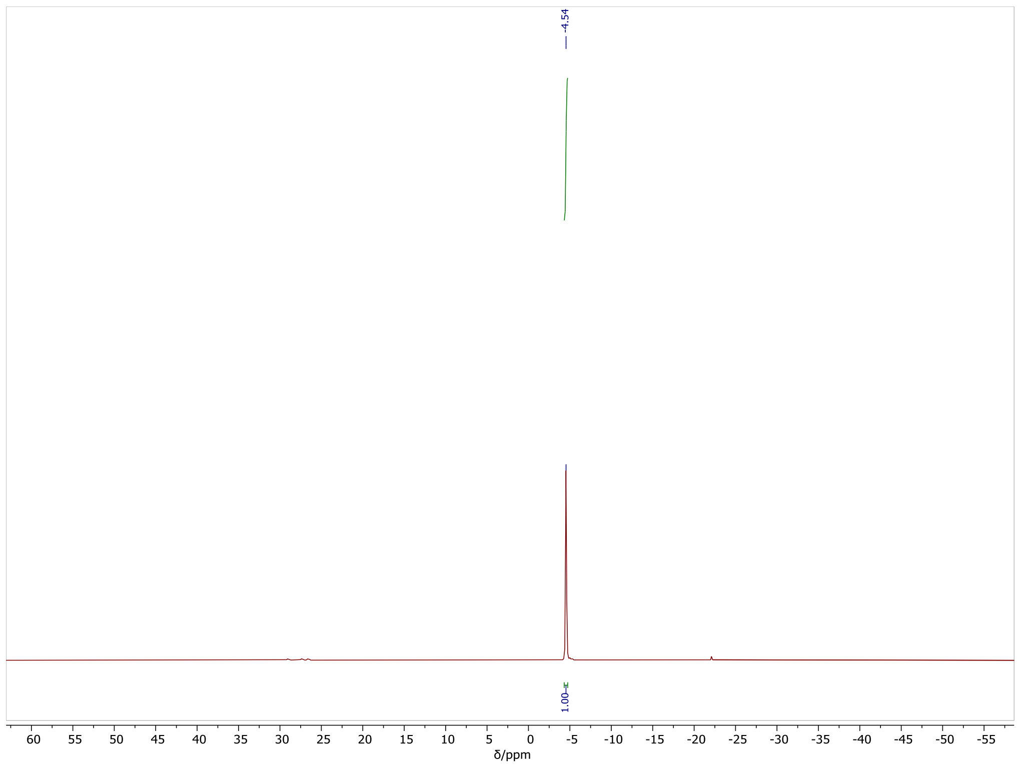

^{31}P NMR spectra of ((diphenylphosphaneyl)(trimethylsilyl)methyl)lithium **9·TMEDA** complex

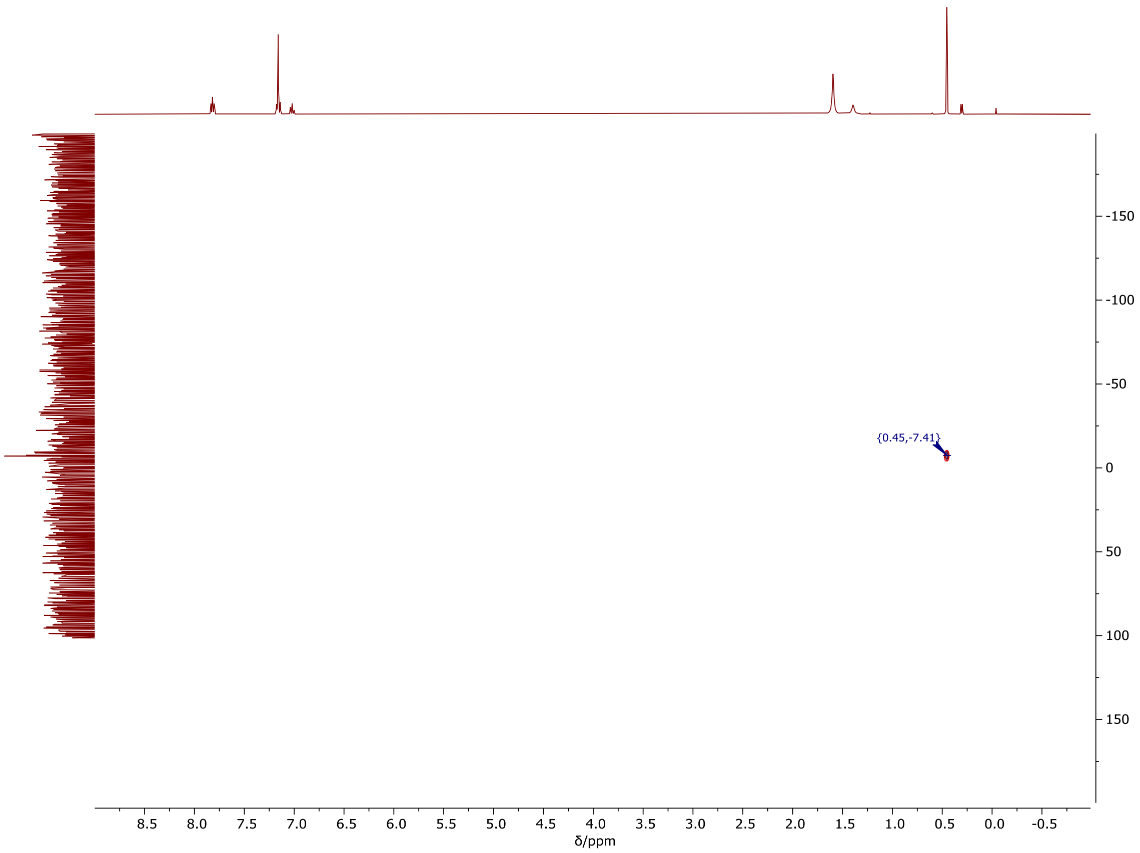

^{29}Si–^{1}H HMBC NMR spectra of ((diphenylphosphaneyl)(trimethylsilyl)methyl)lithium **9·TMEDA** complex

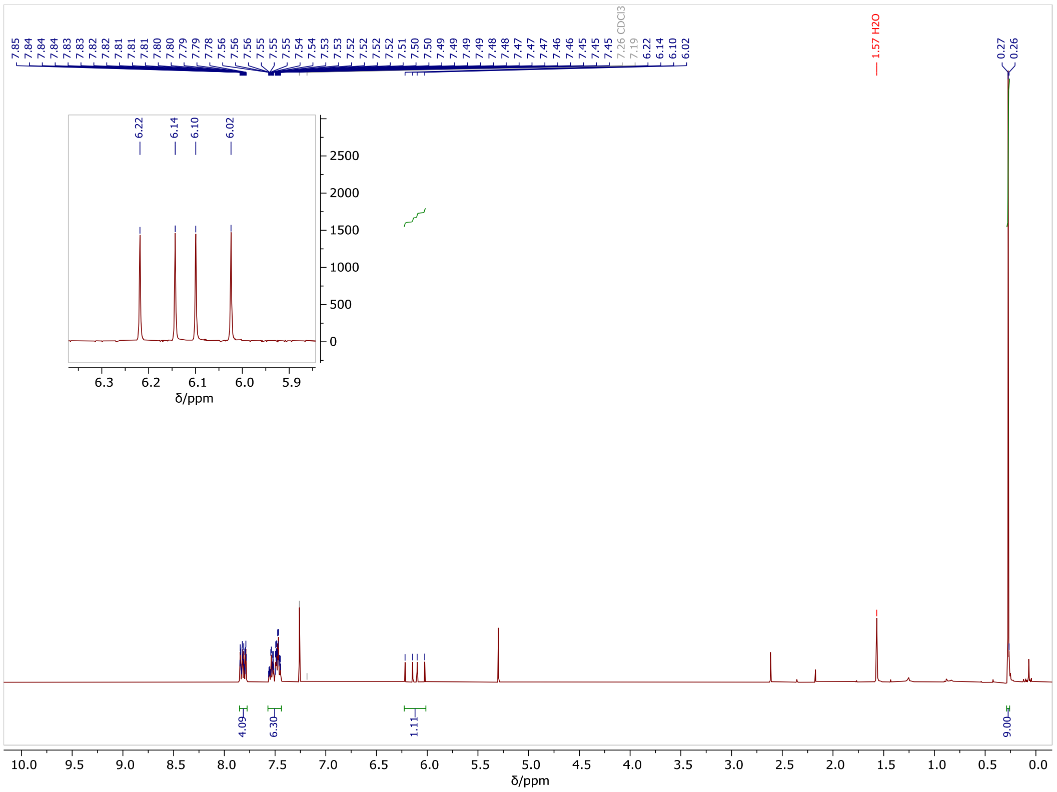

^{1}H NMR spectra of **S1**

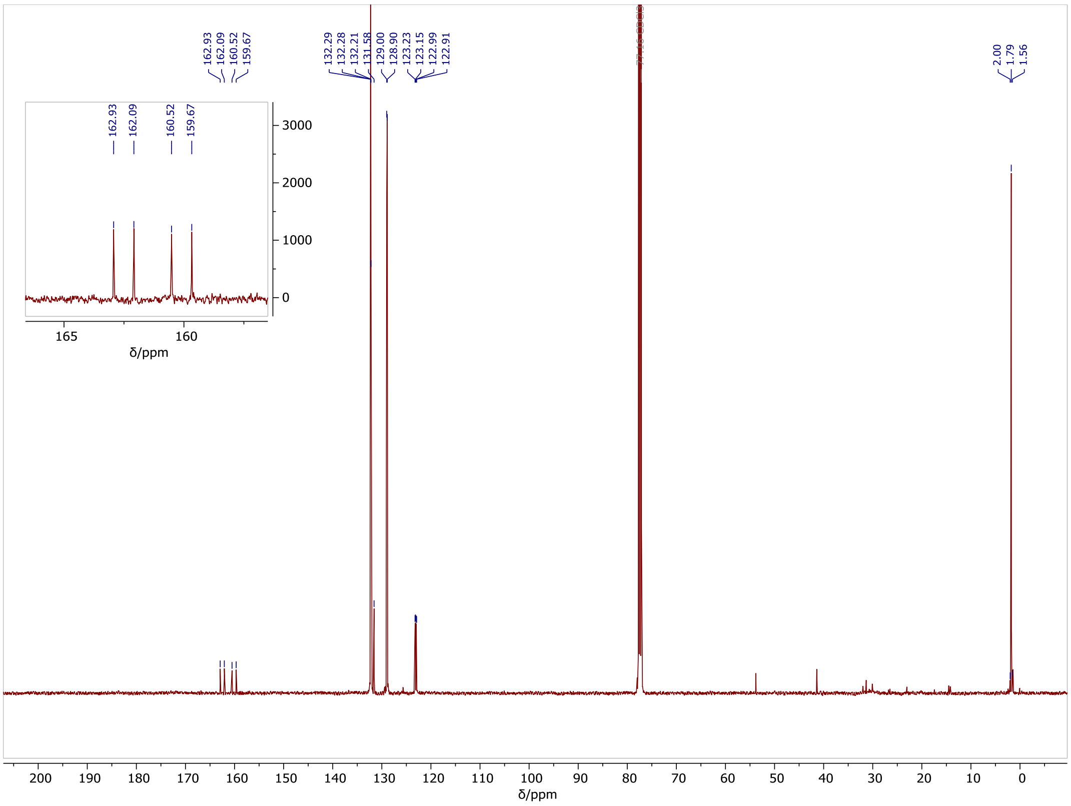

^{13}C NMR spectra of **S1**

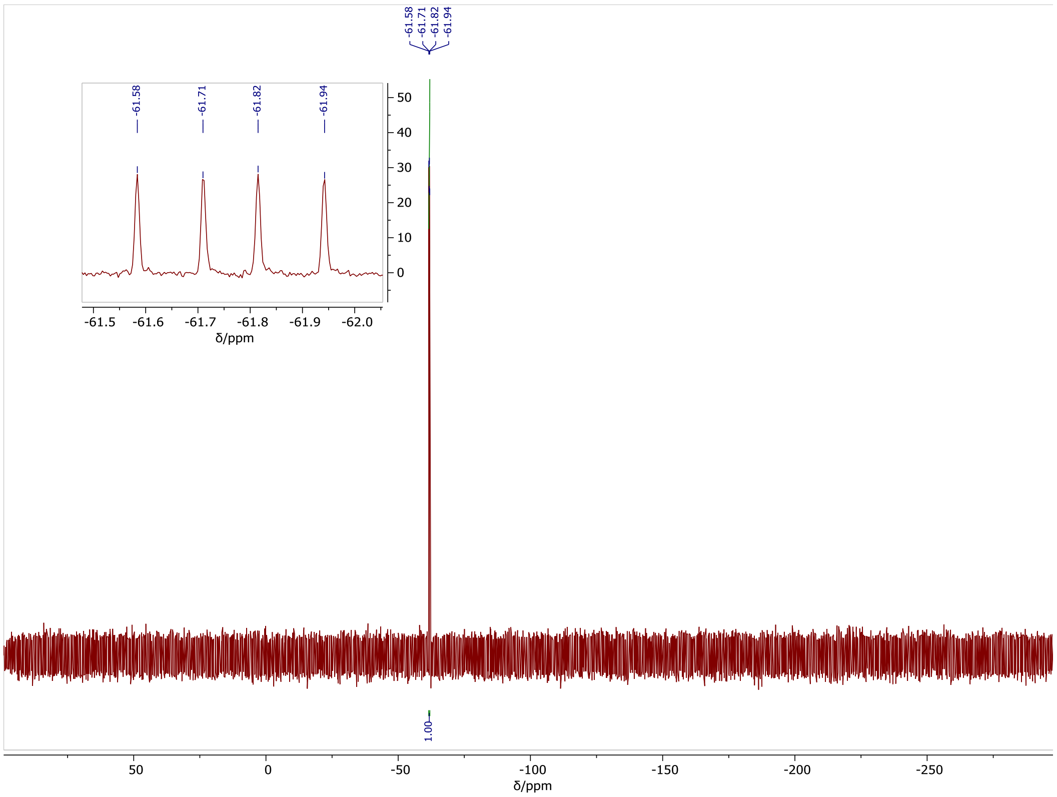

^{19}F NMR spectra of **S1**

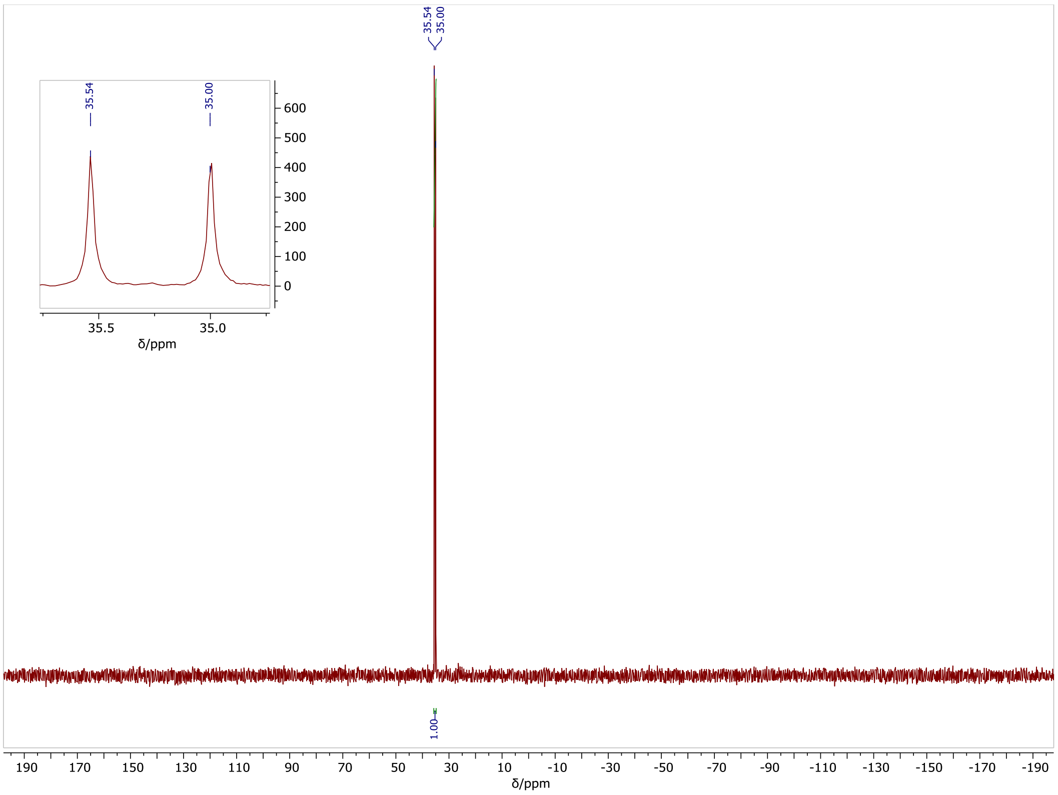

^{31}P{^{1}H} NMR spectra of **S1**

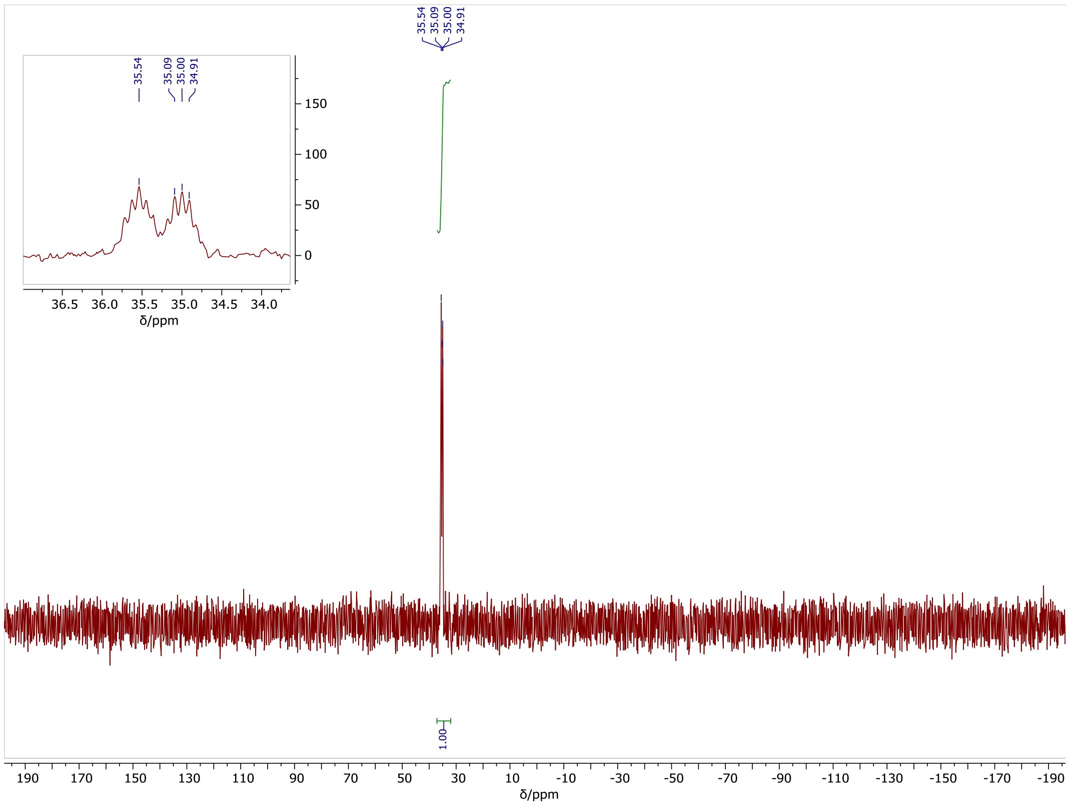

^{31}P NMR spectra of **S1**

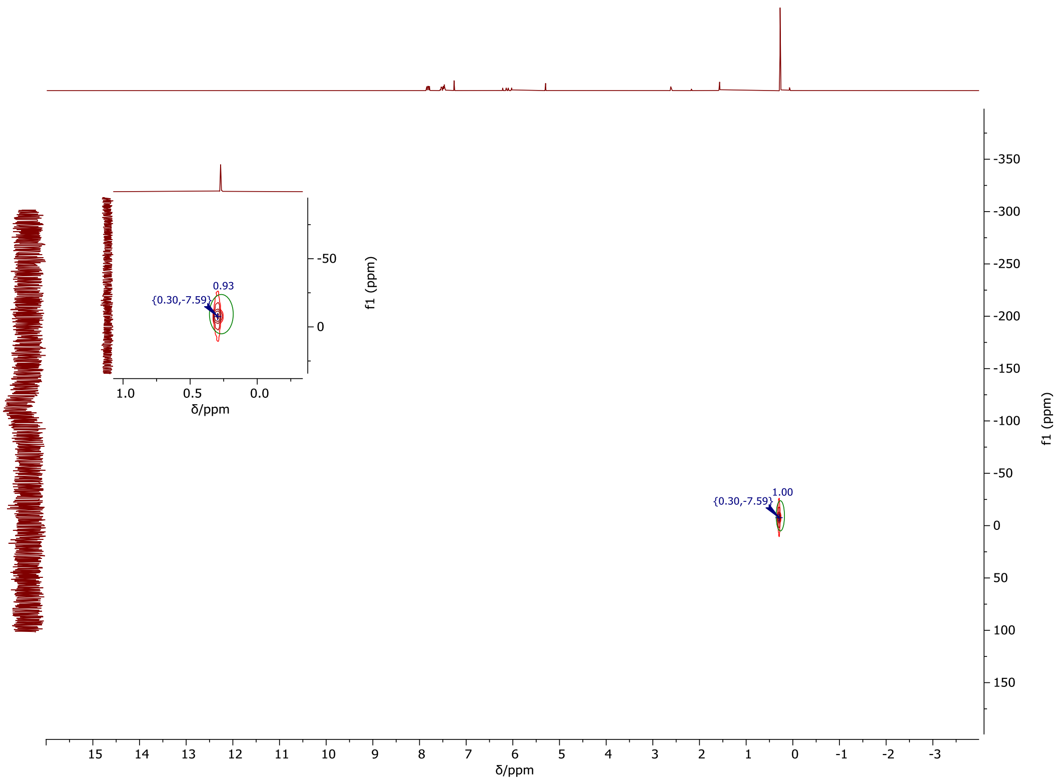

^{29}Si–^{1}H HMBC NMR spectra of **S1**

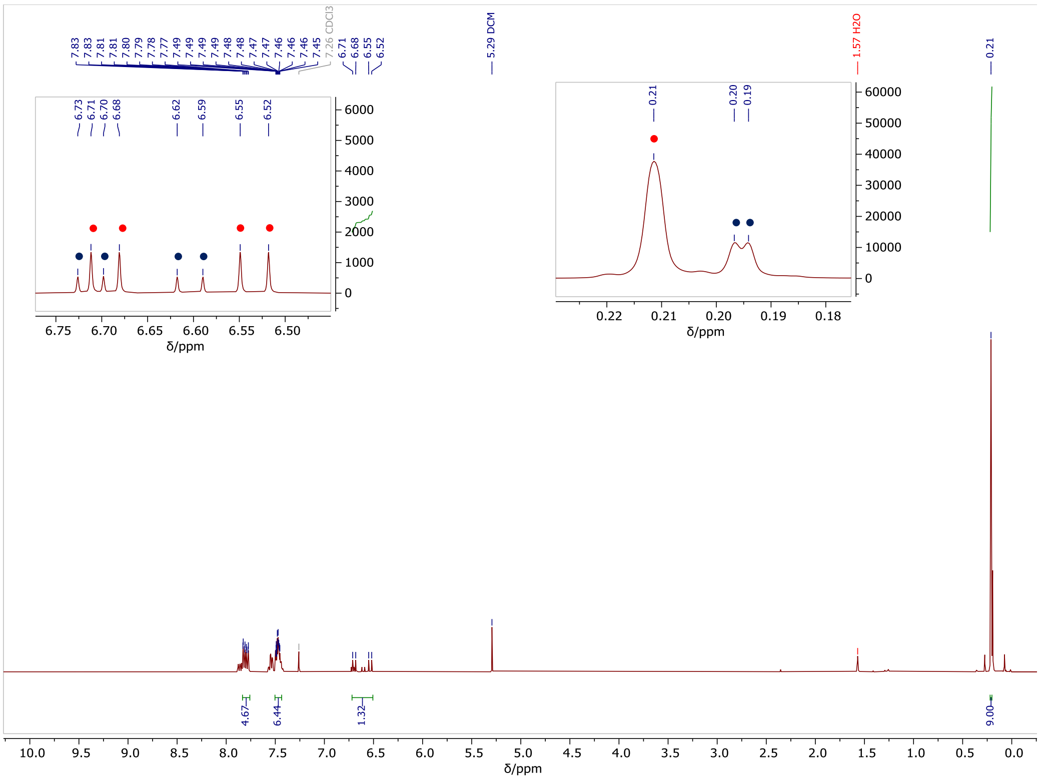

^{1}H NMR spectra of **S2 (red), S3 (blue)**

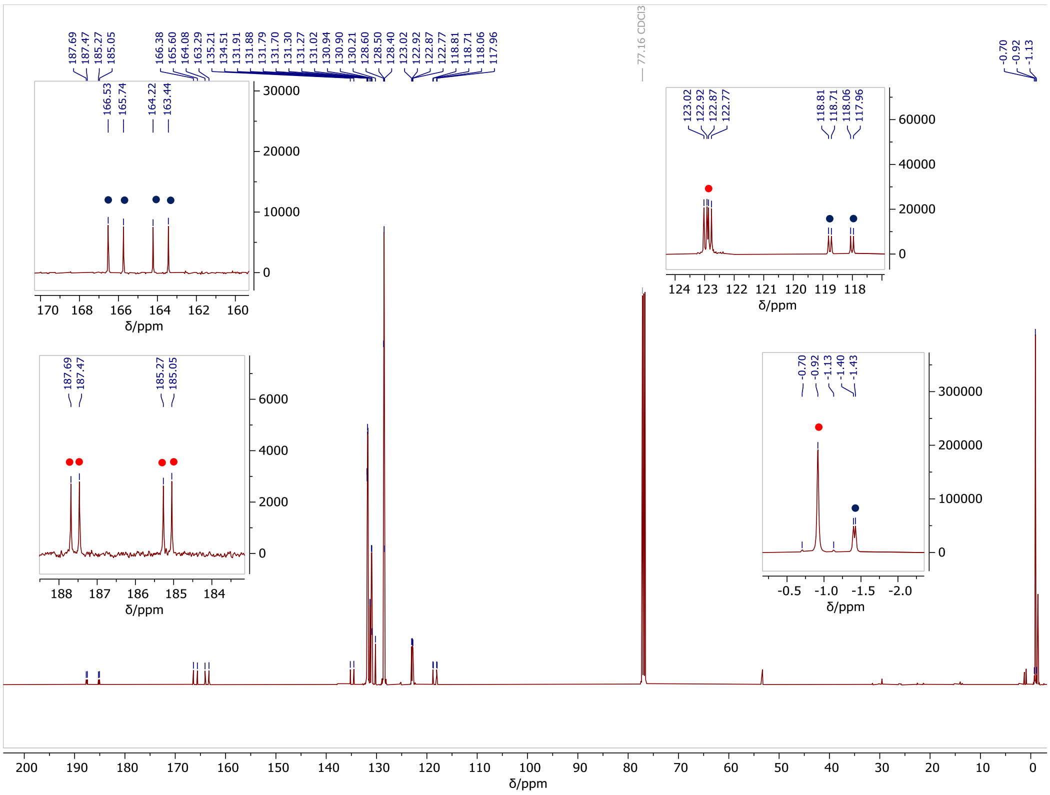

^{13}C NMR spectra of **S2 (red), S3 (blue)**

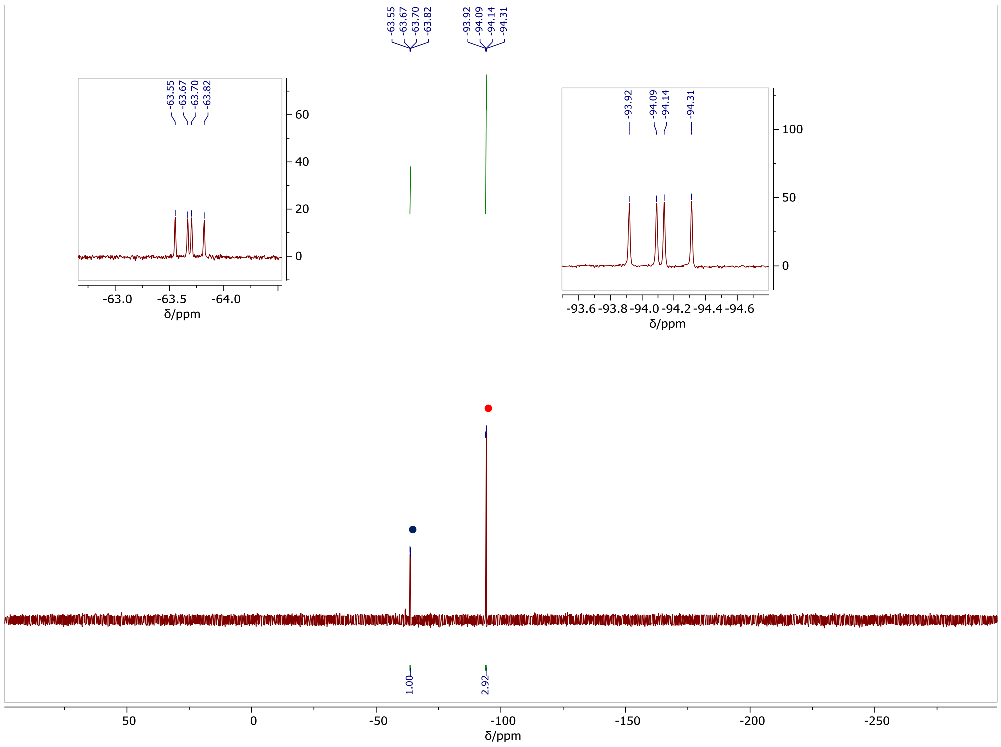

^{19}F NMR spectra of **S2 (red), S3 (blue)**

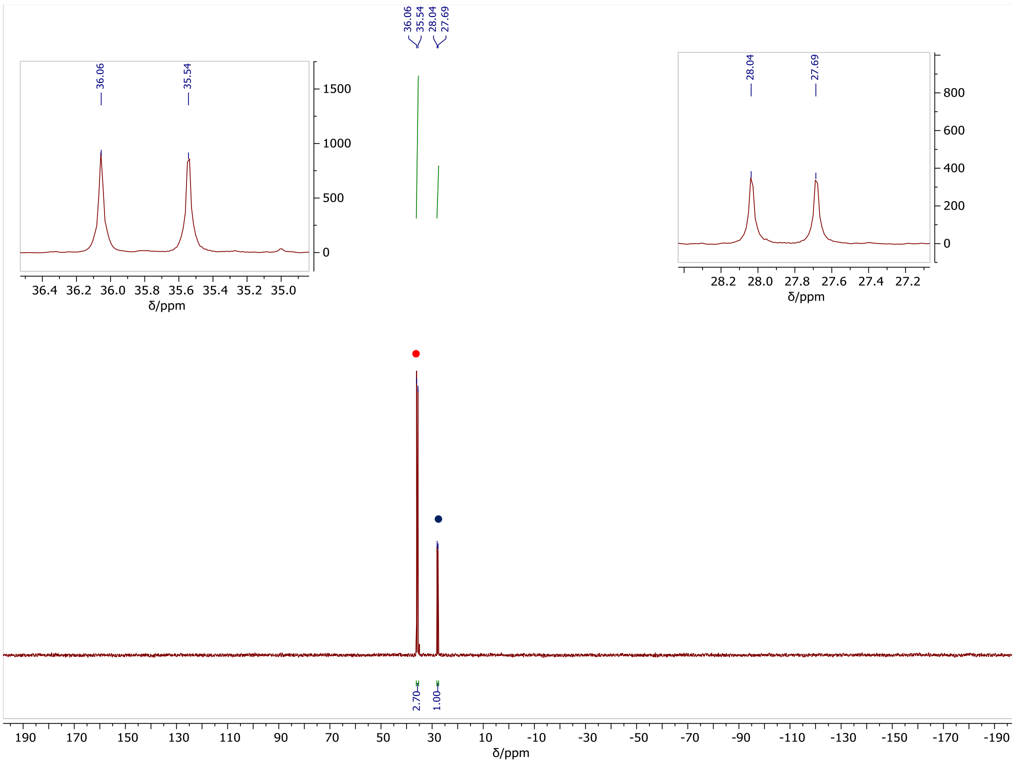

^{31}P{^{1}H} NMR spectra of **S2 (red), S3 (blue)**

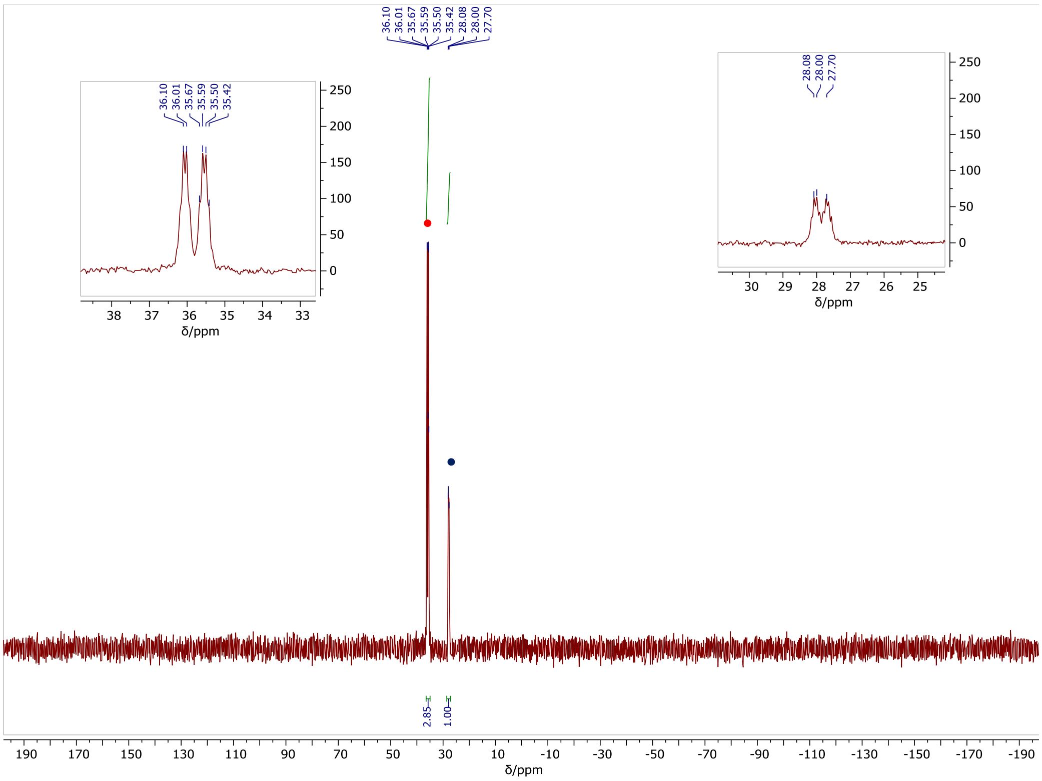

^{31}P NMR spectra of **S2 (red), S3 (blue)**

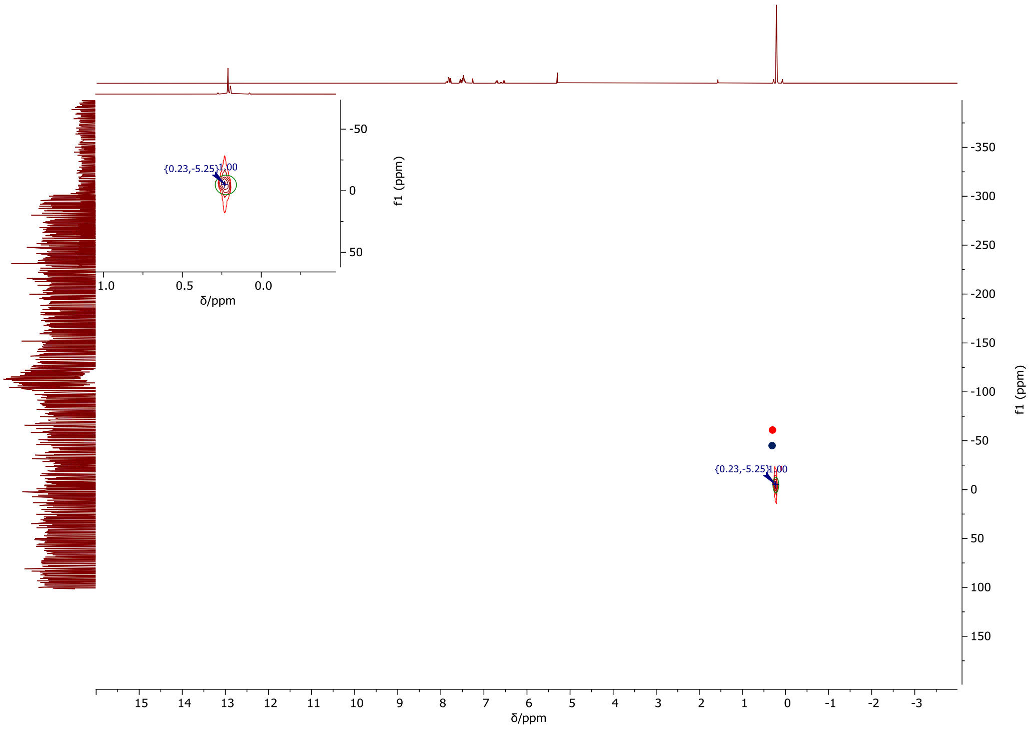

^{29}Si–^{1}H HMBC NMR spectra of, **S2 (red), S3 (blue) – overlapping**

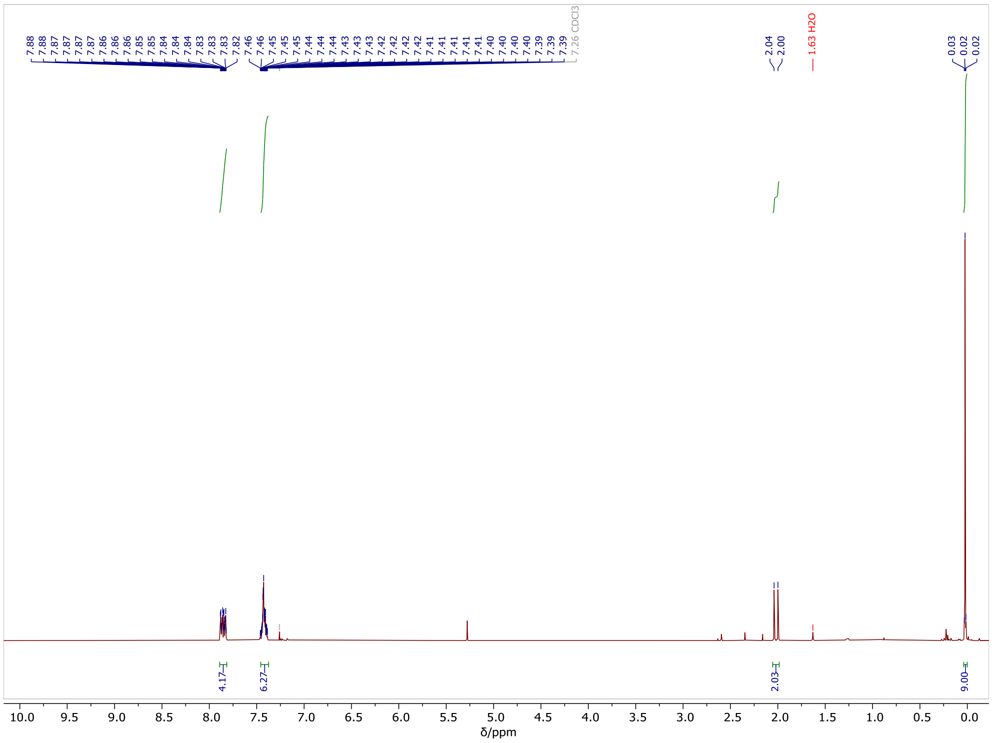

^{1}H NMR spectra of diphenyl((trimethylsilyl)methyl)phosphine sulfide, **S4**

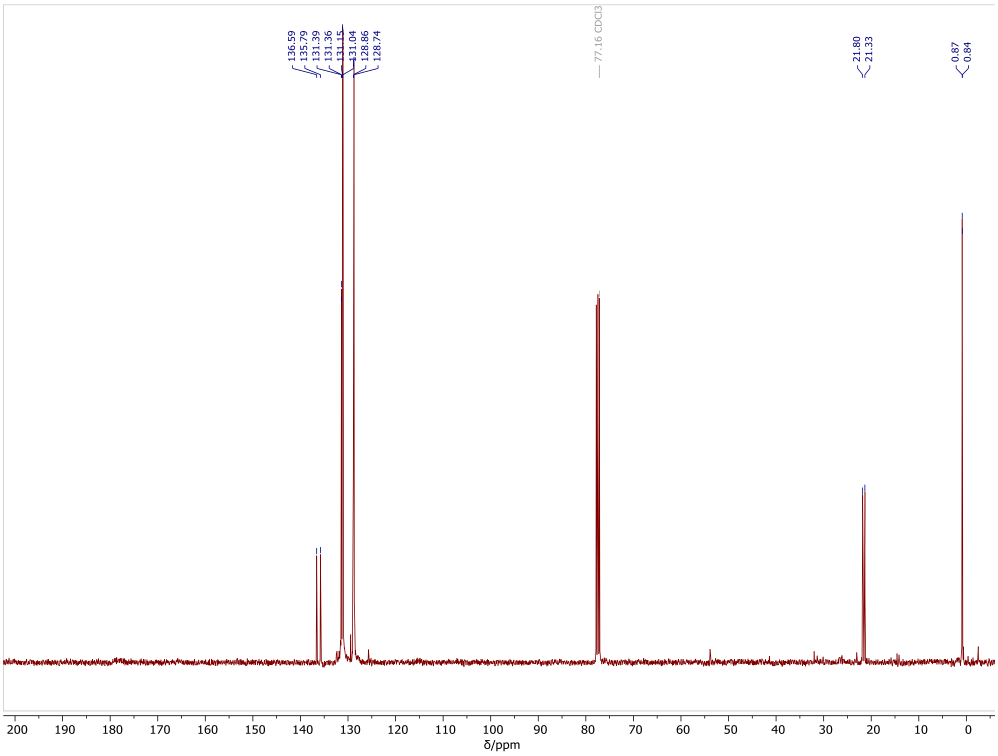

^{13}C NMR spectra of diphenyl((trimethylsilyl)methyl)phosphine sulfide, **S4**

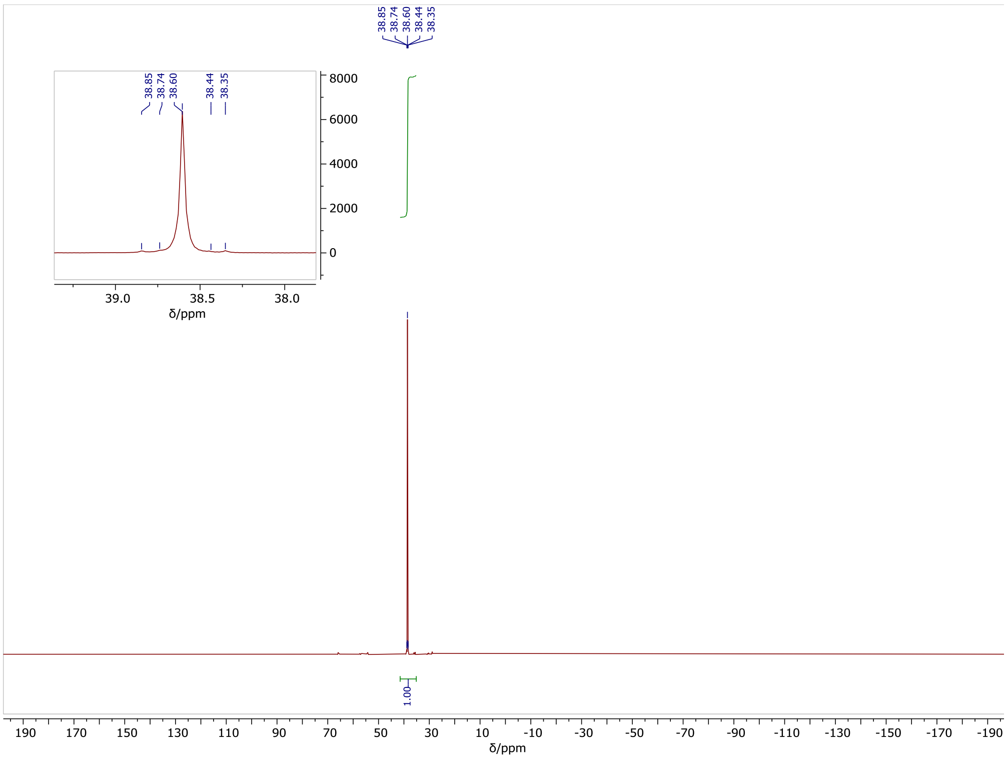

$^{31}P\{^{1}H\}$ NMR spectra of diphenyl((trimethylsilyl)methyl)phosphine sulfide, **S4**

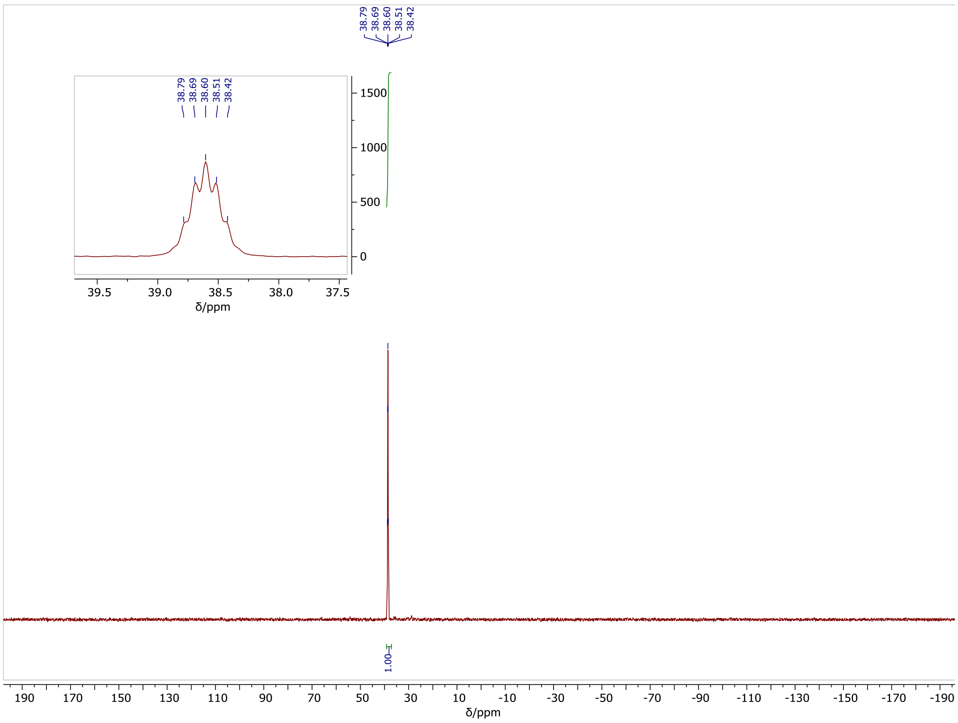

^{31}P NMR spectra of diphenyl((trimethylsilyl)methyl)phosphine sulfide, **S4**

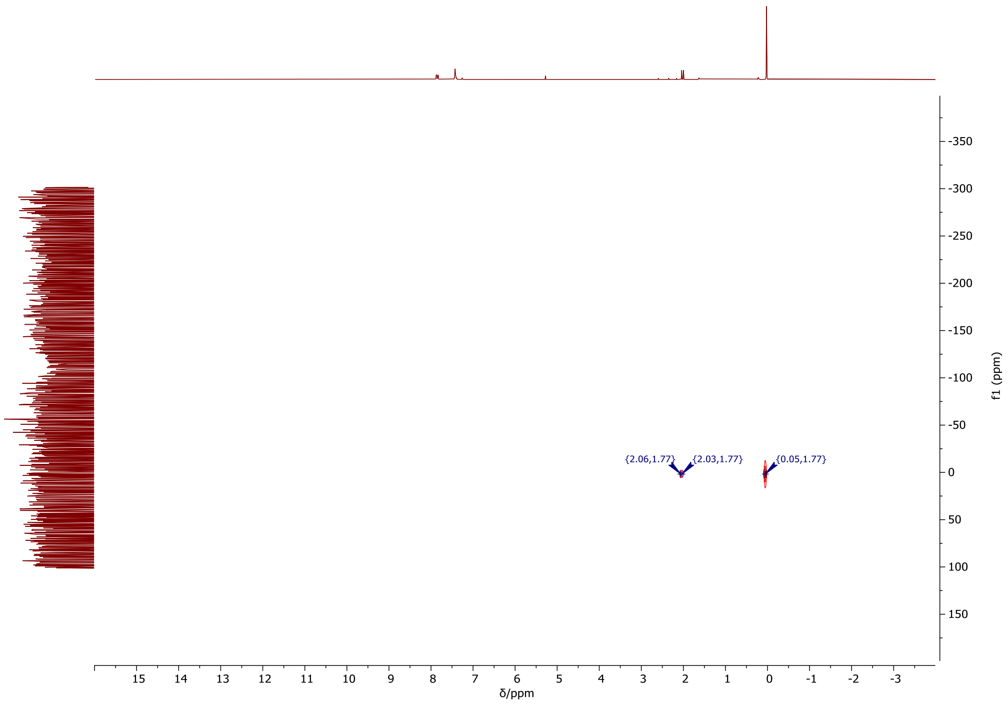

^{29}Si–^{1}H HMBC NMR spectra of diphenyl((trimethylsilyl)methyl)phosphine sulfide, **S4**

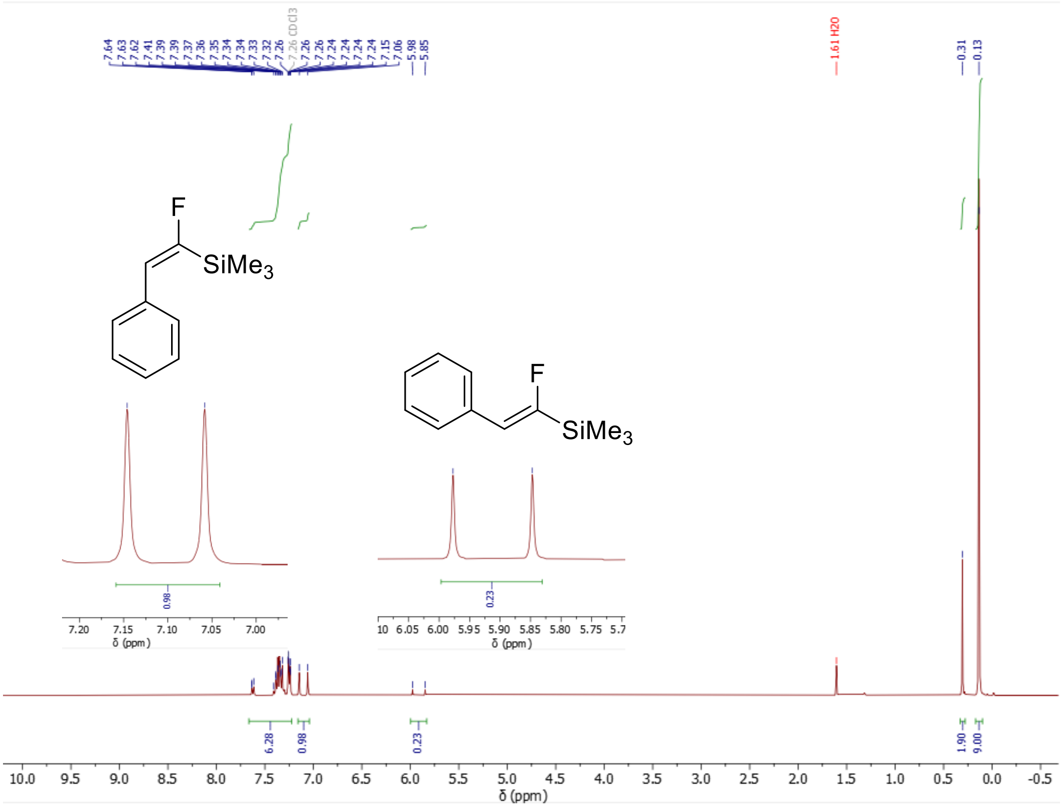

^{1}H NMR spectra of *E*/*Z*-1-fluoro-2-phenyl-1-trimethylsilylethene, ***Z/E*-7**

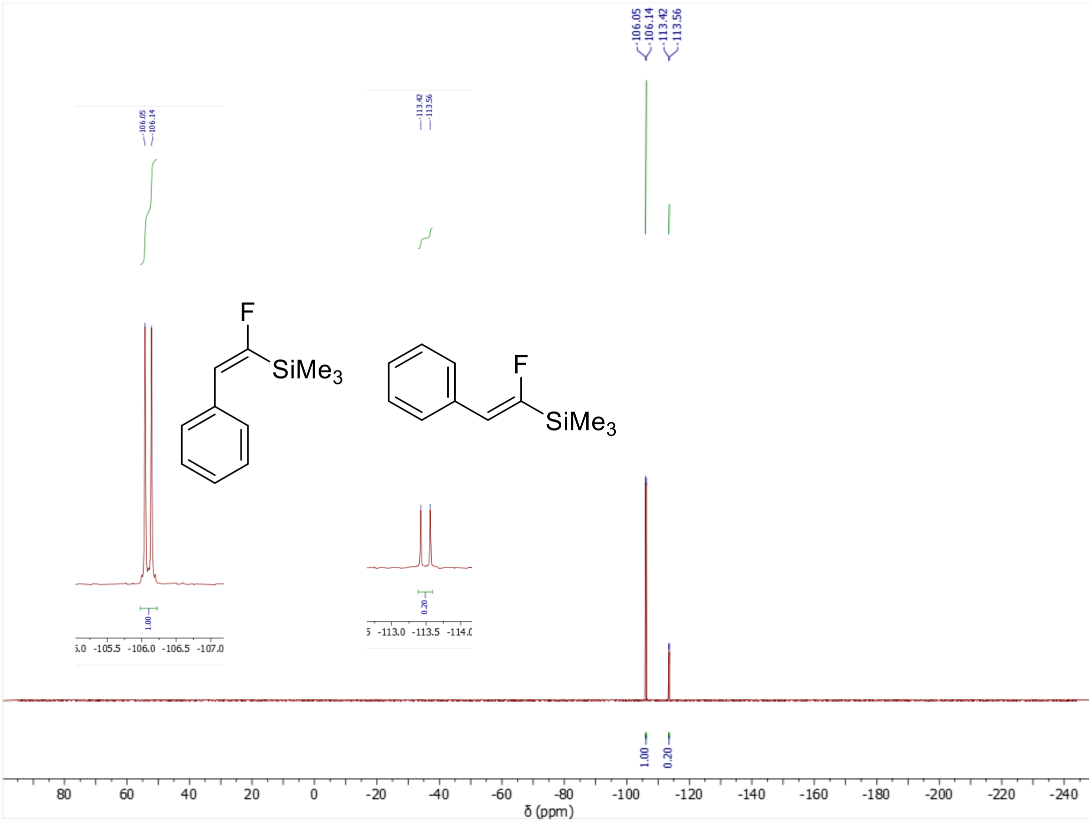

^{19}F NMR spectra of *E*/*Z*-1-fluoro-2-phenyl-1-trimethylsilylethene, ***Z/E*-7**

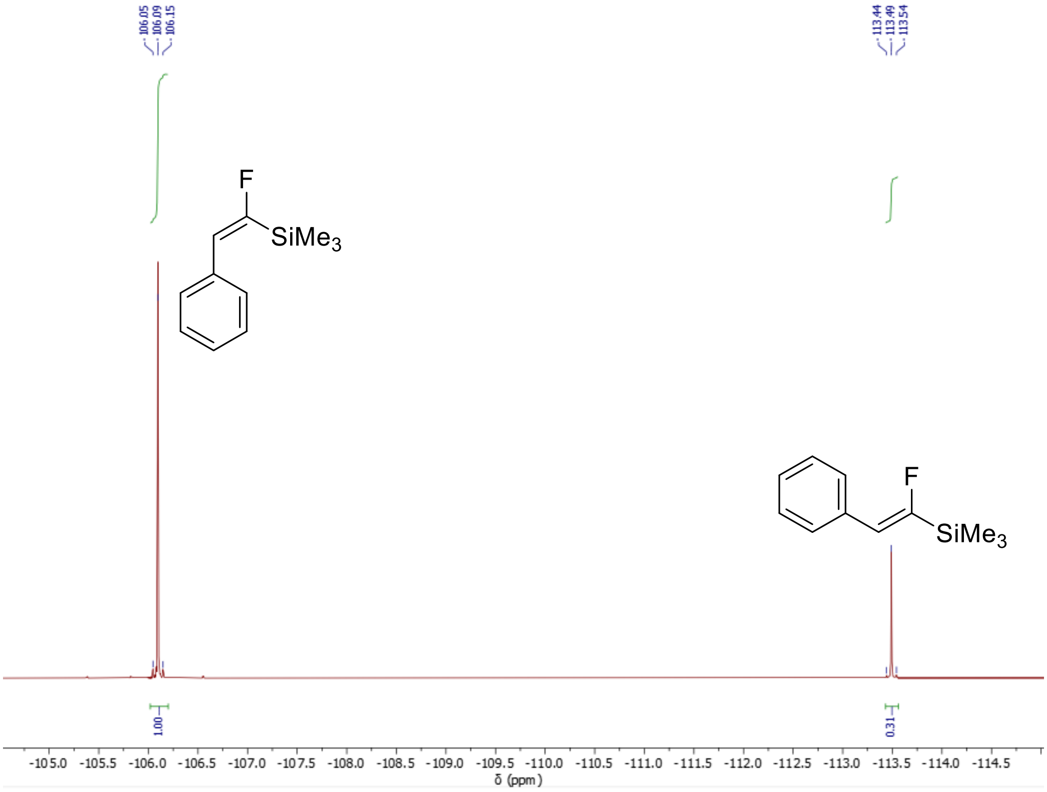

^{19}F{^{1}H} NMR spectra of *E*/*Z*-1-fluoro-2-phenyl-1-trimethylsilylethene, ***Z/E*-7**

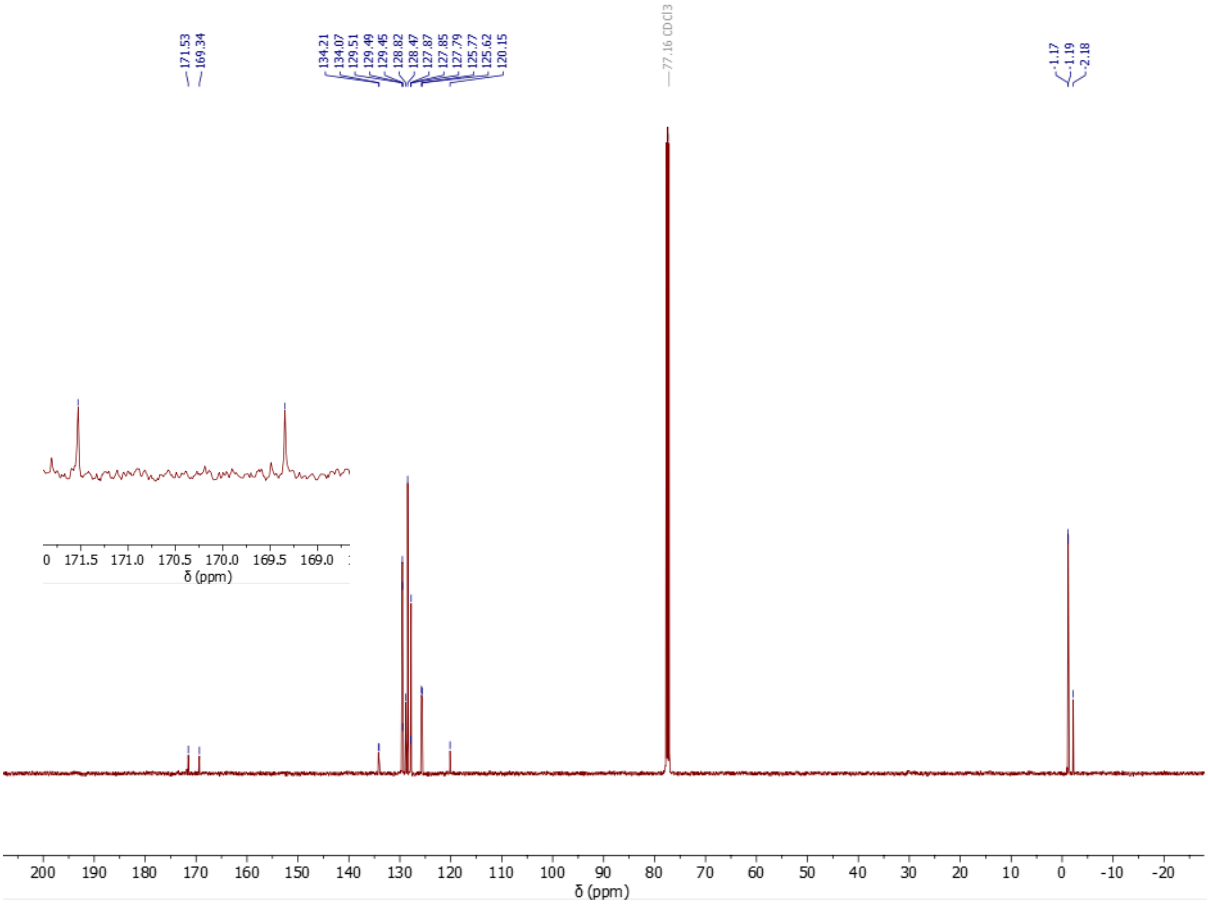

^{13}C NMR spectra of *E*/*Z*-1-fluoro-2-phenyl-1-trimethylsilylethene, ***Z/E*-7**

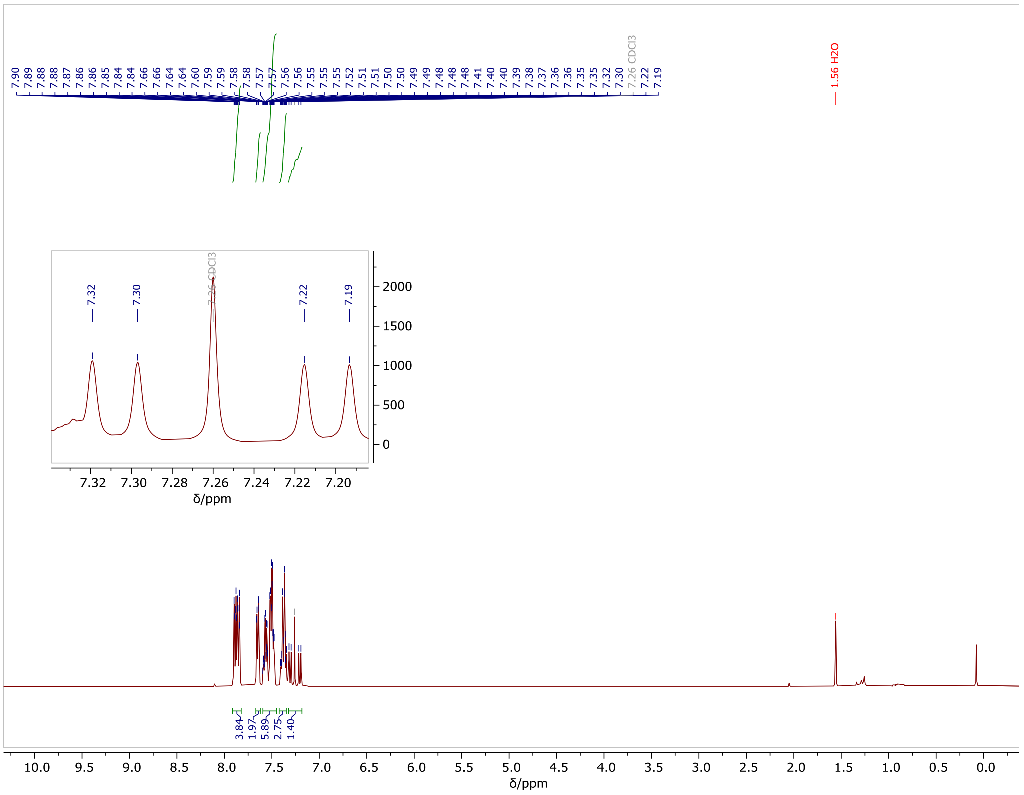

^{1}H NMR spectra of **8**

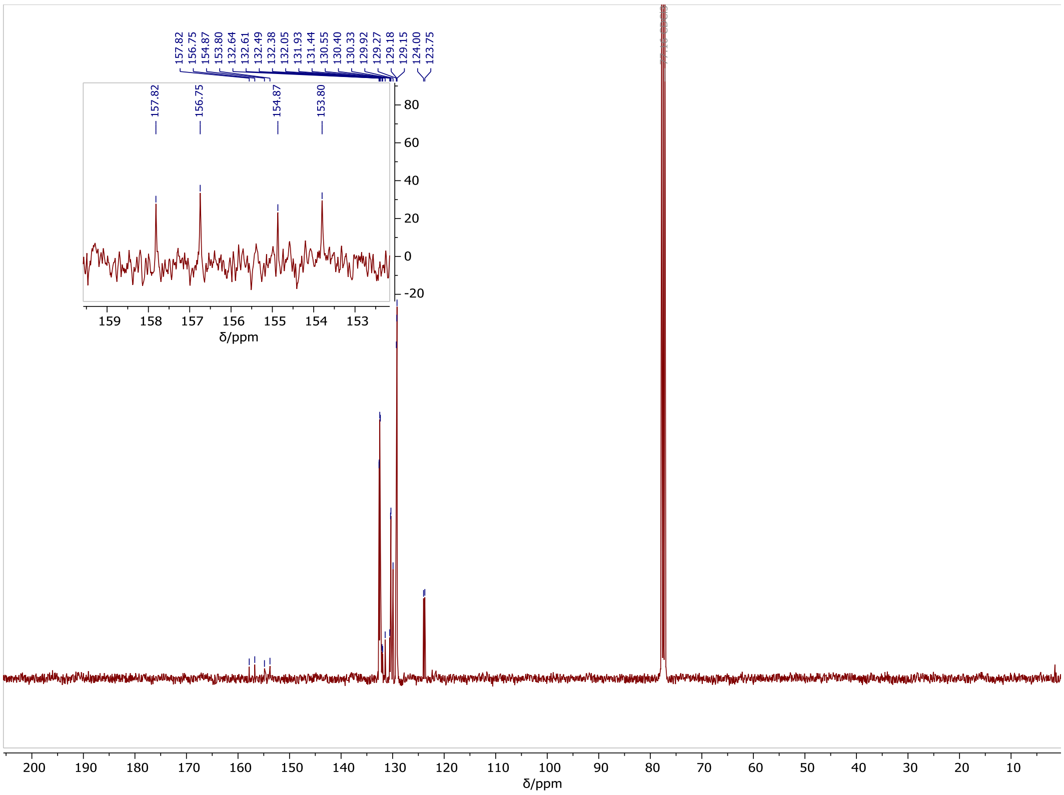

^{13}C NMR spectra of **8**

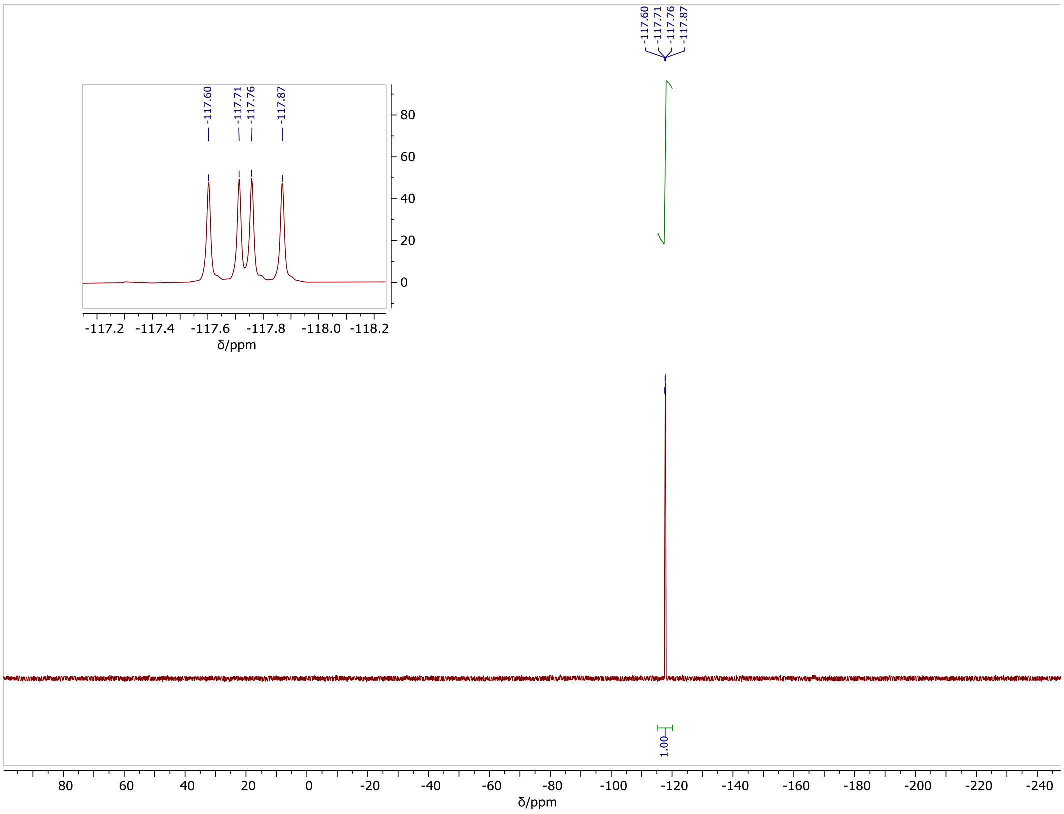

^{19}F NMR spectra of **8**

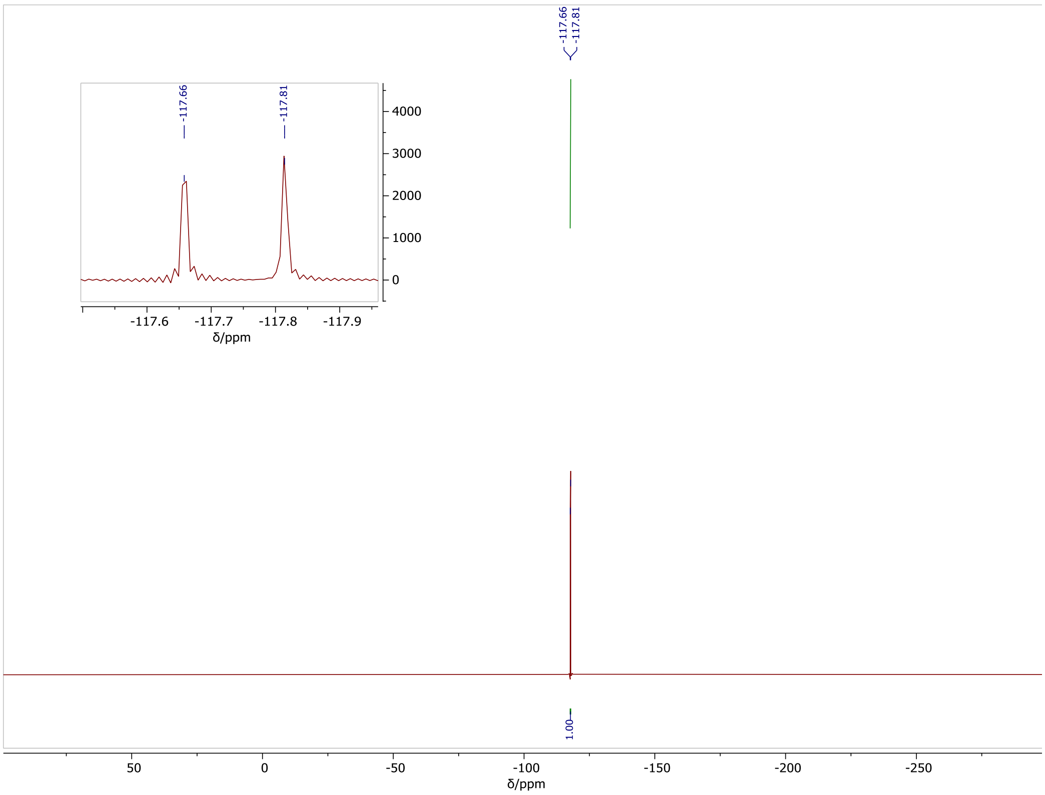

$^{19}F\{^1H\}$ NMR spectra of **8**

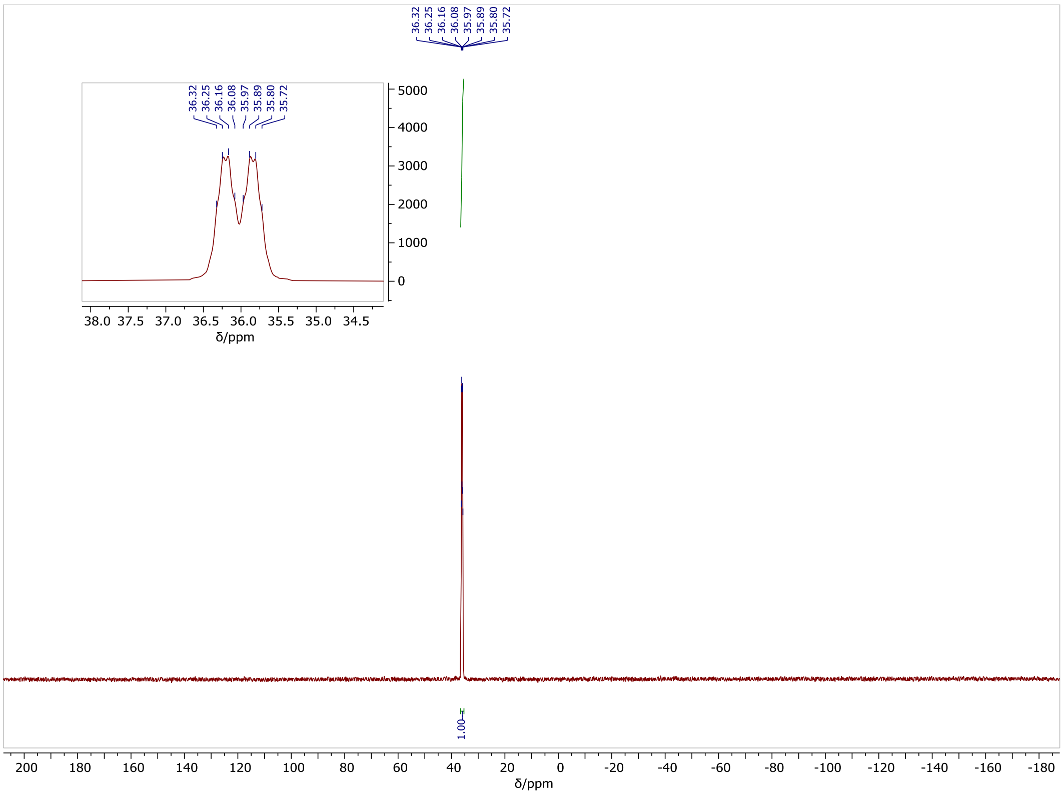

^{31}P NMR spectra of **8**

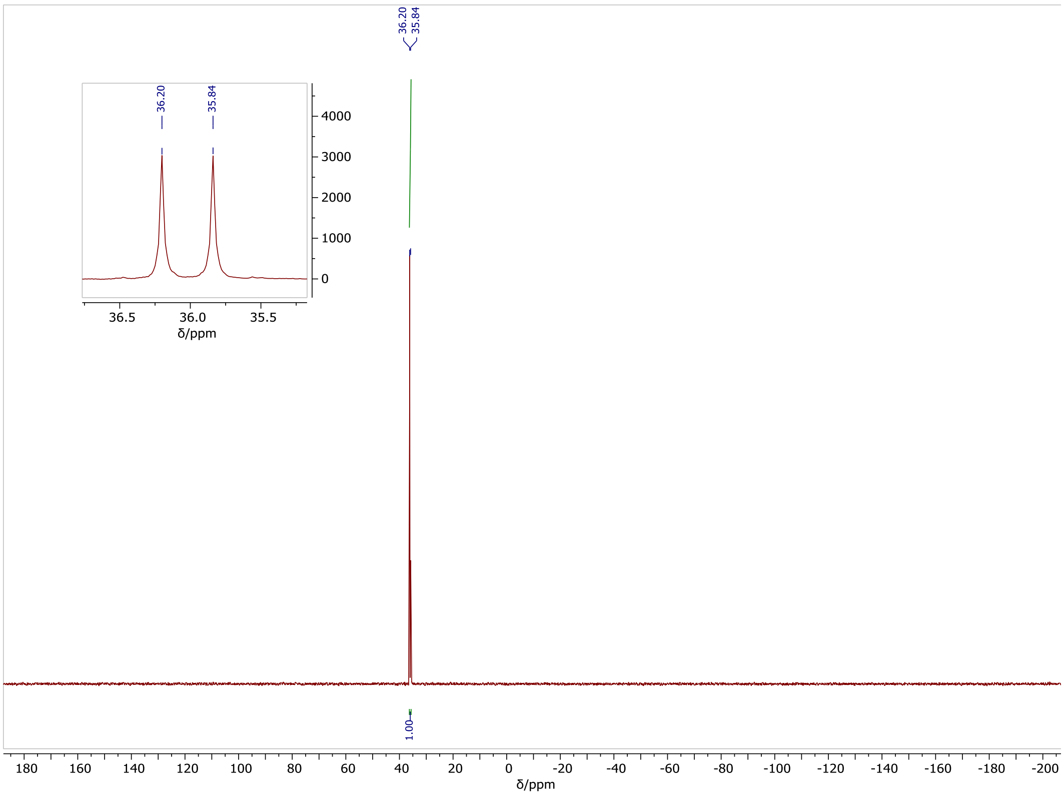

^{31}P{^{1}H} NMR spectra of **8**

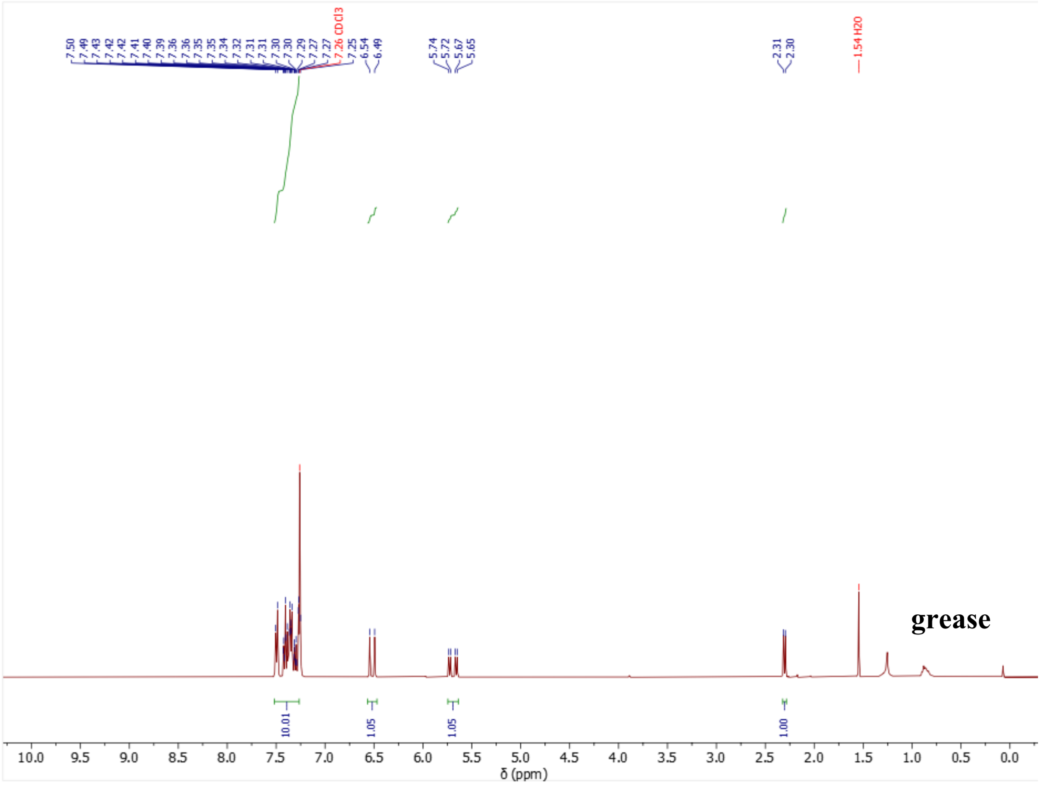

^{1}H NMR spectra of (E)-2-fluoro-1,3-diphenylprop-2-en-1-ol, **11a**

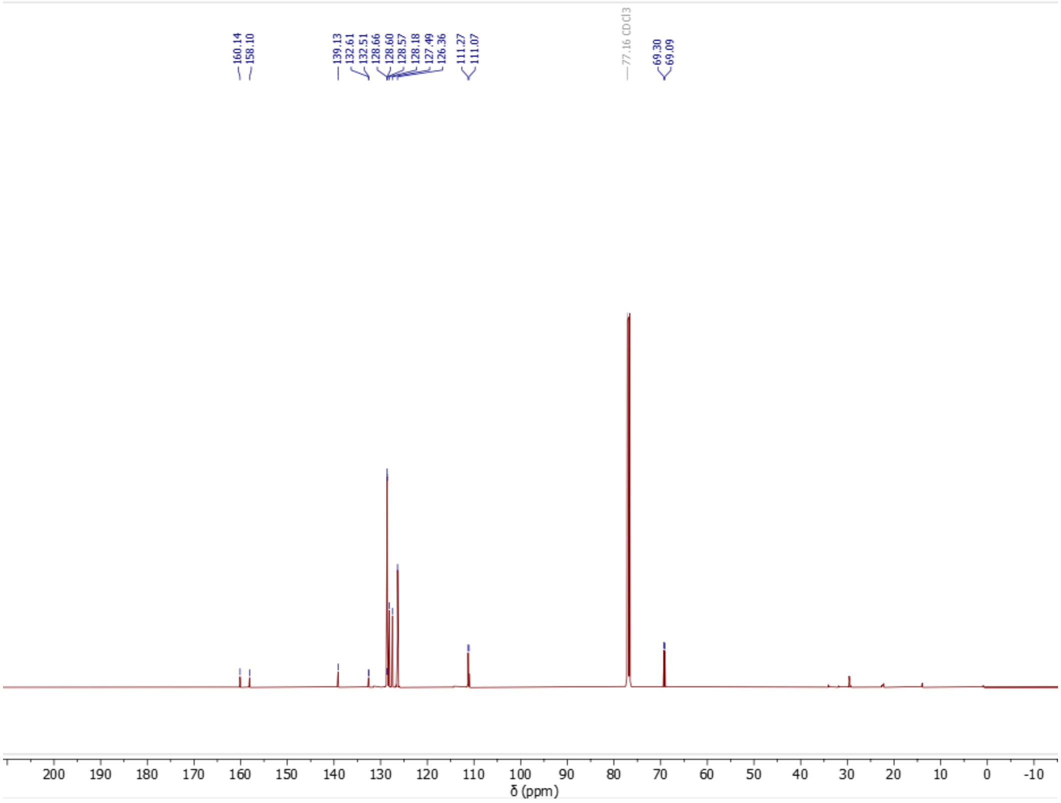

^{13}C NMR spectra of (E)-2-fluoro-1,3-diphenylprop-2-en-1-ol**, 11a**

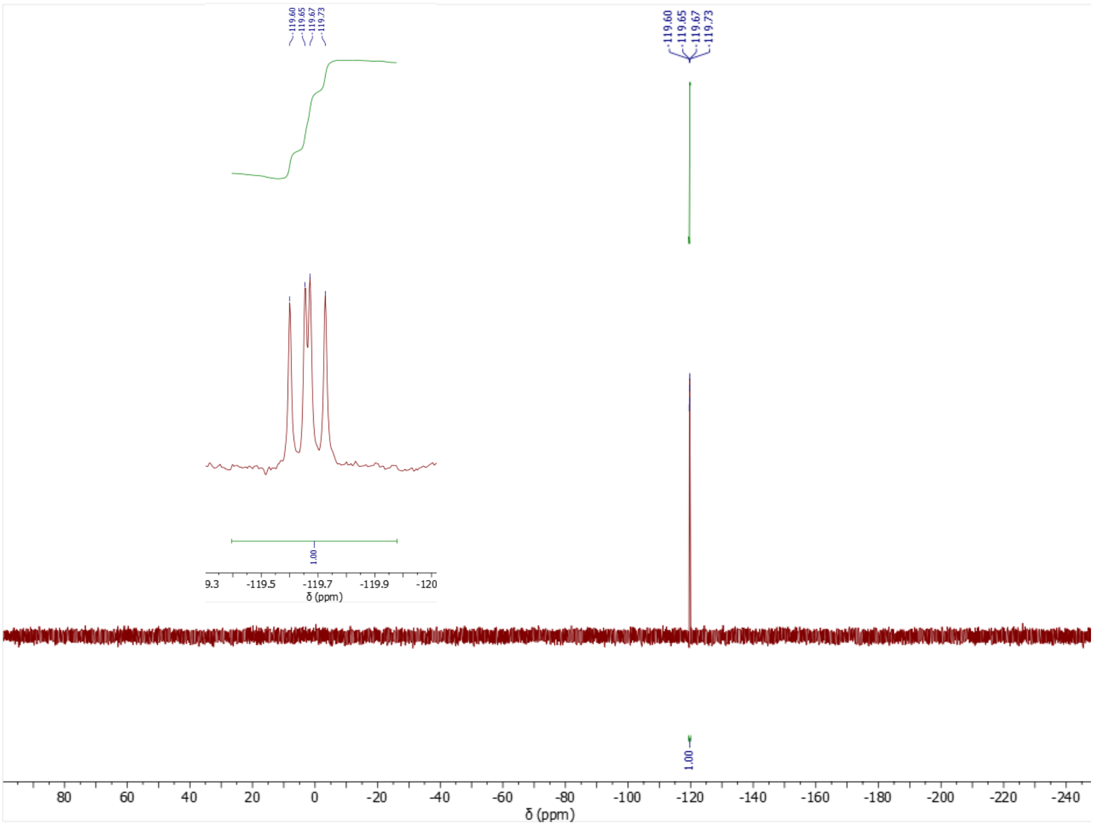

^{19}F NMR spectra of (E)-2-fluoro-1,3-diphenylprop-2-en-1-ol**, 11a**

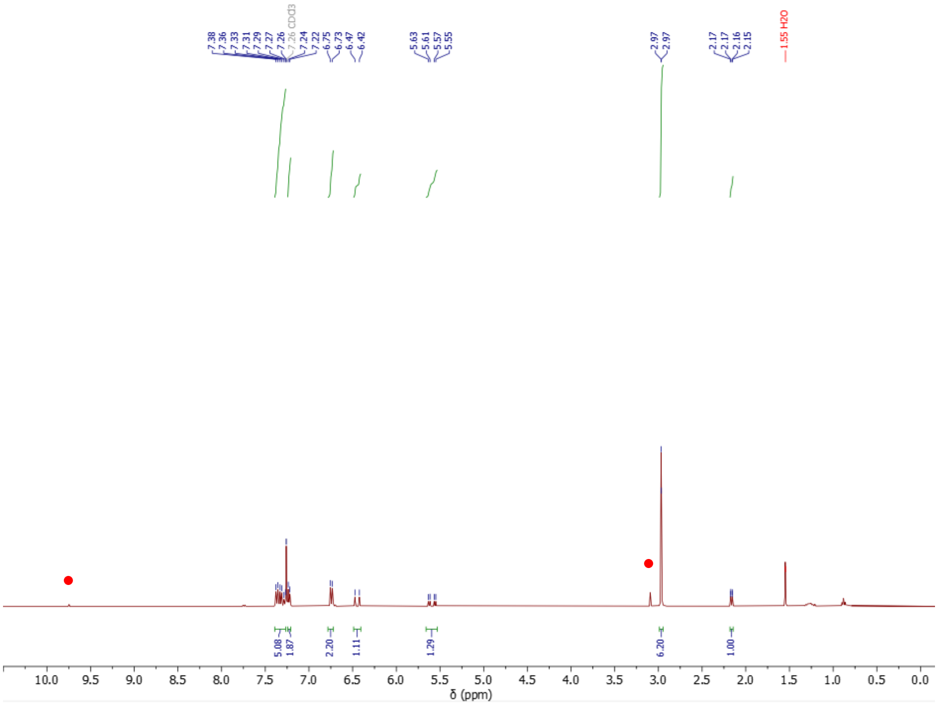

^{1}H NMR spectra of (E)-1-(4-(dimethylamino)phenyl)-2-fluoro-3-phenylprop-2-en-1-ol, **11b** (impurity 8% starting material in red)

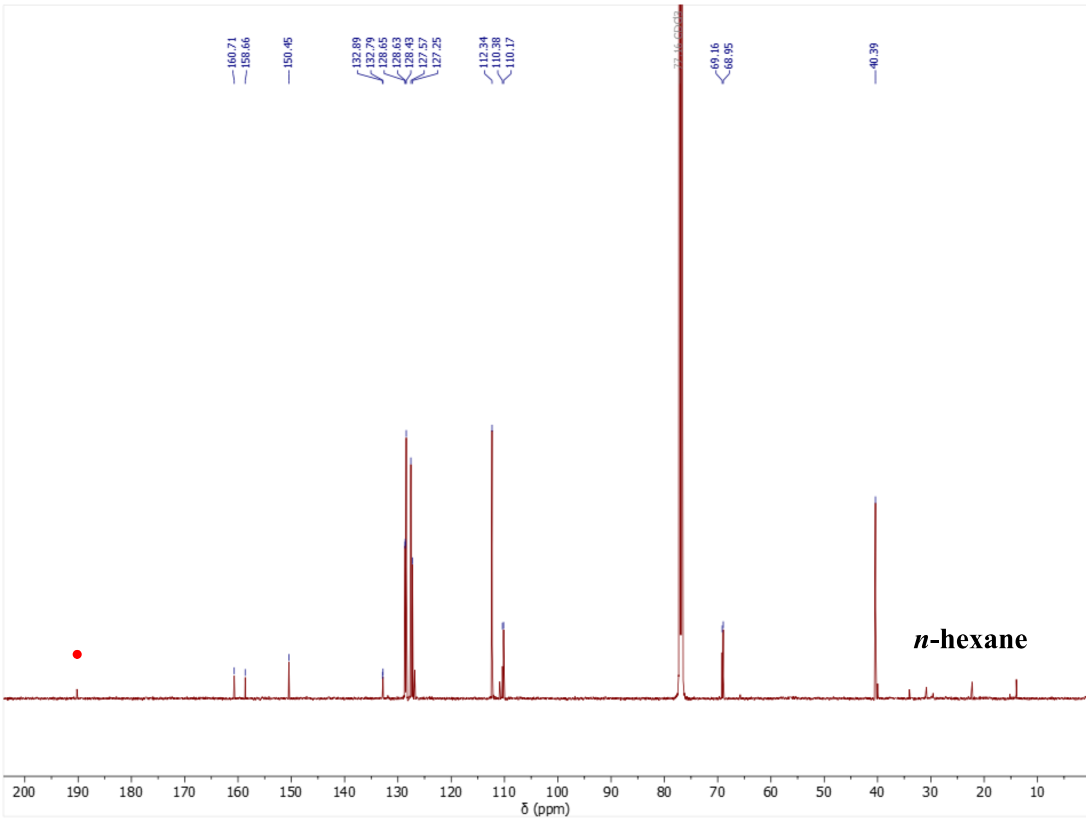

^{13}C NMR spectra of (E)-1-(4-(dimethylamino)phenyl)-2-fluoro-3-phenylprop-2-en-1-ol, **11b**
(impurity 8% starting material in red)

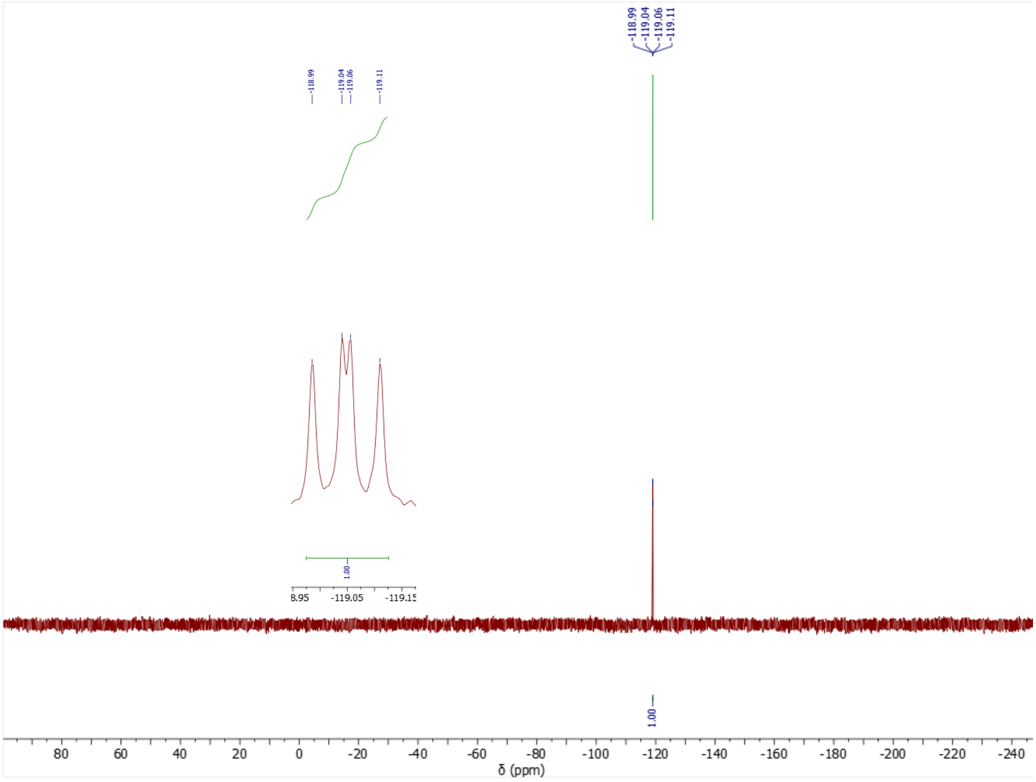

^{19}F NMR spectra of (E)-1-(4-(dimethylamino)phenyl)-2-fluoro-3-phenylprop-2-en-1-ol, **11b**

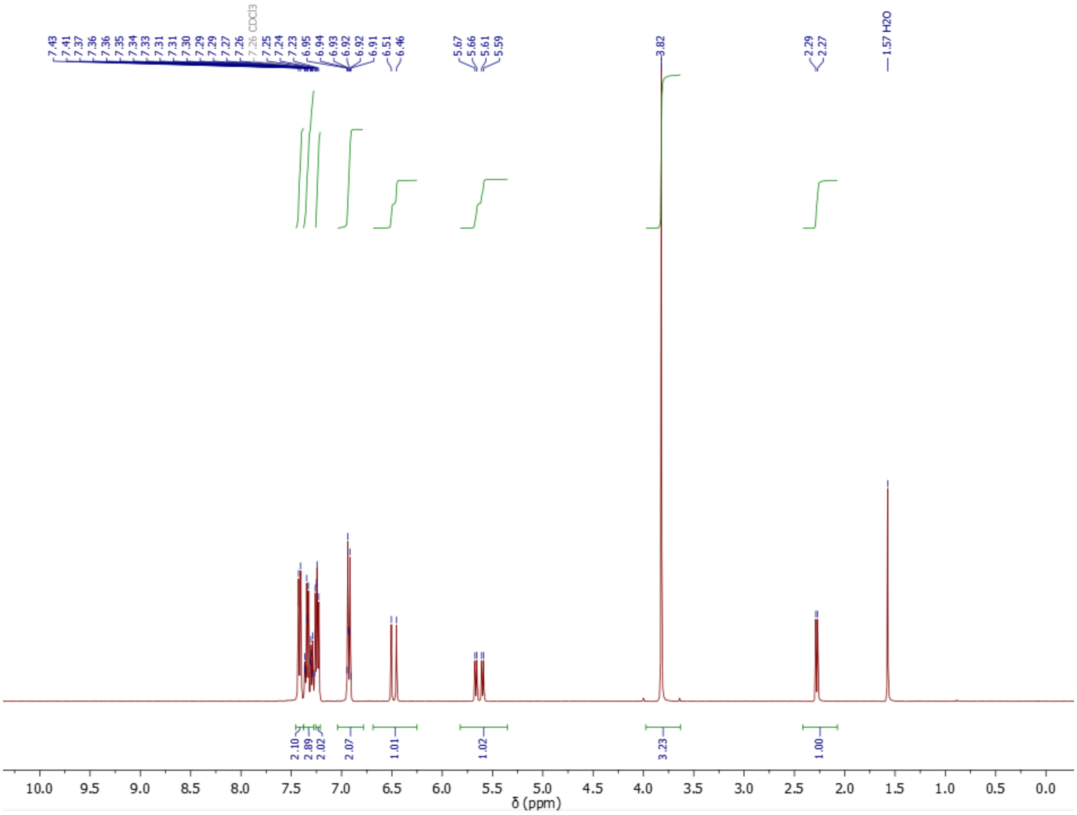

^{1}H NMR spectra of (E)-2-fluoro-1-(4-methoxyphenyl)-3-phenylprop-2-en-1-ol**, 11c**

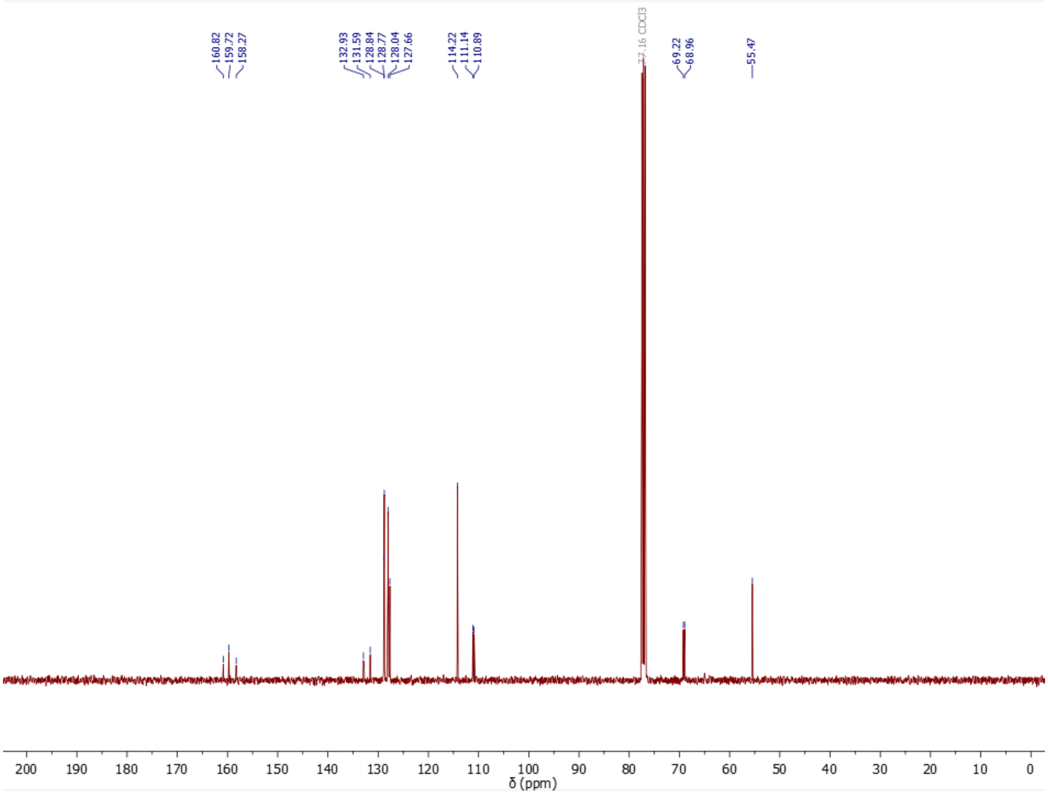

^{13}C NMR spectra of (E)-2-fluoro-1-(4-methoxyphenyl)-3-phenylprop-2-en-1-ol, **11c**

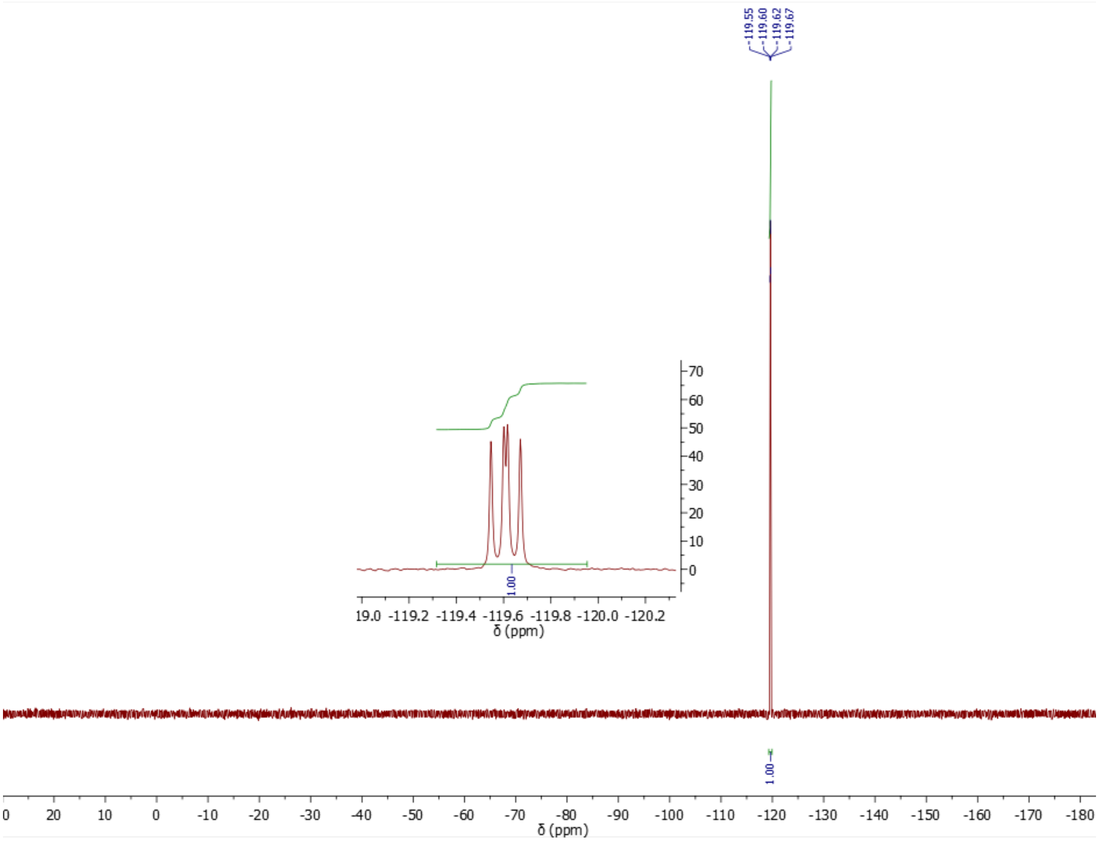

^{19}F NMR spectra of (E)-2-fluoro-1-(4-methoxyphenyl)-3-phenylprop-2-en-1-ol, **11c**

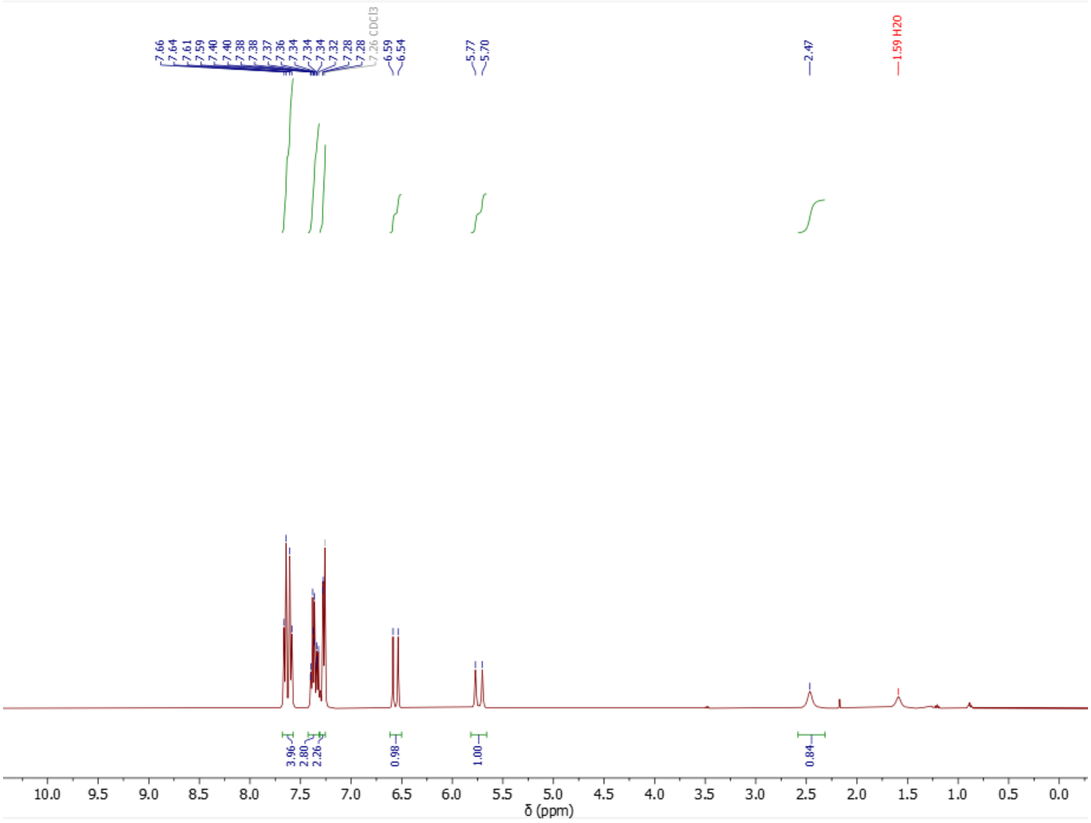

^{1}H NMR spectra of (E)-2-fluoro-3-phenyl-1-(4-(trifluoromethyl)phenyl)prop-2-en-1-ol, **11d**

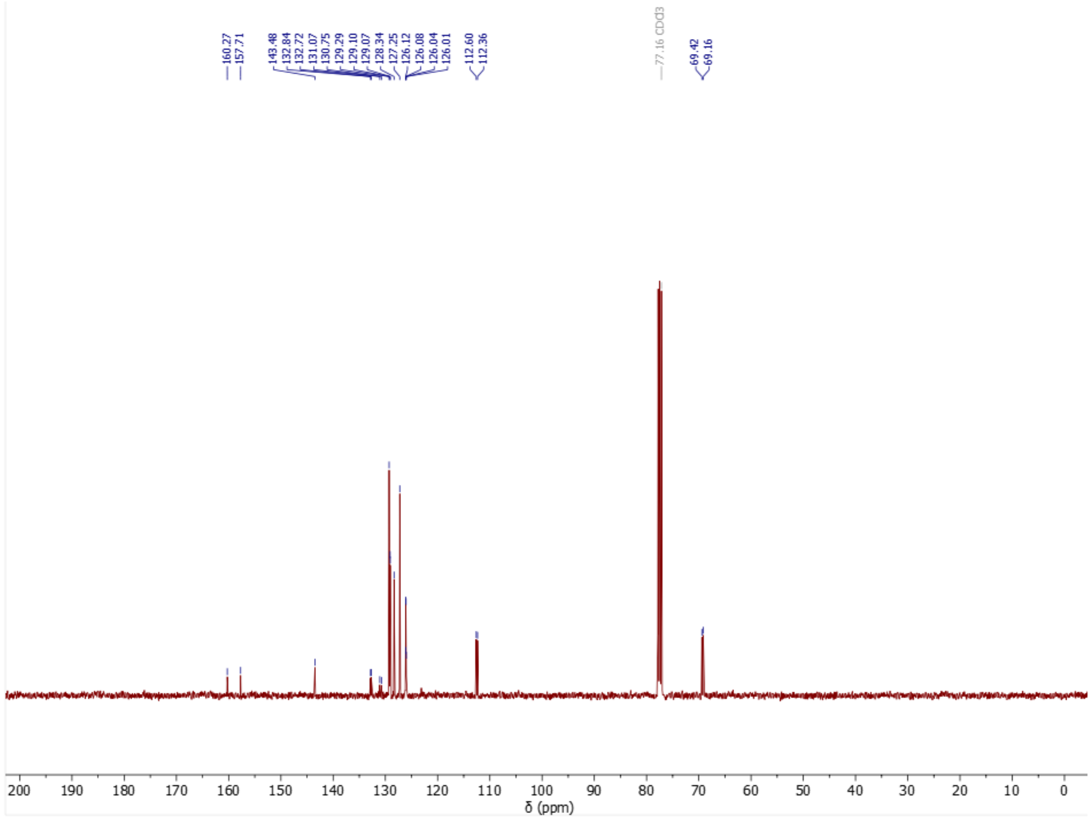

^{13}C NMR spectra of (E)-2-fluoro-3-phenyl-1-(4-(trifluoromethyl)phenyl)prop-2-en-1-ol, **11d**

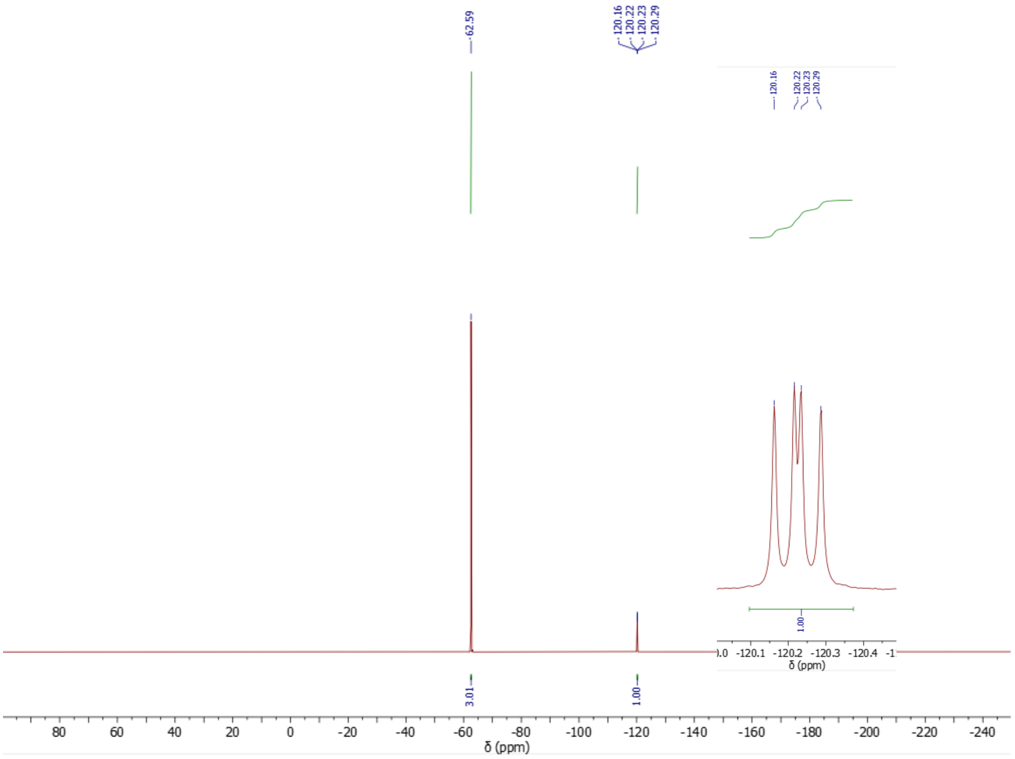

^{19}F NMR spectra of (E)-2-fluoro-3-phenyl-1-(4-(trifluoromethyl)phenyl)prop-2-en-1-ol, **11d**

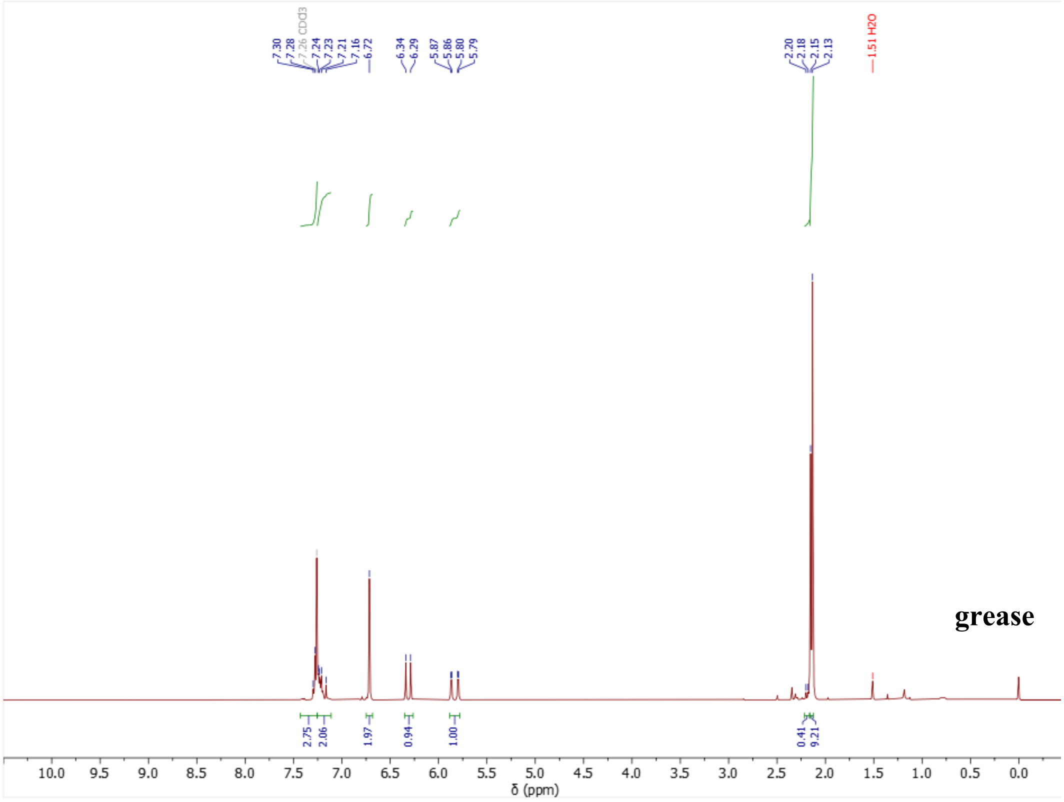

^{1}H NMR spectra of (*E*)-2-fluoro-1-mesityl-3-phenylprop-2-en-1-ol, **11e**

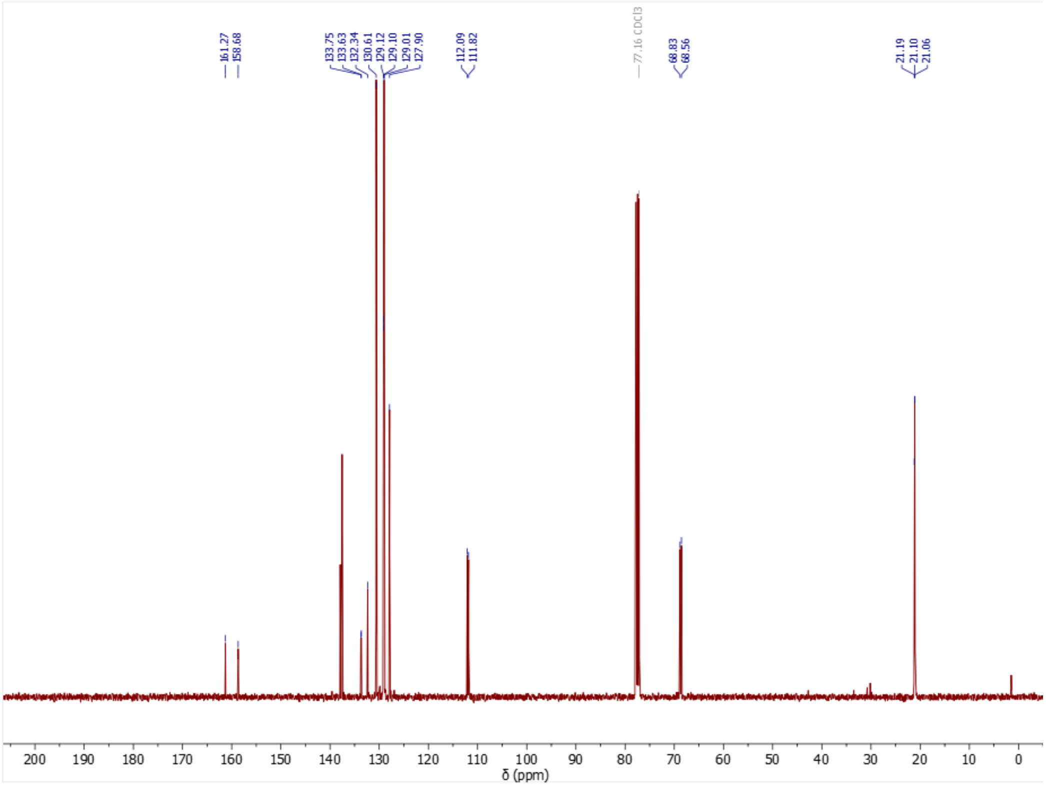

^{13}C NMR spectra of (*E*)-2-fluoro-1-mesityl-3-phenylprop-2-en-1-ol, **11e**

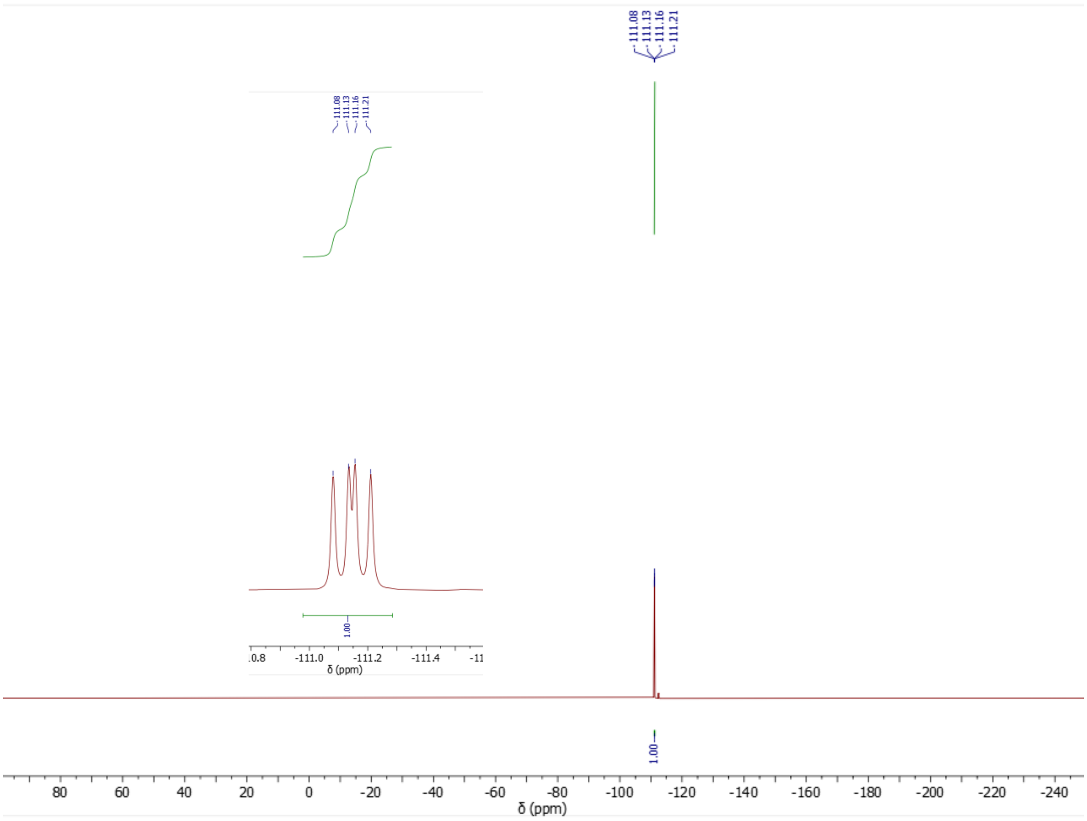

^{19}F NMR spectra of (*E*)-2-fluoro-1-mesityl-3-phenylprop-2-en-1-ol, **11e**

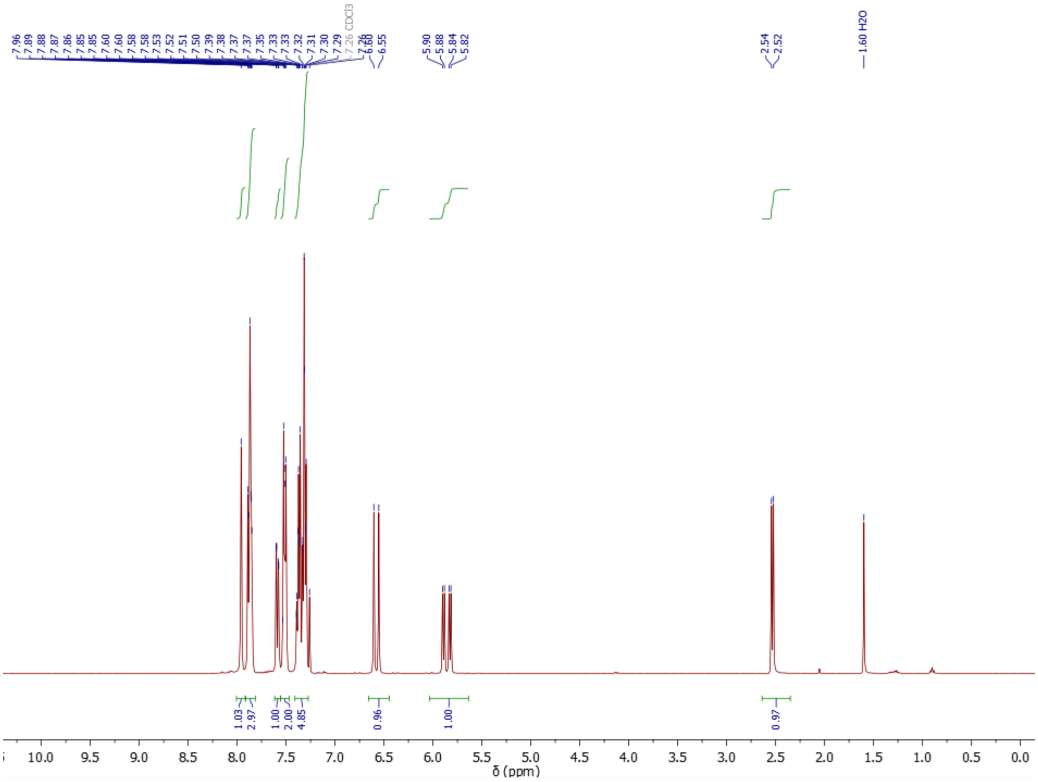

^{1}H NMR spectra of (E)-2-fluoro-1-(naphthalen-2-yl)-3-phenylprop-2-en-1-ol, **11f**

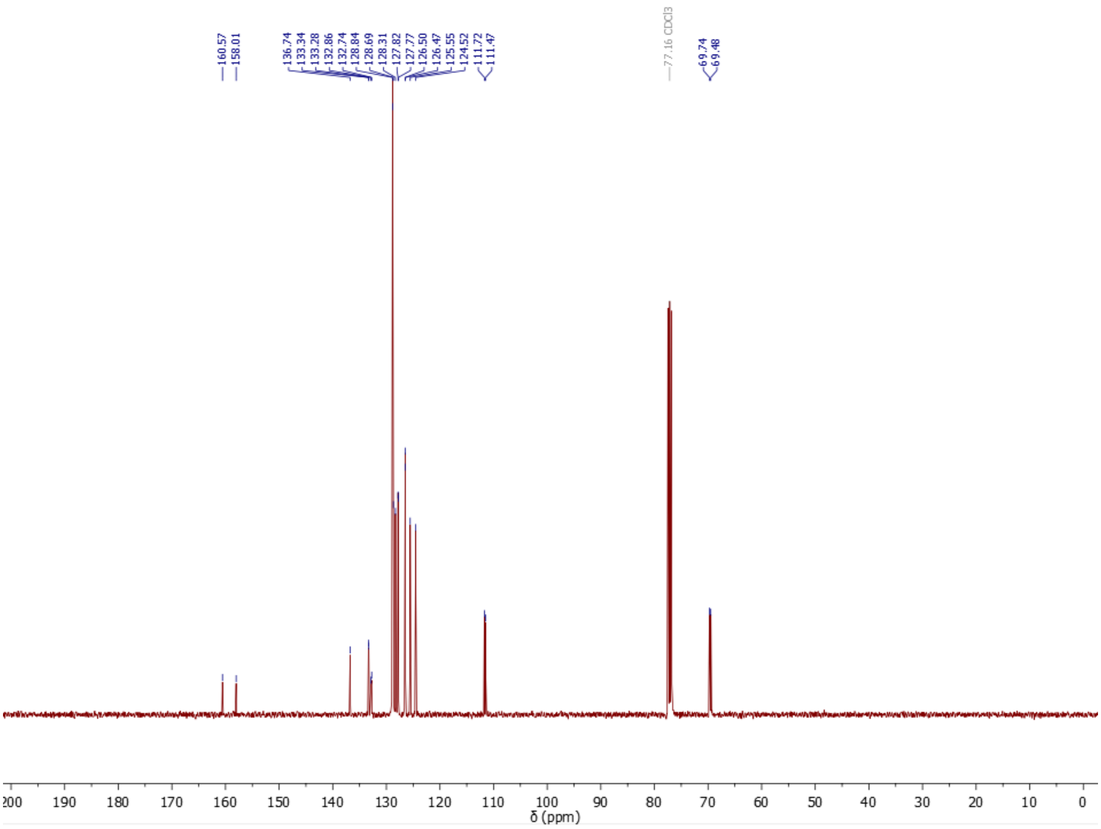

^{13}C NMR spectra of (E)-2-fluoro-1-(naphthalen-2-yl)-3-phenylprop-2-en-1-ol, **11f**

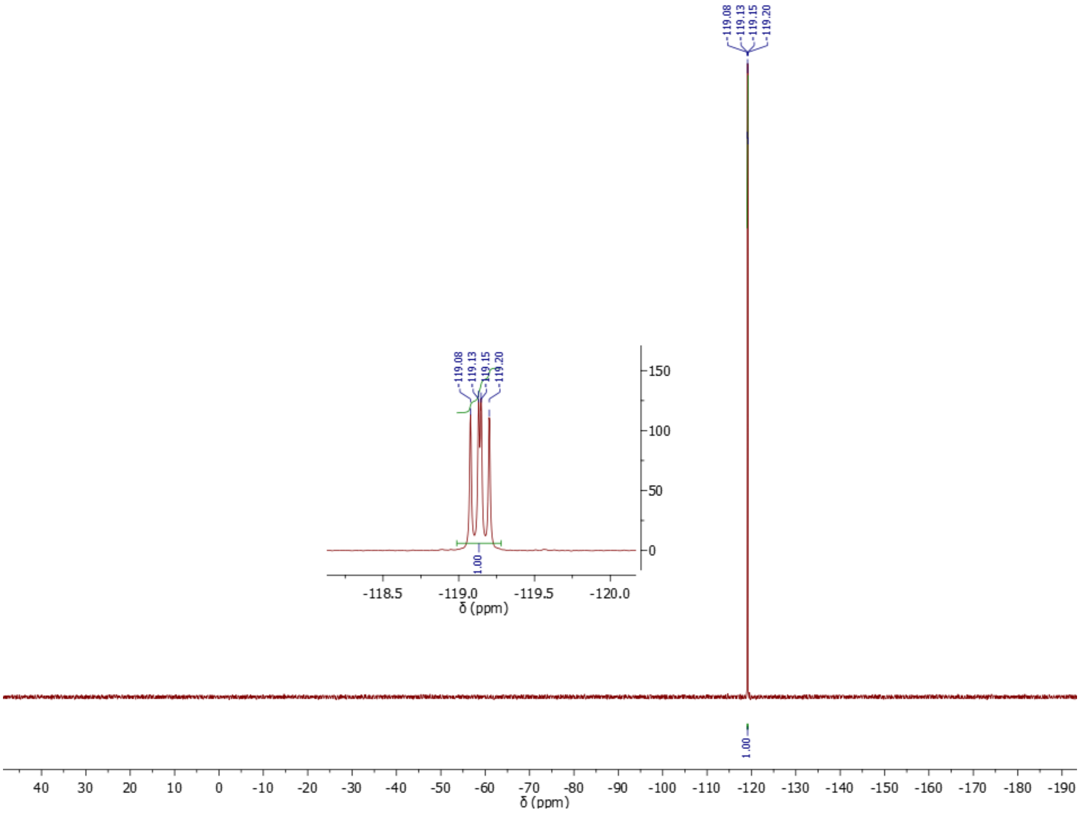

^{19}F NMR spectra of (E)-2-fluoro-1-(naphthalen-2-yl)-3-phenylprop-2-en-1-ol, **11f**

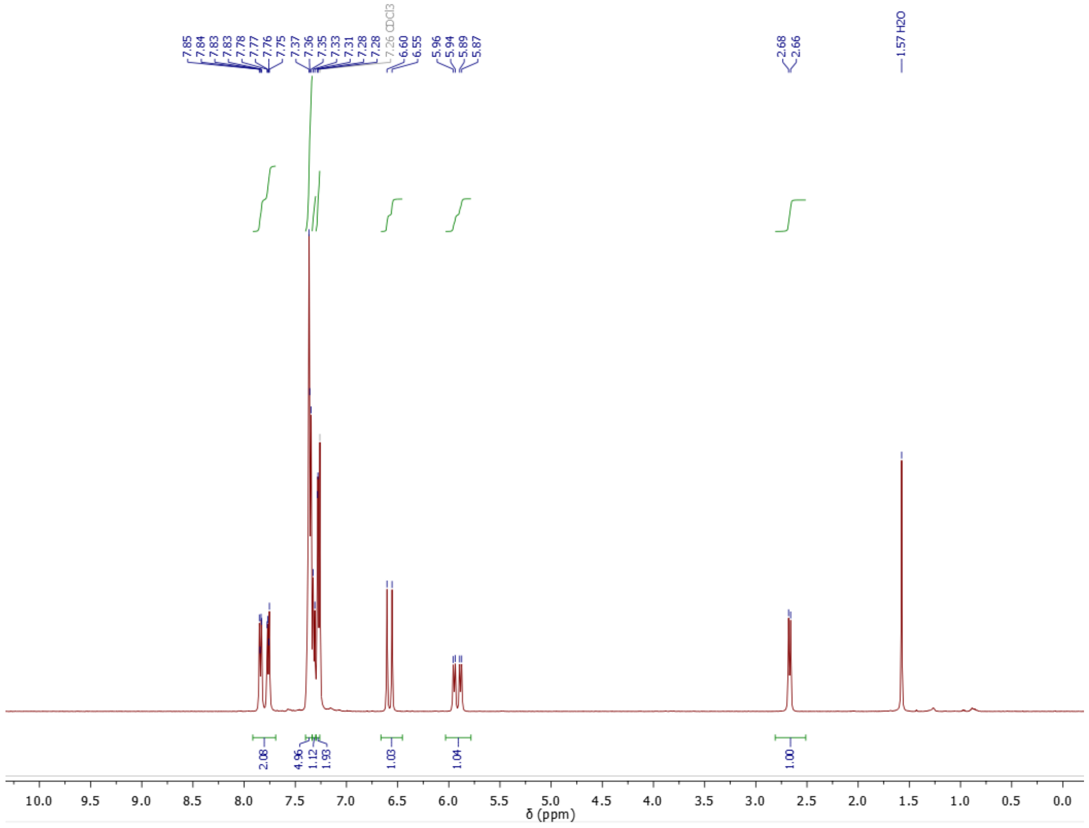

^{1}H NMR spectra of (E)-1-(benzo[b]thiophen-2-yl)-2-fluoro-3-phenylprop-2-en-1-ol, **11g**

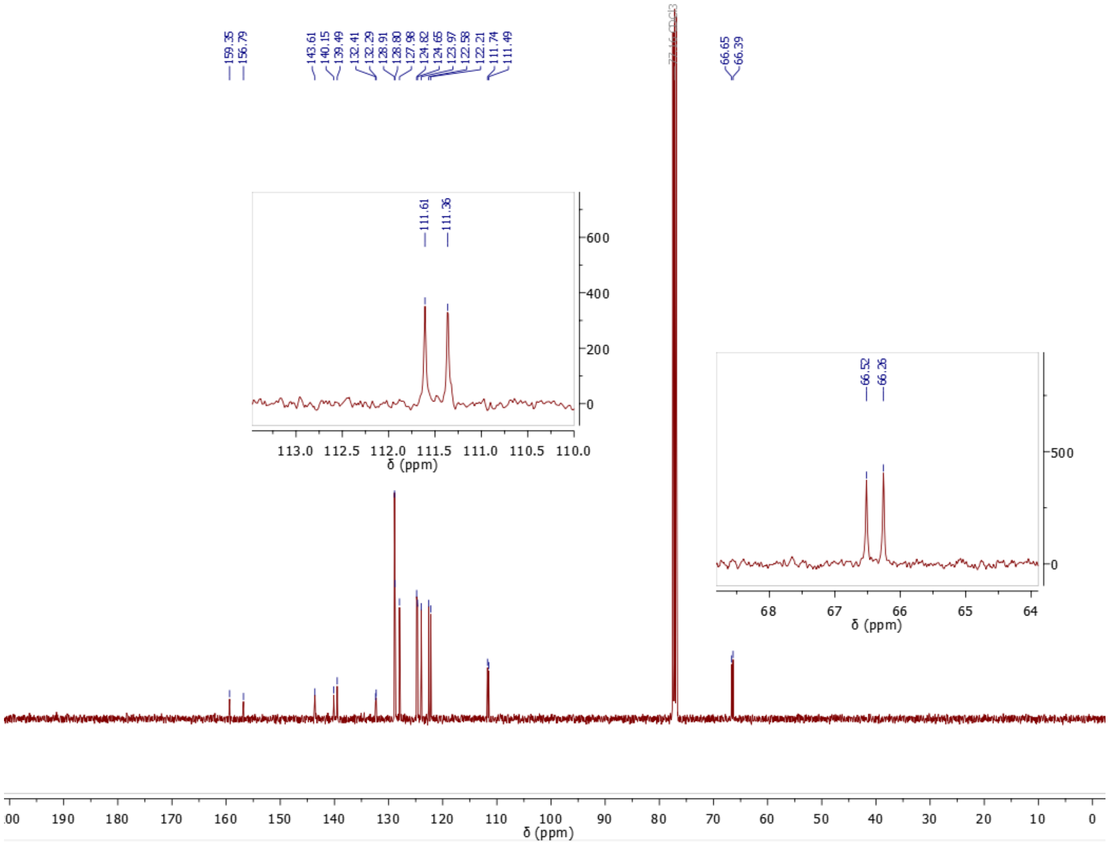

^{13}C NMR spectra of (E)-1-(benzo[b]thiophen-2-yl)-2-fluoro-3-phenylprop-2-en-1-ol, **11g**

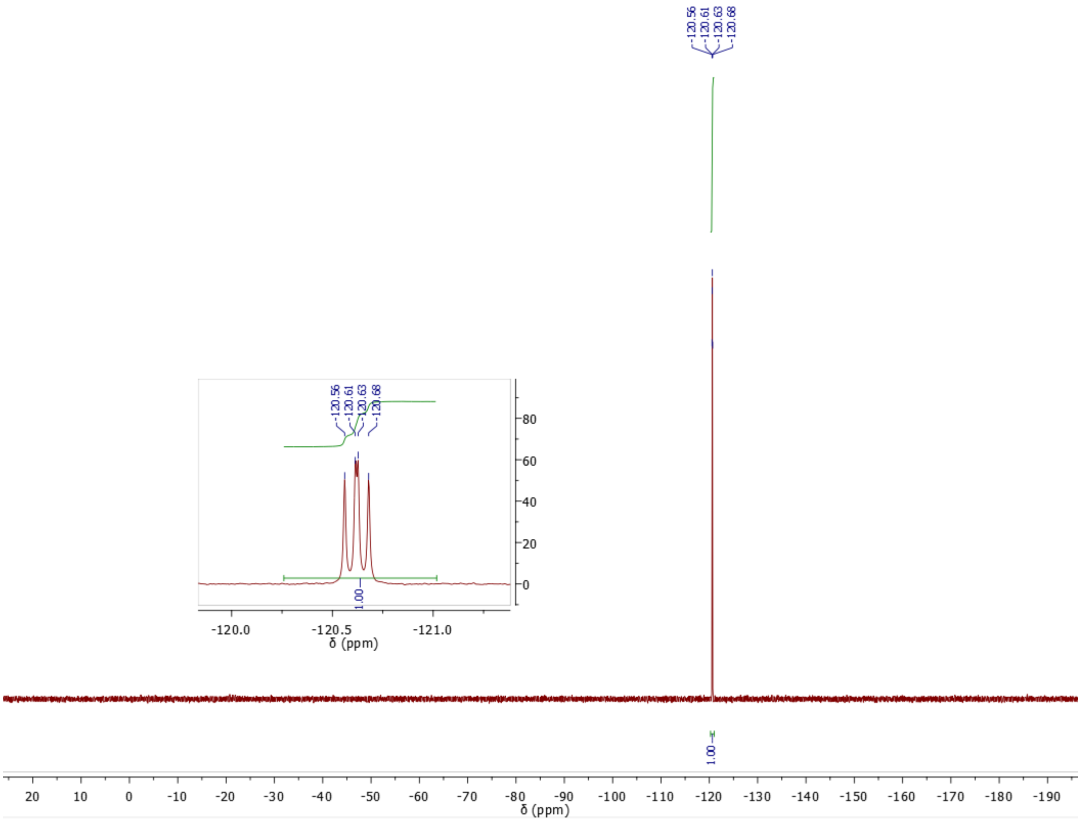

^{19}F NMR spectra of (E)-1-(benzo[b]thiophen-2-yl)-2-fluoro-3-phenylprop-2-en-1-ol, **11g**

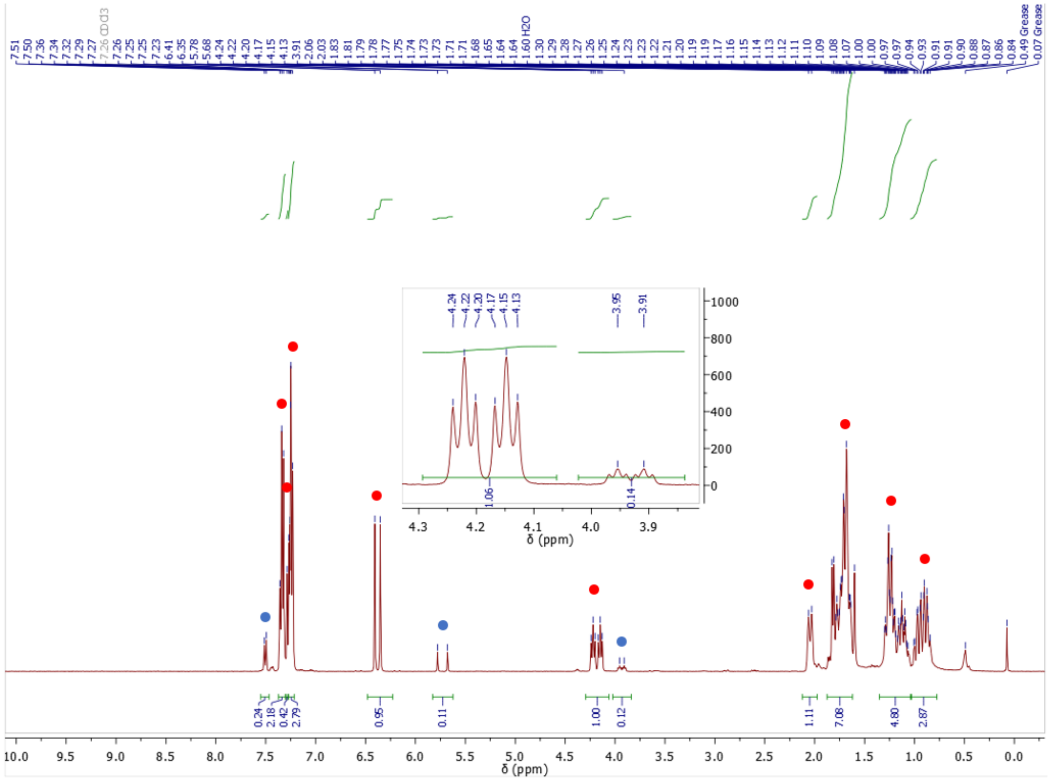

^{1}H NMR spectra of (E/Z)-1-cy clohexyl-2-fluoro-3-phenylprop-2-en-1-ol (*E* in red, *Z* in blue), **11h**

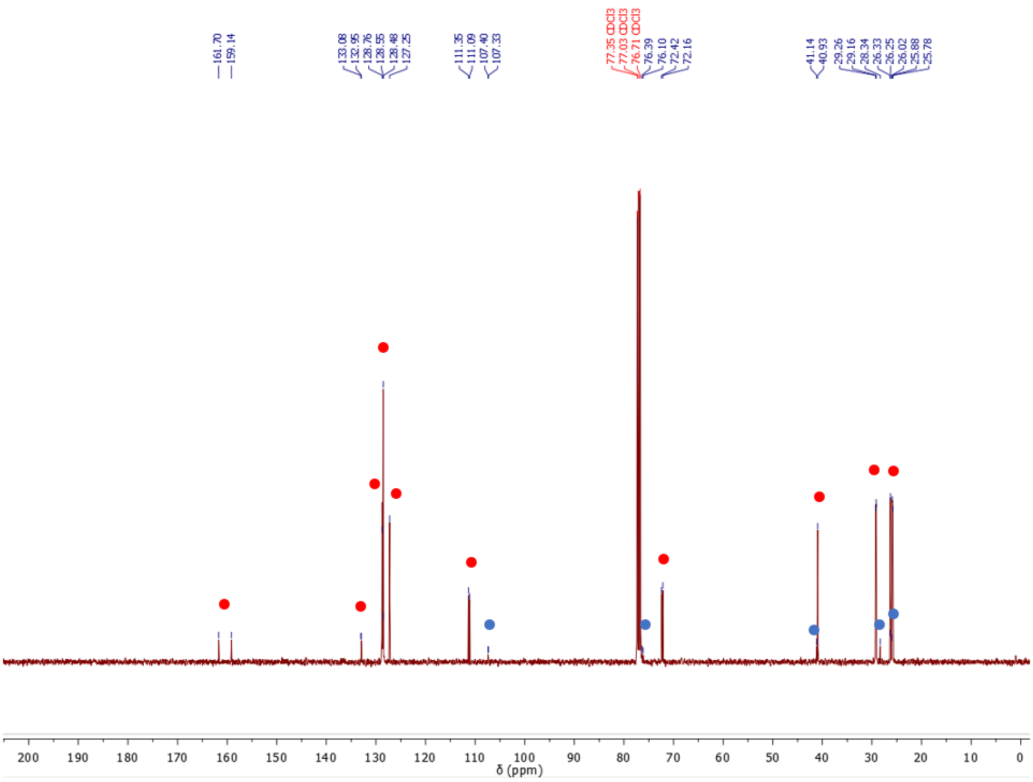

^{13}C NMR spectra of (E/Z)-1-cyclohexyl-2-fluoro-3-phenylprop-2-en-1-ol (*E* in red, *Z* in blue), **11h**

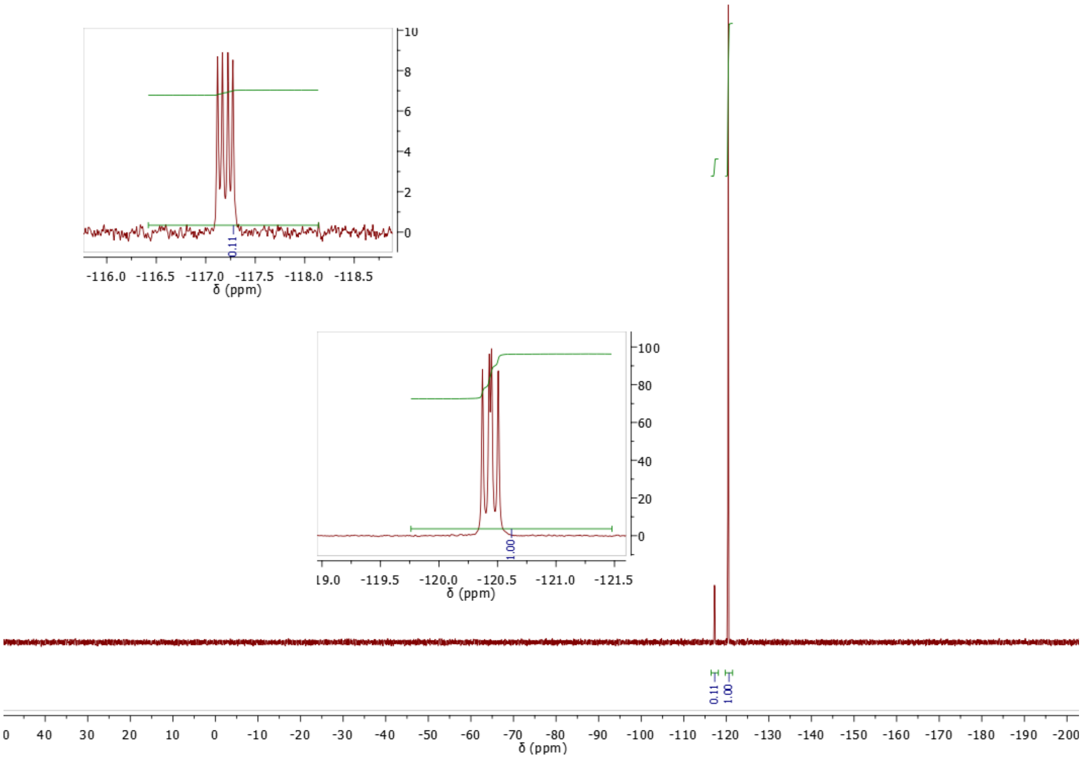

^{19}F NMR spectra of (E/Z)-1-cyclohexyl-2-fluoro-3-phenylprop-2-en-1-ol, **11h**

16.1 Computational Studies

DFT calculations were run using Gaussian 09 (Revision D.01) using the B3PW91 density functional.[13-18] Solvent and dispersion effects were integrated into the optimisation process.

Solvent effects were treated by the polarised continuum model (PCM) with a dielectric constant of 2.2706 (benzene).[19] Dispersion effects were treated by Grimme's D3 correction, 6-31G**(C, H)/6-311+G*(N, Si, Li, F) or def2-SVP(C,H)/def2-TZVP(Li,N,F,P) was used as hybrid basis sets.[20] Geometry optimisation calculations was performed without symmetry constraints. The Gaussian 09 default optimisation criteria was tightened to 10^{-9} on the density matrix and 10^{-7} on the energy matrix. The default numerical integration grid was also enhanced using a pruned grid with 99 radial shells and 590 angular points per shell. Frequency analyses for all stationary points were performed using the enhanced criteria to confirm the nature of the structures as either minima (no imaginary frequency) or transition states (only one imaginary frequency). Intrinsic reaction coordinate (IRC) calculations followed by full geometry optimisations on final points were used to connect transition states and minima located on the potential energy surface allowing a full energy profile (calculated at 298.15 K, 1 atm) to be assembled.[21, 22] The graphical user interface used to visualise the structures of the intermediates and transition states was GaussView 5.0.9.

Single point energy calculations were performed using Gaussian 09 (Revision D.01) using an ultrafine integration grid on the geometries optimised on the B3PW91/6-31G**(C, H)/6-311+G*(N, Si, Li, F)/ PCM level, and including Grimme's D3 dispersion correction, and implicit solvation was modelled using PCM method (benzene). The def2-TZVPP basis set was used for all atoms without the use of ECPs. Unless otherwise stated, all orbital analyses were performed using densities calculated at this level of theory. The free energies were then recomputed using GoodVibes (v3.2) using the following settings: c = 0.018/0.073 M; T = 298.15 K; v = 0.972; entropy: Grimme damping on RRHO, cutoff = 100 cm^{-1}; enthalpy: Head-Gordon q-RRHO, cutoff = 100 cm^{-1}; symmetry corrections enabled.[23-25]

Functional testing was performed using the ωB97X-D, M06-2X, and M06-L functionals. GIAO ^{19}F NMR simulation calculations were performed using Gaussian 09 (Revision D.01). .[13-18] The geometries were optimised on the B3PW91/6-31G**(C, H)/6-311+G*(N, Si, Li, F)/ PCM level, and including Grimme's D3 dispersion correction, and implicit solvation was modelled using PCM method (benzene).[20] Single point energies were calculated using the def2-QZVPP basis set for all atoms. Fluorobenzene (B3PW91/def2-QZVPP) (shielding = 286.3 ppm) was used as a reference.

A full NBO analysis was carried out and the relevant NPA charges and Wiberg Bond Indices were calculated. NBO analysis was performed using NBO 6.0 (using Gaussian 09 Rev D.01).[26]

16.2 NMR Simulations

NMR chemical shifts were carried out to aid assignment of the products.

Compound	**Experimental ^{19}F NMR Chemical Shift (ppm)**	**Computational ^{19}F NMR Chemical Shift (ppm)[b]**	**Dd**
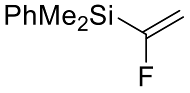	-102.9	-102.6	0.4
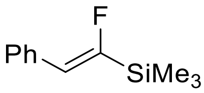	-114.4	-106.3	8.1
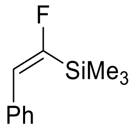	-106.1	-97.4	8.7
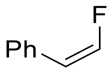	-122.0	-114.3	7.7
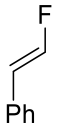	-130.3	-125.4	4.9
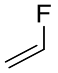	-115.0	-108.8	6.2
HCF_3	-79.6	-89.5	9.9

Table S11: Comparison of experimental and simulated NMR data for structures.[b] B3PW91/def2TZVPP/PCM(benzene)/GIAO//B3PW91/6.31G**(C,H)/6-311+G*(Li,N,F,Si)/PCM(benzene). Referenced to fluorobenzene (shielding = 286.3 ppm).

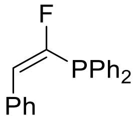

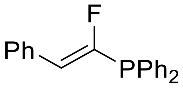

***Z* isomer** ***E* isomer**

Compound	Experimental ^{19}F NMR Chemical Shift (ppm)	Computational ^{19}F NMR Chemical Shift (ppm)[a]	Dd
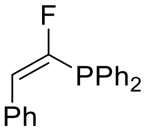 ***Z*-isomer**	-98.8	-86.9	11.9
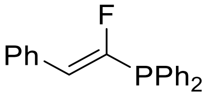 ***E*-isomer**	-105.1	-104.7	0.4

Table S12: Comparison of experimental and simulated NMR data for *Z* and *E*-isomer.[a] B3PW91-D3/def2-TZVPP/PCM(benzene)/GIAO//B3PW91-D3/def2-SVP(C,H)/def2-TZVP(Li,N,F,P)/PCM(benzene).Referenced to fluorobenzene (shielding = 286.3 ppm).

16.3 1·PMDETA + HCF_3 DFT Study

DFT calculations were performed to determine the transition state energies of the geminal, *E/Z*-isomers. Results showed that all transition states adopted carbene character where **PMDETA·LiF** was lost, and 1,2-migration occurred forming the geminal, *E/Z*-isomers. The geminal isomer proceeded via **TS4** ($\Delta G^{\ddagger}_{4}$ = 16.4 kcal/mol), and the *Z*-isomer proceeded via **TS2** ($\Delta G^{\ddagger}_{2}$ = 26.1 kcal/mol). The difference in energy between the transition states of the isomers ($\Delta\Delta G^{\ddagger}$ = 9.7 kcal/mol). This result alongside the NMR simulations suggests that the product observed experimentally is the geminal isomer as the reaction is more favourable kinetically. It is unlikely the product is the *E*-isomer due to its high energy transition state **TS3** ($\Delta G^{\ddagger}_{3}$= 33.8 kcal/mol) which is implausible to proceed at room temperature.

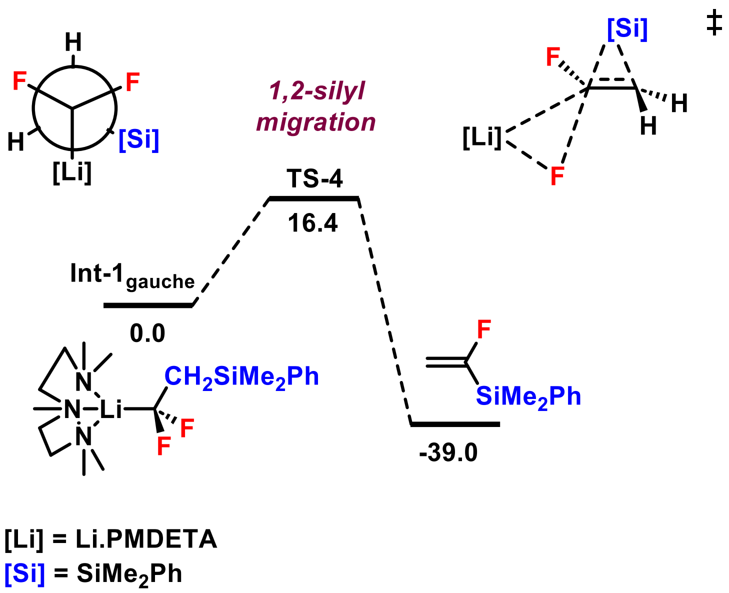

Figure S15: Calculated potential energy surface for the geminal in kcal/mol. B3PW91-D3/def2-TZVPP/PCM(benzene)/GoodVibes//B3PW91-D3/6-31G**(C,H)/6-311+G*(Li,N,F,Si)/PCM(benzene).

An alternative mechanism was found where the transition state proceeded with an α-elimination of **LiF·PMDETA** to form the carbene – we predict this transition state to be a low energy barrierless process. The carbene then undergoes 1,2-migration of the silyl group to the carbene centre with concurrent formation of the C=C double bond *via* **TS5** ($\Delta G^{\ddagger}_2$ = 9.1 kcal/mol). This alternative mechanism is lower in energy in comparison to the concerted 1,2-migration.

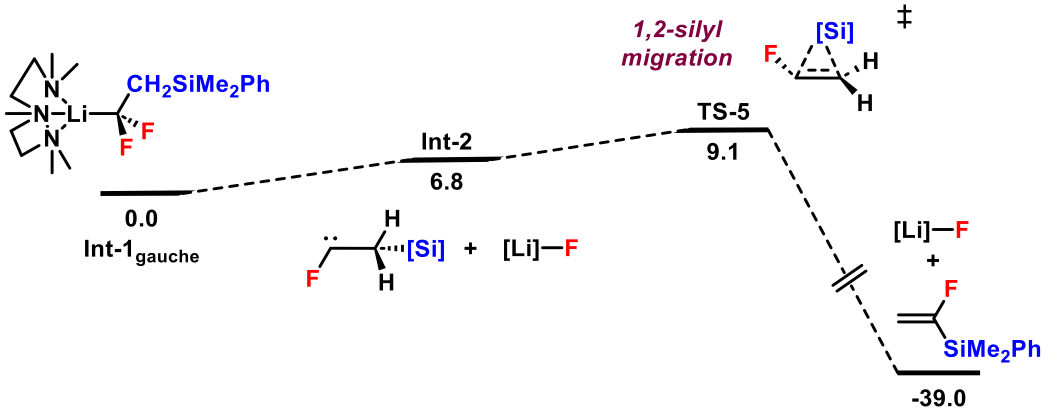

Figure S16: Alternative calculated potential energy surface for the geminal in kcal/mol. B3PW91-D3/def2-TZVPP/PCM(benzene)/GoodVibes//B3PW91-D3/6-31G**(C,H)/6-311+G*(Li,N,F,Si)/PCM(benzene).

DFT calculations were performed on reprotonation of the intermediate via **TS1** ($\Delta G^{\ddagger}_4$ = 27.5 kcal/mol). The difference in energy between the transition states is ($\Delta\Delta G^{\ddagger}$ = 11.1 kcal/mol), which suggests that reprotonation of the intermediate is not expected to be competitive.

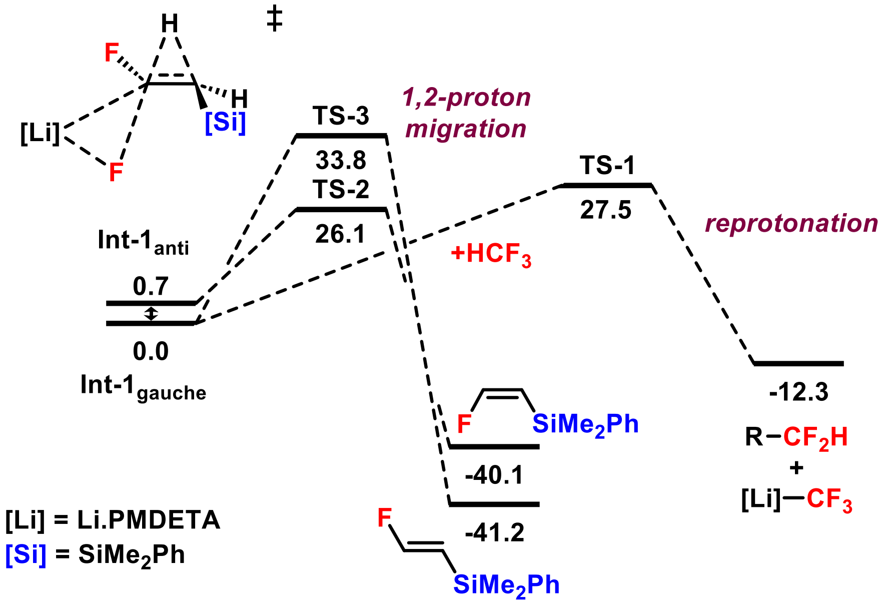

Figure S17: Calculated potential energy surface for the E/Z isomers in kcal/mol. B3PW91-D3/def2-TZVPP/PCM(benzene)/GoodVibes//B3PW91-D3/6-31G**(C,H)/6-311+G*(Li,N,F,Si)/PCM(benzene).

16.4 1·PMDETA Functional Testing

Functional benchmarking calculations were performed using the hybrid GGA functional B3PW91, the Minnesota hybrid meta functional M062X, and the long-range corrected functional ωB97xD with Grimme's D2 dispersion correction. The basis set along with solvent corrections (Benzene, PCM) and dispersion corrections (GD3) was maintained except from ωB97xD which includes Grimme's D2 dispersion corrections in the functional.

Across the different functionals, results were consistent and suggested that **TS1** is the kinetic product and **TS2**, **TS3**, and **TS4** are non-competitive transition states.

**Functional**	**$\Delta G_1^{\ddagger}$ (kcal/mol) TS-1**	**$\Delta G_2^{\ddagger}$ (kcal/mol) TS-2**	**$\Delta G_3^{\ddagger}$ (kcal/mol) TS-3**	**$\Delta G_4^{\ddagger}$ (kcal/mol) TS-4**
B3PW91	24.0	26.2	34.5	17.5
ωB97xD	23.5	21.7	28.7	17.9
M062X	23.6	26.2	29.7	17.4
M06L	23.0	19.5	28.6	12.7

Table S13: Calculated transition state energies for **1·PMDETA** with various density functionals.

16.5 NBO Analysis for Silyl Pathway

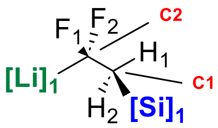

Int-1$_{gauche}$

	Int-1$_{gauche}$	TS4	HHC=CF(SiMe$_2$Ph)	LiF·PMDETA
Si1	1.65	1.62	1.56	
Li1	0.80	0.87		0.90
C1	-0.96	-0.68	-0.48	
C2	0.16	0.15	-0.001	
F1	-0.45	-0.37	-0.36	
F2	-0.46	-0.92		-0.95
H1	0.24	0.25	0.22	
H2	0.22	0.24	0.22	

Table S14: NPA charges of key stationary points for concerted silyl migration.

	Int-1$_{gauche}$	TS4	HHC=CF(SiMe$_2$Ph)	LiF·PMDETA
C1-C2	1.04	1.53	1.94	
C2-F1	0.79	0.89	0.87	
C2-F2	0.76	0.04		
C2-Li1	0.17	0.05		
C1-Si1	0.80	0.44		
C2-Si1		0.38	0.80	
C1-H1	0.90	0.89	0.92	
C1-H2	0.91	0.88	0.93	
Li1-F2				0.07

Table S15: Wiberg Bond Indices of key stationary points for concerted silyl migration

	**Int-1$_{gauche}$**	**Int-2**	**TS5**	**HHC=CF(SiMe$_2$Ph)**	**LiF·PMDETA**
Si1	1.65	1.66	1.63	1.56	
Li1	0.80				0.90
C1	-0.96	-0.97	-0.79	-0.48	
C2	0.16	0.32	0.20	-0.001	
F1	-0.45	-0.35	-0.36	-0.36	
F2	-0.46				-0.95
H1	0.24	0.25	0.23	0.22	
H2	0.22	0.27	0.26	0.22	

Table S16: NPA charges of key stationary points for 1,2-silyl migration.

	**Int-1$_{gauche}$**	**Int-2**	**TS5**	**HHC=CF(SiMe$_2$Ph)**	**LiF·PMDETA**
C1-C2	1.04	1.28	1.21	1.94	
C2-F1	0.79	0.98	0.97	0.87	
C2-F2	0.76				
C2-Li1	0.17				
C1-Si1	0.80	0.64	0.71		
C2-Si1		0.09	0.52	0.80	
C1-H1	0.90	0.88	0.94	0.92	
C1-H2	0.91	0.88	0.94	0.93	
Li1-F2					0.07

Table S17: Wiberg Bond Indices of key stationary points for 1,2-silyl migration

16.6 Energies for the Possible Pathways for Silyl Pathway

	SCF (BS1) eH	SCF (BS2) eH	G_{corr} (GoodVibes) eH	H_{corr} (GoodVibes) eH
**Int-1$_{gauche}$**	-1406.494329	-1406.769283	-1406.314181	-1406.221954
**HCF_3**	-338.2158349	-338.2694182	-338.271918	-338.240548
**TS1**	-1744.689359	-1745.013352	-1744.542322	-1744.440684
**R-CF_2H**	-878.5822549	-878.7383977	-878.571448	-878.513808
**LiCF_3.PMDETA**	-866.1467769	-866.3164114	-866.034219	-865.964923
**Int-1$_{anti}$**	-1406.489619	-1406.765514	-1406.313106	-1406.218779
**TS2**	-1406.446509	-1406.722095	-1406.272704	-1406.179227
**HFC=C(SiMe_2Ph)H**	-778.0999391	-778.2463684	-778.093534	-778.038115
**LiF·PMDETA**	-628.4317607	-628.5630399	-628.284538	-628.223272
**Int-1$_{gauche}$**	-1406.494329	-1406.769283	-1406.314181	-1406.221954
**TS3**	-1406.43372	-1406.709587	-1406.260344	-1406.167395
**HFC=CH(SiMe_2Ph)**	-778.1016288	-778.2476555	-778.095245	-778.039348
**LiF·PMDETA**	-628.4317607	-628.5630399	-628.284538	-628.223272
**Int-1$_{gauche}$**	-1406.494329	-1406.769283	-1406.314181	-1406.221954
**TS4**	-1406.464990	-1406.741158	-1406.288101	-1406.19536
**HHC=CF(SiMe_2Ph)**	-778.0983724	-778.2442035	-778.091716	-778.036315
**LiF·PMDETA**	-628.4317607	-628.5630399	-628.284538	-628.223272
**Int-1$_{gauche}$**	-1406.494329	-1406.769283	-1406.314181	-1406.221954
**Int-2**	-778.0239164	-778.1693296	-777.873321	-777.817503
**TS5**	-778.0216275	-778.1672164	-777.86957	-777.815579
**HHC=CF(SiMe_2Ph)**	-778.0983724	-778.2442035	-778.091716	-778.036315

Table S18: Energies for the possible pathways calculated at the B3PW91-D3/def2-TZVPP/PCM(benzene)/GoodVibes//B3PW91-D3/6-31G**(C,H)/6-311+G*(Li,N,F,Si)/PCM(benzene) level of theory

16.7 2·TMEDA + HCF_3 DFT Study

DFT calculations were performed to determine the transition state energies of the geminal isomer. Interestingly, results differed from the initial DFT study of **1·PMDETA**. Instead of the transition state proceeding with a concerted 1,2-migration and α-elimination of **LiF·PMDETA** to form the fluorinated alkene, this reaction proceeds differently. The first transition state proceeds with the α-elimination of **LiF·PMDETA** *via* **TS6** ($\Delta G^{\ddagger}_1$ = 21.8 kcal/mol) to form the carbene. The carbene then undergoes 1,2-migration of the phosphine group to the carbene centre with concurrent formation of the C=C double bond *via* **TS7** ($\Delta G^{\ddagger}_2$ = 21.7 kcal/mol).

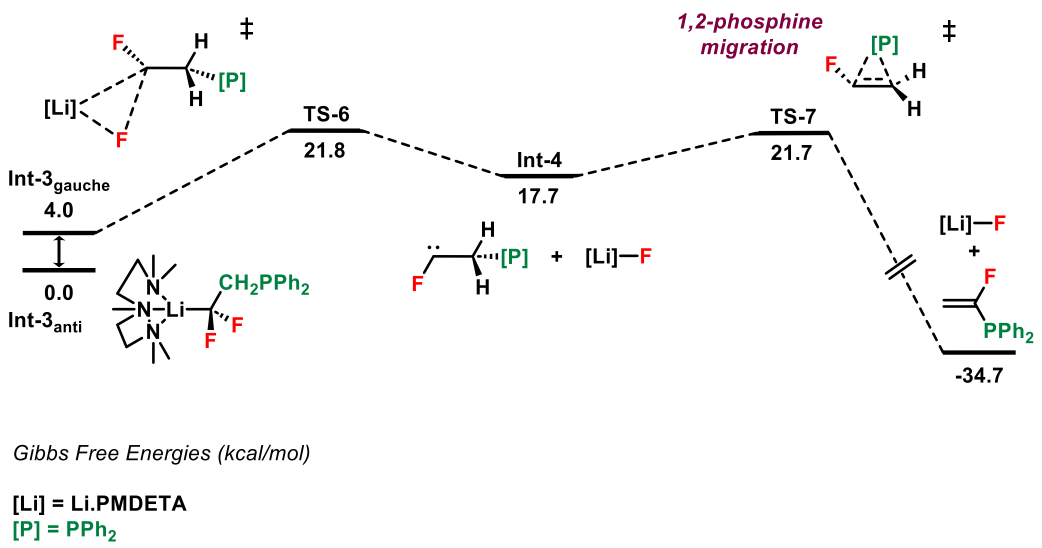

Figure S18: Calculated potential energy surface for phosphine migration in kcal/mol. B3PW91-D3/def2-TZVPP/PCM(benzene)/GoodVibes//B3PW91-D3/def2-SVP(C,H)/def2-TZVP(Li,N,F,P)/PCM(benzene).

16.8 4·TMEDA + HCF_3 DFT Study

DFT calculations were performed to determine the transition state energies of the *E* and *Z* isomers. Similarly to the **2·TMEDA** pathway, instead of the transition state proceeding with a concerted 1,2-migration and α-elimination of **LiF·PMDETA** to form the fluorinated alkene, this reaction proceeds differently. The first transition state proceeds with the α-elimination of **LiF·PMDETA** *via* **TS8** ($\Delta G^{\ddagger}_1$ = 23.7 kcal/mol) to form the carbene. The carbene then undergoes 1,2-migration of the phosphine group to the carbene centre with concurrent formation of the C=C double bond *via* **TS9** ($\Delta G^{\ddagger}_2$ = 19.4 kcal/mol) and **TS10** ($\Delta G^{\ddagger}_2$ = 19.6 kcal/mol) to form the *E* and *Z* isomers, respectively.

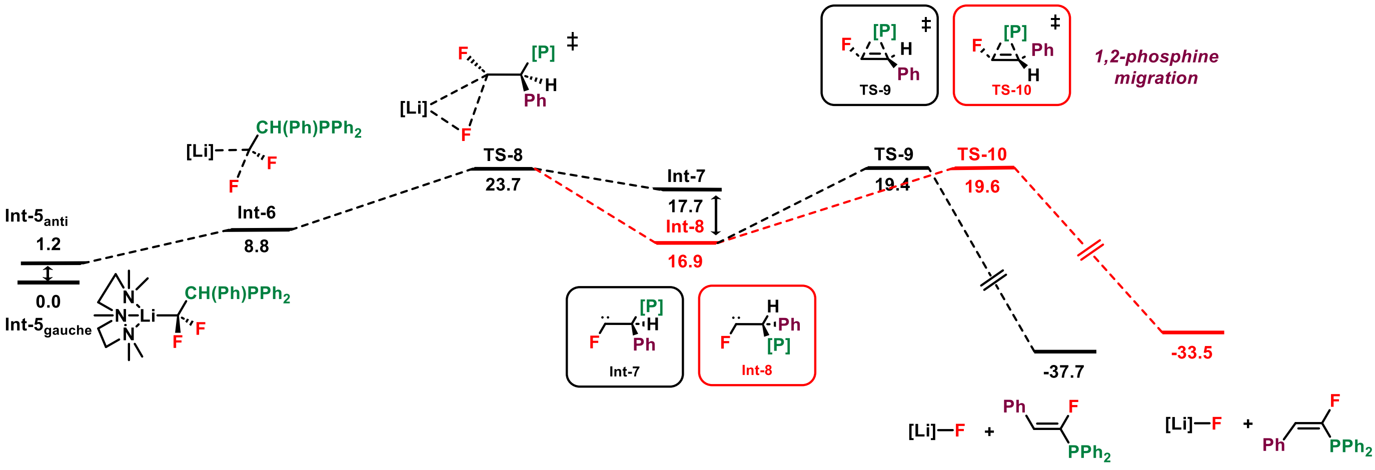

Figure S19: Calculated potential energy surface for phosphine migration in kcal/mol. B3PW91-D3/def2-TZVPP/PCM(benzene)/GoodVibes//B3PW91-D3/def2-SVP(C,H)/def2-TZVP(Li,N,F,P)/PCM(benzene).

16.9 NBO Analysis for Phosphine Pathway

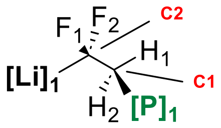

Int-3gauche

	**Int-3$_{gauche}$**	**TS6**	**Int-4**	**TS7**	**HHC=CF(PPh$_2$)**
P1	0.77	0.86	0.88	0.92	0.77
Li1	0.80	0.85			
C1	-0.78	-0.88	-0.85	-0.02	-0.45
C2	0.18	0.37	0.32	-0.48	0.09
F1	-0.43	-0.34	-0.34	-0.33	-0.34
F2	-0.44	-0.86			
H1	0.22	0.25	0.25	0.23	0.22
H2	0.24	0.26	0.27	0.24	0.23

Table S19: NPA charges of key stationary points for 1,2-phosphine migration.

	$Int\text{-}3_{gauche}$	TS6	Int-4	TS7	HHC=CF(PPh_2)
C1-C2	1.04	1.13	1.17	1.51	1.91
C2-F1	0.80	1.00	1.00	0.89	0.88
C2-F2	0.78	0.10			
C2-Li1	0.18	0.09			
C1-P1	0.89	0.82	0.80	0.45	0.95
C2-P1	0.06	0.10	0.17	0.89	0.02
C1-H1	0.91	0.88	0.89	0.90	0.92
C1-H2	0.91	0.89	0.88	0.90	0.92
Li1-F2	0.01	0.03			

Table S20: Wiberg Bond Indices of key stationary points for 1,2-phosphine migration

16.10 Energies for the Possible Pathways for Phosphine Pathway

	SCF (BS1) eH	SCF (BS2) eH	G_{corr} (GoodVibes) eH	H_{corr} (GoodVibes) eH
**Int-3$_{gauche}$**	-1609.448400	-1610.403282	-1608.978869	-1608.885421
**TS6**	-1609.417639	-1610.374798	-1608.947773	-1608.856326
**Int-4**	-981.226455	-981.7971215	-981.058636	-981.002497
**TS7**	-981.216752	-981.7869186	-981.050265	-980.994327
**HHC=CF(PPh$_2$)**	-981.309363	-981.8789172	-981.139954	-981.084248
**Int-5$_{anti}$**	-1840.269180	-1841.462606	-1839.724098	-1839.623389
**Int-6**	-1840.256719	-1841.451514	-1839.710663	-1839.610545
**TS8**	-1840.229113	-1841.426066	-1839.684771	-1839.584684
**Int-7**	-1212.038773	-1212.849447	-1211.796698	-1211.731668
**Int-8**	-1212.040684	-1212.851001	-1211.798369	-1211.733634
**TS9**	-1212.036271	-1212.036271	-1212.036271	-1211.730169
**TS10**	-1212.036127	-1212.846521	-1211.794062	-1211.730354
**H(Ph)C=CF(PPh$_2$)**	-1212.129874	-1212.939398	-1211.886285	-1211.821246
**(Ph)HC=CF(PPh$_2$)**	-1212.124943	-1212.933273	-1211.880733	-1211.816947

Table S21: Energies for the possible pathways calculated at the B3PW91-D3/def2-TZVPP/PCM(benzene)/GoodVibes//B3PW91-D3/6-31G**(C,H)/6-311+G*(Li,N,F,Si)/PCM(benzene) level of theory

16.11 Computational Coordinates

FHC=CHSiMe2Ph.log

SCF (RB3PW91) = -778.099939111
E(SCF)+ZPE(0 K)= -777.899047
H(298 K)= -777.884382
G(298 K)= -777.94074
Lowest Frequency = 24.6263cm-1

C	4.47854400	1.43307000	-0.88693300
H	5.17675500	1.04072000	-1.63470700
H	4.89595100	1.24261800	0.10725800
H	4.40866100	2.51697100	-1.02318900
Si	2.77888800	0.65746900	-1.07535400
C	1.59344500	1.38139400	0.19353700
H	1.13096900	0.73297700	0.93598200
H	0.58476100	3.12390200	1.00797500
C	2.10667100	0.96434400	-2.80361900
H	1.13721700	0.47314500	-2.93800900
H	1.97436200	2.03673100	-2.98021700
H	2.79464900	0.56931500	-3.55925400
C	1.25682500	2.66218800	0.28869300
F	1.75616300	3.59396600	-0.56266600
C	2.85583000	-1.19273200	-0.74075700
C	1.67170500	-1.94946000	-0.69849600
C	4.07086100	-1.86671400	-0.54146400
C	1.69810500	-3.32329000	-0.46594800
H	0.71033300	-1.45955600	-0.84762700
C	4.10487400	-3.24230300	-0.30903500
H	5.00733900	-1.31366700	-0.56448700
C	2.91799800	-3.97262900	-0.27071600
H	0.76990600	-3.88809800	-0.43644300
H	5.05727800	-3.74336300	-0.15646200
H	2.94219600	-5.04374200	-0.08866300

HCF3.log

SCF (RB3PW91) = -338.215834929
E(SCF)+ZPE(0 K)= -338.190714
H(298 K)= -338.186298
G(298 K)= -338.216833
Lowest Frequency = 504.8436cm-1

C	-0.68301500	0.57278400	0.00000000
H	-1.77690600	0.57281600	-0.00000100
F	-0.21038700	1.19925700	-1.08507000
F	-0.21039000	1.19925600	1.08507100
F	-0.21042300	-0.68015400	0.00000100

HFC=CHSiMe2Ph.log

SCF (RB3PW91) = -778.101628843
E(SCF)+ZPE(0 K)= -777.900883
H(298 K)= -777.886098
G(298 K)= -777.942913
Lowest Frequency = 15.5382cm-1

C	1.21573900	0.09394000	1.87037900
H	1.45750500	1.11634900	2.18091500
H	1.44621900	-0.57630100	2.70513800
H	0.13753400	0.04831000	1.68338100
Si	2.16939600	-0.39337000	0.32651800
C	1.72274700	-2.14666300	-0.15127100
H	1.85471200	-2.95759600	0.56563200
H	1.06640000	-1.79635400	-2.18352000
C	1.74948600	0.77415900	-1.08734800
H	2.30155600	0.51485800	-1.99694800
H	0.67788900	0.76111400	-1.31510700
H	2.02438600	1.79894100	-0.81508400
C	1.25378900	-2.46981300	-1.35101700
F	0.94011500	-3.74346600	-1.68862400
C	4.01981800	-0.34558600	0.67074600

C	4.57352000	0.57387300	1.57754500
C	4.89746400	-1.20804800	-0.00772000
C	5.94953800	0.63340600	1.79611100
H	3.92541600	1.25378800	2.12689700
C	6.27421800	-1.15330200	0.20660700
H	4.49711800	-1.93848200	-0.70845900
C	6.80256200	-0.23095900	1.10981500
H	6.35631000	1.35131600	2.50361300
H	6.93432900	-1.83195100	-0.32734600
H	7.87493700	-0.18771700	1.28064500

HHC=CFSiMe2Ph.log

SCF (RB3PW91) = -778.09727322
E(SCF)+ZPE(0 K)= -777.896874
H(298 K)= -777.882127
G(298 K)= -777.938473
Lowest Frequency = 18.1642cm-1

C	-0.53657700	-2.17546600	-2.45204500
H	-0.72280200	-2.14012400	-3.52187600
H	0.00406800	-3.02512200	-2.05003700
C	-0.94899200	-1.21051500	-1.63345200
F	-1.63192800	-0.15149000	-2.18494000
C	0.28393400	0.47385800	0.59073900
C	1.55470100	0.40385700	1.18365300
C	-0.21730100	1.74310200	0.25165200
C	2.29868200	1.55761500	1.43189500
H	1.97448900	-0.56148300	1.45783900
C	0.52350600	2.89804500	0.49491400
H	-1.19574800	1.83245400	-0.21533600
C	1.78375600	2.80653600	1.08712000
H	3.27966600	1.48146500	1.89355700
H	0.11922500	3.86936500	0.22251800
H	2.36233000	3.70628700	1.27856700
Si	-0.73248300	-1.06646500	0.24347000
C	0.14452100	-2.61107800	0.84292100
H	-0.45202000	-3.50201300	0.62132100
H	1.12433300	-2.72976900	0.36891900
H	0.29573500	-2.57000800	1.92689600
C	-2.44190600	-0.89105300	0.99685200
H	-3.05569600	-1.77393100	0.78911300
H	-2.37193300	-0.76493700	2.08256300
H	-2.95677700	-0.01611200	0.58699800

Int-1_anti.log

SCF (RB3PW91) = -1406.48961883
E(SCF)+ZPE(0 K)= -1405.95606
H(298 K)= -1405.922844
G(298 K)= -1406.02308
Lowest Frequency = 4.4757cm-1

C	3.24092100	-0.86886100	-1.99795000
H	3.86021900	-1.41164400	-2.72176000
H	3.56009100	0.17808900	-1.97905200
H	2.20177000	-0.88481800	-2.33848400
Si	3.34712000	-1.64555100	-0.29072600
C	2.39589500	-0.66947400	1.03547500
H	2.37667600	-1.29206700	1.94218800
H	2.97488800	0.22394900	1.30497000
C	2.74758200	-3.43092800	-0.35532300
H	2.81791700	-3.90596500	0.62998400
H	1.69963700	-3.45836300	-0.66779300
H	3.34613300	-4.02272200	-1.05716500
C	-2.06836200	-0.74954900	4.36397700
H	-2.70086200	0.14113200	4.32467000
H	-1.22738900	-0.53121900	5.02842300
H	-2.65863900	-1.57068000	4.80745600
C	-0.68225700	-2.25383000	3.10883100
H	-1.21382600	-3.15444700	3.46266500
H	0.13849800	-2.04529600	3.80186100
H	-0.25487500	-2.44614400	2.12216000
C	-2.65573400	-1.32021500	2.07776000
H	-3.42630300	-1.99046100	2.49932200
H	-2.22459700	-1.83060700	1.21085900
C	-3.30124300	-0.01525400	1.61704400
H	-4.16456600	-0.24314700	0.96903400
H	-3.70441500	0.52204000	2.48114400

C	-2.20395800	0.48909300	-0.48481900
H	-1.95986300	-0.57014600	-0.57937200
H	-3.66324100	2.52102700	0.98239800
H	-2.06065200	2.81178300	0.32272500
C	-2.12714900	2.76840200	2.47690200
H	-2.28920400	3.86052800	2.53577200
H	-2.74317900	2.32044400	3.26564100
C	-0.40828500	2.68218900	4.15750700
H	-0.49289400	3.75284000	4.41301700
H	0.61590700	2.35940100	4.36550100
H	-1.08323800	2.11802200	4.80794800
C	0.20127600	3.12483900	1.87475500
H	0.07367700	4.21987800	1.93447600
H	0.07558900	2.79908200	0.84018100
H	1.22585600	2.87371100	2.15929100
Li	-0.54897900	0.44906000	2.00846000
N	-1.56586600	-1.08768100	3.03560000
N	-2.33193400	0.84400200	0.92840500
N	-0.73091800	2.41736400	2.75798200
C	0.97072400	-0.21368500	0.74872600
F	1.10086500	0.71263100	-0.35730400
F	0.35249000	-1.36181800	0.06754900
C	5.15825100	-1.67565000	0.26458200
C	5.52906600	-2.36889200	1.43123700
C	6.17154900	-0.99411600	-0.42976800
C	6.84708500	-2.38253300	1.88497600
H	4.77433900	-2.91206400	1.99845900
C	7.49409500	-1.00225700	0.01680000
H	5.92758700	-0.44707100	-1.33812700
C	7.83518600	-1.69706400	1.17651500
H	7.10590800	-2.92842900	2.78913600
H	8.25880600	-0.46780200	-0.54172000
H	8.86434400	-1.70615600	1.52622800
H	-3.12856600	0.70120700	-1.04920800
C	-2.59523400	2.27212100	1.11006100

Int-1_gauche.log

SCF (RB3PW91) = -1406.49432912

E(SCF)+ZPE(0 K)= -1405.960108

H(298 K)= -1405.927293

G(298 K)= -1406.023583

Lowest Frequency = 15.4339cm-1

C	2.68611100	1.13366100	3.60869500
H	3.18005100	2.00152400	4.05899500
H	2.68587900	0.31826200	4.34095500
H	1.64227500	1.39288000	3.40851700
Si	3.49473000	0.58745100	1.99538700
C	2.45576900	-0.78831100	1.23587700
H	2.95652900	-1.22819400	0.35621500
H	2.39211100	-1.57735700	1.99798000
C	3.68293900	2.04059900	0.81575900
H	4.28814800	1.74206900	-0.04747900
H	2.70826900	2.35815800	0.43540700
H	4.17960200	2.88869700	1.30023900
C	-1.95907900	-0.36521100	4.57962800
H	-2.65969700	0.44803000	4.37445400
H	-1.14826100	0.04028100	5.19177300
H	-2.48907300	-1.13453100	5.16842000
C	-0.43780600	-1.95837600	3.63388900
H	-0.89726800	-2.80522000	4.17296600
H	0.36612800	-1.54947400	4.25352600
H	0.00023100	-2.31660200	2.70016300
C	-2.45945700	-1.39873100	2.43685000
H	-3.17663000	-2.04745500	2.97129800
H	-1.96944400	-2.01376600	1.67569400
C	-3.20806700	-0.25962800	1.74841900
H	-4.03655100	-0.67565800	1.15088200
H	-3.67234900	0.38547000	2.50071700
C	-2.15229300	-0.03453800	-0.42096300
H	-1.84116200	-1.07640100	-0.33836000
H	-1.35161200	0.48344500	-0.95309000
H	-3.08841300	0.02368000	-1.00286500

C	-2.69030500	1.95436100	0.84333000
H	-3.76935500	2.08399100	0.64710700
H	-2.16980600	2.39755900	-0.01118600
C	-2.32417700	2.71406900	2.11428200
H	-2.59108900	3.77866400	1.98097400
H	-2.92647900	2.34749000	2.95352500
C	-0.70006000	3.13875300	3.81980900
H	-0.93978700	4.21569700	3.85248900
H	0.34327000	3.01108400	4.11847000
H	-1.32928400	2.62223500	4.55018300
C	-0.02240400	3.20609400	1.51549500
H	-0.29306600	4.26159300	1.33900500
H	-0.03674600	2.66528800	0.56617900
H	1.00334800	3.16998500	1.88874200
Li	-0.51409400	0.48959600	2.04279300
N	-1.40997200	-0.90343400	3.33886100
N	-2.30582000	0.54464100	0.91422900
N	-0.91216600	2.56691500	2.49056100
C	1.04027600	-0.35303900	0.90994500
F	1.16108900	0.39123300	-0.32652600
F	0.40669400	-1.58494200	0.41731000
C	5.21497000	-0.09705800	2.37318500
C	5.97297300	-0.69346500	1.34930500
C	5.78951800	-0.03396700	3.65302000
C	7.24678200	-1.20506700	1.59148900
H	5.56086300	-0.76397500	0.34383500
C	7.06481900	-0.54310600	3.90376100
H	5.23547800	0.41966800	4.47234700
C	7.79608200	-1.13037500	2.87237200
H	7.81141300	-1.66289800	0.78315600
H	7.48713300	-0.48175200	4.90376200
H	8.78889000	-1.52882300	3.06459900

Int-2.log

SCF (RB3PW91) = -778.023916413

E(SCF)+ZPE(0 K)= -777.8252

H(298 K)= -777.810227

G(298 K)= -777.866669

Lowest Frequency = 31.9235cm-1

C	-0.62249100	-1.71671300	-1.60830400
H	0.40903800	-1.95542800	-1.90196700
H	-1.28662400	-2.58745200	-1.58894800
C	-1.26230600	-0.58251500	-2.21772100
F	-0.30263700	0.19122400	-2.76252400
C	0.30813000	0.34466600	0.58418500
C	1.57826000	0.36254900	1.18310700
C	-0.26406900	1.57312700	0.20975000
C	2.25449800	1.56307800	1.39996700
H	2.05002700	-0.56853300	1.48875300
C	0.40801800	2.77438200	0.42410300
H	-1.24156300	1.59340000	-0.26795400
C	1.66961500	2.77033600	1.02041300
H	3.23665600	1.55585500	1.86499400
H	-0.04992000	3.71271300	0.12352000
H	2.19574400	3.70639900	1.18703200
Si	-0.61666300	-1.25825000	0.30303300
C	0.27982700	-2.70367400	1.09399300
H	-0.27109300	-3.63374200	0.91966400
H	1.28978300	-2.82769100	0.69074900
H	0.35860200	-2.55444300	2.17656900
C	-2.38576600	-1.14804800	0.90052100
H	-2.94771800	-2.04617900	0.62246600
H	-2.40712500	-1.05322300	1.99180500
H	-2.89816000	-0.28411400	0.46845900

LiCF3PMDETA.log

SCF (RB3PW91) = -866.146776864

E(SCF)+ZPE(0 K)= -865.804438

H(298 K)= -865.782729

G(298 K)= -865.853622

Lowest Frequency = 28.6793cm-1

C	3.75889600	3.14340400	0.88481200
H	2.89970700	2.92262300	1.52354700
H	4.27117200	2.19997600	0.67616600
H	3.38149300	3.55309000	-0.06817600
C	5.84259300	4.31920600	0.69756300

H	5.56127500	4.81994200	-0.24463100
H	6.33027000	3.37157500	0.45163300
H	6.56279200	4.93876100	1.23688300
C	4.01756800	5.32192600	1.92201400
H	3.41944600	5.72398100	1.08514700
H	4.80169600	6.05642700	2.13174000
C	3.13565900	5.16313200	3.15807600
H	2.60515500	6.11006100	3.35434200
H	2.36024300	4.41606500	2.96254600
C	4.54854300	5.88249500	4.99219300
H	5.14025600	6.45590300	4.27574800
H	5.24141200	5.52397900	5.75643500
H	3.80333300	6.55289400	5.45343400
C	3.16101900	3.91350600	5.26163900
H	2.15492000	4.32254900	5.45807700
H	3.68773500	3.92598200	6.22045400
C	3.02782800	2.47342500	4.77026500
H	2.49344300	1.88122800	5.53523000
H	2.40637500	2.44601400	3.86758600
C	4.12664700	0.61155200	3.71309900
H	3.60139600	-0.14240100	4.32436000
H	5.09750000	0.20131600	3.42125600
H	3.54228700	0.78765200	2.80538700
C	5.14997500	1.64845100	5.62180900
H	4.64045000	1.00802800	6.36232900
H	5.41519200	2.59820400	6.09014100
H	6.08598800	1.16900000	5.32585200
Li	5.34979700	3.43406900	3.45106000
N	4.67882500	4.06245900	1.55157200
N	3.92236500	4.74191300	4.32351700
N	4.32183700	1.86958900	4.43158300
C	7.28015000	4.01466600	3.98651100
F	7.47293800	4.20037500	5.37124900
F	8.41318800	3.26847700	3.63958500
F	7.61812100	5.29429100	3.48681600

LiFPMDETA.log

SCF (RB3PW91) = -628.431760726

E(SCF)+ZPE(0 K)= -628.099315

H(298 K)= -628.081154

G(298 K)= -628.142149

Lowest Frequency = 58.2599cm-1

C	3.88388900	3.18211600	0.93219600
H	3.01725600	2.92892400	1.54811500
H	4.43543400	2.25796000	0.73853800
H	3.51649600	3.57820500	-0.03130100
C	5.92929200	4.42761900	0.81185500
H	5.66715800	4.92807700	-0.13689600
H	6.45475300	3.49731500	0.58032200
H	6.61546400	5.06138000	1.37839200
C	4.03353300	5.36066300	1.98260900
H	3.43946100	5.74661900	1.13436000
H	4.78211800	6.12561100	2.21373300
C	3.12669000	5.16388800	3.19512600
H	2.56130600	6.09258000	3.38458700
H	2.38023400	4.39622000	2.97078100
C	4.46525400	5.90098000	5.07384300
H	5.05628100	6.50187400	4.37864800
H	5.14521200	5.55317500	5.85538300
H	3.69312200	6.54855700	5.52511300
C	3.11850500	3.90117300	5.28477000
H	2.10479700	4.29873800	5.46924400
H	3.62598900	3.90114300	6.25420900
C	3.00505300	2.46735400	4.76853000
H	2.46079400	1.86220200	5.51739300
H	2.39631800	2.44949700	3.85692200
C	4.14208600	0.64206500	3.68827700
H	3.62059800	-0.13854400	4.27018700
H	5.12450000	0.25732900	3.40028800
H	3.56893000	0.83197900	2.77627400
C	5.11733700	1.64177700	5.63614300
H	4.61516200	0.95800500	6.34352900
H	5.33955400	2.58202300	6.14396900
H	6.07639300	1.21414700	5.33870800
Li	5.46499700	3.47720300	3.55825200
N	4.74917400	4.13155200	1.62470100
N	3.88947900	4.75327500	4.37807300
N	4.30582100	1.88150000	4.44142000

F	7.11412900	3.61545100	3.97944500

R-CF2H.log

SCF (RB3PW91) = -878.582254897
E(SCF)+ZPE(0 K)= -878.365422
H(298 K)= -878.349739
G(298 K)= -878.408433
Lowest Frequency = 24.8182cm-1

C	1.85239000	-0.99923300	3.05555300
H	2.41841700	-0.78306800	3.96803100
H	2.01049900	-2.05258000	2.79972300
H	0.78840900	-0.86743600	3.28185600
Si	2.36872600	0.14473300	1.65587200
C	1.28858000	-0.36374000	0.16246900
H	1.30333000	-1.45563200	0.06518800
H	0.25400200	-0.08516300	0.40035700
C	1.99997500	1.92952900	2.10731200
H	2.29683100	2.60943300	1.30350700
H	0.92896500	2.07237300	2.28951600
H	2.54222600	2.21339400	3.01577000
C	1.62077800	0.21151300	-1.18868300
F	1.79604000	1.57776900	-1.11367200
F	2.79856800	-0.30241900	-1.66452000
C	4.20072100	-0.05607200	1.29035500
C	4.86521100	0.86091100	0.45715400
C	4.95197700	-1.10752100	1.83932100
C	6.22439600	0.73033500	0.18017000
H	4.31227000	1.68096200	0.00511800
C	6.31345000	-1.24333400	1.56542800
H	4.47410600	-1.83343100	2.49356500
C	6.95168100	-0.32376600	0.73463700
H	6.71661100	1.44850100	-0.47031000
H	6.87522300	-2.06529500	2.00159500
H	8.01192900	-0.42718700	0.51953700
H	0.85978000	0.03542500	-1.95837300

TS-1.log

SCF (RB3PW91) = -1744.68935851
E(SCF)+ZPE(0 K)= -1744.132643
H(298 K)= -1744.095219
G(298 K)= -1744.202119
Lowest Frequency = -1210.0875cm-1

C	3.57635600	0.92150500	5.22087600
H	4.46239500	1.37414000	5.67992400
H	3.52437400	-0.12429400	5.54412500
H	2.69514700	1.43735700	5.62007800
Si	3.62056400	1.06954400	3.34052000
C	1.98567700	0.29107000	2.73641000
H	1.88862500	-0.67155700	3.25108000
H	1.18367200	0.92767900	3.12874000
C	3.71857300	2.89323100	2.88330900
H	3.67298300	3.02481200	1.79834300
H	2.90020100	3.46493400	3.33530400
H	4.66288100	3.31783400	3.24215900
C	-1.26345000	-0.59249600	4.03103000
H	-1.78151900	0.37006000	4.06132500
H	-0.22567900	-0.42170600	4.32134800
H	-1.72852500	-1.25850400	4.77739600
C	-0.52835900	-2.41157300	2.65152000
H	-0.93370700	-3.16583100	3.34627100
H	0.50628400	-2.20453900	2.92936400
H	-0.53022700	-2.81958400	1.63943200
C	-2.70158300	-1.45370200	2.28938900
H	-3.24275200	-1.97160500	3.10013300
H	-2.67074100	-2.14043500	1.43854900
C	-3.45571400	-0.19260000	1.89269900
H	-4.52234200	-0.43221400	1.74487000
H	-3.41812500	0.53255800	2.71149100
C	-3.39613600	-0.24731600	-0.51678200
H	-3.18050600	-1.31524700	-0.47621900
H	-2.88924800	0.14590900	-1.39898300
H	-4.48475700	-0.10638500	-0.62480900
C	-3.17488200	1.85807300	0.62173100
H	-4.21757100	2.07540800	0.90936900

H	-3.07489900	2.17584000	-0.41975100
C	-2.23330200	2.67739700	1.49391500
H	-2.46871200	3.74814300	1.35491200
H	-2.40650600	2.45566100	2.55438800
C	0.01721700	3.15645000	2.15348500
H	-0.14986200	4.24401900	2.07382400
H	1.06712700	2.94970100	1.94424900
H	-0.20645500	2.84943400	3.18037800
C	-0.45359600	2.78908900	-0.15998000
H	-0.68817500	3.84799900	-0.36134600
H	-0.97560300	2.16437800	-0.88636000
H	0.61604800	2.62387400	-0.29814600
Li	-0.70527700	0.26986200	1.21609400
N	-1.31183000	-1.16978700	2.68408200
N	-2.88390700	0.42022100	0.68440100
N	-0.81748100	2.40779200	1.21251400
C	1.76669500	0.03251500	1.25389200
F	2.26984600	1.17422100	0.55807500
F	2.67462800	-0.98503800	0.91013100
C	5.14680200	0.19771600	2.67367300
C	5.47027400	0.27839900	1.30774300
C	6.01490300	-0.51929600	3.51220800
C	6.61486500	-0.33314900	0.80120500
H	4.80400700	0.80475800	0.62929200
C	7.16169000	-1.13658700	3.00945800
H	5.79926100	-0.60200700	4.57540800
C	7.46396300	-1.04288100	1.65199200
H	6.84121500	-0.26389000	-0.25962500
H	7.81816700	-1.68861500	3.67750400
H	8.35567600	-1.52288000	1.25712200
C	0.14461100	-1.01925500	-0.81525000
H	0.74944000	-0.45794600	0.32399800
F	-0.48225900	-0.12732100	-1.66527700
F	1.07300900	-1.63340200	-1.58773500
F	-0.81597500	-1.99306100	-0.58120000

TS-2.log

SCF (RB3PW91) = -1406.44650911

E(SCF)+ZPE(0 K)= -1405.917101

H(298 K)= -1405.883646

G(298 K)= -1405.981362

Lowest Frequency = -1004.6449cm-1

C	5.31319800	1.17471000	0.04287900
H	5.87347700	1.05618900	-0.89153100
H	5.97715900	0.90722200	0.87220800
H	5.04696300	2.23138200	0.14639800
Si	3.75007200	0.12549800	0.02744300
C	2.87894400	0.42095700	1.71073900
H	2.67296600	-0.45725300	2.32172100
H	3.49953500	1.11029000	2.66645100
C	2.62564200	0.61016300	-1.38352900
H	1.67457800	0.08878800	-1.24348800
H	2.45068600	1.69055300	-1.38700100
H	3.07142600	0.32309200	-2.34228300
C	-0.74733100	0.79805800	4.61612200
H	-1.65589000	1.25964200	4.22270600
H	0.07094700	1.51340400	4.49870500
H	-0.90201200	0.59760400	5.69132100
C	0.79364000	-1.01707000	4.40750200
H	0.66600400	-1.36834300	5.44669100
H	1.59167700	-0.26987700	4.39238200
H	1.09524400	-1.86373400	3.78378000
C	-1.53180100	-1.38253200	3.88566500
H	-1.92527100	-1.53445600	4.90733900
H	-1.13128100	-2.34746200	3.55717000
C	-2.67212800	-0.96896700	2.95681900
H	-3.48414200	-1.71407500	3.02339600
H	-3.09939300	-0.02356700	3.30356100
C	-2.12690600	-2.07549000	0.86961600
H	-1.53331000	-2.78813700	1.44648000
H	-1.60586700	-1.92922600	-0.07881400
H	-3.12351900	-2.51489700	0.68692200
C	-2.96824500	0.19175800	0.82477500
H	-4.05908300	0.05631600	0.93611000
H	-2.74859300	0.04141700	-0.23680300
C	-2.60132600	1.61931600	1.22351100
H	-3.17031000	2.32249400	0.58683500
H	-2.92295500	1.80988400	2.25403800

C	-0.84918400	3.15712100	1.79881400
H	-1.33454700	4.00752300	1.28769100
H	0.23113400	3.31297000	1.79576300
H	-1.18572800	3.14283300	2.83890200
C	-0.67074300	1.88646800	-0.22346700
H	-1.22138600	2.60689000	-0.85431600
H	-0.74282500	0.88885000	-0.66172700
H	0.38626900	2.15735200	-0.22121700
Li	-0.22644000	0.00739000	1.77336300
N	-0.42715000	-0.41804900	3.87252800
N	-2.20680500	-0.80275000	1.57893600
N	-1.16171400	1.88136900	1.15645500
C	2.35907000	1.58920800	2.27270000
F	2.61590300	2.66686400	1.41999300
F	0.82664800	-0.86860500	0.70498100
C	4.19968500	-1.69774800	-0.01952800
C	3.19038100	-2.67273400	0.09572300
C	5.52765200	-2.12786900	-0.17521200
C	3.50852700	-4.02992200	0.05326300
H	2.15975300	-2.34208400	0.23318100
C	5.84490400	-3.48642000	-0.21692300
H	6.33061200	-1.39874400	-0.26388700
C	4.83372000	-4.43991400	-0.10352600
H	2.71899000	-4.77249300	0.14312500
H	6.87892500	-3.79959500	-0.33830500
H	5.07822500	-5.49881100	-0.13628400

TS-3.log

SCF (RB3PW91) = -1406.43371998

E(SCF)+ZPE(0 K)= -1405.904924

H(298 K)= -1405.871576

G(298 K)= -1405.968638

Lowest Frequency = -1280.861cm-1

C	2.49574100	1.13565400	3.25216700
H	2.90258400	2.13652600	3.43435200
H	2.52712200	0.58391400	4.19816900
H	1.44908900	1.24500100	2.95386700
Si	3.48278800	0.27348300	1.91341000
C	2.75935000	-1.47480700	1.65941300
H	3.37806000	-2.26872800	1.21595300
H	2.12949300	-1.68257600	2.86976400
C	3.34041100	1.08567800	0.24047700
H	4.00249400	0.60905400	-0.49037100
H	2.29924600	0.97347600	-0.09474900
H	3.60648800	2.14678800	0.29851900
C	-1.10656900	-0.12458400	4.20146600
H	-1.17175400	0.87814000	3.76926000
H	-0.05264000	-0.31294200	4.42461400
H	-1.67498000	-0.15251200	5.14935500
C	-1.27955800	-2.46762000	3.72232100
H	-1.79923300	-2.69806700	4.67025800
H	-0.20346100	-2.57147300	3.87806700
H	-1.57928200	-3.20301900	2.97072800
C	-2.99966500	-0.98712300	2.95584400
H	-3.60861700	-1.05316500	3.87799600
H	-3.29175000	-1.83656700	2.32909300
C	-3.32751000	0.32552000	2.24928100
H	-4.39025600	0.31424000	1.94351200
H	-3.22094100	1.14412100	2.96436500
C	-2.75469600	-0.30305400	-0.01199900
H	-2.71972400	-1.35277600	0.29365600
H	-1.99627900	-0.15937100	-0.78514200
H	-3.76057900	-0.09833700	-0.42161600
C	-2.43747000	1.96226100	0.67005400
H	-3.45178900	2.27034600	0.34987500
H	-1.79584100	2.00280400	-0.21472900
C	-1.95452400	2.98294800	1.69617800
H	-2.10443600	3.98603200	1.24690900
H	-2.59849700	2.95958200	2.58269900
C	-0.32015600	3.64911700	3.30249500
H	-0.44739200	4.72895500	3.09305200
H	0.70371200	3.49088800	3.65339300
H	-1.00085500	3.37928700	4.11762900
C	0.36874200	3.10985000	1.06812200
H	0.21010500	4.12111200	0.64556700
H	0.29438400	2.35110900	0.28454500
H	1.38741100	3.06722200	1.46168100
Li	-0.43371800	-0.23153400	1.41874200

N	-1.57282200	-1.12292800	3.24328100
N	-2.43678500	0.56877600	1.11969500
N	-0.57628300	2.81332000	2.14037100
C	1.39925900	-1.68765600	1.83325500
F	0.26423500	0.22284400	-0.12136600
F	1.02947600	-2.95862100	1.44992800
C	5.26763600	0.03698200	2.45427600
C	5.72268400	0.43055400	3.72323200
C	6.19368000	-0.56118700	1.58096300
C	7.04955700	0.23428300	4.10769700
H	5.03624100	0.89973600	4.42466900
C	7.51918500	-0.76307600	1.96050700
H	5.87914800	-0.87377000	0.58643000
C	7.94921100	-0.36434100	3.22692700
H	7.38084100	0.54885800	5.09385700
H	8.21748600	-1.22807400	1.26971300
H	8.98280800	-0.51877600	3.52468300

TS-4.log

SCF (RB3PW91) = -1406.46498951

E(SCF)+ZPE(0 K)= -1405.932774

H(298 K)= -1405.899586

G(298 K)= -1405.995748

Lowest Frequency = -295.1434cm-1

C	2.64122200	0.41960200	4.28483600
H	3.19203500	0.86393900	5.12176200
H	2.56739600	-0.65523300	4.47661000
H	1.63164500	0.83412000	4.26223600
Si	3.54973000	0.77903700	2.68840200
C	2.83755800	-0.48452200	1.17340400
H	3.50056100	-0.75358900	0.34713700
H	2.59144100	-1.28236300	1.87380400
C	3.82554900	2.59974900	2.33448400
H	4.20923800	2.74295100	1.31968200
H	2.89896400	3.17142900	2.41872900
H	4.55785100	3.00350700	3.04289600
C	-1.48067600	-0.56131500	4.25144400
H	-2.22299100	0.23996300	4.29114600
H	-0.55900000	-0.18228100	4.70190500
H	-1.84851100	-1.39973700	4.87110200
C	-0.17617200	-1.98586000	2.83291500
H	-0.49480800	-2.92778900	3.31514600
H	0.70682200	-1.61617900	3.36201800
H	0.08912600	-2.16078500	1.78634300
C	-2.43110300	-1.45390500	2.20577500
H	-2.95510800	-2.20357300	2.82807400
H	-2.10452700	-1.95075400	1.28726600
C	-3.39314100	-0.32801000	1.83777600
H	-4.34354000	-0.76642000	1.48557100
H	-3.64367400	0.25297600	2.73122500
C	-3.04387900	0.06560100	-0.52170000
H	-2.58153300	-0.91757000	-0.62371900
H	-2.54115200	0.71708700	-1.24047200
H	-4.11807800	0.00798900	-0.77621300
C	-3.21688100	1.95830600	0.99713200
H	-4.29252900	2.07387700	1.22252500
H	-3.05010000	2.47452400	0.04671000
C	-2.40024900	2.63402600	2.09656800
H	-2.69389600	3.69823700	2.16898500
H	-2.64442300	2.17776500	3.06263400
C	-0.22928700	2.84054300	3.10444500
H	-0.35382600	3.90391300	3.37892000
H	0.83188200	2.63675300	2.94914100
H	-0.57482500	2.22530500	3.94025100
C	-0.47428800	3.27708400	0.76572100
H	-0.67977500	4.35502600	0.89795500
H	-0.94114700	2.94525000	-0.16479900
H	0.60222000	3.12957000	0.65834500
Li	-0.63653200	0.36782100	1.24752000
N	-1.22100600	-0.96343400	2.87584900
N	-2.82081000	0.56534000	0.82942300
N	-0.95841400	2.48472000	1.89472100
C	1.97919300	0.60282300	1.16730900
F	2.19885300	1.38823600	0.06854800
F	-0.16978000	-0.77339800	0.00716400
C	5.25572200	-0.03625400	2.78080900
C	5.40220900	-1.32015000	3.33418200
C	6.40769400	0.60126100	2.29209300

C	6.64791900	-1.93994000	3.40559500
H	4.53277800	-1.85161700	3.71711000
C	7.65747500	-0.01559400	2.35816500
H	6.33660900	1.59315300	1.85262000
C	7.78004500	-1.28697700	2.91639600
H	6.73671000	-2.93160900	3.84128300
H	8.53514500	0.49765400	1.97424000
H	8.75266500	-1.76851100	2.96921500

TS-5.log

SCF (RB3PW91) = -778.021627451
E(SCF)+ZPE(0 K)= -777.822842
H(298 K)= -777.80867
G(298 K)= -777.862896
Lowest Frequency = -220.2302cm-1

C	5.32373800	-4.51986900	-0.40903400
H	6.24298900	-4.97546200	-0.79425000
H	4.50391900	-5.20686400	-0.18942800
C	4.94229400	-3.19894100	-0.67178800
F	5.94322900	-2.54837500	-1.32387800
C	6.36362100	-2.22067600	1.81349300
C	7.65519300	-2.24271500	2.36479200
C	5.78083900	-0.97077400	1.54020300
C	8.34237900	-1.05873400	2.63311500
H	8.13720300	-3.19027100	2.59187200
C	6.46162500	0.21294900	1.81268400
H	4.79234400	-0.92838500	1.08985900
C	7.74537800	0.17050200	2.35884800
H	9.34234300	-1.09701700	3.05684300
H	5.99489000	1.16918600	1.59251100
H	8.27928600	1.09372800	2.56742600
Si	5.41446900	-3.80899800	1.51972300
C	6.37399300	-5.24939700	2.26579500
H	5.81634300	-6.18277500	2.13873500
H	7.35824300	-5.37703900	1.80513300
H	6.51561500	-5.08011800	3.33942000
C	3.68504000	-3.76258100	2.22758200
H	3.10328300	-4.63608800	1.91479600
H	3.74039500	-3.76603100	3.32225200
H	3.15249300	-2.86448100	1.90626900

HHC=CFPPh2.log

SCF (RB3PW91) = -981.309362644
E(SCF)+ZPE(0 K)= -981.091237
H(298 K)= -981.076021
G(298 K)= -981.134597
Lowest Frequency = 22.3013cm-1

C	-0.22305000	-0.95388200	-3.35118400
H	-0.62118900	-0.49157500	-4.25848600
H	0.25142000	-1.93361800	-3.41798900
C	-0.30485900	-0.33850100	-2.17058400
F	-0.90108600	0.87965000	-2.09984700
C	1.35303600	0.37457000	-0.01875200
C	2.74566100	0.21247300	0.00998400
C	0.79903800	1.60003300	0.38703400
C	3.57363200	1.25700900	0.42826700
H	3.18458200	-0.74132200	-0.29664000
C	1.62594000	2.64063500	0.80638300
H	-0.28463500	1.73410000	0.37667600
C	3.01408600	2.47157700	0.82676600
H	4.65765800	1.11837700	0.44744700
H	1.18567300	3.59128500	1.11854600
H	3.65952900	3.28940100	1.15784300
C	-1.13535500	-0.94486400	0.45020800
C	-2.42306800	-1.14457100	-0.06966000
C	-0.98286000	-0.79244300	1.83811300
C	-3.53212100	-1.17928400	0.77705600
H	-2.56595000	-1.27066200	-1.14630400
C	-2.09263800	-0.82726700	2.68217700
H	0.01209800	-0.63400300	2.26325100
C	-3.37186300	-1.01968900	2.15480700
H	-4.52944600	-1.32720800	0.35448300
H	-1.95649900	-0.70094900	3.75949600
H	-4.24160100	-1.04375200	2.81623500
P	0.35204100	-1.04096700	-0.61438200

(Ph)HC=C(PPh2)F.log

SCF (RB3PW91) = -1212.12987388

E(SCF)+ZPE(0 K)= -1211.829516

H(298 K)= -1211.80975

G(298 K)= -1211.880408

Lowest Frequency = 17.1221cm-1

C	0.99462900	-1.47085600	-1.09699000
H	1.43174900	-2.26133500	-0.48039900
C	0.41644600	-0.48756900	-0.38074100
F	-0.17756500	0.56644800	-0.99653300
C	1.01397400	1.18387700	1.79291800
C	2.28354300	1.28969400	2.37913100
C	0.30392600	2.35620200	1.48554100
C	2.83983900	2.54260100	2.64755400
H	2.84019700	0.38171800	2.62849300
C	0.85875300	3.60590600	1.75608500
H	-0.68890800	2.28545200	1.03626300
C	2.12755800	3.70154500	2.33637300
H	3.82976200	2.61204300	3.10577700
H	0.29893400	4.51252500	1.51168300
H	2.55908900	4.68324700	2.54872700
C	-1.41102500	-0.32139500	1.75037000
C	-2.36250600	-0.88903500	0.88963800
C	-1.85146800	0.29121300	2.93505700
C	-3.72207100	-0.83538800	1.20109100
H	-2.04236000	-1.37481600	-0.03621700
C	-3.21052700	0.34420100	3.24391300
H	-1.12590800	0.74125500	3.61840400
C	-4.15133700	-0.21810400	2.37754600
H	-4.45078200	-1.27548000	0.51520100
H	-3.53657500	0.83010400	4.16736000
H	-5.21646600	-0.17382000	2.61855900
P	0.38569700	-0.50255700	1.44115700
C	1.12366900	-1.64809500	-2.54354300
C	0.64332500	-0.73083200	-3.50113200
C	1.76924400	-2.81240900	-3.00697600
C	0.80636600	-0.97804700	-4.86245500
H	0.14179300	0.18042100	-3.17859100
C	1.92991600	-3.05614300	-4.36877400
H	2.14985700	-3.53623000	-2.28086600
C	1.44796300	-2.13814700	-5.30422400
H	0.42746200	-0.25399800	-5.58843600
H	2.43418900	-3.96694700	-4.70156100
H	1.57212100	-2.32512300	-6.37389800

H(Ph)C=C(PPh2)F.log

SCF (RB3PW91) = -1212.12494298

E(SCF)+ZPE(0 K)= -1211.825236

H(298 K)= -1211.805583

G(298 K)= -1211.873759

Lowest Frequency = 30.6746cm-1

C	-0.22190900	-0.34606200	-2.39003600
C	0.72709200	0.25355300	-1.65724800
F	1.43665800	1.25985000	-2.22336800
C	2.01855100	1.33613200	0.64018900
C	3.24510800	1.68217400	0.04630300
C	1.55577200	2.09972900	1.72235700
C	3.96830800	2.78450900	0.49767200
H	3.63813000	1.08579200	-0.78112800
C	2.29090700	3.19455800	2.18299700
H	0.61602700	1.84173900	2.21469500
C	3.49350700	3.54580400	1.56923800
H	4.91321700	3.04611300	0.01429600
H	1.91495500	3.77813300	3.02758700
H	4.06410300	4.40626700	1.92794900
C	-0.46702500	-0.12832900	0.83984300
C	-1.34847100	0.94696600	0.64295800
C	-0.88990400	-1.22855200	1.59446700
C	-2.62585200	0.92063600	1.19671500
H	-1.03201700	1.80307000	0.04155000
C	-2.17454900	-1.25934200	2.14206400
H	-0.21752600	-2.07928500	1.73089900
C	-3.04198100	-0.18549200	1.94513500

H	-3.30564100	1.76179100	1.03737800
H	-2.49971100	-2.13101600	2.71520500
H	-4.04915700	-0.21041400	2.36933800
P	1.19474200	-0.19868200	0.07575700
C	-1.03088300	-1.48218800	-1.89895700
C	-2.41899000	-1.34670000	-1.74007000
C	-0.42728800	-2.69909500	-1.54609300
C	-3.17736200	-2.38959500	-1.21206000
H	-2.89772600	-0.39986000	-2.00142700
C	-1.18806400	-3.74502000	-1.02262600
H	0.65066700	-2.81837000	-1.67937900
C	-2.56394200	-3.59105000	-0.84761600
H	-4.25379400	-2.26046500	-1.07386000
H	-0.70127900	-4.68457800	-0.74838100
H	-3.15972400	-4.40652600	-0.42959500
H	-0.42872800	0.06589500	-3.38471500

Int-3_gauche.log

SCF (RB3PW91) = -1609.4484

E(SCF)+ZPE(0 K)= -1608.898233

H(298 K)= -1608.864593

G(298 K)= -1608.965212

Lowest Frequency = 10.6413cm-1

C	1.99082600	-1.11831800	1.67750800
H	1.84649900	-1.49128500	2.70567300
H	2.60325100	-0.20679400	1.75834600
C	-2.56390800	0.26763500	4.44595400
H	-2.96697700	1.24717200	4.15365000
H	-1.68449300	0.44533900	5.08197900
H	-3.33358100	-0.24985500	5.05500400
C	-1.60900600	-1.79214000	3.66718700
H	-2.34011300	-2.42550300	4.20981700
H	-0.74118700	-1.63048600	4.32334500
H	-1.26128000	-2.32778400	2.77395600
C	-3.29219100	-0.68290600	2.33463100
H	-4.21835500	-0.99786100	2.85860200
H	-3.02373600	-1.50193700	1.65166600
C	-3.56162800	0.57575800	1.51947300
H	-4.45865000	0.42116400	0.88639500
H	-3.81125700	1.40434500	2.19776600
C	-2.32260900	0.19136700	-0.52950500
H	-2.35323900	-0.88512300	-0.31752200
H	-1.36004700	0.38127300	-1.02230300
H	-3.15220200	0.44173500	-1.22160800
C	-2.29009500	2.39146800	0.48422000
H	-3.25314800	2.83883400	0.16265600
H	-1.59212500	2.54480800	-0.35117000
C	-1.78225100	3.13371700	1.71488800
H	-1.68089400	4.21222500	1.46924100
H	-2.53080700	3.07367900	2.52103400
C	-0.23188200	3.12202800	3.55623200
H	-0.06435400	4.21850700	3.53729500
H	0.67343800	2.64512700	3.95968200
H	-1.06285600	2.91538900	4.24599200
C	0.59509500	2.80583000	1.32520400
H	0.75048000	3.88442600	1.11760200
H	0.44628300	2.27155400	0.37819100
H	1.51266900	2.39917000	1.77183600
Li	-0.78347000	0.48249400	2.00641600
N	-2.17670600	-0.50486600	3.27202500
N	-2.39630100	0.95137800	0.71491800
N	-0.52886900	2.58050800	2.23552100
C	0.66359600	-0.70510800	1.07960600
F	0.98403600	-0.14374200	-0.20059500
F	-0.00555300	-1.92686700	0.69895000
P	3.04896800	-2.33816600	0.71964400
C	4.67455600	-1.90897100	1.46767800
C	5.57463800	-1.16026400	0.69371200
C	5.03403600	-2.22965300	2.78817700
C	6.79630700	-0.73602800	1.22284300
H	5.31245800	-0.90988600	-0.33868500
C	6.25598100	-1.81355000	3.31589500
H	4.34998800	-2.81857700	3.40517800
C	7.14127800	-1.06349600	2.53521300
H	7.48498000	-0.15500100	0.60322200
H	6.52061400	-2.07480100	4.34448600
H	8.09985200	-0.73948100	2.94938800

C	2.71483500	-3.93570200	1.55433100
C	3.68592300	-4.95164100	1.47242800
C	1.50296100	-4.23020800	2.19783700
C	3.46197900	-6.20789500	2.03196400
H	4.63728200	-4.74891300	0.97165600
C	1.27559400	-5.49262100	2.75299100
H	0.72142400	-3.47215200	2.25191700
C	2.25275300	-6.48535500	2.67751600
H	4.23584900	-6.97743900	1.96236300
H	0.32291200	-5.69889600	3.24924900
H	2.07446500	-7.47141700	3.11462500

Int-4.log

SCF (RB3PW91) = -1609.42212159

E(SCF)+ZPE(0 K)= -1608.874004

H(298 K)= -1608.839612

G(298 K)= -1608.938611

Lowest Frequency = 21.8013cm-1

C	0.68785500	-2.55727400	-0.36311700
H	1.36284700	-3.29576700	-0.82371900
H	-0.31034600	-2.96492900	-0.16039600
C	0.65439400	-1.24034800	-0.95250600
F	1.76280500	-1.09740500	-1.69212800
C	2.79283800	-1.17120500	1.30494300
C	3.97198400	-1.79233600	1.75915200
C	2.85113900	0.15920200	0.84940400
C	5.18685200	-1.10602200	1.76369000
H	3.93318700	-2.82600700	2.11556800
C	4.06981800	0.83871900	0.86328700
H	1.96256200	0.69162600	0.47522200
C	5.23542800	0.21480100	1.31558000
H	6.09281700	-1.60353300	2.11926700
H	4.10180000	1.87261800	0.50994900
H	6.18406500	0.75880400	1.31776300
C	-0.04658100	-1.36536500	2.21860300
C	-0.25033000	0.02234400	2.27303700
C	-0.95631200	-2.21522800	2.87439900
C	-1.34092000	0.53483900	2.97856400
H	0.39013900	0.71801000	1.72483200
C	-2.05926200	-1.69789500	3.55408400
H	-0.79464100	-3.29770000	2.85245400
C	-2.24828000	-0.31582900	3.61449500
H	-1.48318400	1.61745200	3.02061000
H	-2.76043600	-2.37394200	4.04997300
H	-3.10167500	0.09780300	4.15846200
P	1.33297400	-2.25475300	1.41785000
C	-0.52996500	0.94244400	-4.13463300
H	-1.61626000	0.78742000	-4.08611300
H	-0.05928500	-0.04880700	-4.21108300
H	-0.30623300	1.50641900	-5.06577400
C	1.40327100	1.77771000	-2.99067800
H	1.71781900	2.47317900	-3.79767000
H	1.87877700	0.80515200	-3.17071600
H	1.74930700	2.15308700	-2.01777000
C	-0.67755900	2.92484400	-2.73639300
H	-0.58147000	3.56117300	-3.64365600
H	-0.12196300	3.41968500	-1.92731300
C	-2.14519000	2.83697800	-2.34735700
H	-2.58056900	3.85875100	-2.36010400
H	-2.69347300	2.27291900	-3.11473900
C	-2.13903900	3.13826100	0.04280000
H	-1.11998800	3.54012200	-0.00416300
H	-2.22140000	2.61067000	1.00242300
H	-2.87580100	3.97032300	0.02817200
C	-3.65305500	1.55068800	-0.94729100
H	-4.47859700	2.21533200	-1.28339000
H	-3.84644500	1.35007400	0.11682600
C	-3.72636400	0.24651300	-1.72853600
H	-4.75196600	-0.17401000	-1.61989000
H	-3.59831900	0.45219200	-2.80281400
C	-2.61036600	-1.79633400	-2.29841600
H	-3.54209400	-2.40009700	-2.36705700
H	-1.78845300	-2.47291600	-2.02318900
H	-2.38944700	-1.39561600	-3.29855500
C	-2.91776800	-1.22621600	0.00516300
H	-3.89774400	-1.73971500	0.11839200
H	-2.85901800	-0.42278100	0.75081900
H	-2.12760800	-1.94171300	0.26289900

Li	-0.47399700	0.75384300	-0.96794200
N	-0.04780600	1.61912100	-2.94184800
N	-2.35092500	2.19739800	-1.04814500
N	-2.70567000	-0.71461400	-1.33722900
F	0.55053600	1.83173500	-0.00742300

Int-5_anti.log

SCF (RB3PW91) = -1840.26918008

E(SCF)+ZPE(0 K)= -1839.637477

H(298 K)= -1839.599277

G(298 K)= -1839.708195

Lowest Frequency = 17.0643cm-1

C	1.99350900	-1.06025200	1.48499700
H	1.70584900	-1.57852100	2.41210100
C	-1.53577300	0.78516400	4.56467600
H	-2.07644200	1.68350100	4.23770200
H	-0.51349900	1.09062600	4.83131200
H	-2.03479400	0.40035400	5.47800500
C	-0.74588700	-1.38667900	3.94057800
H	-1.24336800	-1.90469800	4.78583800
H	0.26067100	-1.09005400	4.26632400
H	-0.63937200	-2.09054800	3.10417100
C	-2.82452200	-0.58851000	3.02904300
H	-3.50112000	-0.82710700	3.87609300
H	-2.71918000	-1.51373400	2.44341200
C	-3.45639000	0.48512200	2.15506000
H	-4.48657500	0.17827700	1.88277500
H	-3.56331200	1.41280700	2.73536600
C	-2.89016200	-0.20420600	-0.10122800
H	-2.71925800	-1.22459800	0.26651200
H	-2.17461000	-0.04217700	-0.91911300
H	-3.92183800	-0.14086000	-0.50350000
C	-2.77318200	2.14650500	0.50629900
H	-3.82432300	2.49944600	0.51593700
H	-2.45224900	2.18641500	-0.54401200
C	-1.91247300	3.09148700	1.33765100
H	-2.01323800	4.12273200	0.93688400
H	-2.29024400	3.12730100	2.37209500
C	0.23651300	3.39732300	2.40222100
H	0.29783400	4.48290700	2.18126000
H	1.25907500	2.99814600	2.46532800
H	-0.24058400	3.27575000	3.38560000
C	0.14133200	2.78850500	0.08572400
H	0.09351100	3.82669800	-0.30291400
H	-0.30991200	2.11015000	-0.64961200
H	1.19206100	2.49320300	0.18324900
Li	-0.63760800	0.56299300	1.67397600
N	-1.48761000	-0.20947700	3.49942200
N	-2.64907900	0.76220500	0.96478200
N	-0.51348400	2.66485600	1.38768800
C	0.68894400	-0.76111300	0.73951400
F	1.04599800	-0.34742000	-0.57826600
F	0.07894100	-2.03349400	0.48845400
P	3.26655300	-2.18694500	0.62782500
C	4.62738300	-1.98457700	1.85308900
C	5.66577800	-1.09166500	1.54718300
C	4.62574800	-2.60209600	3.11415600
C	6.65922800	-0.80014900	2.48301900
H	5.68134600	-0.60408900	0.56889500
C	5.62339300	-2.31981700	4.04744600
H	3.83116100	-3.30844400	3.36928800
C	6.64037200	-1.41258000	3.73769600
H	7.45273100	-0.09196600	2.22962600
H	5.60568800	-2.80890000	5.02563200
H	7.41922700	-1.18789900	4.47137500
C	2.76034700	-3.89744700	1.06522400
C	3.65144000	-4.90948200	0.66345900
C	1.60259000	-4.27336700	1.76000400
C	3.40462000	-6.24804900	0.96131500
H	4.55915500	-4.63757800	0.11562000
C	1.34899300	-5.61658300	2.05234300
H	0.87990300	-3.51770300	2.06393600
C	2.24783600	-6.60816500	1.65910200
H	4.11579700	-7.01530800	0.64334100
H	0.43737500	-5.88737400	2.59248500
H	2.04737200	-7.65791900	1.88941400
C	2.69146600	0.21938000	1.87895000
C	3.19186400	1.10750800	0.91325600

C	2.89549100	0.53379200	3.23019600
C	3.86919100	2.26866600	1.28854100
H	3.05172900	0.87132900	-0.14292100
C	3.57238800	1.69369100	3.61003300
H	2.54278200	-0.15999800	3.99795800
C	4.06294900	2.56874300	2.63863400
H	4.25211800	2.94322300	0.51787000
H	3.72625000	1.90911400	4.67078300
H	4.59785400	3.47567000	2.93216000

Int-5_gauche.log

SCF (RB3PW91) = -1840.27194319

E(SCF)+ZPE(0 K)= -1839.64015

H(298 K)= -1839.602214

G(298 K)= -1839.709503

Lowest Frequency = 17.7741cm-1

C	2.12950600	-0.13937200	1.89618600
H	2.71859700	-0.77601500	1.21005100
C	-2.70122300	-1.10120000	4.16493700
H	-3.31404100	-0.19033800	4.21522800
H	-1.92474600	-1.02759000	4.93986600
H	-3.35477000	-1.96531800	4.40612900
C	-1.23050700	-2.43269900	2.82096900
H	-1.83507100	-3.35945600	2.90100400
H	-0.51497200	-2.41204800	3.65268500
H	-0.65586300	-2.45080400	1.88576000
C	-3.04568600	-1.25678300	1.76528800
H	-3.86632400	-1.97625800	1.97031700
H	-2.52510700	-1.62245200	0.86815500
C	-3.63354500	0.11759400	1.47572500
H	-4.41234200	0.02701000	0.69124500
H	-4.15091100	0.49081100	2.37115900
C	-2.22466000	0.94901800	-0.31470300
H	-1.91490400	-0.08143700	-0.53011500
H	-1.35396000	1.58364500	-0.52560400
H	-3.05714900	1.22256000	-0.99480500
C	-2.93158900	2.45651600	1.44405700
H	-3.97090700	2.72301500	1.16123800
H	-2.27988000	3.12107500	0.85933700
C	-2.73537400	2.72997000	2.92947000
H	-2.96718500	3.79770600	3.13061900
H	-3.46175400	2.14474200	3.51602800
C	-1.35182400	2.43956300	4.86439500
H	-1.57940700	3.45442500	5.25008500
H	-0.35190300	2.15981300	5.22523700
H	-2.07886700	1.73598100	5.29566600
C	-0.35950300	3.23324000	2.82590400
H	-0.54102600	4.30638900	3.04047900
H	-0.29655100	3.08666000	1.74011800
H	0.62429100	2.95343500	3.22710200
Li	-0.98546100	0.53528100	2.34873800
N	-2.06814800	-1.23348300	2.85860400
N	-2.59731500	1.07840900	1.09041400
N	-1.39512800	2.37636500	3.40798400
C	0.77733900	0.24778700	1.26872400
F	1.05528000	1.25667800	0.29096700
F	0.47314700	-0.88381600	0.42160200
P	3.17109300	1.37474100	2.28499000
C	4.45711700	0.55008400	3.31218000
C	4.37426100	0.68128200	4.70540800
C	5.45195200	-0.27921700	2.77166600
C	5.24548400	-0.01843600	5.54247000
H	3.60285700	1.32377100	5.13813200
C	6.33073900	-0.97025200	3.60534200
H	5.53310300	-0.38932200	1.68699100
C	6.22568500	-0.84672900	4.99383500
H	5.15818500	0.08510800	6.62731300
H	7.09912600	-1.61502300	3.16977400
H	6.91068500	-1.39417400	5.64684900
C	4.14615100	1.66794300	0.76138800
C	5.19966700	2.59328200	0.87342500
C	3.91837300	1.06293700	-0.48252000
C	6.01327500	2.89139200	-0.21762200
H	5.39133400	3.07734200	1.83627200
C	4.73075600	1.36746100	-1.57855300
H	3.09430900	0.36073400	-0.60946600
C	5.78084500	2.27721400	-1.45193200
H	6.83138100	3.60812700	-0.10546800

H	4.53673600	0.88585200	-2.54092800
H	6.41467500	2.51113700	-2.31142500
C	1.90618400	-0.89145700	3.18354100
C	2.44615900	-2.16491100	3.39400500
C	1.17282800	-0.30484000	4.22777500
C	2.25687500	-2.83464300	4.60515000
H	3.02990200	-2.63394100	2.59792600
C	0.98446900	-0.96464900	5.44104700
H	0.76173100	0.69536300	4.08031400
C	1.52472800	-2.23907900	5.63452600
H	2.68808700	-3.82928700	4.74606500
H	0.41688200	-0.48062800	6.24089200
H	1.37992900	-2.76284800	6.58294100

Int-6.log

SCF (RB3PW91) = -1840.25671853
E(SCF)+ZPE(0 K)= -1839.624805
H(298 K)= -1839.586788
G(298 K)= -1839.694698
Lowest Frequency = 17.1009cm-1

C	1.19573000	-0.89630400	0.29478900
H	0.50917500	-1.75354800	0.34230000
C	0.57920200	0.04772400	-0.73770700
F	1.44471800	1.14154900	-0.89977200
C	1.92067200	1.24316500	2.26099900
C	3.16557800	1.40405500	2.89014100
C	1.26601800	2.38491200	1.76744700
C	3.75153000	2.66639600	3.01460700
H	3.68252300	0.52288700	3.28073200
C	1.84244400	3.64598800	1.90492000
H	0.31027000	2.27328600	1.25625300
C	3.08834100	3.79118100	2.52408300
H	4.72548400	2.76937900	3.50047200
H	1.32155800	4.52454300	1.51413500
H	3.54034400	4.78199000	2.62209800
C	-0.46025700	-0.40330300	2.62158400
C	-1.04950000	0.66036600	3.32420900
C	-1.23806800	-1.55933700	2.41372700
C	-2.37063600	0.58282700	3.77104900
H	-0.47178000	1.56349600	3.53015300
C	-2.56004200	-1.63375800	2.84989100
H	-0.79675300	-2.43041300	1.92074200
C	-3.13771800	-0.55860400	3.53147200
H	-2.80382100	1.42833200	4.31276300
H	-3.13914000	-2.54337700	2.66705600
H	-4.17261100	-0.61409800	3.87812800
P	1.30727300	-0.47781600	2.14403800
C	2.53582800	-1.42542600	-0.16143200
C	3.66566000	-0.59421400	-0.23206800
C	2.68445300	-2.77439300	-0.51445100
C	4.89955400	-1.09993600	-0.64170800
H	3.56983400	0.45860200	0.03703800
C	3.91725500	-3.28303100	-0.92770200
H	1.81897700	-3.44129800	-0.45686900
C	5.03202400	-2.44560000	-0.99303300
H	5.76648400	-0.43497600	-0.68687500
H	4.00670900	-4.33911000	-1.19703700
H	5.99994600	-2.83973300	-1.31414800
C	-1.73242000	1.18143200	-4.48562200
H	-2.58316400	0.49026200	-4.56171700
H	-0.83332000	0.64432600	-4.82058400
H	-1.91581200	2.02416500	-5.18404700
C	-0.40621400	2.53684100	-3.02163200
H	-0.55487500	3.45776100	-3.62217300
H	0.49802500	2.02417100	-3.37589400
H	-0.22989700	2.81338900	-1.97411900
C	-2.76252400	2.26387700	-2.57630600
H	-3.18369300	3.00600000	-3.28659300
H	-2.47422500	2.82448800	-1.67510200
C	-3.83106900	1.24365000	-2.20775800
H	-4.74951900	1.77416000	-1.88220200
H	-4.11575500	0.67476500	-3.10433100
C	-3.45481300	0.88809400	0.15530500
H	-2.92301100	1.84675900	0.19635100
H	-2.96760800	0.22873800	0.88330100
H	-4.50707000	1.05511600	0.46541700
C	-4.00523900	-0.99377500	-1.25205900

H	-5.10930900	-0.91487600	-1.33810300
H	-3.81340000	-1.51048700	-0.30004800
C	-3.47490300	-1.83336600	-2.40801100
H	-3.97877400	-2.82335600	-2.39883800
H	-3.74723500	-1.36211300	-3.36538100
C	-1.50871600	-2.48912300	-3.64450800
H	-1.86656400	-3.51836700	-3.85360500
H	-0.40929200	-2.50128000	-3.62132800
H	-1.82469300	-1.84229100	-4.47520600
C	-1.56626200	-2.80334500	-1.27289100
H	-1.99719200	-3.82504900	-1.31537800
H	-1.83661700	-2.35147400	-0.30871700
H	-0.47163900	-2.88138100	-1.30519700
Li	-1.22700300	-0.02765900	-1.79994400
N	-1.55208600	1.63380400	-3.11112000
N	-3.35914300	0.31387200	-1.18136300
N	-2.01833400	-1.96825700	-2.38168100
F	-0.56689200	0.68020400	-0.04868200

Int-7.log

SCF (RB3PW91) = -1212.0387728
E(SCF)+ZPE(0 K)= -1211.740279
H(298 K)= -1211.72028
G(298 K)= -1211.790348
Lowest Frequency = 22.9905cm-1

C	-0.49320700	-0.73180400	-1.23946100
H	-1.29996800	-1.47242500	-1.12060600
C	-1.11179500	0.57778100	-1.39189200
F	-0.27858500	1.42048700	-2.01193200
C	0.68418500	1.10766800	0.86242700
C	2.07916200	1.23238400	0.76434300
C	-0.08439800	2.23984700	1.17649500
C	2.69334800	2.47348900	0.94436200
H	2.68718700	0.34921800	0.54939300
C	0.53259200	3.47363000	1.37204200
H	-1.16969100	2.15138700	1.25894800
C	1.92049000	3.59463400	1.24933100
H	3.77943300	2.56049700	0.85848600
H	-0.07385200	4.34995300	1.61475500
H	2.39979000	4.56559600	1.39869500
C	-1.55495600	-0.56427900	1.58755900
C	-2.81586700	-0.12895900	1.14932100
C	-1.42394100	-1.07304700	2.89183000
C	-3.91762700	-0.20284900	2.00309900
H	-2.90870700	0.28411500	0.14107500
C	-2.52391500	-1.12608800	3.74798300
H	-0.45041600	-1.43241900	3.23936200
C	-3.77524000	-0.69455300	3.30261300
H	-4.89528500	0.13636100	1.65051000
H	-2.40482100	-1.51681000	4.76175200
H	-4.64079800	-0.74467200	3.96838000
P	0.00081200	-0.57106200	0.63193500
C	0.64410700	-1.26400600	-2.06559300
C	1.80811000	-0.52144200	-2.32208200
C	0.54207500	-2.55791200	-2.59774900
C	2.83747500	-1.06406000	-3.09205800
H	1.91365600	0.48493200	-1.91689900
C	1.57105600	-3.09860900	-3.36854300
H	-0.35662500	-3.15062500	-2.40403400
C	2.72408600	-2.35201700	-3.61883700
H	3.73641100	-0.47189500	-3.28223900
H	1.47136700	-4.10871500	-3.77418600
H	3.53220600	-2.77319000	-4.22227400

Int-8.log

SCF (RB3PW91) = -1212.04068442
E(SCF)+ZPE(0 K)= -1211.742379
H(298 K)= -1211.722432
G(298 K)= -1211.791834
Lowest Frequency = 16.4679cm-1

C	0.50737200	-0.47249300	-1.55780300
C	0.09473700	0.90040300	-1.79768700
F	1.13843000	1.55614400	-2.32772000
C	1.87110200	1.21358200	0.59296800
C	3.05976700	1.38944900	-0.13747000
C	1.55265700	2.14019100	1.59850600

C	3.88857900	2.48198200	0.10668500
H	3.33829100	0.67076600	-0.91260700
C	2.39285400	3.22652700	1.84872500
H	0.64833600	2.01423000	2.19650500
C	3.55739500	3.40606200	1.10118900
H	4.80146500	2.60996200	-0.48045800
H	2.12976600	3.93983400	2.63409800
H	4.20932300	4.26141800	1.29529400
C	-0.68848500	-0.08541200	1.12797100
C	-1.50225100	1.05277500	0.99083000
C	-1.15680800	-1.17378400	1.87927000
C	-2.75409500	1.09507400	1.59995800
H	-1.15940800	1.88643900	0.37577300
C	-2.41571800	-1.13230000	2.48192500
H	-0.53569800	-2.06824100	1.97708300
C	-3.21416500	0.00264900	2.34305600
H	-3.38295500	1.98143100	1.48325000
H	-2.77268000	-1.99073200	3.05613500
H	-4.20166200	0.03751400	2.81075300
P	0.93397100	-0.32841300	0.34173500
C	-0.54446500	-1.54900500	-1.62020400
C	-1.91450500	-1.25150900	-1.58194000
C	-0.14187400	-2.89093500	-1.66412700
C	-2.85740300	-2.27842100	-1.58340400
H	-2.23099500	-0.20842600	-1.54056600
C	-1.08729700	-3.91704800	-1.66917100
H	0.92450200	-3.13331000	-1.68748200
C	-2.44939500	-3.61357600	-1.62529700
H	-3.92154600	-2.03142800	-1.54906200
H	-0.75682500	-4.95830100	-1.70578200
H	-3.19145300	-4.41616300	-1.62563600
H	1.45549300	-0.78213300	-2.03130100

LiFPMDETA.log

SCF (RB3PW91) = -628.174182206

E(SCF)+ZPE(0 K)= -627.843858

H(298 K)= -627.825515

G(298 K)= -627.887021

Lowest Frequency = 56.5142cm-1

C	3.89475400	3.18526400	0.91892700
H	3.01382000	2.93200100	1.52478500
H	4.45056600	2.25438800	0.73515700
H	3.53637900	3.57467700	-0.05707000
C	5.93518600	4.44039600	0.82357700
H	5.68620800	4.93897400	-0.13613200
H	6.47633000	3.50992700	0.60070800
H	6.61334800	5.08496100	1.39928600
C	4.02381700	5.35841400	1.98383700
H	3.42510800	5.75189400	1.13529900
H	4.77009200	6.13115600	2.22084800
C	3.12138100	5.14930100	3.19349300
H	2.53293200	6.07093900	3.37967500
H	2.38570800	4.36496500	2.96619300
C	4.47033600	5.90098200	5.05918100
H	5.06833500	6.49549900	4.35516500
H	5.15437000	5.55715800	5.84745900
H	3.70275600	6.56392600	5.50966200
C	3.12611300	3.90261000	5.29302200
H	2.11121200	4.30377700	5.49587000
H	3.64817100	3.90181100	6.26074800
C	3.00260600	2.47158000	4.78172400
H	2.45061300	1.86875600	5.53492600
H	2.38587900	2.45578400	3.86904000
C	4.12840300	0.64295000	3.69237200
H	3.61104300	-0.14871500	4.27371300
H	5.11286200	0.25745200	3.38924300
H	3.54241600	0.83550000	2.78210900
C	5.11776300	1.63701000	5.63548300
H	4.62523900	0.94642700	6.35156200
H	5.34284000	2.58079500	6.14830800
H	6.08235000	1.21154700	5.33027400
Li	5.46927800	3.47300800	3.55867400
N	4.74678700	4.13870700	1.61618700
N	3.88482600	4.75358100	4.37841000
N	4.29560300	1.87467300	4.45130300
F	7.09713700	3.60755700	3.96272200

TS-6.log

SCF (RB3PW91) = -1609.41763907

E(SCF)+ZPE(0 K)= -1608.86928

H(298 K)= -1608.835934

G(298 K)= -1608.932317

Lowest Frequency = -79.4319cm-1

C	-0.73043000	-1.02903000	-1.70077200
H	0.05235800	-1.72769000	-2.04390900
H	-1.66932900	-1.57721300	-1.53911900
C	-0.94900600	0.05616600	-2.67370900
F	0.21753000	0.28928400	-3.27337400
C	1.37964500	0.35889600	-0.19700700
C	2.54964700	-0.24339500	0.29630800
C	1.45941000	1.61157300	-0.83136000
C	3.78668100	0.38862000	0.15697600
H	2.48803800	-1.21519200	0.79551900
C	2.69983800	2.23723900	-0.96275500
H	0.53840900	2.07695900	-1.21996200
C	3.86145500	1.63186600	-0.47394600
H	4.69064700	-0.08889300	0.54412600
H	2.75903500	3.21087200	-1.45740100
H	4.82833300	2.13106500	-0.58387800
C	-1.41225400	0.56997700	0.66511900
C	-1.09098000	1.78839200	1.27882400
C	-2.74612900	0.13125400	0.70635600
C	-2.07768100	2.54887200	1.90660200
H	-0.06326500	2.15390400	1.26137900
C	-3.73545100	0.89931000	1.31849100
H	-3.02111500	-0.83109500	0.26611900
C	-3.40298400	2.11193000	1.92801500
H	-1.80722500	3.49892800	2.37466300
H	-4.76874600	0.54184600	1.33209700
H	-4.17457600	2.71343800	2.41516300
P	-0.15196900	-0.59714400	0.05007800
C	-2.33366700	2.91430700	-6.01934100
H	-3.43225500	2.91584600	-6.00614700
H	-2.00775000	1.94566700	-6.42591700
H	-2.00296300	3.71047900	-6.72089300
C	-0.33274500	3.03661400	-4.71918200
H	0.10176000	3.87283800	-5.30750500
H	-0.01011100	2.09048900	-5.17563900
H	0.04922400	3.06389800	-3.69014300
C	-2.23434400	4.34396700	-4.06348300
H	-2.05919500	5.20975600	-4.73928700
H	-1.61609600	4.49226000	-3.16652800
C	-3.69849500	4.31352900	-3.64782700
H	-4.01170100	5.33506400	-3.34748800
H	-4.32292000	4.05619900	-4.51559900
C	-3.67825100	3.92227500	-1.26404200
H	-2.64390300	4.28477700	-1.22439100
H	-3.75243100	3.14176400	-0.49611300
H	-4.37549800	4.75007600	-1.01142500
C	-5.26001900	2.72609600	-2.65470100
H	-6.07307600	3.45751200	-2.85107700
H	-5.48410000	2.28722200	-1.67115000
C	-5.29982400	1.63672600	-3.71988500
H	-6.31917000	1.19320000	-3.75485800
H	-5.12921500	2.08803500	-4.70918100
C	-4.05681900	-0.18255500	-4.70494600
H	-4.94385500	-0.79312400	-4.97884000
H	-3.20571500	-0.86033100	-4.54001600
H	-3.80962000	0.46508300	-5.55729500
C	-4.56135000	-0.22699300	-2.37003400
H	-5.51879800	-0.77993800	-2.48336700
H	-4.60717300	0.36412900	-1.44631300
H	-3.75401400	-0.96130900	-2.24744600
Li	-2.32237000	1.78972100	-2.91294800
N	-1.79140100	3.09244900	-4.68230700
N	-3.94807500	3.35225000	-2.57501100
N	-4.27152600	0.62189200	-3.51419800
F	-1.13012400	2.45172800	-1.79435200

TS-7.log

SCF (RB3PW91) = -981.216751841

E(SCF)+ZPE(0 K)= -981.001379

H(298 K)= -980.986216

G(298 K)= -981.045525

Lowest Frequency = -319.3936cm-1

C	5.35249300	-4.30344500	-0.67111900
H	6.28089200	-4.45281300	-1.24826300
H	4.70797600	-5.18315400	-0.56878500
C	4.77931500	-3.05456400	-0.39252200
F	5.56658200	-2.03396100	-0.86837800
C	6.44858700	-2.44568900	1.74414600
C	7.83437900	-2.46573800	1.53920400
C	5.84177500	-1.30725500	2.29248400
C	8.60662500	-1.35136500	1.86932900
H	8.31015200	-3.35732200	1.12114500
C	6.62069600	-0.20544000	2.64257000
H	4.75952100	-1.28612300	2.43913300
C	8.00173500	-0.22325700	2.42755800
H	9.68636600	-1.36943000	1.70101100
H	6.14500800	0.67824800	3.07562400
H	8.60810600	0.64532300	2.69693000
C	3.96544600	-3.89778300	2.31763000
C	2.75092300	-3.34834300	1.89293800
C	4.06191700	-4.46856900	3.59754500
C	1.64734400	-3.35452500	2.74731200
H	2.70578300	-2.91934600	0.88752600
C	2.96121200	-4.45782300	4.45387600
H	5.00484800	-4.91685700	3.92531200
C	1.75114800	-3.90294600	4.02814400
H	0.69983000	-2.92461000	2.41158200
H	3.04507900	-4.89329300	5.45287200
H	0.88479100	-3.90521300	4.69483400
P	5.49638100	-3.93263000	1.32592100

TS-8.log

SCF (RB3PW91) = -1840.2291126

E(SCF)+ZPE(0 K)= -1839.599029

H(298 K)= -1839.561013

G(298 K)= -1839.668322

Lowest Frequency = -103.0657cm-1

C	-0.41718100	-0.71750000	-1.52489800
H	-0.99638600	-1.65333500	-1.53879600
C	-1.12622300	0.20330400	-2.47136400
F	-0.33055000	1.23412600	-2.73245100
C	0.10788800	1.35109500	0.57626100
C	1.23319000	1.52148500	1.40316000
C	-0.48835000	2.48139600	-0.01099500
C	1.75429400	2.79281300	1.64767000
H	1.70717100	0.64346800	1.85140200
C	0.03976000	3.74935700	0.23716700
H	-1.36192700	2.35564800	-0.66621600
C	1.15655700	3.91035800	1.06200300
H	2.62946900	2.90907900	2.29234900
H	-0.42822000	4.62349000	-0.22419000
H	1.56335600	4.90851000	1.24701100
C	-2.16115400	-0.52920000	0.81722200
C	-3.06659700	0.52155500	1.01581500
C	-2.58940400	-1.84605700	1.06524900
C	-4.37000100	0.25413800	1.43824300
H	-2.76602900	1.54776000	0.81870000
C	-3.89877200	-2.11465700	1.46287400
H	-1.88156800	-2.67289700	0.94567900
C	-4.79445000	-1.05958100	1.65203800
H	-5.06300700	1.08439300	1.59719400
H	-4.21533100	-3.14601700	1.63847200
H	-5.81903200	-1.26005800	1.97666000
P	-0.39755700	-0.38770700	0.35544800
C	1.00159900	-1.02842100	-1.96171500
C	2.03862100	-0.08610200	-1.87063400
C	1.29214800	-2.29884600	-2.47911100
C	3.33124500	-0.41611600	-2.27766500
H	1.83333100	0.90875800	-1.47330200
C	2.58610300	-2.62854100	-2.88545300
H	0.49300500	-3.04145100	-2.56021400
C	3.61146000	-1.68683200	-2.78554500
H	4.12731400	0.32843500	-2.19439600
H	2.79199700	-3.62602300	-3.28257900
H	4.62596400	-1.94177900	-3.10262900
C	-3.31710400	1.27550600	-6.00971900
H	-4.17703900	0.59550100	-6.08147200
H	-2.40603500	0.66724200	-6.10689200

H	-3.36290500	1.97494900	-6.87165100
C	-2.13818700	2.83483100	-4.62392000
H	-2.18028000	3.68311700	-5.33913600
H	-1.22780800	2.25633400	-4.82770300
H	-2.06407000	3.21960200	-3.59744800
C	-4.53090300	2.73651700	-4.49988300
H	-4.76624300	3.39012900	-5.36792800
H	-4.34281600	3.40250100	-3.64515400
C	-5.73397700	1.85904300	-4.18690900
H	-6.64407000	2.49399700	-4.14152400
H	-5.90114700	1.15954100	-5.01851900
C	-5.85747700	1.88490900	-1.77166600
H	-5.24811200	2.79713100	-1.76146400
H	-5.57775100	1.32076600	-0.87249100
H	-6.93126800	2.16511900	-1.71157800
C	-6.24617300	-0.18333300	-2.96855900
H	-7.28168300	-0.10651000	-3.36330100
H	-6.34734400	-0.52624800	-1.92870600
C	-5.48058500	-1.22568400	-3.77397900
H	-6.02818700	-2.19286100	-3.72849500
H	-5.46140500	-0.93373400	-4.83598400
C	-3.31729000	-2.16884100	-4.25479400
H	-3.66763100	-3.22123900	-4.31392600
H	-2.26214200	-2.16712500	-3.94319400
H	-3.36828800	-1.73392000	-5.26365400
C	-4.01270300	-1.90103800	-1.97591500
H	-4.54420000	-2.86971200	-1.86440400
H	-4.41570200	-1.19426400	-1.23868800
H	-2.96161100	-2.06026700	-1.71155500
Li	-3.24993200	0.68621600	-2.94926900
N	-3.30722700	1.96773600	-4.73008600
N	-5.55447700	1.09781500	-2.95501600
N	-4.09855100	-1.36661000	-3.32760800
F	-2.80936600	1.70539900	-1.59126300

TS-9.log

SCF (RB3PW91) = -1212.03627123

E(SCF)+ZPE(0 K)= -1211.738427

H(298 K)= -1211.71898

G(298 K)= -1211.788169

Lowest Frequency = -175.7397cm-1

C	0.06899600	-1.36845600	-2.33054200
H	-0.64189500	-2.18311400	-2.13233100
C	-0.55942800	-0.10682500	-2.14725900
F	0.16801200	0.91363100	-2.69081500
C	1.20571500	0.59713100	-0.09328600
C	2.59572700	0.64048700	-0.27782000
C	0.52298400	1.75343600	0.31240900
C	3.29240400	1.83418000	-0.07985100
H	3.13171400	-0.26503900	-0.57433400
C	1.22587100	2.93544500	0.53368100
H	-0.56076900	1.72173600	0.44468800
C	2.60920300	2.98000500	0.33084400
H	4.37410900	1.86422500	-0.23334300
H	0.69074600	3.83262500	0.85523300
H	3.15563800	3.91192500	0.49745400
C	-1.12539100	-0.99899700	0.66756800
C	-2.38290900	-0.50700300	0.29450200
C	-0.95242400	-1.55281700	1.94805500
C	-3.44714700	-0.55782100	1.19586300
H	-2.48962900	-0.08726300	-0.70952100
C	-2.01432600	-1.58427900	2.85188900
H	0.02174200	-1.95789400	2.23894200
C	-3.26518000	-1.08896900	2.47517200
H	-4.42659100	-0.17437600	0.89745300
H	-1.86712200	-2.00691300	3.84906600
H	-4.10131200	-1.12346400	3.17860300
P	0.38512200	-1.00069400	-0.35760600
C	1.24667400	-1.78590000	-3.13762000
C	2.29295100	-0.90820900	-3.47155100
C	1.32489500	-3.12285400	-3.56031600
C	3.38283600	-1.36462600	-4.21194000
H	2.25602500	0.13075800	-3.14664700
C	2.41156800	-3.57417100	-4.30735500
H	0.52161800	-3.81747500	-3.29864900

C	3.44554900	-2.69444400	-4.63473400
H	4.19036300	-0.67229900	-4.46330400
H	2.45354200	-4.61709800	-4.63137800
H	4.30072800	-3.04538600	-5.21799400

TS-10.log

SCF (RB3PW91) = -1212.03612694

E(SCF)+ZPE(0 K)= -1211.738757

H(298 K)= -1211.719258

G(298 K)= -1211.787471

Lowest Frequency = -271.008cm-1

C	-0.13290700	-1.25144800	-2.51633500
C	-0.36251100	0.12195400	-2.29062700
F	0.68004300	0.83751000	-2.83212400
C	1.29277700	0.57572600	-0.05231200
C	2.58946000	0.66488600	-0.58220100
C	0.84978900	1.55634200	0.84743900
C	3.42067200	1.72929300	-0.23890700
H	2.94820500	-0.10116100	-1.27516800
C	1.69395500	2.60899400	1.20374900
H	-0.15044400	1.49821300	1.27923200
C	2.97582900	2.70338300	0.65840600
H	4.42394000	1.79371000	-0.66764800
H	1.34186400	3.36502700	1.91042300
H	3.63011400	3.53390200	0.93524100
C	-1.26828600	-0.82069400	0.38741900
C	-2.20168200	0.22091200	0.26652200
C	-1.56553900	-1.92075900	1.20476900
C	-3.40141800	0.16809900	0.97143000
H	-1.98297200	1.04276900	-0.41808300
C	-2.77570800	-1.97818500	1.89917200
H	-0.85084400	-2.74464600	1.28210100
C	-3.69220600	-0.93268800	1.78492400
H	-4.12442700	0.98224300	0.87418800
H	-3.00395300	-2.84479700	2.52456400
H	-4.64213600	-0.97725100	2.32425800
P	0.32154400	-0.90068000	-0.48903900
C	-1.20569100	-2.26638400	-2.37200700
C	-2.55684400	-1.89031300	-2.31074800
C	-0.86670600	-3.62360900	-2.25290900
C	-3.54421700	-2.85116100	-2.10353100
H	-2.81304100	-0.83524100	-2.41754800
C	-1.85684400	-4.58348600	-2.05454700
H	0.18445900	-3.92116800	-2.29784700
C	-3.19775300	-4.19781600	-1.97181100
H	-4.59187400	-2.54609100	-2.04641700
H	-1.58215900	-5.63701000	-1.95981000
H	-3.97431200	-4.95022700	-1.81165000
H	0.76766500	-1.57762100	-3.06435200

References

1. J. Dunn and A. P. Dobbs, *Tetrahedron*, 2015, **71**, 7386-7414.
2. J. W. Bruno, G. M. Smith, T. J. Marks, C. K. Fair, A. J. Schultz and J. M. Williams, *J. Am. Chem. Soc.*, 1986, **108**, 40-56.
3. P. B. Hitchcock, M. F. Lappert, M. Linnolahti, J. R. Severn, P. G. H. Uiterweerd and Z.-X. Wang, *J. Organomet. Chem.*, 2009, **694**, 3487-3499.
4. T. Kawashima, N. Mitsuda and N. Inamoto, *Bull. Chem. Soc. Jpn.*, 2006, **64**, 708-710.
5. H. Sakaguchi, M. Ohashi and S. Ogoshi, *Angew. Chem. Int. Ed.*, 2018, **57**, 328-332.
6. M. Shimizu, T. Hata and T. Hiyama, *Bull. Chem. Soc. Jpn.*, 2001, **73**, 1685-1690.
7. S. L. Patrick, J. A. Bull, P. W. Miller and M. R. Crimmin, *Org. Lett.*, 2024, **26**, 8605-8609.
8. H. M. Dinh, Y.-T. He, R. R. Fayzullin, S. Vasylevskyi, E. Khaskin and J. R. Khusnutdinova, *Eur. J. Inorg. Chem.*, 2023, **26**, e202300460.
9. P. S. Pregosin, P. G. A. Kumar and I. Fernández, *Chem. Rev.*, 2005, **105**, 2977-2998.
10. O. V. Dolomanov, L. J. Bourhis, R. J. Gildea, J. A. K. Howard and H. Puschmann, *J. Appl. Crystallogr.*, 2009, **42**, 339-341.
11. G. Sheldrick, *Acta Crystallogr. A*, 2015, **71**, 3-8.
12. G. Sheldrick, *Acta Crystallogr. C*, 2015, **71**, 3-8.
13. J. P. Perdew, J. A. Chevary, S. H. Vosko, K. A. Jackson, M. R. Pederson, D. J. Singh and C. Fiolhais, *Phys. Rev. B*, 1992, **46**, 6671-6687.
14. J. P. Perdew, J. A. Chevary, S. H. Vosko, K. A. Jackson, M. R. Pederson, D. J. Singh and C. Fiolhais, *Phys. Rev. B*, 1993, **48**, 4978-4978.
15. J. P. Perdew, K. Burke and M. Ernzerhof, *Phys. Rev. Lett.*, 1996, **77**, 3865-3868.
16. A. D. Becke, *J. Chem. Phys.*, 1993, **98**, 5648-5652.
17. J. P. Perdew, K. Burke and Y. Wang, *Phys. Rev. B*, 1996, **54**, 16533-16539.
18. M. J. Frisch, G. W. Trucks, H. B. Schlegel, G. E. Scuseria, M. A. Robb, J. R. Cheeseman, G. Scalmani, V. Barone, G. A. Petersson, H. Nakatsuji, X. Li, M. Caricato, A. V. Marenich, J. Bloino, B. G. Janesko, R. Gomperts, B. Mennucci, H. P. Hratchian, J. V. Ortiz, A. F. Izmaylov, J. L. Sonnenberg, Williams, F. Ding, F. Lipparini, F. Egidi, J. Goings, B. Peng, A. Petrone, T. Henderson, D. Ranasinghe, V. G. Zakrzewski, J. Gao, N. Rega, G. Zheng, W. Liang, M. Hada, M. Ehara, K. Toyota, R. Fukuda, J. Hasegawa, M. Ishida, T. Nakajima, Y. Honda, O. Kitao, H. Nakai, T. Vreven, K. Throssell, J. A. Montgomery Jr., J. E. Peralta, F. Ogliaro, M. J. Bearpark, J. J. Heyd, E. N. Brothers, K. N. Kudin, V. N. Staroverov, T. A. Keith, R. Kobayashi, J. Normand, K. Raghavachari, A. P. Rendell, J. C. Burant, S. S. Iyengar, J. Tomasi, M. Cossi, J. M. Millam, M. Klene, C. Adamo, R. Cammi, J. W. Ochterski, R. L. Martin, K. Morokuma, O. Farkas, J. B. Foresman and D. J. Fox, *Journal*, 2016.
19. J. Tomasi, B. Mennucci and R. Cammi, *Chem. Rev.*, 2005, **105**, 2999-3094.
20. S. Grimme, J. Antony, S. Ehrlich and H. Krieg, *J Chem Phys*, 2010, **132**, 154104.
21. C. E. Dykstra, G. Frenking, K. S. Kim and G. E. Scuseria, 2005.
22. K. Fukui, *Acc. Chem. Res.*, 1981, **14**, 363-368.
23. G. Luchini, J. Alegre-Requena, I. Funes-Ardoiz and R. Paton, *F1000Res.*, 2020, **9**.
24. I. M. Alecu, J. Zheng, Y. Zhao and D. G. Truhlar, *J. Chem. Theory Comput.*, 2010, **6**, 2872-2887.
25. S. Grimme, *Chem. Eur. J.*, 2012, **18**, 9955-9964.
26. E. D. Glendening, C. R. Landis and F. Weinhold, *J. Comput. Chem.*, 2013, **34**, 1429-1437.
